# Critical illness impairs coordinated myogenesis: a prospective longitudinal study of skeletal muscle progenitor cell biology using serial muscle biopsies (SATELLITE study)

**DOI:** 10.1186/s13054-026-06131-5

**Published:** 2026-06-16

**Authors:** Adéla Krajčová, Vlasta Němcová, Pavel Škrha, Lucie Genserová, Andrea Musilová, Michal Fric, Petr Waldauf, František Duška

**Affiliations:** 1 OXYLAB-Laboratory for Mitochondrial Physiology, Department of Anaesthesia and Intensive Care, Third Faculty of Medicine, Charles University and Kralovske Vinohrady University Hospital, Prague, Czech Republic; 2 Department of Biochemistry, Cell and Molecular Biology, Third Faculty of Medicine, Charles University, Prague, Czech Republic; 3 Department of Internal Medicine, Third Faculty of Medicine, Charles University and Kralovske Vinohrady University Hospital, Prague, Czech Republic; 4 Department of Pathology, Third Faculty of Medicine, Charles University and Kralovske Vinohrady University Hospital, Prague, Czech Republic

**Keywords:** Satellite cells, Myogenesis, Muscle stem cells, Skeletal muscle regeneration, Critical illness

## Abstract

**Background:**

Critical illness often causes prolonged weakness, possibly due to impaired skeletal muscle regeneration, but the timing and nature of satellite cell (SC) dysfunction remain unclear. We aimed to determine whether SC depletion and dysfunction are detectable early after intensive care unit admission and describe their pathophysiological nature.

**Methods:**

In this prospective single-centre observational cohort study, mechanically ventilated adults underwent paired vastus lateralis biopsies within 72 h of ICU admission and again after 7 and 180 days. Isolated satellite cells were studied for proliferation, differentiation and fusion, mitochondrial morphology, respiratory function, substrate oxidation, and selected signalling proteins.

**Results:**

We enrolled 20 healthy control subjects and 33 ICU patients. Twenty three ICU patients survived to day 7 with a repeat biopsy. During 7 days in ICU, the patient developed profound weakness (MRC score 16 [0–32]) and insulin resistance (whole body glucose disposal 4.0 [3.5–5.1] versus 13.8 [8.8–16.1] mg/kg/min in controls). Satellite cell number per fibre was similar in controls and patients at admission (0.106 [0.085–0.129] vs. 0.098 [0.056–0.125]) and after 7 days (0.084 [0.066–0.117]; paired *p* = 0.784). SC proliferation was lower in older patients (ρ=-0.68 and − 0.49) and associated with lower muscle strength (ρ = 0.55 and 0.62). Myogenic differentiation was transiently impaired at day 0 (fusion index 68.9% [66.3–71.7] vs. 74.0% [70.3–77.1] in controls; *p* = 0.029). Satellite cell bioenergetics and substrate preferences were broadly preserved. In contrast, a more fragmented mitochondrial phenotype was associated with lower proliferation, lower respiratory performance, and worse muscle strength (ρ≈-0.6 to -0.8), whereas more interconnected morphology was associated with better function (ρ ≈ 0.6–0.7). Out of 10 ICU survivors at day 180, only 7 attended follow up. In those, impaired SF-36 physical score (62.5 [55.0 to 75.0]) and SC proliferation capacity (~50 %), contrasted with improved insulin sensitivity and SC number per fiber (~71 % and ~95% of control values, respectively).

**Conclusions:**

Critical illness was associated with disturbed satellite cell regenerative programming and altered mitochondrial remodelling rather than early depletion of the satellite cell pool or overt bioenergetic failure. Age was a stronger predictor of early satellite cell dysfunction than disease severity.

**Trial registration:**

ClinicalTrials.gov, NCT05671614. Registered 4 January 2023.

**Supplementary Information:**

The online version contains supplementary material available at 10.1186/s13054-026-06131-5.

## Introduction

Critical illness profoundly disrupts skeletal muscle homeostasis, leading to acute atrophy [1], prolonged weakness [2] and impaired regenerative capacity in ICU survivors [3, 4]. Central to the regenerative failure is dysfunction of satellite cells (SC) - the resident muscle stem cells responsible for repair and hypertrophy following injury [5, 6]. In a pivotal human study, Dos Santos et al. [7] demonstrated that ICU survivors with persistent muscle atrophy exhibited a ~ 45% reduction in SC compared to those who recovered muscle mass, implicating SC depletion in chronic ICU-acquired weakness. The same group identified dysregulated expression of muscle regenerative genes and increased fibrotic remodelling in patients with sustained weakness, suggesting a disrupted regenerative milieu [3]. Animal models of critical illness reported mitochondrial dysfunction and impaired proliferation in SC, which could be partially rescued by mesenchymal stem cell therapy [8]. Chen et al. [9] linked SC senescence to sepsis-induced weakness. Nonetheless, the timing and nature of SC dysfunction during critical illness remain incompletely characterised.

In this study, we aimed to determine whether SC depletion and dysfunction are already detectable early after ICU admission or whether they develop later during the course of critical illness. Furthermore, we sought to establish whether functional alterations in SC reflect impaired proliferative capacity, defective myogenic differentiation, or both, and whether these defects are associated with altered cellular bioenergetic profiles or reliance to specific substrates. Using serial skeletal muscle biopsies and SC cultures obtained within 72 h of ICU admission and again after seven days and 6 months, we examined how critical illness alters satellite cell behaviour over time within the same individuals. We quantified satellite cell proliferation by cell-cycle analysis, assessed myogenic differentiation, fusion capacity, cellular bioenergetics including substrate utilization in vitro, and related these functional measures to patients age, disease severity, and muscle strength. We hypothesised declining SC number over ICU stay correlated with disease severity, and alteration of cellular bioenergetics in remaining SC.

## Methods

### Study design and setting

This prospective, single-centre observational cohort study was conducted at the Department of Anaesthesia and Intensive Care, Královské Vinohrady University Hospital, Prague. Critically ill patients were enrolled within 72 h of ICU admission and followed longitudinally with assessments at baseline and after seven days of ICU stay. The study was approved by the institutional Research Ethics Committee (EK-VP-41-2020), and written informed consent was obtained from patients or their legally authorised representatives. The study was preregistered at ClinicalTrials.gov (NCT05671614) and is reported in accordance with STROBE guidelines. Detailed laboratory protocols are provided in the Supplementary Appendix.

## Participants

Adult patients receiving mechanical ventilation were eligible if they had a sudden-onset critical illness (e.g. traumatic brain injury, multiple trauma, sepsis) and were expected to require at least seven days of intensive care. Exclusion criteria included anticipated survival of less than six months, poor premorbid functional status (ECOG ≥ 3) or unknown baseline function, known mitochondrial disease, endocrine crisis as the cause of admission, pregnancy, or contraindications to muscle biopsy (e.g. bleeding disorders). For the present analysis, only patients who survived to Day 7 and had paired muscle biopsies available at baseline and Day 7 were included. Demographic data and APACHE II scores (first 24 h) were collected at baseline, and SOFA scores were recorded daily. After seven days of ICU stay, a euglycemic hyperinsulinemic clamp (120 mIU/m² BSA·hour) was performed, and metabolised glucose normalized to body surface area (M-value) was recorded as a measure of whole-body insulin sensitivity [10, 11]. A control group, consisting of metabolically healthy volunteers (with no diabetes, endocrine or neuromuscular disease, or chronic medication; BMI < 30; ECOG 0–1), also underwent biopsy and euglycemic hyperinsulinemic clamp.

## Muscle ultrasound and muscle strength assessment

We used muscle ultrasound to measure a rectus femoris cross-sectional area by a linear probe (at the standardized mid-thigh position with the patient supine and knee extended). Muscle strength was measured using the MRC score protocol (Medical Research Council scale, sum across 6 muscle groups bilaterally, scored from 0 to 60 points by the same trained physician and nurse).

## Muscle biopsies and immunohistochemistry

Skeletal muscle biopsies were obtained from the vastus lateralis using the Bergström needle technique at baseline and again after seven days. *Cryo-sectioning and fixation.* The muscle tissue was snap-frozen in pre-cooled isopentane and transverse sections (~ 7 μm) were cut in a cryostat. After fixation in 4% paraformaldehyde, sections were blocked by 0.5% Triton and 1% bovine serum albumin. *Fibre-type distribution and SC number*. For determination of the SC number and categorization of fibers into type I and II, staining of Pax7⁺ positive nuclei, total number of nuclei, laminin and type II myosin was performed on the same section as previously described [12]. Microscopy images were obtained at × 20 magnification using the fluorescence microscope Zeiss Axio Scan.Z1. Images were analysed using a software program Image J (Fiji).

**Isolation and culturing of SC**. Second part of muscle tissue was used for isolation [13, 14] and purification of satellite muscle cells based on the gentle enzymatic digestion of tissue using collagenase and dispase followed by magnetic activated cell sorting as previously described [15]. Isolated cells were subsequently cultured under standard conditions in growth medium at 37 °C in a humidified atmosphere with 5% CO₂ until further analysis [15].

**Measuring proliferation and myogenic differentiation.** *Cell proliferation* was assessed by measuring the incorporation of the thymidine analogue BrdU into newly synthesized DNA of proliferating cells, using a BrdU Cell Proliferation ELISA colorimetric kit (11647229001; Merck Millipore, Darmstadt, Germany) according to the manufacturer’s instructions. Fusion index was determined as the measure of *myogenic differentiation.* At ~ 90% confluency, the growth medium for myoblasts was switched to DMEM with 2% Horse serum to induce differentiation into multinucleated myotubes. After 7–8 days, the myotubes were fixed with 4% formaldehyde and stained with DAPI and CellMask™ Green Actin Tracking Stain for visualization of nuclei and F-actin. Microscopy images were captured using a Leica TCS SP8 system (Leica Microsystems) at a 20x magnification. The fusion index was calculated as the ratio of at least 3 nuclei within differentiated myotubes over the total number of nuclei in the culture [16, 17]. Analysis was performed using a MATLAB-based software MyoCount [16].

## Bioenergetic profile analysis

*Extracellular Flux analysis.* Extracellular Flux Analyzer (Agilent Technologies Inc., Santa Clara, CA, USA) was used to measure oxygen consumption rate (OCR) in living cells seeded on 96-well plate at 37° C [18]. Before starting the extracellular flux assay, culture medium was replaced by the assay medium containing 10 mM Glucose, 1 mM Pyruvate and 2 mM Glutamine to perform experiment under conditions of saturating substrates [19]. Respiration in intact cells was firstly measured at the baseline. After that, substrate preference was determined using addition of specific inhibitors of oxidation pathways (pyruvate inhibitor carrier UK5099 for glycolysis; 2 µM), etomoxir (carnitine palmitoyl-transferase I inhibitor; 4 µM) and BPTES (glutaminase inhibitor; 3 µM). In the control group, medium with no inhibitor was added. Subsequently, oligomycin (1 µM) was added to block ATP synthase. Uncoupling agent carbonyl cyanide-4- (trifluoromethoxy)phenylhydrazone (FCCP; 1 µM) was then injected to determine maximal respiratory capacity. Finally, rotenone (rot; 3 µM) and antimycin A (AA; 4 µM) were added to block complex I and III and determine non-mitochondrial respiration. The sequential compound injections in the absence and presence of inhibitor (etomoxir or UK5099 or BPTES) allowed to measure acute response to an inhibitor, and maximal respiration [19]. 

*Mitochondrial membrane potential and reactive oxygen species (ROS) production.* Cells were stained with tetramethylrhodamine ethyl ester (TMRE; 7 nm) and MitoSOX™ Red superoxide indicator (1.25 µM) and imaged with a 63x water immersion objective (Leica TCS SP8 system, Leica Microsystems) for membrane potential and ROS production, respectively, as previously described [20, 21]. Prior to staining with TMRE, cells were labelled with MitoTracker™ Green FM (MTG; 200 nm) to normalize the values on mitochondrial volume. Mitochondrial membrane potential and ROS production were calculated as a ratio of TMRE or MitoSOX™ to MTG fluorescence intensity, respectively. All the images were analysed with Image J (Fiji).

### Mitochondrial morphology

MitoTracker™ Green FM labelled mitochondria were used for analysis of mitochondrial network architecture. Parameters of mitochondrial morphology (individuals, networks, mean branch length, mean network size, footprint) were analysed with ImageJ™ plugin “MINA” in a software program Image J (Fiji) [22]. This plugin recognizes only two types of mitochondrial structures in a skeletonized image: networks and individuals. Individuals are punctate (a single pixel in the skeletonized image) and rods (unbranched structures with two or more pixels in the skeletonized image) [22]. Networks are mitochondrial structures with at last a single node and three branches [22]. A higher number of individuals together with a higher number of small networks indicates a more fragmented mitochondrial reticulum. Conversely, a smaller number of larger networks (greater median network size) and longer mean branch length indicate a more interconnected, fused phenotype.

## Western blots

The following antibodies were used for Western blot analysis: Lipin1, Fatty Acid Synthase (FASN), Phospho-ATP-Citrate Lyase, ATP-Citrate Lyase, Phospho-AMPK alpha (Thr172) and Tom20, Tom40, OPA1, DRP1, Phospho-DRP1 (Ser616), Phospho-DRP1 (Ser637), Mitofusin-1 and Mitofusin-2 from Mitochondrial Dynamics Antibody Sampler Kit II (#74792,Cell Signalling), anti-GAPDH antibody. For more details see Supplementary Appendix.

## Variables and outcomes

The primary outcomes were changes in SC proliferative activity and fusion capacity between baseline and day 7. Secondary variables included in situ SC abundance, muscle fibre morphology, and substrate-specific oxidative capacity. The paired study design allowed within-patient comparisons over time.

### Statistical analysis

Analyses were performed in the per-protocol cohort of patients with paired biopsies at baseline and day 7, with healthy controls used for cross-sectional comparisons. Continuous variables are reported as mean ± standard deviation or median [interquartile range], as appropriate, and categorical variables as counts (%). Between-group comparisons (ICU patients versus healthy controls) and within-patient paired comparisons (day 0 versus day 7) were performed using parametric or non-parametric tests according to data distribution and pairing. For selected longitudinal variables, paired changes are presented as mean differences with 95% confidence intervals. Because satellite-cell proliferation slopes were non-normally distributed, they are reported as medians [interquartile range] and analysed using non-parametric methods. Associations between satellite-cell variables, muscle morphology, age, disease severity, mitochondrial markers, and clinical phenotypes were assessed using Spearman rank correlation. Given the limited number of observations at day 180, follow-up data were summarised descriptively and no formal inferential analyses were emphasised for that time point. Missing data were not imputed. All tests were two-sided, and a p value < 0.05 was considered statistically significant. Analyses were performed in R.

Given the exploratory mechanistic design and the limited feasibility of serial muscle biopsies in critically ill patients, no formal a priori sample size calculation was performed and the sample size was pragmatic.

## Results

**Fig. 1:**
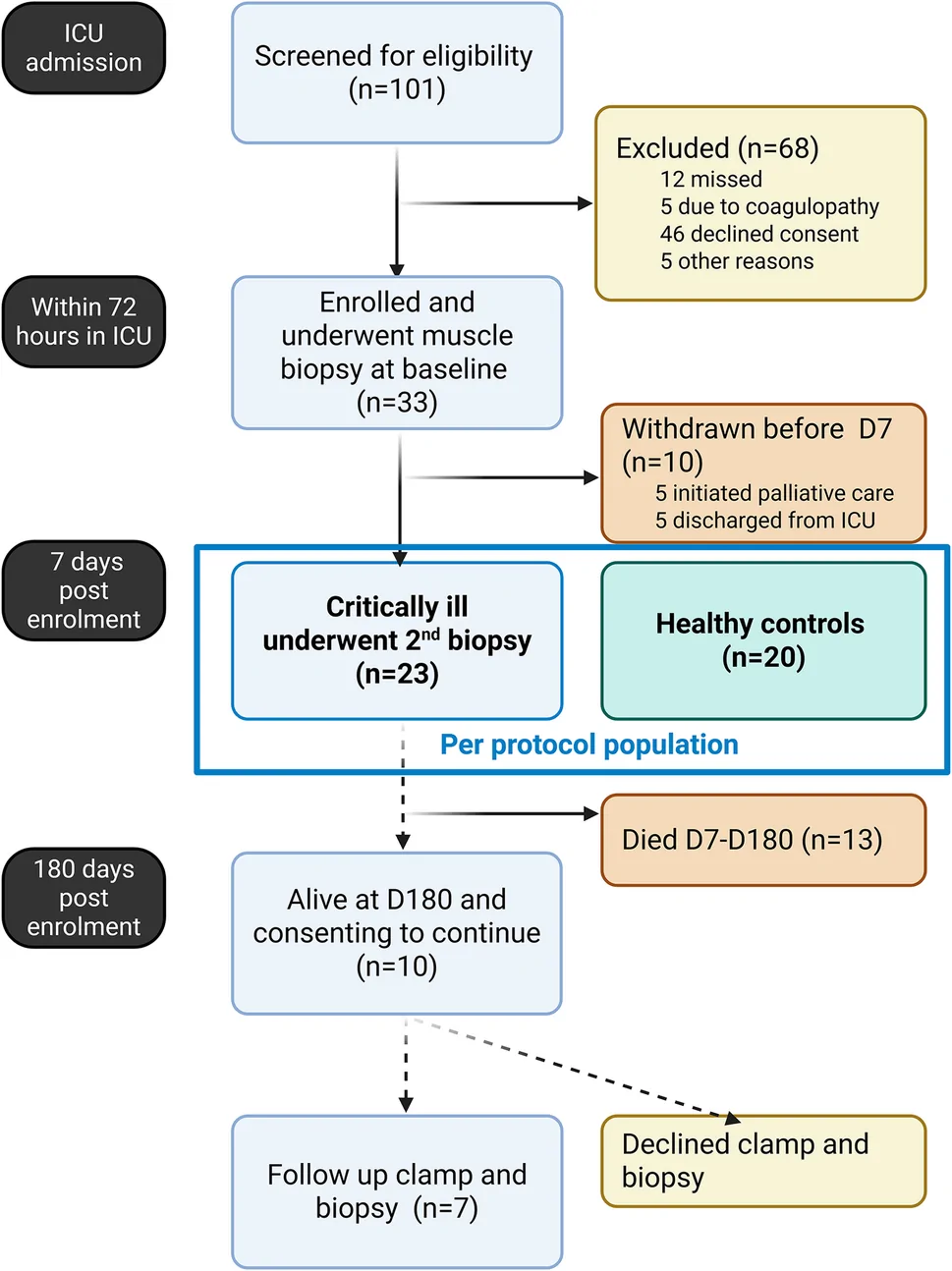
Study flowchart.

A total of 101 critically ill patients were screened for eligibility between May 2021 and December 2024. Of these, 68 were excluded, predominantly due to lack of informed consent obtained within 72-hour after ICU admission (see Fig. 1). The most common contributing factors were unavailability of legally authorized representatives in the early phase of admission, refusal of consent by the family, and rapid clinical deterioration that made the planned baseline biopsy unsafe. Thirty-three patients were enrolled and underwent skeletal muscle biopsy at baseline. Twenty-three patients survived at least 7 days and had the follow-up muscle biopsy and these patients form the *per protocol* population, in addition to 20 healthy subjects enrolled as the control group.

The demographic characteristic of both cohorts is given in Table 1.

**Table 1:** Baseline characteristics of study population (control and ICU group including comparison between survivors and non-survivors at day 180)

Characteristic	Controls (*n* = 20)	ICU patients (*n* = 23)		*p*-value
Survived to day 180	-	**Yes (n = 10)**	**No (n = 13)**	
Age, years	38.8 [33.5–44.1]	43.0 [38.0–49.0]	60.0 [56.0–68.0]	0.002
Female sex	4 (20%)	0 (0%)	2 (15%)	0.5
BMI, kg/m²	25.2 [23.3–28.7]	27.8 [27.7–30.5]	28.1 [25.7–34.0]	0.8
Diabetes mellitus	0 (0%)	1 (10%)	2 (15%)	˃ 0.9
APACHE II (day 0)	-	14.5 [13.0–16.0]	20.0 [17.0–23.0]	˂ 0.001
APACHE II (day 7)	-	10.0 [8.0–14.0]	20.5 [14.0–22.0]	0.003
SOFA (day 0)	-	9.0 [6.0–11.0]	11.0 [8.5–13.0]	0.2
SOFA (day 7)	-	4.0 [3.0–5.0]	7.5 [5.0–9.0]	0.010
Admission diagnosis: Traumatic brain injury Multiple trauma Sepsis Other	- - - -	4 (40%)	6 (46%)	˃ 0.9
		4 (40%)	4 (31%)	
		1 (10%)	2 (15%)	
		1 (10%)	1 (7.7%)	

Data are reported as median [IQR]. Note: BMI = body mass index, ICU = intensive care unit.

*Early preferential loss of fast-twitch fibre representation and profound weakness develop within the first week of critical illness.* At baseline, ICU patients did not differ from healthy controls in muscle fibre-type distribution, mean fibre cross-sectional area (CSA), or rectus femoris CSA on ultrasound (318.5 [226.3–377.0] vs. 285 [179.0–416.5] mm²; *p* = 0.658). By day 7, paired analyses showed directional reductions in both fibre thickness and fibre CSA, with a mean change from day 0 (D0) to day 7 (D7) of − 3.92 μm (95% CI − 8.98 to 1.15) and − 360 μm² (95% CI − 905 to 184) for type I fibres, and − 3.64 μm (95% CI − 9.84 to 2.56) and − 365 μm² (95% CI − 1054 to 323) for type II fibres. In parallel, fibre-type composition changed significantly, with a 14.8%-point decrease in type II fibre abundance (95% CI − 23.1 to − 6.4), mirrored by a corresponding increase in type I fibres, consistent with preferential loss of fast-twitch fibre representation during early critical illness. Ultrasound findings showed a similar direction of change, with rectus femoris CSA trending to decrease from 318.5 [226.3–377.0] mm² at ICU day 0 to 275.0 [179.5–334.0] mm² at day 7. This corresponds to 86% of the ICU day-0 median and 96% of the healthy-control median (285.0 [179.0–416.5] mm²) and in paired analyses, the mean decline in ICU patients was − 30.5 mm² (95% CI − 75.8 to 14.8; *p* = 0.187). See Figure S2 in Supplementary Appendix.

In contrast, muscle strength at day 7 was profoundly reduced in ICU patients (MRC score 16 [0–32]). Lower day-7 MRC scores were associated with older age (ρ = −0.73, *p* = 0.0004) and higher APACHE II score (ρ = −0.59, *p* = 0.007), but not significantly with SOFA score (ρ = −0.36, *p* = 0.190).

*SC abundance in skeletal muscle.* SC number per muscle fibre was similar in healthy controls and ICU patients at admission (controls 0.106 [0.085–0.129], *n* = 20; ICU D0 0.098 [0.056–0.125], *n* = 21; *p* = 0.246). In ICU patients, SC number per fibre remained unchanged over the first week (D7 0.084 [0.066–0.117], *n* = 20; paired D0 vs. D7 *p* = 0.784), although individual trajectories were heterogeneous, with 10 patients increasing and 10 decreasing. SC number per fibre was not associated with age or disease severity and did not differ significantly according to day 180 (D180) survival status. See Figure S2 in Supplementary Appendix.

*Satellite cell proliferation rate*. SC proliferation rate did not differ between healthy controls (median [IQR] of proliferation slope = 0.199 [0.164–0.315]) and ICU patients at admission (0.161 [0.146–0.238], *p* = 0.119) or after 7 days (0.173 [0.131–0.224], *p* = 0.173). Nonetheless, SC proliferation rate was highly heterogeneous and significantly lower in older ICU patients both at D0 (ρ = −0.68, *p* = 0.001) and at D7 (ρ = −0.49, *p* = 0.029). Associations with clinical severity were not statistically significant at D0 (SOFA: ρ = −0.28, *p* = 0.250; APACHE II: ρ = −0.37, *p* = 0.110) nor at D7 (SOFA: ρ = −0.46, *p* = 0.072). SC proliferation at both D0 and D7 was associated with muscle power measured as MRC score (ρ = 0.55, *p* = 0.023; and ρ = 0.62, *p* = 0.008). See Figures S3-S5 and S9 in Supplementary Appendix.

*Transient reduction of myogenic differentiation and loss of coordination of myogenic programming during critical illness.* Myogenic differentiation, assessed by fusion index after 7 days in differentiation medium, was transiently reduced in SC obtained early after ICU admission. Median fusion index was 74.0% (IQR 70.3–77.1; *n* = 19) in healthy controls, compared with 68.9% (66.3–71.7; *n* = 20) at ICU day 0 (*p* = 0.029) and 71.6% (66.6–73.9; *n* = 20) at ICU day 7 (*p* = 0.095 vs. controls). In healthy controls, SC proliferation correlated positively with fusion index (Spearman ρ = 0.57, *p* = 0.011), whereas no such association was observed in ICU patients at day 0 (ρ = −0.14, *p* = 0.565) or day 7 (ρ = −0.19, *p* = 0.431), suggesting disruption of coordinated myogenic programming during critical illness. Fusion index at ICU day 7 was not significantly associated with age (ρ = −0.17, *p* = 0.473) or disease severity.

**Fig. 2:**
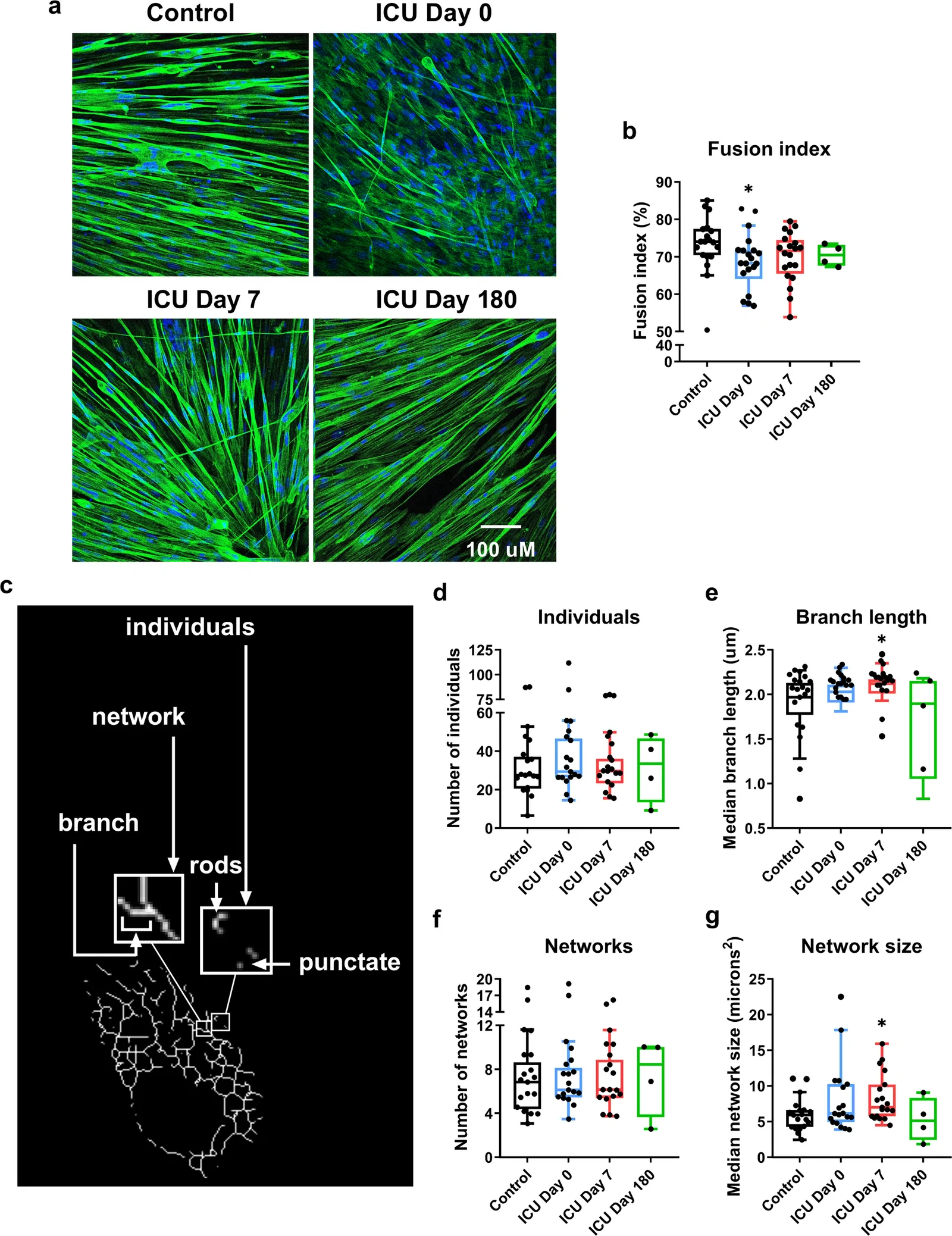
Mitochondrial network morphology in control SC and SC obtained from ICU patients over time**. (a)** Representative confocal images of myotubes stained with DAPI and CellMask™ Green Actin Tracking Stain for visualization of nuclei and F-actin. Scale bar = 100 μm; (**b)** Fusion index calculated as the ratio of at least 3 nuclei within differentiated myotubes over the total number of nuclei in the culture; (**c)** The schematic illustrates the image-analysis workflow and the morphometric features quantified: mitochondrial individuals, networks, and branches (segments within the skeletonized network). Individuals are punctate (a single pixel in the skeletonized image) and rods (unbranched structures with two or more pixels in the skeletonized image). Networks are mitochondrial structures with at least a single node and three branches. Scale bar = 10 μm. **(d-g)** Quantitative comparison of mitochondrial morphology across groups, including **(d)** number of individuals, **(e)** median branch length, **(f)** number of networks, and **(g)** median network size (number of branches per network). Box plots show the median and interquartile range, with whiskers indicating the spread of the data; individual dots represent individual samples. Statistical significance is indicated as *p < 0.05 compared to control group (healthy volunteers).

*Fragmented mitochondrial architecture is associated with impaired SC function and weakness.* Mitochondrial morphology of SC was quantified using descriptors of isolated mitochondrial structures (individuals), connected structures (networks), branch length, network size (number of branches), and mitochondrial footprint (see Fig.2). At baseline, mitochondrial morphology of ICU-derived SC was broadly similar to healthy controls. Median numbers of individuals were 27.7 [22.4–37.7] in controls and 31.7 [26.5–49.1] at ICU day 0 (*p* = 0.386), while networks were 7.0 [4.8–9.0] and 6.7 [5.6–8.7], respectively (*p* = 0.682). Likewise, mean branch length (2.5 [2.4–2.6] vs. 2.5 [2.4–2.6] μm, *p* = 0.574), median network size (6.0 [4.3–6.6] vs. 6.1 [5.2–10.1], *p* = 0.323), and mitochondrial footprint (264.2 [199.0–331.5] vs. 300.0 [218.4–419.7] μm², *p* = 0.421) did not differ significantly. Over the first week, mean branch length increased modestly from day 0 to day 7 (+ 0.1, 95% CI 0.0 to 0.2; *p* = 0.045). Compared with controls, ICU day 7-cells showed slightly higher median branch length (2.1 [2.0–2.2] vs. 2.0 [1.8–2.1] μm, *p* = 0.039) and median network size (7.0 [5.8–9.9] vs. 6.0 [4.3–6.6], *p* = 0.019).

Mitochondrial morphology was closely linked to satellite-cell function within the ICU cohort. At ICU day 0, a more fragmented mitochondrial phenotype- reflected by higher numbers of individuals and networks - was associated with older age, lower maximal respiration and reduced SC proliferation (ρ ≈ −0.6 to − 0.8, *p* < 0.001) and after 7 days fragmented phenotype was associated with lower muscle strength (MRC score; ρ ≈ −0.7 to − 0.8, *p* < 0.001). In contrast, features of a more interconnected network, including greater median network size and longer branch length, were associated with higher respiratory capacity, ATP production, and greater SC proliferation (ρ ≈ 0.6–0.7, *p* < 0.01) (see Fig. S10 in Supplementary Appendix). 

**Fig. 3:**
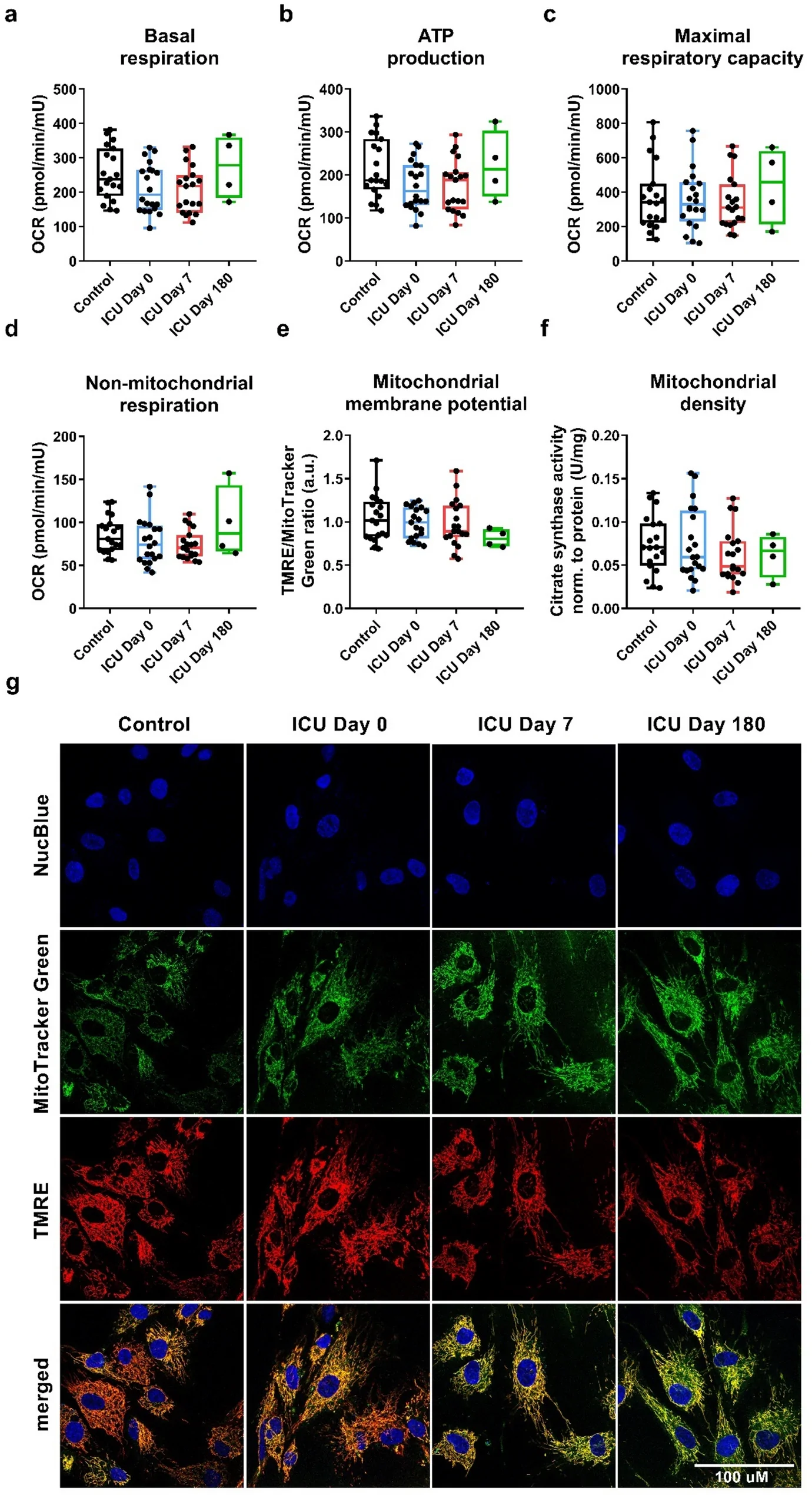
Mitochondrial respiratory function indices in SC from ICU patients and healthy controls.** (a–d)** Mitochondrial respiratory parameters, including **(a)** basal respiration, **(b)** ATP production, **(c)** maximal respiratory capacity, and **(d)** non-mitochondrial respiration, in healthy controls (*n* = 19) and ICU patients at day 0 (*n* = 20), day 7 (*n* = 20), and day 180 (*n* = 4). OCR values are normalized to CS activity. **e–f)** Quantification of mitochondrial membrane potential (TMRE/MitoTracker Green ratio) and mitochondrial density (citrate synthase activity normalized to protein content) in controls (*n* = 19), ICU patients at day 0 (*n* = 20), day 7 (*n* = 20) and day 180 (*n* = 4). **(g)** Representative fluorescence microscopy images of SC from controls and ICU patients (day 0, day 7, day 180), stained with NucBlue (nuclei, blue), MitoTracker Green (mitochondrial mass, green), and TMRE (mitochondrial membrane potential, red). Merged images are shown in the bottom row. Scale bar: 100 μm. Data are presented as box-and-whisker plots (median, interquartile range, and individual data points. Note: OCR, oxygen consumption rate; ICU, intensive care unit; a.u., arbitrary units.

*Core satellite-cell bioenergetics are broadly preserved despite profound systemic insulin resistance.* Specific citrate synthase activity, used as a proxy of total mitochondrial content, did not differ significantly between groups. Median values were 0.07 [0.05–0.10] U/mg in healthy controls, 0.06 [0.04–0.11] U/mg in ICU patients at day 0 (*p* = 0.768), and 0.05 [0.04–0.08] U/mg at day 7 (*p* = 0.189 vs. controls), with a non-significant downward trend from D0 to D7 (*p* = 0.066). Likewise, normalized ATP production, maximal respiratory capacity, and coupling efficiency were broadly preserved in ICU-derived SC. ATP production was 187.8 [170.9–275.6] pmol/min/mU in controls, 162.5 [132.6–222.8] pmol/min/mU at ICU day 0 (*p* = 0.084) and 188.7 [128.3–204.5] pmol/min/mU at day 7 (*p* = 0.122; paired *p* = 0.984). Maximal respiratory capacity was 342.5 [229.9–433.6] pmol/min/mU in controls, 329.3 [251.4–453.3] pmol/min/mU at ICU day 0 (*p* = 0.989) and 311.3 [221.8–408.5] pmol/min/mU at day 7 (*p* = 0.640; paired *p* = 0.651). Coupling efficiency was also similar across groups (*p* = 0.119–0.540) and unchanged over time (*p* = 0.332). Mitochondrial membrane potential was 1.016 [0.829–1.233] in controls, 0.995 [0.811–1.168] at ICU day 0 (*p* = 0.6) and 0.891 [0.822–1.188] at ICU day 7 (*p* = 0.5; paired = 0.56). See Fig. 3. ROS production tended to increase at ICU groups day 0 and 7 compared to control group, although these changes did not reach statistical significance; see Fig. S6 in Supplementary Appendix.

**Fig. 4:**
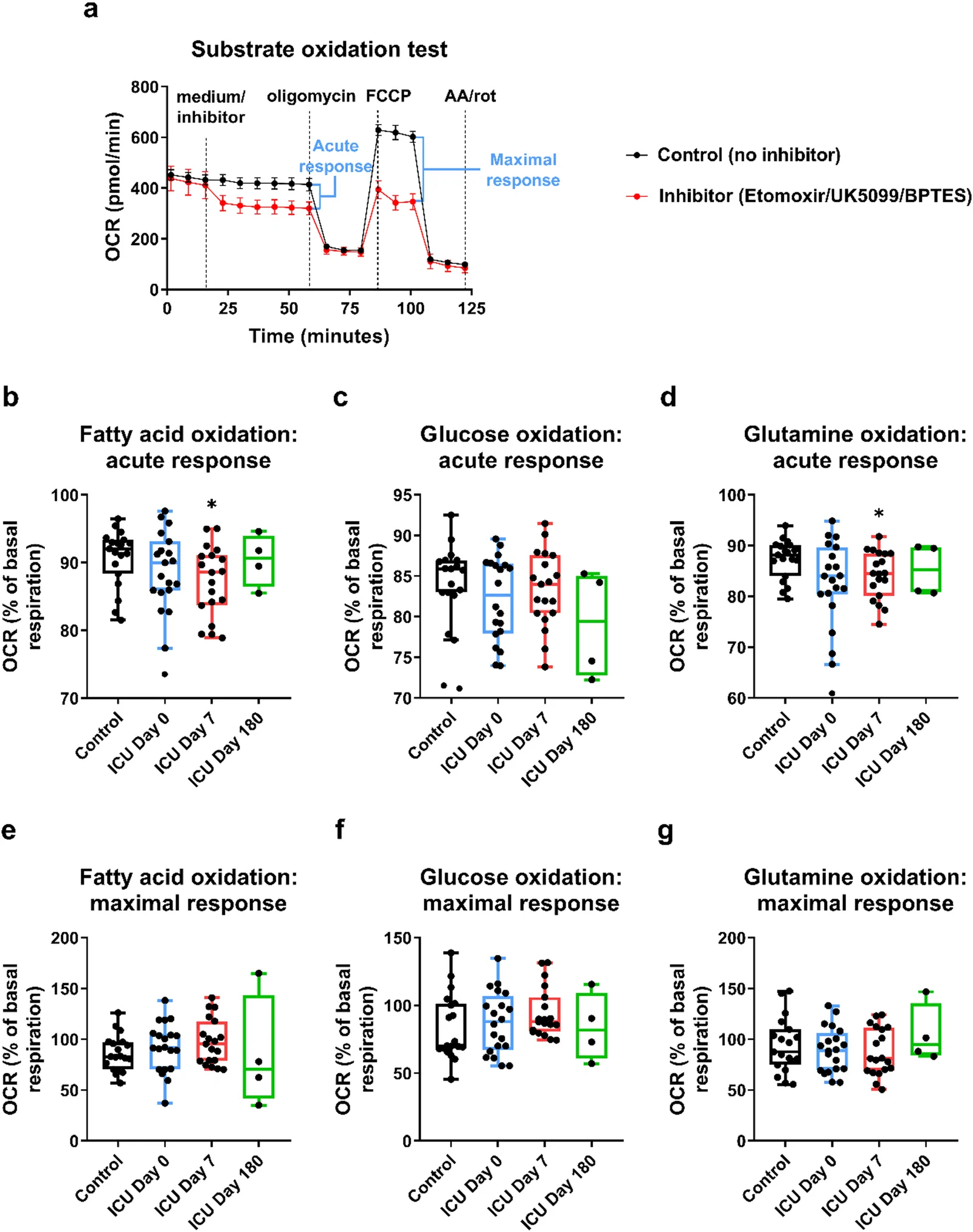
Substrate oxidation and mitochondrial respiratory responses in SC from ICU patients and healthy controls.** (a)** Representative oxygen consumption rate (OCR) trace during the substrate oxidation assay. Measurements were performed under control conditions (no inhibitor) and after inhibition of specific metabolic pathways (Etomoxir for fatty acid oxidation, UK5099 for pyruvate import, and BPTES for glutaminase). Sequential additions included oligomycin, FCCP, and antimycin A/rotenone (AA/rot), allowing assessment of acute and maximal respiratory responses. **(b–d)** Acute OCR response (expressed as % of basal respiration) to the respective inhibitors representing **(b)** fatty acid oxidation, **(c)** glucose oxidation, and **(d)** glutamine oxidation in controls (*n* = 19) and ICU patients at day 0 (*n* = 20), day 7 (*n* = 20) and day 180 (*n* = 4). **(e–g)** Maximal OCR response (expressed as % of control maximal respiration without inhibitor) to the respective inhibitors representing **(e)** fatty acid oxidation, **(f)** glucose oxidation, and **(g)** glutamine oxidation in controls (*n* = 19) and ICU patients at day 0 (*n* = 20), day 7 (*n* = 20) and day 180 (*n* = 4). Data are presented as box-and-whisker plots (median, interquartile range, and individual data points). Statistical significance is indicated as *p < 0.05 compared to control group (= healthy volunteers). Note: OCR, oxygen consumption rate; ICU, intensive care unit; FCCP, carbonyl cyanide-p-trifluoromethoxyphenylhydrazone (mitochondrial uncoupler); AA/rot, antimycin A and rotenone (inhibitors of complexes III and I, respectively); Etomoxir, inhibitor of carnitine palmitoyltransferase 1 (fatty acid oxidation); UK5099, inhibitor of mitochondrial pyruvate carrier; BPTES, inhibitor of glutaminase; FA, fatty acids.

Substrate-response profiles were largely stable during the first week of critical illness. Compared with controls, ICU-derived SC showed lower acute OCR responses to UK5099 at day 0 (84.9 [83.1–86.4]% vs. 86.2 [85.5–87.7]%; *p* = 0.048) and lower acute responses to etomoxir at both day 0 (81.5 [80.5–82.8]% vs. 84.2 [82.1–85.0]%; *p* = 0.019) and day 7 (81.5 [76.2–83.4]%; *p* = 0.041 vs. controls). However, maximal responses to UK5099 or etomoxir, as well as glutamine-related responses, did not differ significantly between groups, and none of the substrate-response variables changed significantly from D0 to D7 in paired analyses. See Fig. 4.

*Whole-body insulin sensitivity* was markedly reduced during early critical illness. M-value at day 7 was substantially lower in ICU patients than in healthy controls (4.0 [3.5–5.1] vs. 13.8 [8.8–16.1] mg/kg/min; *p* < 0.001). M-value was not significantly associated with age, BMI, or severity scores, and was not associated with the acute OCR response to UK5099 in either group.

*Western blot profiling links mitochondrial dynamics and lipid-synthesis signalling to satellite-cell phenotype.* To help explaining the heterogeneity in the functional bioenergetics and proliferation capacity of SC in ICU patients, we performed Western blotting to assess proteins related to mitochondrial content and protein import (Tom20, Tom40), mitochondrial fusion and network remodelling (Mfn1, Mfn2, OPA1), mitochondrial fission/fusion signalling (DRP1 phosphorylation markers), and metabolic/anabolic pathways (Lipin1, FASN, p-ATP citrate lyase). At ICU day 0, SC proliferation correlated positively with Mfn1, a mediator of outer mitochondrial membrane fusion (ρ = 0.8, *p* < 0.001), and with FASN, a key enzyme of de novo fatty-acid synthesis (ρ = 0.8, *p* < 0.001), and inversely with p-DRP1(S637)/DRP1, a marker linked to regulation of mitochondrial fission (ρ = −0.6, *p* = 0.003). Maximal respiratory capacity correlated positively with Mfn1 (ρ = 0.6, *p* = 0.009) and with p-ATP citrate lyase, which links mitochondrial metabolism to cytosolic acetyl-CoA production and anabolic signalling (ρ = 0.5, *p* = 0.022). By day 7, pro-fission phenotype (p-DRP1(S637) and p-DRP1(S637)/DRP1) remained inversely associated with both SC proliferation (ρ = −0.5 to − 0.6, *p* = 0.014 to 0.009), and MRC score (ρ = −0.6, *p* = 0.015 to 0.018), whereas p-DRP1(S616)/DRP1, a marker of mitochondrial fusion activation state, correlated positively with MRC score (ρ = 0.6, *p* = 0.013). For more information see section Correlation Analysis and Fig. S11 in Supplementary Appendix.

*Patients outcomes and SC biology after 180 days.* By day 180, of the 23 patients in the study, 13 had died after a median ICU stay of 15.0 [11.0–22.0] days, whereas 10 were alive after a median ICU stay of 12.5 [9.0–18.0] days. Seven survivors attended the follow-up visit with insulin clamp, and all of them consented to muscle biopsy (Fig. 1; one biopsy sample was excluded due to insufficient material). Among survivors, SF-36 physical score was 62.5 [55.0–75.0] and Baecke physical activity score was 7.56 [6.72–8.27]. In comparison, healthy controls had SF-36 physical score 97.5 [95.0–100.0] and Baecke score 8.14 [6.88–9.14]. Glucose disposal rates (M-values) were numerically higher compared with ICU day 7 (9.8 [5.5–11.6] mg/kg/min), but remained lower (∼71%) than in healthy controls. In a very limited subset of patients (*n* = 4–6 depending on the assay) with muscle biopsy available, SC number per fibre was 0.101 [0.095–0.108], corresponding to approximately 95% of control values, with a signal of impaired proliferation (0.183 [0.160–0.284], ∼50% of controls) and preserved myogenic differentiation (70.5 [68.3–72.5], ∼95% of controls). Metabolic characteristics of SC at day 180 are shown in Figs. 3 and 4.

## Discussion

In this study, we performed serial vastus lateralis biopsies in mechanically ventilated critically ill patients to assess biological characteristics of SC – progenitor mesenchymal stem cells with key role in muscle regeneration. Our cohort of critically ill patients developed early fast-twitch atrophy and severe insulin resistance of critical illness, with insulin-mediated glucose disposal dropping to one third of healthy controls. In these patients, our finding on satellite cell biology can be summarized as follows:

First, contrary to our hypothesis, satellite-cell depletion was not detectable early after ICU admission. SC number per fibre was similar in controls and ICU patients at day 0 and remained unchanged over the first week. In contrast, satellite-cell dysfunction was already detectable early, but it was subtle and qualitative rather than a simple loss of cells.

Second, the early functional defect was driven by impaired myogenic differentiation rather than decreased proliferation. Differentiation was transiently reduced at ICU day 0, losing the normal proliferation-fusion relationship and suggesting disruption of coordinated myogenic programming. Proliferation was not significantly lower overall at day 0 or 7, although it was heterogeneous and was lower in older patients associated with increased mitochondrial fission. Thus, the data support early dysregulation of myogenic programming, with clearer signals for impaired differentiation and context-dependent proliferation limits rather than global failure. The primary determinant of early proliferative capacity in ICU patients was age, while disease severity played a secondary role.

Third, contrary to our expectations, these defects were not accompanied by significant alterations of mitochondrial functions that were broadly preserved in ICU-derived SC. Instead, the strongest associations were with mitochondrial network organization and remodelling state: a more fragmented mitochondrial phenotype was linked to lower proliferation, lower respiratory performance, and worse muscle strength, whereas more interconnected morphology was linked to better respiratory capacity, ATP production, and proliferation. The absence of major bioenergetic failure in SC contrasts with prior reports of mitochondrial depletion and metabolic dysfunction in whole skeletal muscle and myotube models of critical illness [23–27], partially rescuable with lipid-substrates exposure [23, 28]. This discrepancy may reflect differences in cell type, disease phase, and analytical readout: unlike mature myofibres, SC are metabolically distinct progenitors, and in our ICU cohort they appeared to preserve core oxidative function despite severe systemic insulin resistance. Our data therefore suggest that the earliest satellite-cell lesion in critical illness may involve disturbed mitochondrial remodelling and impaired coupling of metabolism to differentiation, rather than overt bioenergetic failure.

Fourth, reliance on specific substrates was only modestly altered. At ICU Day 7-derived cells, the acute response to etomoxir and BPTES were significantly higher suggesting higher reliance on fatty acids and glutamin in basal state. However, maximal substrate-linked responses were largely preserved demonstrating their metabolic flexibility during increased energy demands. This suggests that the dominant abnormality is not a major switch in substrate dependence, but rather altered mitochondrial dynamics and disrupted regenerative programming. Notably, the lack of association between the reliance of SC OCR on glucose and the systemic insulin-induce glucose disposal (M-value) is suggestive that SC preserve metabolic characteristics of insulin independent progenitor cells.

Placed in the context of prior work, these findings refine the current model of muscle regeneration failure in critical illness. The early fast-twitch-predominant wasting in our cohort is consistent with earlier biopsy studies showing rapid muscle loss during the first week of critical illness, and with reports that ICU-acquired weakness is accompanied by molecular signals linked to impaired muscle homeostasis and repair, including GDF-15/microRNA dysregulation [1, 29]. However, our serial ICU-phase biopsies suggest that the earliest regenerative lesion is not satellite-cell depletion itself, but rather disturbed function despite preserved cell number; this contrasts with later survivor studies in which persistent weakness and sustained atrophy were associated with reduced satellite-cell content and abnormal muscle repair/remodelling programmes months after discharge [3, 4, 7]. Our data therefore support the view that regenerative failure in critical illness may evolve over time, with an early phase of preserved but dysfunctional SC preceding overt depletion in only some patients with incomplete recovery. This aligns with muscle stem-cell biology, where ageing impairs activation and proliferation without complete satellite-cell loss. Additionally, mitochondrial dynamics is increasingly recognized as a central regulator of SC proliferation, differentiation, mitophagy, and regeneration [30–32].

For clinical practice, the main implication is that ICU-acquired weakness may reflect not only muscle wasting, but also early regenerative dysfunction. Given the overall ageing of the ICU patient population, investigating the role of older age is highly relevant. In our cohort, older age was the clearest determinant of lower SC proliferative capacity. These results on muscle biology indicate that standard rehabilitation might have a limited impact in this specific subgroup. Consequently, this observation may help inform and optimize future rehabilitation frameworks; for instance, large multicentric studies are already underway (e.g., the ERUPT trial, NCT06960642) to determine whether earlier or adapted rehabilitation associates with better outcomes in older age groups. Next logical research step is to use banked SC cultures and determine in “ex vivo rescue” experiments, whether proliferation ability, coordination with myogenic differentiation and mitochondrial morphology abnormalities could be mitigated by targeted interventions. In addition, finding surrogate biomarkers of muscle regeneration capacity would be extremely helpful to personalise care, such as the dose and timing of physical rehabilitation or nutritional interventions.

The main strengths of this study are the serial biopsy design during early critical illness, the paired within-patient analyses, and the integrated assessment of satellite-cell number, function, mitochondrial morphology, bioenergetics, and signalling in relation to clinical phenotype. The main limitations are the younger age of control group and the small sample size, especially at day 180 which occurred due to higher-than-expected delayed mortality. In this context, the number of correlation analyses, although pre-specified, risks false positives and overinterpretations. On the same note, there is a risk of type II error, important relations could have been missed for the same reason. In addition, ex vivo satellite-cell assays may not fully reflect the in vivo niche.

In conclusion, early critical illness was associated with profound weakness, preferential loss of fast-twitch muscle fibres, and severe whole-body insulin resistance, but not with early depletion of the satellite-cell pool or overt bioenergetic failure in SC. Instead, the dominant early abnormality was a subtle but biologically meaningful disturbance of regenerative programming, characterized by transiently impaired differentiation, heterogeneous and age-sensitive proliferative capacity, and close links between satellite-cell function and mitochondrial network organization. These findings suggest that, in critical illness, impaired muscle recovery may arise not only from muscle wasting itself but, particularly in older patients, also from early dysfunction of muscle repair and regeneration. Future therapeutic targets may therefore include proteins regulating mitochondrial fusion and fission, mitochondrial morphology, and the coordination of satellite-cell myogenic differentiation.

## Supplementary Information

Below is the link to the electronic supplementary material.

Supplementary Appendix

Supplementary Material 2.

Supplementary Material 3.

Supplementary Material 4.

## Data Availability

Raw data are available with corresponding author upon reasonable request.
