# Supplementary material for: Critical illness impairs coordinated myogenesis: a prospective longitudinal study of skeletal muscle progenitor cell biology using serial muscle biopsies (SATELLITE study)
Source: Crit Care. 2026 Jun 16;30:443. doi: 10.1186/s13054-026-06131-5 (PMC13501688; doi:10.1186/s13054-026-06131-5)
Supplement: Supplementary file 1 [file 13054_2026_6131_MOESM3_ESM.pdf]

Mitofusin 1

GEL 1/ set A

	WB ID	Control/Patient ID	protein density	protein normalized to calibrator	GAPDH density	GAPDH normalized to calibrator	protein/GAPDH
		cal. mean	10981867,5		cal. mean	25207886	
	calibrator		11430450	1,04	26237938	1,04	1,00
**A1**	**H1/B**	**Control 17/B**	9591795	0,87	25525559	1,01	0,86
**A2**	**H1/T**	**Control 17/T**	d.n.r.	d.n.r.	d.n.r.	d.n.r.	d.n.r.
**A3**	**Sample X/B**	**Sample X/B**	excl.	#####	20146728	0,80	########
**A4**	**Sample X/T**	**Sample X/T**	excl.	#####	19557452	0,78	########
**A5**	**H3/B**	**Control 3/B**	9857655	0,90	20382535	0,81	1,11
**A6**	**H3/T**	**Control 3/T**	d.n.r.	d.n.r.	d.n.r.	d.n.r.	d.n.r.
**A7**	**CR4/B/D0**	**ICU 4/B/D0**	10237275	0,93	20062467	0,80	1,17
**A8**	**CR4/B/D7**	**ICU 4/B/D7**	8426700	0,77	19913007	0,79	0,97
**A9**	**CR4/T/D0**	**ICU 4/T/D0**	d.n.r.	d.n.r.	d.n.r.	d.n.r.	d.n.r.
**A10**	**CR4/T/D7**	**ICU 4/T/D7**	d.n.r.	d.n.r.	d.n.r.	d.n.r.	d.n.r.
**A11**	**CR8/B/D0**	**ICU 11/B/D0**	9640485	0,88	16150422	0,64	1,37
**A12**	**CR8/B/D7**	**ICU 11/B/D7**	10728945	0,98	17634541	0,70	1,40
**A13**	**CR8/T/D0**	**ICU 11/T/D0**	d.n.r.	d.n.r.	d.n.r.	d.n.r.	d.n.r.
**A14**	**CR8/T/D7**	**ICU 11/T/D7**	d.n.r.	d.n.r.	d.n.r.	d.n.r.	d.n.r.
**A15**	**CR10/B/D0**	**ICU 13/B/D0**	9848925	0,90	17586742	0,70	1,29
**A16**	**CR10/B/D7**	**ICU 13/B/D7**	9398565	0,86	18543098	0,74	1,16
**A17**	**CR10/T/D0**	**ICU 13/T/D0**	d.n.r.	d.n.r.	d.n.r.	d.n.r.	d.n.r.
**A18**	**CR10/T/D7**	**ICU 13/T/D7**	d.n.r.	d.n.r.	d.n.r.	d.n.r.	d.n.r.
**A19**	**CR11/B/D0**	**ICU 9/B/D0**	9021330	0,82	20294868	0,81	1,02
**A20**	**CR11/B/D7**	**ICU 9/B/D7**	10016415	0,91	21152021	0,84	1,09
**A21**	**CR11/T/D0**	**ICU 9/T/D0**	d.n.r.	d.n.r.	d.n.r.	d.n.r.	d.n.r.
**A22**	**CR11/T/D7**	**ICU 9/T/D7**	d.n.r.	d.n.r.	d.n.r.	d.n.r.	d.n.r.
	calibrator		10533285	0,96	24177834	0,96	1,00

Mitofusin 1

GEL 2/ set B

	WB ID	Control/Patient ID	protein density	protein normalized to calibrator	GAPDH density	GAPDH normalized to calibrator	protein/GAPDH
		cal. mean	10873196		cal. mean	28899095,5	
	calibrator		10859124	1,00	27854350	0,96	1,04
B1	H4/B	Control 12/B	7693488	0,71	28818080	1,00	0,71
B2	H4/T	Control 12/T	d.n.r.	d.n.r.	d.n.r.	d.n.r.	d.n.r.
B3	H5/B	Control 6/B	7412370	0,68	29889932	1,03	0,66
B4	H5/T	Control 6/T	d.n.r.	d.n.r.	d.n.r.	d.n.r.	d.n.r.
B5	H6/B	Control 8/B	6837078	0,63	23959672	0,83	0,76
B6	H6/T	Control 8/T	d.n.r.	d.n.r.	d.n.r.	d.n.r.	d.n.r.
B7	Sample Y/B	Sample Y/B	excl.	#####	29444064	1,02	########
B8	Sample Y/T	Sample Y/T	excl.	#####	27218925	0,94	########
B9	Sample Z/B	Sample Z/B	excl.	#####	25503469	0,88	########
B10	Sample Z/T	Sample Z/T	excl.	#####	22796640	0,79	########
B11	CR9/B/D0	ICU 3/B/D0	7719515	0,71	26684496	0,92	0,77
B12	CR9/B/D7	ICU 3/B/D7	6568250	0,60	27844674	0,96	0,63
B13	CR9/T/D0	ICU 3/T/D0	d.n.r.	d.n.r.	d.n.r.	d.n.r.	d.n.r.
B14	CR9/T/D7	ICU 3/T/D7	d.n.r.	d.n.r.	d.n.r.	d.n.r.	d.n.r.
B15	CR12/B/D0	ICU 20/B/D0	4411132	0,41	29392896	1,02	0,40
B16	CR12/B/D7	ICU 20/B/D7	6324978	0,58	29492584	1,02	0,57
B17	CR12/T/D0	ICU 20/T/D0	d.n.r.	d.n.r.	d.n.r.	d.n.r.	d.n.r.
B18	CR12/T/D7	ICU 20/T/D7	d.n.r.	d.n.r.	d.n.r.	d.n.r.	d.n.r.
B19	CR13/B/D0	ICU 22/B/D0	8398324	0,77	28194210	0,98	0,79
B20	CR13/B/D7	ICU 22/B/D7	9962825	0,92	25151087	0,87	1,05
B21	CR13/T/D0	ICU 22/T/D0	d.n.r.	d.n.r.	d.n.r.	d.n.r.	d.n.r.
B22	CR13/T/D7	ICU 22/T/D7	d.n.r.	d.n.r.	d.n.r.	d.n.r.	d.n.r.
	calibrator		10887268	1,00	29943841	1,04	0,97

Mitofusin 1

	WB ID	Control/Patient ID	protein density	protein normalized to calibrator	GAPDH density	GAPDH normalized to calibrator	protein/GAPDH
**GEL 3/ set C**		cal. mean	10128671		cal. mean	34079936	
	calibrator		10666480	1,05	34138962	1,00	1,05
**C1**	**H15/B**	**Control 2/B**	7824968	0,77	29730870	0,87	0,89
**C2**	**H15/T**	**Control 2/T**	d.n.r.	d.n.r.	d.n.r.	d.n.r.	d.n.r.
**C3**	**H16/B**	**Control 14/B**	8223374	0,81	30098518	0,88	0,92
**C4**	**H16/T**	**Control 14/T**	d.n.r.	d.n.r.	d.n.r.	d.n.r.	d.n.r.
**C5**	**H17/B**	**Control 10/B**	9694316	0,96	20994664	0,62	1,55
**C6**	**H17/T**	**Control 10/T**	d.n.r.	d.n.r.	d.n.r.	d.n.r.	d.n.r.
**C7**	**CR3/B/D0**	**ICU 5/B/D0**	3264620	0,32	22660589	0,66	0,48
**C8**	**CR3/B/D7**	**ICU 5/B/D7**	3680046	0,36	18025875	0,53	0,69
**C9**	**CR3/T/D0**	**ICU 5/T/D0**	d.n.r.	d.n.r.	d.n.r.	d.n.r.	d.n.r.
**C10**	**CR3/T/D7**	**ICU 5/T/D7**	d.n.r.	d.n.r.	d.n.r.	d.n.r.	d.n.r.
**C11**	**CR5/B/D0**	**ICU 8/B/D0**	2987240	0,29	19994035	0,59	0,50
**C12**	**CR5/B/D7**	**ICU 8/B/D7**	2749512	0,27	17391984	0,51	0,53
**C13**	**CR5/T/D0**	**ICU 8/T/D0**	d.n.r.	d.n.r.	d.n.r.	d.n.r.	d.n.r.
**C14**	**CR5/T/D7**	**ICU 8/T/D7**	d.n.r.	d.n.r.	d.n.r.	d.n.r.	d.n.r.
**C15**	**CR7/B/D0**	**ICU 6/B/D0**	5866150	0,58	22085112	0,65	0,89
**C16**	**CR7/B/D7**	**ICU 6/B/D7**	6670874	0,66	18388750	0,54	1,22
**C17**	**CD7/T/D0**	**ICU 6/T/D0**	d.n.r.	d.n.r.	d.n.r.	d.n.r.	d.n.r.
**C18**	**CR7/T/D7**	**ICU 6/T/D7**	d.n.r.	d.n.r.	d.n.r.	d.n.r.	d.n.r.
**C19**	**CR20/B/D0**	**ICU 7/B/D0**	6890156	0,68	24852613	0,73	0,93
**C20**	**CR20/B/D7**	**ICU 7/B/D7**	8036200	0,79	25778115	0,76	1,05
**C21**	**CR20/T/D0**	**ICU 7/T/D0**	d.n.r.	d.n.r.	d.n.r.	d.n.r.	d.n.r.
**C22**	**CR20/T/D7**	**ICU 7/T/D7**	d.n.r.	d.n.r.	d.n.r.	d.n.r.	d.n.r.
	calibrator		9590862	0,95	34020910	1,00	0,95

Mitofusin 1

	WB ID	Control/Patient ID	protein density	protein normalized to calibrator	GAPDH density	GAPDH normalized to calibrator	protein/GAPDH
GEL 4/ set D		cal. mean	9740400		cal. mean	35190284	
	calibrator		10686850	1,10	37167787	1,06	1,04
**D1**	**H2/B**	**Control 4/B**	10420656	1,07	30949908	0,88	1,22
**D2**	**H2/T**	**Control 4/T**	d.n.r.	d.n.r.	d.n.r.	d.n.r.	d.n.r.
**D3**	**H7/B**	**Control 13/B**	8056348	0,83	31992571	0,91	0,91
**D4**	**H7/T**	**Control 13/T**	d.n.r.	d.n.r.	d.n.r.	d.n.r.	d.n.r.
**D5**	**H8/B**	**Control 18/B**	12798994	1,31	24406066	0,69	1,89
**D6**	**H8/T**	**Control 18/T**	d.n.r.	d.n.r.	d.n.r.	d.n.r.	d.n.r.
**D7**	**H11/B**	**Control 19/B**	7979298	0,82	22372376	0,64	1,29
**D8**	**H11/T**	**Control 19/T**	d.n.r.	d.n.r.	d.n.r.	d.n.r.	d.n.r.
**D9**	**H19/B**	**Control 5/B**	10037660	1,03	18229138	0,52	1,99
**D10**	**H19/T**	**Control 5/T**	d.n.r.	d.n.r.	d.n.r.	d.n.r.	d.n.r.
**D11**	**CR2/B/D0**	**ICU 14/B/D0**	8955464	0,92	17679473	0,50	1,83
**D12**	**CR2/B/D7**	**ICU 14/B/D7**	8405764	0,86	18461459	0,52	1,64
**D13**	**CR2/B/D180**	**ICU 14/B/D180**	8926760	0,92	19948365	0,57	1,62
**D14**	**CR2/T/D0**	**ICU 14/T/D0**	d.n.r.	d.n.r.	d.n.r.	d.n.r.	d.n.r.
**D15**	**CR2/T/D7**	**ICU 14/T/D7**	d.n.r.	d.n.r.	d.n.r.	d.n.r.	d.n.r.
**D16**	**CR2/T/D180**	**ICU 14/T/D180**	d.n.r.	d.n.r.	d.n.r.	d.n.r.	d.n.r.
**D17**	**CR17/B/D0**	**ICU 23/B/D0**	6749442	0,69	22814050	0,65	1,07
**D18**	**CR17/B/D7**	**ICU 23/B/D7**	4939020	0,51	24909295	0,71	0,72
**D19**	**CR17/B/D180**	**ICU 23/B/D180**	6052726	0,62	26712850	0,76	0,82
**D20**	**CR17/T/D0**	**ICU 23/T/D0**	d.n.r.	d.n.r.	d.n.r.	d.n.r.	d.n.r.
**D21**	**CR17/T/D7**	**ICU 23/T/D7**	d.n.r.	d.n.r.	d.n.r.	d.n.r.	d.n.r.
**D22**	**CR17/T/D180**	**ICU 23/T/D180**	d.n.r.	d.n.r.	d.n.r.	d.n.r.	d.n.r.
	calibrator		8793950	0,90	33212781	0,94	0,96

Mitofusin 1

	WB ID	Control/Patient ID	protein density	protein normalized to calibrator	GAPDH density	GAPDH normalized to calibrator	protein/GAPDH
GEL 5/ set E		cal. mean	14986089		cal. mean	13612646,5	
	calibrator		15077844	1,01	13056250	0,96	1,05
E1	H12/B	Control 7/B	11489400	0,77	15730101	1,16	0,66
E2	H12/T	Control 7/T	d.n.r.	d.n.r.	d.n.r.	d.n.r.	d.n.r.
E3	H13/B	Control 1/B	5244390	0,35	17836110	1,31	0,27
E4	H13/T	Control 1/T	d.n.r.	d.n.r.	d.n.r.	d.n.r.	d.n.r.
E5	H18/B	Control 11/B	10341720	0,69	17137798	1,26	0,55
E6	H18/T	Control 11/T	d.n.r.	d.n.r.	d.n.r.	d.n.r.	d.n.r.
E7	CR6/B/D0	ICU 21/B/D0	15340140	1,02	16121115	1,18	0,86
E8	CR6/B/D7	ICU 21/B/D7	12693420	0,85	12428020	0,91	0,93
E9	CR6/T/D0	ICU 21/T/D0	d.n.r.	d.n.r.	d.n.r.	d.n.r.	d.n.r.
E10	CR6/T/D7	ICU 21/T/D7	d.n.r.	d.n.r.	d.n.r.	d.n.r.	d.n.r.
E11	CR14/B/D0	ICU 10/B/D0	13375755	0,89	12644640	0,93	0,96
E12	CR14/B/D7	ICU 10/B/D7	12600135	0,84	13423200	0,99	0,85
E13	CR14/T/D0	ICU 10/T/D0	d.n.r.	d.n.r.	d.n.r.	d.n.r.	d.n.r.
E14	CR14/T/D7	ICU 10/T/D7	d.n.r.	d.n.r.	d.n.r.	d.n.r.	d.n.r.
E15	Sample W/B/D0	Sample W/B/D0	excl.	#####	13286318	0,98	########
E16	Sample W/B/D7	Sample W/B/D7	excl.	#####	14503083	1,07	########
E17	Sample W/T/D0	Sample W/T/D0	excl.	#####	15358237	1,13	########
E18	Sample W/T/D7	Sample W/T/D7	excl.	#####	14918082	1,10	########
E19	CR19/B/D0	ICU 15/B/D0	11212740	0,75	18376065	1,35	0,55
E20	CR19/B/D7	ICU 15/B/D7	9039825	0,60	18750060	1,38	0,44
E21	CR19/T/D0	ICU 15/T/D0	d.n.r.	d.n.r.	d.n.r.	d.n.r.	d.n.r.
E22	CR19/T/D7	ICU 15/T/D7	d.n.r.	d.n.r.	d.n.r.	d.n.r.	d.n.r.
	calibrator		14894334	0,99	14169043	1,04	0,95

Mitofusin 1

	WB ID	Control/Patient ID	protein density	protein normalized to calibrator	GAPDH density	GAPDH normalized to calibrator	protein/GAPDH
GEL 6/ set F		cal. mean	17262949		cal. mean 24950732		
	calibrator		15682642	0,91	25509639	1,02	0,89
F1	H9/B	Control 9/B	13160462	0,76	32156010	1,29	0,59
F2	H9/T	Control 9/T	d.n.r.	d.n.r.	d.n.r.	d.n.r.	d.n.r.
F3	H10/B	Control 15/B	12082498	0,70	31970444	1,28	0,55
F4	H10/T	Control 15/T	d.n.r.	d.n.r.	d.n.r.	d.n.r.	d.n.r.
F5	H14/B	Control 20/B	13886894	0,80	25759862	1,03	0,78
F6	H14/T	Control 20/T	d.n.r.	d.n.r.	d.n.r.	d.n.r.	d.n.r.
F7	CR1/B/D0	ICU 1/B/D0	12621756	0,73	24703341	0,99	0,74
F8	CR1/B/D7	ICU 1/B/D7	16108234	0,93	22559436	0,90	1,03
F9	CR1/B/D180	ICU 1/B/D180	11964094	0,69	26013983	1,04	0,66
F10	CR1/T/D0	ICU 1/T/D0	d.n.r.	d.n.r.	d.n.r.	d.n.r.	d.n.r.
F11	CR1/T/D7	ICU 1/T/D7	d.n.r.	d.n.r.	d.n.r.	d.n.r.	d.n.r.
F12	CR1/T/D180	ICU 1/T/D180	d.n.r.	d.n.r.	d.n.r.	d.n.r.	d.n.r.
F13	CR18/B/D0	ICU 18/B/D0	12546040	0,73	24749871	0,99	0,73
F14	CR18/B/D7	ICU 18/B/D7	18629402	1,08	21148778	0,85	1,27
F15	CR18/B/D180	ICU 18/B/D180	15140946	0,88	21108311	0,85	1,04
F16	CR18/T/D0	ICU 18/T/D0	d.n.r.	d.n.r.	d.n.r.	d.n.r.	d.n.r.
F17	CR18/T/D7	ICU 18/T/D7	d.n.r.	d.n.r.	d.n.r.	d.n.r.	d.n.r.
F18	CR18/T/D180	ICU 18/T/D180	d.n.r.	d.n.r.	d.n.r.	d.n.r.	d.n.r.
F19	CR15/B/D0	ICU 12/B/D0	7285066	0,42	27652779	1,11	0,38
F20	CR15/B/D7	ICU 12/B/D7	8542752	0,49	28943200	1,16	0,43
F21	CR15/T/D0	ICU 12/T/D0	d.n.r.	d.n.r.	d.n.r.	d.n.r.	d.n.r.
F22	CR15/T/D7	ICU 12/T/D7	d.n.r.	d.n.r.	d.n.r.	d.n.r.	d.n.r.
	calibrator		18843256	1,09	24391825	0,98	1,12

Mitofusin 1

	WB ID	Control/Patient ID	protein density	protein normalized to calibrator	GAPDH density	GAPDH normalized to calibrator	protein/GAPDH
**GEL 7/ set ICU 16**		**cal. mean**	16750874,5	**cal. mean**	40515874		
	calibrator		17202799	1,03	43007588	1,06	
**E15**	**CR16/B/D0**	**ICU 16/B/D0**	15823035	0,94	37231612	0,92	1,03
**E16**	**CR16/B/D7**	**ICU 16/B/D7**	15114956	0,90	33408164	0,82	1,09
**E17**	**CR16/T/D0**	**ICU 16/T/D0**	d.n.r.	d.n.r.	d.n.r.	d.n.r.	d.n.r.
**E18**	**CR16/T/D7**	**ICU 16/T/D7**	d.n.r.	d.n.r.	d.n.r.	d.n.r.	d.n.r.
	calibrator		16298950	0,97	38024160	0,94	

Mitofusin 2

	WB ID	Control/Patient ID	protein density	protein normalized to calibrator	GAPDH density	GAPDH normalized to calibrator	protein/GAPDH
GEL 1/ set A		cal. mean	11020027,5		cal. mean 26252326		
	calibrator		11437110	1,04	27774192	1,06	0,98
A1	H1/B	Control 17/B	11963250	1,09	26549130	1,01	1,07
A2	H1/T	Control 17/T	d.n.r.	d.n.r.	d.n.r.	d.n.r.	d.n.r.
A3	Sample X/B	Sample X/B	excl.	#####	23658300	0,90	########
A4	Sample X/T	Sample X/T	excl.	#####	20212632	0,77	########
A5	H3/B	Control 3/B	7956990	0,72	22203368	0,85	0,85
A6	H3/T	Control 3/T	d.n.r.	d.n.r.	d.n.r.	d.n.r.	d.n.r.
A7	CR4/B/D0	ICU 4/B/D0	8956350	0,81	22318120	0,85	0,96
A8	CR4/B/D7	ICU 4/B/D7	5867100	0,53	21786380	0,83	0,64
A9	CR4/T/D0	ICU 4/T/D0	d.n.r.	d.n.r.	d.n.r.	d.n.r.	d.n.r.
A10	CR4/T/D7	ICU 4/T/D7	d.n.r.	d.n.r.	d.n.r.	d.n.r.	d.n.r.
A11	CR8/B/D0	ICU 11/B/D0	7966530	0,72	24650043	0,94	0,77
A12	CR8/B/D7	ICU 11/B/D7	8601975	0,78	26404414	1,01	0,78
A13	CR8/T/D0	ICU 11/T/D0	d.n.r.	d.n.r.	d.n.r.	d.n.r.	d.n.r.
A14	CR8/T/D7	ICU 11/T/D7	d.n.r.	d.n.r.	d.n.r.	d.n.r.	d.n.r.
A15	CR10/B/D0	ICU 13/B/D0	6799140	0,62	21712328	0,83	0,75
A16	CR10/B/D7	ICU 13/B/D7	7603155	0,69	23581122	0,90	0,77
A17	CR10/T/D0	ICU 13/T/D0	d.n.r.	d.n.r.	d.n.r.	d.n.r.	d.n.r.
A18	CR10/T/D7	ICU 13/T/D7	d.n.r.	d.n.r.	d.n.r.	d.n.r.	d.n.r.
A19	CR11/B/D0	ICU 9/B/D0	6459210	0,59	23880825	0,91	0,64
A20	CR11/B/D7	ICU 9/B/D7	6695145	0,61	21025925	0,80	0,76
A21	CR11/T/D0	ICU 9/T/D0	d.n.r.	d.n.r.	d.n.r.	d.n.r.	d.n.r.
A22	CR11/T/D7	ICU 9/T/D7	d.n.r.	d.n.r.	d.n.r.	d.n.r.	d.n.r.
	calibrator		10602945	0,96	24730460	0,94	1,02

Mitofusin 2

	WB ID	Control/Patient ID	protein density	protein normalized to calibrator	GAPDH density	GAPDH normalized to calibrator	protein/GAPDH
GEL 2/ set B		cal. mean	13475857,5		cal. mean	28899095,5	
	calibrator		13024575	0,97	27854350	0,96	1,00
**B1**	**H4/B**	**Control 12/B**	12407265	0,92	28818080	1,00	0,92
**B2**	**H4/T**	**Control 12/T**	d.n.r.	d.n.r.	d.n.r.	d.n.r.	d.n.r.
**B3**	**H5/B**	**Control 6/B**	10045125	0,75	29889932	1,03	0,72
**B4**	**H5/T**	**Control 6/T**	d.n.r.	d.n.r.	d.n.r.	d.n.r.	d.n.r.
**B5**	**H6/B**	**Control 8/B**	10484460	0,78	23959672	0,83	0,94
**B6**	**H6/T**	**Control 8/T**	d.n.r.	d.n.r.	d.n.r.	d.n.r.	d.n.r.
**B7**	**Sample Y/B**	**Sample Y/B**	excl.	#####	29444064	1,02	########
**B8**	**Sample Y/T**	**Sample Y/T**	excl.	#####	27218925	0,94	########
**B9**	**Sample Z/B**	**Sample Z/B**	excl.	#####	25503469	0,88	########
**B10**	**Sample Z/T**	**Sample Z/T**	excl.	#####	22796640	0,79	########
**B11**	**CR9/B/D0**	**ICU 3/B/D0**	10143765	0,75	26684496	0,92	0,82
**B12**	**CR9/B/D7**	**ICU 3/B/D7**	10041615	0,75	27844674	0,96	0,77
**B13**	**CR9/T/D0**	**ICU 3/T/D0**	d.n.r.	d.n.r.	d.n.r.	d.n.r.	d.n.r.
**B14**	**CR9/T/D7**	**ICU 3/T/D7**	d.n.r.	d.n.r.	d.n.r.	d.n.r.	d.n.r.
**B15**	**CR12/B/D0**	**ICU 20/B/D0**	8912655	0,66	29392896	1,02	0,65
**B16**	**CR12/B/D7**	**ICU 20/B/D7**	11824650	0,88	29492584	1,02	0,86
**B17**	**CR12/T/D0**	**ICU 20/T/D0**	d.n.r.	d.n.r.	d.n.r.	d.n.r.	d.n.r.
**B18**	**CR12/T/D7**	**ICU 20/T/D7**	d.n.r.	d.n.r.	d.n.r.	d.n.r.	d.n.r.
**B19**	**CR13/B/D0**	**ICU 22/B/D0**	11858940	0,88	28194210	0,98	0,90
**B20**	**CR13/B/D7**	**ICU 22/B/D7**	13396950	0,99	25151087	0,87	1,14
**B21**	**CR13/T/D0**	**ICU 22/T/D0**	d.n.r.	d.n.r.	d.n.r.	d.n.r.	d.n.r.
**B22**	**CR13/T/D7**	**ICU 22/T/D7**	d.n.r.	d.n.r.	d.n.r.	d.n.r.	d.n.r.
	calibrator		13927140	1,03	29943841	1,04	1,00

Mitofusin 2

	WB ID	Control/Patient ID	protein density	protein normalized to calibrator	GAPDH density	GAPDH normalized to calibrator	protein/GAPDH
**GEL 3/ set C**		**cal. mean**	16597362,5		**cal. mean** 22029750		
	calibrator		15856359	0,96	20662800	0,94	1,02
**C1**	**H15/B**	**Control 2/B**	12694942	0,76	20219460	0,92	0,83
**C2**	**H15/T**	**Control 2/T**	d.n.r.	d.n.r.	d.n.r.	d.n.r.	d.n.r.
**C3**	**H16/B**	**Control 14/B**	14974200	0,90	15286365	0,69	1,30
**C4**	**H16/T**	**Control 14/T**	d.n.r.	d.n.r.	d.n.r.	d.n.r.	d.n.r.
**C5**	**H17/B**	**Control 10/B**	15073822	0,91	14141864	0,64	1,41
**C6**	**H17/T**	**Control 10/T**	d.n.r.	d.n.r.	d.n.r.	d.n.r.	d.n.r.
**C7**	**CR3/B/D0**	**ICU 5/B/D0**	7036942	0,42	14270928	0,65	0,65
**C8**	**CR3/B/D7**	**ICU 5/B/D7**	9544494	0,58	13239864	0,60	0,96
**C9**	**CR3/T/D0**	**ICU 5/T/D0**	d.n.r.	d.n.r.	d.n.r.	d.n.r.	d.n.r.
**C10**	**CR3/T/D7**	**ICU 5/T/D7**	d.n.r.	d.n.r.	d.n.r.	d.n.r.	d.n.r.
**C11**	**CR5/B/D0**	**ICU 8/B/D0**	11263974	0,68	18034032	0,82	0,83
**C12**	**CR5/B/D7**	**ICU 8/B/D7**	10391906	0,63	15358512	0,70	0,90
**C13**	**CR5/T/D0**	**ICU 8/T/D0**	d.n.r.	d.n.r.	d.n.r.	d.n.r.	d.n.r.
**C14**	**CR5/T/D7**	**ICU 8/T/D7**	d.n.r.	d.n.r.	d.n.r.	d.n.r.	d.n.r.
**C15**	**CR7/B/D0**	**ICU 6/B/D0**	12990814	0,78	17619684	0,80	0,98
**C16**	**CR7/B/D7**	**ICU 6/B/D7**	11267608	0,68	17926524	0,81	0,83
**C17**	**CD7/T/D0**	**ICU 6/T/D0**	d.n.r.	d.n.r.	d.n.r.	d.n.r.	d.n.r.
**C18**	**CR7/T/D7**	**ICU 6/T/D7**	d.n.r.	d.n.r.	d.n.r.	d.n.r.	d.n.r.
**C19**	**CR20/B/D0**	**ICU 7/B/D0**	11900465	0,72	14741100	0,67	1,07
**C20**	**CR20/B/D7**	**ICU 7/B/D7**	13601418	0,82	15625234	0,71	1,16
**C21**	**CR20/T/D0**	**ICU 7/T/D0**	d.n.r.	d.n.r.	d.n.r.	d.n.r.	d.n.r.
**C22**	**CR20/T/D7**	**ICU 7/T/D7**	d.n.r.	d.n.r.	d.n.r.	d.n.r.	d.n.r.
	calibrator		17338366	1,04	23396700	1,06	0,98

Mitofusin 2

	WB ID	Control/Patient ID	protein density	protein normalized to calibrator		GAPDH density	GAPDH normalized to calibrator	protein/GAPDH
GEL 4/ set D		cal. mean	17590980		cal. mean	27123350		
	calibrator		16498328	0,94		21501050	0,79	1,18
D1	H2/B	Control 4/B	13668512	0,78		15463789	0,57	1,36
D2	H2/T	Control 4/T	d.n.r.	d.n.r.		d.n.r.	d.n.r.	d.n.r.
D3	H7/B	Control 13/B	15977016	0,91		15370470	0,57	1,60
D4	H7/T	Control 13/T	d.n.r.	d.n.r.		d.n.r.	d.n.r.	d.n.r.
D5	H8/B	Control 18/B	16747500	0,95		13315368	0,49	1,94
D6	H8/T	Control 18/T	d.n.r.	d.n.r.		d.n.r.	d.n.r.	d.n.r.
D7	H11/B	Control 19/B	16599484	0,94		15176872	0,56	1,69
D8	H11/T	Control 19/T	d.n.r.	d.n.r.		d.n.r.	d.n.r.	d.n.r.
D9	H19/B	Control 5/B	12953996	0,74		12146022	0,45	1,64
D10	H19/T	Control 5/T	d.n.r.	d.n.r.		d.n.r.	d.n.r.	d.n.r.
D11	CR2/B/D0	ICU 14/B/D0	16019388	0,91		13352832	0,49	1,85
D12	CR2/B/D7	ICU 14/B/D7	15501552	0,88		14137728	0,52	1,69
D13	CR2/B/D180	ICU 14/B/D180	13943776	0,79		11800978	0,44	1,82
D14	CR2/T/D0	ICU 14/T/D0	d.n.r.	d.n.r.		d.n.r.	d.n.r.	d.n.r.
D15	CR2/T/D7	ICU 14/T/D7	d.n.r.	d.n.r.		d.n.r.	d.n.r.	d.n.r.
D16	CR2/T/D180	ICU 14/T/D180	d.n.r.	d.n.r.		d.n.r.	d.n.r.	d.n.r.
D17	CR17/B/D0	ICU 23/B/D0	16267196	0,92		13229508	0,49	1,90
D18	CR17/B/D7	ICU 23/B/D7	12926364	0,73		11261786	0,42	1,77
D19	CR17/B/D180	ICU 23/B/D180	17630096	1,00		10989998	0,41	2,47
D20	CR17/T/D0	ICU 23/T/D0	d.n.r.	d.n.r.		d.n.r.	d.n.r.	d.n.r.
D21	CR17/T/D7	ICU 23/T/D7	d.n.r.	d.n.r.		d.n.r.	d.n.r.	d.n.r.
D22	CR17/T/D180	ICU 23/T/D180	d.n.r.	d.n.r.		d.n.r.	d.n.r.	d.n.r.
	calibrator		18683632	1,06		32745650	1,21	0,88

Mitofusin 2

	WB ID	Control/Patient ID	protein density	protein normalized to calibrator	GAPDH density	GAPDH normalized to calibrator	protein/GAPDH
GEL 5/ set E		cal. mean	17913462,5	cal. mean	13612646,5		
	calibrator		18631270	1,04	13056250	0,96	1,08
**E1**	**H12/B**	**Control 7/B**	17375400	0,97	15730101	1,16	0,84
**E2**	**H12/T**	**Control 7/T**	d.n.r.	d.n.r.	d.n.r.	d.n.r.	d.n.r.
**E3**	**H13/B**	**Control 1/B**	11407135	0,64	17836110	1,31	0,49
**E4**	**H13/T**	**Control 1/T**	d.n.r.	d.n.r.	d.n.r.	d.n.r.	d.n.r.
**E5**	**H18/B**	**Control 11/B**	13643160	0,76	17137798	1,26	0,60
**E6**	**H18/T**	**Control 11/T**	d.n.r.	d.n.r.	d.n.r.	d.n.r.	d.n.r.
**E7**	**CR6/B/D0**	**ICU 21/B/D0**	15734331	0,88	16121115	1,18	0,74
**E8**	**CR6/B/D7**	**ICU 21/B/D7**	11669677	0,65	12428020	0,91	0,71
**E9**	**CR6/T/D0**	**ICU 21/T/D0**	d.n.r.	d.n.r.	d.n.r.	d.n.r.	d.n.r.
**E10**	**CR6/T/D7**	**ICU 21/T/D7**	d.n.r.	d.n.r.	d.n.r.	d.n.r.	d.n.r.
**E11**	**CR14/B/D0**	**ICU 10/B/D0**	15051280	0,84	12644640	0,93	0,90
**E12**	**CR14/B/D7**	**ICU 10/B/D7**	14287248	0,80	13423200	0,99	0,81
**E13**	**CR14/T/D0**	**ICU 10/T/D0**	d.n.r.	d.n.r.	d.n.r.	d.n.r.	d.n.r.
**E14**	**CR14/T/D7**	**ICU 10/T/D7**	d.n.r.	d.n.r.	d.n.r.	d.n.r.	d.n.r.
**E15**	**Sample W/B/D0**	**Sample W/B/D0**	excl.	#####	13286318	0,98	########
**E16**	**Sample W/B/D7**	**Sample W/B/D7**	excl.	#####	14503083	1,07	########
**E17**	**Sample W/T/D0**	**Sample W/T/D0**	excl.	#####	15358237	1,13	########
**E18**	**Sample W/T/D7**	**Sample W/T/D7**	excl.	#####	14918082	1,10	########
**E19**	**CR19/B/D0**	**ICU 15/B/D0**	15642023	0,87	18376065	1,35	0,65
**E20**	**CR19/B/D7**	**ICU 15/B/D7**	15919605	0,89	18750060	1,38	0,65
**E21**	**CR19/T/D0**	**ICU 15/T/D0**	d.n.r.	d.n.r.	d.n.r.	d.n.r.	d.n.r.
**E22**	**CR19/T/D7**	**ICU 15/T/D7**	d.n.r.	d.n.r.	d.n.r.	d.n.r.	d.n.r.
	calibrator		17195655	0,96	14169043	1,04	0,92

Mitofusin 2

	WB ID	Control/Patient ID	protein density	protein normalized to calibrator	GAPDH density	GAPDH normalized to calibrator	protein/GAPDH
GEL 6/ set F		cal. mean	18120435		cal. mean	24950732	
	calibrator		16884392	0,93	25509639	1,02	0,91
F1	H9/B	Control 9/B	16249224	0,90	32156010	1,29	0,70
F2	H9/T	Control 9/T	d.n.r.	d.n.r.	d.n.r.	d.n.r.	d.n.r.
F3	H10/B	Control 15/B	19760136	1,09	31970444	1,28	0,85
F4	H10/T	Control 15/T	d.n.r.	d.n.r.	d.n.r.	d.n.r.	d.n.r.
F5	H14/B	Control 20/B	18756638	1,04	25759862	1,03	1,00
F6	H14/T	Control 20/T	d.n.r.	d.n.r.	d.n.r.	d.n.r.	d.n.r.
F7	CR1/B/D0	ICU 1/B/D0	12389640	0,68	24703341	0,99	0,69
F8	CR1/B/D7	ICU 1/B/D7	18136328	1,00	22559436	0,90	1,11
F9	CR1/B/D180	ICU 1/B/D180	12857598	0,71	26013983	1,04	0,68
F10	CR1/T/D0	ICU 1/T/D0	d.n.r.	d.n.r.	d.n.r.	d.n.r.	d.n.r.
F11	CR1/T/D7	ICU 1/T/D7	d.n.r.	d.n.r.	d.n.r.	d.n.r.	d.n.r.
F12	CR1/T/D180	ICU 1/T/D180	d.n.r.	d.n.r.	d.n.r.	d.n.r.	d.n.r.
F13	CR18/B/D0	ICU 18/B/D0	14615074	0,81	24749871	0,99	0,81
F14	CR18/B/D7	ICU 18/B/D7	16344076	0,90	21148778	0,85	1,06
F15	CR18/B/D180	ICU 18/B/D180	18237068	1,01	21108311	0,85	1,19
F16	CR18/T/D0	ICU 18/T/D0	d.n.r.	d.n.r.	d.n.r.	d.n.r.	d.n.r.
F17	CR18/T/D7	ICU 18/T/D7	d.n.r.	d.n.r.	d.n.r.	d.n.r.	d.n.r.
F18	CR18/T/D180	ICU 18/T/D180	d.n.r.	d.n.r.	d.n.r.	d.n.r.	d.n.r.
F19	CR15/B/D0	ICU 12/B/D0	17996810	0,99	27652779	1,11	0,90
F20	CR15/B/D7	ICU 12/B/D7	12544606	0,69	28943200	1,16	0,60
F21	CR15/T/D0	ICU 12/T/D0	d.n.r.	d.n.r.	d.n.r.	d.n.r.	d.n.r.
F22	CR15/T/D7	ICU 12/T/D7	d.n.r.	d.n.r.	d.n.r.	d.n.r.	d.n.r.
	calibrator		19356478	1,07	24391825	0,98	1,09

Mitofusin 2

	WB ID	Control/Patient ID	protein density	protein normalized to calibrator	GAPDH density	GAPDH normalized to calibrator	protein/GAPDH
**GEL 7/ set ICU 16**		**cal. mean**	11266359		**cal. mean**	40515874	
	calibrator		10278270	0,91	43007588	1,06	
**E15**	**CR16/B/D0**	**ICU 16/B/D0**	14640816	1,30	37231612	0,92	1,41
**E16**	**CR16/B/D7**	**ICU 16/B/D7**	12435401	1,10	33408164	0,82	1,34
**E17**	**CR16/T/D0**	**ICU 16/T/D0**	d.n.r.	d.n.r.	d.n.r.	d.n.r.	d.n.r.
**E18**	**CR16/T/D7**	**ICU 16/T/D7**	d.n.r.	d.n.r.	d.n.r.	d.n.r.	d.n.r.
	calibrator		12254448	1,09	38024160	0,94	

Tom20

	WB ID	Control/Patient ID	protein density	protein normalized to calibrator	GAPDH density	GAPDH normalized to calibrator	protein/GAPDH
GEL 1/ set A		cal. mean	4425041,5		cal. mean	25207886	
	calibrator		4451682	1,01	26237938	1,04	0,97
**A1**	**H1/B**	**Control 17/B**	17192040	3,89	25525559	1,01	3,84
**A2**	**H1/T**	**Control 17/T**	d.n.r.	d.n.r.	d.n.r.	d.n.r.	d.n.r.
**A3**	**Sample X/B**	**Sample X/B**	excl.	#####	20146728	0,80	########
**A4**	**Sample X/T**	**Sample X/T**	excl.	#####	19557452	0,78	########
**A5**	**H3/B**	**Control 3/B**	6596168	1,49	20382535	0,81	1,84
**A6**	**H3/T**	**Control 3/T**	d.n.r.	d.n.r.	d.n.r.	d.n.r.	d.n.r.
**A7**	**CR4/B/D0**	**ICU 4/B/D0**	11943288	2,70	20062467	0,80	3,39
**A8**	**CR4/B/D7**	**ICU 4/B/D7**	7102452	1,61	19913007	0,79	2,03
**A9**	**CR4/T/D0**	**ICU 4/T/D0**	d.n.r.	d.n.r.	d.n.r.	d.n.r.	d.n.r.
**A10**	**CR4/T/D7**	**ICU 4/T/D7**	d.n.r.	d.n.r.	d.n.r.	d.n.r.	d.n.r.
**A11**	**CR8/B/D0**	**ICU 11/B/D0**	4212281	0,95	16150422	0,64	1,49
**A12**	**CR8/B/D7**	**ICU 11/B/D7**	8162020	1,84	17634541	0,70	2,64
**A13**	**CR8/T/D0**	**ICU 11/T/D0**	d.n.r.	d.n.r.	d.n.r.	d.n.r.	d.n.r.
**A14**	**CR8/T/D7**	**ICU 11/T/D7**	d.n.r.	d.n.r.	d.n.r.	d.n.r.	d.n.r.
**A15**	**CR10/B/D0**	**ICU 13/B/D0**	9570422	2,16	17586742	0,70	3,10
**A16**	**CR10/B/D7**	**ICU 13/B/D7**	9561210	2,16	18543098	0,74	2,94
**A17**	**CR10/T/D0**	**ICU 13/T/D0**	d.n.r.	d.n.r.	d.n.r.	d.n.r.	d.n.r.
**A18**	**CR10/T/D7**	**ICU 13/T/D7**	d.n.r.	d.n.r.	d.n.r.	d.n.r.	d.n.r.
**A19**	**CR11/B/D0**	**ICU 9/B/D0**	8218935	1,86	20294868	0,81	2,31
**A20**	**CR11/B/D7**	**ICU 9/B/D7**	7490956	1,69	21152021	0,84	2,02
**A21**	**CR11/T/D0**	**ICU 9/T/D0**	d.n.r.	d.n.r.	d.n.r.	d.n.r.	d.n.r.
**A22**	**CR11/T/D7**	**ICU 9/T/D7**	d.n.r.	d.n.r.	d.n.r.	d.n.r.	d.n.r.
	calibrator		4398401	0,99	24177834	0,96	1,04

Tom20

	WB ID	Control/Patient ID	protein density	protein normalized to calibrator	GAPDH density	GAPDH normalized to calibrator	protein/GAPDH
**GEL 2/ set B**		**cal. mean**	4671836		**cal. mean** 28899095,5		
	calibrator		4140040	0,89	27854350	0,96	0,92
**B1**	**H4/B**	**Control 12/B**	11238210	2,41	28818080	1,00	2,41
**B2**	**H4/T**	**Control 12/T**	d.n.r.	d.n.r.	d.n.r.	d.n.r.	d.n.r.
**B3**	**H5/B**	**Control 6/B**	7582886	1,62	29889932	1,03	1,57
**B4**	**H5/T**	**Control 6/T**	d.n.r.	d.n.r.	d.n.r.	d.n.r.	d.n.r.
**B5**	**H6/B**	**Control 8/B**	5051560	1,08	23959672	0,83	1,30
**B6**	**H6/T**	**Control 8/T**	d.n.r.	d.n.r.	d.n.r.	d.n.r.	d.n.r.
**B7**	**Sample Y/B**	**Sample Y/B**	excl.	#####	29444064	1,02	########
**B8**	**Sample Y/T**	**Sample Y/T**	excl.	#####	27218925	0,94	########
**B9**	**Sample Z/B**	**Sample Z/B**	excl.	#####	25503469	0,88	########
**B10**	**Sample Z/T**	**Sample Z/T**	excl.	#####	22796640	0,79	########
**B11**	**CR9/B/D0**	**ICU 3/B/D0**	6755616	1,45	26684496	0,92	1,57
**B12**	**CR9/B/D7**	**ICU 3/B/D7**	8607016	1,84	27844674	0,96	1,91
**B13**	**CR9/T/D0**	**ICU 3/T/D0**	d.n.r.	d.n.r.	d.n.r.	d.n.r.	d.n.r.
**B14**	**CR9/T/D7**	**ICU 3/T/D7**	d.n.r.	d.n.r.	d.n.r.	d.n.r.	d.n.r.
**B15**	**CR12/B/D0**	**ICU 20/B/D0**	7880922	1,69	29392896	1,02	1,66
**B16**	**CR12/B/D7**	**ICU 20/B/D7**	11616288	2,49	29492584	1,02	2,44
**B17**	**CR12/T/D0**	**ICU 20/T/D0**	d.n.r.	d.n.r.	d.n.r.	d.n.r.	d.n.r.
**B18**	**CR12/T/D7**	**ICU 20/T/D7**	d.n.r.	d.n.r.	d.n.r.	d.n.r.	d.n.r.
**B19**	**CR13/B/D0**	**ICU 22/B/D0**	10723770	2,30	28194210	0,98	2,35
**B20**	**CR13/B/D7**	**ICU 22/B/D7**	11730645	2,51	25151087	0,87	2,89
**B21**	**CR13/T/D0**	**ICU 22/T/D0**	d.n.r.	d.n.r.	d.n.r.	d.n.r.	d.n.r.
**B22**	**CR13/T/D7**	**ICU 22/T/D7**	d.n.r.	d.n.r.	d.n.r.	d.n.r.	d.n.r.
	calibrator		5203632	1,11	29943841	1,04	1,07

Tom20

	WB ID	Control/Patient ID	protein density	protein normalized to calibrator	GAPDH density	GAPDH normalized to calibrator	protein/GAPDH
GEL 3/ set C		cal. mean	11802124		cal. mean	34079936	
	calibrator		12292398	1,04	34138962	1,00	1,04
**C1**	**H15/B**	**Control 2/B**	15812774	1,34	29730870	0,87	1,54
**C2**	**H15/T**	**Control 2/T**	d.n.r.	d.n.r.	d.n.r.	d.n.r.	d.n.r.
**C3**	**H16/B**	**Control 14/B**	19865736	1,68	30098518	0,88	1,91
**C4**	**H16/T**	**Control 14/T**	d.n.r.	d.n.r.	d.n.r.	d.n.r.	d.n.r.
**C5**	**H17/B**	**Control 10/B**	20022517	1,70	20994664	0,62	2,75
**C6**	**H17/T**	**Control 10/T**	d.n.r.	d.n.r.	d.n.r.	d.n.r.	d.n.r.
**C7**	**CR3/B/D0**	**ICU 5/B/D0**	8461645	0,72	22660589	0,66	1,08
**C8**	**CR3/B/D7**	**ICU 5/B/D7**	9581264	0,81	18025875	0,53	1,53
**C9**	**CR3/T/D0**	**ICU 5/T/D0**	d.n.r.	d.n.r.	d.n.r.	d.n.r.	d.n.r.
**C10**	**CR3/T/D7**	**ICU 5/T/D7**	d.n.r.	d.n.r.	d.n.r.	d.n.r.	d.n.r.
**C11**	**CR5/B/D0**	**ICU 8/B/D0**	6937680	0,59	19994035	0,59	1,00
**C12**	**CR5/B/D7**	**ICU 8/B/D7**	8281176	0,70	17391984	0,51	1,37
**C13**	**CR5/T/D0**	**ICU 8/T/D0**	d.n.r.	d.n.r.	d.n.r.	d.n.r.	d.n.r.
**C14**	**CR5/T/D7**	**ICU 8/T/D7**	d.n.r.	d.n.r.	d.n.r.	d.n.r.	d.n.r.
**C15**	**CR7/B/D0**	**ICU 6/B/D0**	11165085	0,95	22085112	0,65	1,46
**C16**	**CR7/B/D7**	**ICU 6/B/D7**	9844808	0,83	18388750	0,54	1,55
**C17**	**CD7/T/D0**	**ICU 6/T/D0**	d.n.r.	d.n.r.	d.n.r.	d.n.r.	d.n.r.
**C18**	**CR7/T/D7**	**ICU 6/T/D7**	d.n.r.	d.n.r.	d.n.r.	d.n.r.	d.n.r.
**C19**	**CR20/B/D0**	**ICU 7/B/D0**	9816420	0,83	24852613	0,73	1,14
**C20**	**CR20/B/D7**	**ICU 7/B/D7**	13241936	1,12	25778115	0,76	1,48
**C21**	**CR20/T/D0**	**ICU 7/T/D0**	d.n.r.	d.n.r.	d.n.r.	d.n.r.	d.n.r.
**C22**	**CR20/T/D7**	**ICU 7/T/D7**	d.n.r.	d.n.r.	d.n.r.	d.n.r.	d.n.r.
	calibrator		11311850	0,96	34020910	1,00	0,96

Tom20

	WB ID	Control/Patient ID	protein density	protein normalized to calibrator	GAPDH density	GAPDH normalized to calibrator	protein/GAPDH
**GEL 4/ set D**		cal. mean	8388701		cal. mean	35190284	
	calibrator		6974050	0,83	37167787	1,06	0,79
**D1**	**H2/B**	**Control 4/B**	10812209	1,29	30949908	0,88	1,47
**D2**	**H2/T**	**Control 4/T**	d.n.r.	d.n.r.	d.n.r.	d.n.r.	d.n.r.
**D3**	**H7/B**	**Control 13/B**	12141984	1,45	31992571	0,91	1,59
**D4**	**H7/T**	**Control 13/T**	d.n.r.	d.n.r.	d.n.r.	d.n.r.	d.n.r.
**D5**	**H8/B**	**Control 18/B**	17289900	2,06	24406066	0,69	2,97
**D6**	**H8/T**	**Control 18/T**	d.n.r.	d.n.r.	d.n.r.	d.n.r.	d.n.r.
**D7**	**H11/B**	**Control 19/B**	15443580	1,84	22372376	0,64	2,90
**D8**	**H11/T**	**Control 19/T**	d.n.r.	d.n.r.	d.n.r.	d.n.r.	d.n.r.
**D9**	**H19/B**	**Control 5/B**	12351350	1,47	18229138	0,52	2,84
**D10**	**H19/T**	**Control 5/T**	d.n.r.	d.n.r.	d.n.r.	d.n.r.	d.n.r.
**D11**	**CR2/B/D0**	**ICU 14/B/D0**	13085616	1,56	17679473	0,50	3,10
**D12**	**CR2/B/D7**	**ICU 14/B/D7**	13055130	1,56	18461459	0,52	2,97
**D13**	**CR2/B/D180**	**ICU 14/B/D180**	13513769	1,61	19948365	0,57	2,84
**D14**	**CR2/T/D0**	**ICU 14/T/D0**	d.n.r.	d.n.r.	d.n.r.	d.n.r.	d.n.r.
**D15**	**CR2/T/D7**	**ICU 14/T/D7**	d.n.r.	d.n.r.	d.n.r.	d.n.r.	d.n.r.
**D16**	**CR2/T/D180**	**ICU 14/T/D180**	d.n.r.	d.n.r.	d.n.r.	d.n.r.	d.n.r.
**D17**	**CR17/B/D0**	**ICU 23/B/D0**	11457082	1,37	22814050	0,65	2,11
**D18**	**CR17/B/D7**	**ICU 23/B/D7**	10009395	1,19	24909295	0,71	1,69
**D19**	**CR17/B/D180**	**ICU 23/B/D180**	8937150	1,07	26712850	0,76	1,40
**D20**	**CR17/T/D0**	**ICU 23/T/D0**	d.n.r.	d.n.r.	d.n.r.	d.n.r.	d.n.r.
**D21**	**CR17/T/D7**	**ICU 23/T/D7**	d.n.r.	d.n.r.	d.n.r.	d.n.r.	d.n.r.
**D22**	**CR17/T/D180**	**ICU 23/T/D180**	d.n.r.	d.n.r.	d.n.r.	d.n.r.	d.n.r.
	calibrator		9803352	1,17	33212781	0,94	1,24

Tom20

	WB ID	Control/Patient ID	protein density	protein normalized to calibrator	GAPDH density	GAPDH normalized to calibrator	protein/GAPDH
**GEL 5/ set E**		cal. mean	12505153		cal. mean	13612646,5	
	calibrator		13380434	1,07	13056250	0,96	1,12
**E1**	**H12/B**	**Control 7/B**	20682106	1,65	15730101	1,16	1,43
**E2**	**H12/T**	**Control 7/T**	d.n.r.	d.n.r.	d.n.r.	d.n.r.	d.n.r.
**E3**	**H13/B**	**Control 1/B**	6727140	0,54	17836110	1,31	0,41
**E4**	**H13/T**	**Control 1/T**	d.n.r.	d.n.r.	d.n.r.	d.n.r.	d.n.r.
**E5**	**H18/B**	**Control 11/B**	12127824	0,97	17137798	1,26	0,77
**E6**	**H18/T**	**Control 11/T**	d.n.r.	d.n.r.	d.n.r.	d.n.r.	d.n.r.
**E7**	**CR6/B/D0**	**ICU 21/B/D0**	13466267	1,08	16121115	1,18	0,91
**E8**	**CR6/B/D7**	**ICU 21/B/D7**	7086624	0,57	12428020	0,91	0,62
**E9**	**CR6/T/D0**	**ICU 21/T/D0**	d.n.r.	d.n.r.	d.n.r.	d.n.r.	d.n.r.
**E10**	**CR6/T/D7**	**ICU 21/T/D7**	d.n.r.	d.n.r.	d.n.r.	d.n.r.	d.n.r.
**E11**	**CR14/B/D0**	**ICU 10/B/D0**	14370768	1,15	12644640	0,93	1,24
**E12**	**CR14/B/D7**	**ICU 10/B/D7**	9519427	0,76	13423200	0,99	0,77
**E13**	**CR14/T/D0**	**ICU 10/T/D0**	d.n.r.	d.n.r.	d.n.r.	d.n.r.	d.n.r.
**E14**	**CR14/T/D7**	**ICU 10/T/D7**	d.n.r.	d.n.r.	d.n.r.	d.n.r.	d.n.r.
**E15**	**Sample W/B/D0**	**Sample W/B/D0**	excl.	#####	13286318	0,98	########
**E16**	**Sample W/B/D7**	**Sample W/B/D7**	excl.	#####	14503083	1,07	########
**E17**	**Sample W/T/D0**	**Sample W/T/D0**	excl.	#####	15358237	1,13	########
**E18**	**Sample W/T/D7**	**Sample W/T/D7**	excl.	#####	14918082	1,10	########
**E19**	**CR19/B/D0**	**ICU 15/B/D0**	23031312	1,84	18376065	1,35	1,36
**E20**	**CR19/B/D7**	**ICU 15/B/D7**	18353622	1,47	18750060	1,38	1,07
**E21**	**CR19/T/D0**	**ICU 15/T/D0**	d.n.r.	d.n.r.	d.n.r.	d.n.r.	d.n.r.
**E22**	**CR19/T/D7**	**ICU 15/T/D7**	d.n.r.	d.n.r.	d.n.r.	d.n.r.	d.n.r.
	calibrator		11629872	0,93	14169043	1,04	0,89

Tom20

	WB ID	Control/Patient ID	protein density	protein normalized to calibrator	GAPDH density	GAPDH normalized to calibrator	protein/GAPDH
**GEL 6/ set F**		cal. mean	13395526		cal. mean	24950732	
	calibrator		8776741	0,66	25509639	1,02	0,64
**F1**	**H9/B**	**Control 9/B**	23084964	1,72	32156010	1,29	1,34
**F2**	**H9/T**	**Control 9/T**	d.n.r.	d.n.r.	d.n.r.	d.n.r.	d.n.r.
**F3**	**H10/B**	**Control 15/B**	22475025	1,68	31970444	1,28	1,31
**F4**	**H10/T**	**Control 15/T**	d.n.r.	d.n.r.	d.n.r.	d.n.r.	d.n.r.
**F5**	**H14/B**	**Control 20/B**	22393710	1,67	25759862	1,03	1,62
**F6**	**H14/T**	**Control 20/T**	d.n.r.	d.n.r.	d.n.r.	d.n.r.	d.n.r.
**F7**	**CR1/B/D0**	**ICU 1/B/D0**	12288857	0,92	24703341	0,99	0,93
**F8**	**CR1/B/D7**	**ICU 1/B/D7**	16994474	1,27	22559436	0,90	1,40
**F9**	**CR1/B/D180**	**ICU 1/B/D180**	13326004	0,99	26013983	1,04	0,95
**F10**	**CR1/T/D0**	**ICU 1/T/D0**	d.n.r.	d.n.r.	d.n.r.	d.n.r.	d.n.r.
**F11**	**CR1/T/D7**	**ICU 1/T/D7**	d.n.r.	d.n.r.	d.n.r.	d.n.r.	d.n.r.
**F12**	**CR1/T/D180**	**ICU 1/T/D180**	d.n.r.	d.n.r.	d.n.r.	d.n.r.	d.n.r.
**F13**	**CR18/B/D0**	**ICU 18/B/D0**	7140735	0,53	24749871	0,99	0,54
**F14**	**CR18/B/D7**	**ICU 18/B/D7**	19496475	1,46	21148778	0,85	1,72
**F15**	**CR18/B/D180**	**ICU 18/B/D180**	10199203	0,76	21108311	0,85	0,90
**F16**	**CR18/T/D0**	**ICU 18/T/D0**	d.n.r.	d.n.r.	d.n.r.	d.n.r.	d.n.r.
**F17**	**CR18/T/D7**	**ICU 18/T/D7**	d.n.r.	d.n.r.	d.n.r.	d.n.r.	d.n.r.
**F18**	**CR18/T/D180**	**ICU 18/T/D180**	d.n.r.	d.n.r.	d.n.r.	d.n.r.	d.n.r.
**F19**	**CR15/B/D0**	**ICU 12/B/D0**	13406890	1,00	27652779	1,11	0,90
**F20**	**CR15/B/D7**	**ICU 12/B/D7**	12205260	0,91	28943200	1,16	0,79
**F21**	**CR15/T/D0**	**ICU 12/T/D0**	d.n.r.	d.n.r.	d.n.r.	d.n.r.	d.n.r.
**F22**	**CR15/T/D7**	**ICU 12/T/D7**	d.n.r.	d.n.r.	d.n.r.	d.n.r.	d.n.r.
	calibrator		18014311	1,34	24391825	0,98	1,38

Tom20

	WB ID	Control/Patient ID	protein density	protein normalized to calibrator	GAPDH density	GAPDH normalized to calibrator	protein/GAPDH
**GEL 7/ set ICU 16**		**cal. mean**	12471333	**cal. mean**	40515874		
	calibrator		14151445	1,13	43007588	1,06	
**E15**	**CR16/B/D0**	**ICU 16/B/D0**	22650750	1,82	37231612	0,92	1,98
**E16**	**CR16/B/D7**	**ICU 16/B/D7**	21523344	1,73	33408164	0,82	2,09
**E17**	**CR16/T/D0**	**ICU 16/T/D0**	d.n.r.	d.n.r.	d.n.r.	d.n.r.	d.n.r.
**E18**	**CR16/T/D7**	**ICU 16/T/D7**	d.n.r.	d.n.r.	d.n.r.	d.n.r.	d.n.r.
	calibrator		10791221	0,87	38024160	0,94	

Tom40

	WB ID	Control/Patient ID	protein density	protein normalized to calibrator	GAPDH density	GAPDH normalized to calibrator	protein/GAPDH
GEL 1/ set A		cal. mean	6086490	cal. mean	26252326		
	calibrator		8780756	1,44	27774192	1,06	1,36
A1	H1/B	Control 17/B	13770652	2,26	26549130	1,01	2,24
A2	H1/T	Control 17/T	d.n.r.	d.n.r.	d.n.r.	d.n.r.	d.n.r.
A3	Sample X/B	Sample X/B	excl.	#####	23658300	0,90	########
A4	Sample X/T	Sample X/T	excl.	#####	20212632	0,77	########
A5	H3/B	Control 3/B	8353370	1,37	22203368	0,85	1,62
A6	H3/T	Control 3/T	d.n.r.	d.n.r.	d.n.r.	d.n.r.	d.n.r.
A7	CR4/B/D0	ICU 4/B/D0	9404010	1,55	22318120	0,85	1,82
A8	CR4/B/D7	ICU 4/B/D7	7821932	1,29	21786380	0,83	1,55
A9	CR4/T/D0	ICU 4/T/D0	d.n.r.	d.n.r.	d.n.r.	d.n.r.	d.n.r.
A10	CR4/T/D7	ICU 4/T/D7	d.n.r.	d.n.r.	d.n.r.	d.n.r.	d.n.r.
A11	CR8/B/D0	ICU 11/B/D0	8265970	1,36	24650043	0,94	1,45
A12	CR8/B/D7	ICU 11/B/D7	8713182	1,43	26404414	1,01	1,42
A13	CR8/T/D0	ICU 11/T/D0	d.n.r.	d.n.r.	d.n.r.	d.n.r.	d.n.r.
A14	CR8/T/D7	ICU 11/T/D7	d.n.r.	d.n.r.	d.n.r.	d.n.r.	d.n.r.
A15	CR10/B/D0	ICU 13/B/D0	5147124	0,85	21712328	0,83	1,02
A16	CR10/B/D7	ICU 13/B/D7	4873470	0,80	23581122	0,90	0,89
A17	CR10/T/D0	ICU 13/T/D0	d.n.r.	d.n.r.	d.n.r.	d.n.r.	d.n.r.
A18	CR10/T/D7	ICU 13/T/D7	d.n.r.	d.n.r.	d.n.r.	d.n.r.	d.n.r.
A19	CR11/B/D0	ICU 9/B/D0	2436804	0,40	23880825	0,91	0,44
A20	CR11/B/D7	ICU 9/B/D7	2069080	0,34	21025925	0,80	0,42
A21	CR11/T/D0	ICU 9/T/D0	d.n.r.	d.n.r.	d.n.r.	d.n.r.	d.n.r.
A22	CR11/T/D7	ICU 9/T/D7	d.n.r.	d.n.r.	d.n.r.	d.n.r.	d.n.r.
	calibrator		3392224	0,56	24730460	0,94	0,59

Tom40

	WB ID	Control/Patient ID	protein density	protein normalized to calibrator	GAPDH density	GAPDH normalized to calibrator	protein/GAPDH
**GEL 2/ set B**		cal. mean	5630952,5		cal. mean	28899095,5	
	calibrator		8553812	1,52	27854350	0,96	1,58
**B1**	**H4/B**	**Control 12/B**	11642840	2,07	28818080	1,00	2,07
**B2**	**H4/T**	**Control 12/T**	d.n.r.	d.n.r.	d.n.r.	d.n.r.	d.n.r.
**B3**	**H5/B**	**Control 6/B**	6467576	1,15	29889932	1,03	1,11
**B4**	**H5/T**	**Control 6/T**	d.n.r.	d.n.r.	d.n.r.	d.n.r.	d.n.r.
**B5**	**H6/B**	**Control 8/B**	8627696	1,53	23959672	0,83	1,85
**B6**	**H6/T**	**Control 8/T**	d.n.r.	d.n.r.	d.n.r.	d.n.r.	d.n.r.
**B7**	**Sample Y/B**	**Sample Y/B**	excl.	#####	29444064	1,02	########
**B8**	**Sample Y/T**	**Sample Y/T**	excl.	#####	27218925	0,94	########
**B9**	**Sample Z/B**	**Sample Z/B**	excl.	#####	25503469	0,88	########
**B10**	**Sample Z/T**	**Sample Z/T**	excl.	#####	22796640	0,79	########
**B11**	**CR9/B/D0**	**ICU 3/B/D0**	7114545	1,26	26684496	0,92	1,37
**B12**	**CR9/B/D7**	**ICU 3/B/D7**	6541366	1,16	27844674	0,96	1,21
**B13**	**CR9/T/D0**	**ICU 3/T/D0**	d.n.r.	d.n.r.	d.n.r.	d.n.r.	d.n.r.
**B14**	**CR9/T/D7**	**ICU 3/T/D7**	d.n.r.	d.n.r.	d.n.r.	d.n.r.	d.n.r.
**B15**	**CR12/B/D0**	**ICU 20/B/D0**	6018452	1,07	29392896	1,02	1,05
**B16**	**CR12/B/D7**	**ICU 20/B/D7**	7390797	1,31	29492584	1,02	1,29
**B17**	**CR12/T/D0**	**ICU 20/T/D0**	d.n.r.	d.n.r.	d.n.r.	d.n.r.	d.n.r.
**B18**	**CR12/T/D7**	**ICU 20/T/D7**	d.n.r.	d.n.r.	d.n.r.	d.n.r.	d.n.r.
**B19**	**CR13/B/D0**	**ICU 22/B/D0**	5199656	0,92	28194210	0,98	0,95
**B20**	**CR13/B/D7**	**ICU 22/B/D7**	4362737	0,77	25151087	0,87	0,89
**B21**	**CR13/T/D0**	**ICU 22/T/D0**	d.n.r.	d.n.r.	d.n.r.	d.n.r.	d.n.r.
**B22**	**CR13/T/D7**	**ICU 22/T/D7**	d.n.r.	d.n.r.	d.n.r.	d.n.r.	d.n.r.
	calibrator		2708093	0,48	29943841	1,04	0,46

Tom40

GEL 3/ set C — cal. mean (protein density): 2093057 — cal. mean (GAPDH density): 33101325,5

	WB ID	Control/Patient ID	protein density	protein normalized to calibrator	GAPDH density	GAPDH normalized to calibrator	protein/GAPDH
	calibrator		2885850	1,38	39634150	1,20	1,15
**C1**	**H15/B**	**Control 2/B**	4144545	1,98	41027124	1,24	1,60
**C2**	**H15/T**	**Control 2/T**	d.n.r.	d.n.r.	d.n.r.	d.n.r.	d.n.r.
**C3**	**H16/B**	**Control 14/B**	9156690	4,37	36119890	1,09	4,01
**C4**	**H16/T**	**Control 14/T**	d.n.r.	d.n.r.	d.n.r.	d.n.r.	d.n.r.
**C5**	**H17/B**	**Control 10/B**	13209660	6,31	32155610	0,97	6,50
**C6**	**H17/T**	**Control 10/T**	d.n.r.	d.n.r.	d.n.r.	d.n.r.	d.n.r.
**C7**	**CR3/B/D0**	**ICU 5/B/D0**	3349980	1,60	31662000	0,96	1,67
**C8**	**CR3/B/D7**	**ICU 5/B/D7**	4827375	2,31	32580420	0,98	2,34
**C9**	**CR3/T/D0**	**ICU 5/T/D0**	d.n.r.	d.n.r.	d.n.r.	d.n.r.	d.n.r.
**C10**	**CR3/T/D7**	**ICU 5/T/D7**	d.n.r.	d.n.r.	d.n.r.	d.n.r.	d.n.r.
**C11**	**CR5/B/D0**	**ICU 8/B/D0**	4450635	2,13	35399622	1,07	1,99
**C12**	**CR5/B/D7**	**ICU 8/B/D7**	4859595	2,32	28886670	0,87	2,66
**C13**	**CR5/T/D0**	**ICU 8/T/D0**	d.n.r.	d.n.r.	d.n.r.	d.n.r.	d.n.r.
**C14**	**CR5/T/D7**	**ICU 8/T/D7**	d.n.r.	d.n.r.	d.n.r.	d.n.r.	d.n.r.
**C15**	**CR7/B/D0**	**ICU 6/B/D0**	7071525	3,38	25648220	0,77	4,36
**C16**	**CR7/B/D7**	**ICU 6/B/D7**	9559890	4,57	24801885	0,75	6,10
**C17**	**CD7/T/D0**	**ICU 6/T/D0**	d.n.r.	d.n.r.	d.n.r.	d.n.r.	d.n.r.
**C18**	**CR7/T/D7**	**ICU 6/T/D7**	d.n.r.	d.n.r.	d.n.r.	d.n.r.	d.n.r.
**C19**	**CR20/B/D0**	**ICU 7/B/D0**	6109246	2,92	23268402	0,70	4,15
**C20**	**CR20/B/D7**	**ICU 7/B/D7**	4242288	2,03	26161994	0,79	2,56
**C21**	**CR20/T/D0**	**ICU 7/T/D0**	d.n.r.	d.n.r.	d.n.r.	d.n.r.	d.n.r.
**C22**	**CR20/T/D7**	**ICU 7/T/D7**	d.n.r.	d.n.r.	d.n.r.	d.n.r.	d.n.r.
	calibrator		1300264	0,62	26568501	0,80	0,77

Tom40

	WB ID	Control/Patient ID	protein density	protein normalized to calibrator	GAPDH density	GAPDH normalized to calibrator	protein/GAPDH
GEL 4/ set D		cal. mean	2148817,5		cal. mean	37613053,5	
	calibrator		2035980	0,95	35832900	0,95	0,99
D1	H2/B	Control 4/B	2928996	1,36	33954570	0,90	1,51
D2	H2/T	Control 4/T	d.n.r.	d.n.r.	d.n.r.	d.n.r.	d.n.r.
D3	H7/B	Control 13/B	7108470	3,31	37204616	0,99	3,34
D4	H7/T	Control 13/T	d.n.r.	d.n.r.	d.n.r.	d.n.r.	d.n.r.
D5	H8/B	Control 18/B	9924885	4,62	27370416	0,73	6,35
D6	H8/T	Control 18/T	d.n.r.	d.n.r.	d.n.r.	d.n.r.	d.n.r.
D7	H11/B	Control 19/B	13499280	6,28	33493056	0,89	7,05
D8	H11/T	Control 19/T	d.n.r.	d.n.r.	d.n.r.	d.n.r.	d.n.r.
D9	H19/B	Control 5/B	10860120	5,05	29291952	0,78	6,49
D10	H19/T	Control 5/T	d.n.r.	d.n.r.	d.n.r.	d.n.r.	d.n.r.
D11	CR2/B/D0	ICU 14/B/D0	12405105	5,77	26753646	0,71	8,12
D12	CR2/B/D7	ICU 14/B/D7	11848950	5,51	28843383	0,77	7,19
D13	CR2/B/D180	ICU 14/B/D180	16130970	7,51	26981280	0,72	10,46
D14	CR2/T/D0	ICU 14/T/D0	d.n.r.	d.n.r.	d.n.r.	d.n.r.	d.n.r.
D15	CR2/T/D7	ICU 14/T/D7	d.n.r.	d.n.r.	d.n.r.	d.n.r.	d.n.r.
D16	CR2/T/D180	ICU 14/T/D180	d.n.r.	d.n.r.	d.n.r.	d.n.r.	d.n.r.
D17	CR17/B/D0	ICU 23/B/D0	8504100	3,96	28709933	0,76	5,18
D18	CR17/B/D7	ICU 23/B/D7	7105770	3,31	28537335	0,76	4,36
D19	CR17/B/D180	ICU 23/B/D180	5527215	2,57	29401155	0,78	3,29
D20	CR17/T/D0	ICU 23/T/D0	d.n.r.	d.n.r.	d.n.r.	d.n.r.	d.n.r.
D21	CR17/T/D7	ICU 23/T/D7	d.n.r.	d.n.r.	d.n.r.	d.n.r.	d.n.r.
D22	CR17/T/D180	ICU 23/T/D180	d.n.r.	d.n.r.	d.n.r.	d.n.r.	d.n.r.
	calibrator		2261655	1,05	39393207	1,05	1,00

Tom40

	WB ID	Control/Patient ID	protein density	protein normalized to calibrator	GAPDH density	GAPDH normalized to calibrator	protein/GAPDH
**GEL 5/ set E**		**cal. mean**	7591203		**cal. mean** 33459356		
	calibrator		6328791	0,83	32367400	0,97	0,86
**E1**	**H12/B**	**Control 7/B**	2810480	0,37	33789330	1,01	0,37
**E2**	**H12/T**	**Control 7/T**	d.n.r.	d.n.r.	d.n.r.	d.n.r.	d.n.r.
**E3**	**H13/B**	**Control 1/B**	405720	0,05	32255844	0,96	0,06
**E4**	**H13/T**	**Control 1/T**	d.n.r.	d.n.r.	d.n.r.	d.n.r.	d.n.r.
**E5**	**H18/B**	**Control 11/B**	1587105	0,21	27424188	0,82	0,26
**E6**	**H18/T**	**Control 11/T**	d.n.r.	d.n.r.	d.n.r.	d.n.r.	d.n.r.
**E7**	**CR6/B/D0**	**ICU 21/B/D0**	2641995	0,35	29010256	0,87	0,40
**E8**	**CR6/B/D7**	**ICU 21/B/D7**	2254185	0,30	22604584	0,68	0,44
**E9**	**CR6/T/D0**	**ICU 21/T/D0**	d.n.r.	d.n.r.	d.n.r.	d.n.r.	d.n.r.
**E10**	**CR6/T/D7**	**ICU 21/T/D7**	d.n.r.	d.n.r.	d.n.r.	d.n.r.	d.n.r.
**E11**	**CR14/B/D0**	**ICU 10/B/D0**	6847200	0,90	24246002	0,72	1,24
**E12**	**CR14/B/D7**	**ICU 10/B/D7**	7173450	0,94	26351913	0,79	1,20
**E13**	**CR14/T/D0**	**ICU 10/T/D0**	d.n.r.	d.n.r.	d.n.r.	d.n.r.	d.n.r.
**E14**	**CR14/T/D7**	**ICU 10/T/D7**	d.n.r.	d.n.r.	d.n.r.	d.n.r.	d.n.r.
**E15**	**Sample W/B/D0**	**Sample W/B/D0**	excl.	#####	29617830	0,89	########
**E16**	**Sample W/B/D7**	**Sample W/B/D7**	excl.	#####	26893768	0,80	########
**E17**	**Sample W/T/D0**	**Sample W/T/D0**	excl.	#####	29597504	0,88	########
**E18**	**Sample W/T/D7**	**Sample W/T/D7**	excl.	#####	32157358	0,96	########
**E19**	**CR19/B/D0**	**ICU 15/B/D0**	13346055	1,76	32525266	0,97	1,81
**E20**	**CR19/B/D7**	**ICU 15/B/D7**	11550240	1,52	38332904	1,15	1,33
**E21**	**CR19/T/D0**	**ICU 15/T/D0**	d.n.r.	d.n.r.	d.n.r.	d.n.r.	d.n.r.
**E22**	**CR19/T/D7**	**ICU 15/T/D7**	d.n.r.	d.n.r.	d.n.r.	d.n.r.	d.n.r.
	calibrator		8853615	1,17	34551312	1,03	1,13

Tom40

	WB ID	Control/Patient ID	protein density	protein normalized to calibrator	GAPDH density	GAPDH normalized to calibrator	protein/GAPDH
GEL 6/ set F		cal. mean	2152623		cal. mean	37954347	
	calibrator		2223522	1,03	26550450	0,70	1,48
F1	H9/B	Control 9/B	2249889	1,05	30236175	0,80	1,31
F2	H9/T	Control 9/T	d.n.r.	d.n.r.	d.n.r.	d.n.r.	d.n.r.
F3	H10/B	Control 15/B	4566156	2,12	28345900	0,75	2,84
F4	H10/T	Control 15/T	d.n.r.	d.n.r.	d.n.r.	d.n.r.	d.n.r.
F5	H14/B	Control 20/B	3759345	1,75	27331912	0,72	2,43
F6	H14/T	Control 20/T	d.n.r.	d.n.r.	d.n.r.	d.n.r.	d.n.r.
F7	CR1/B/D0	ICU 1/B/D0	3697920	1,72	26053802	0,69	2,50
F8	CR1/B/D7	ICU 1/B/D7	7857900	3,65	27329985	0,72	5,07
F9	CR1/B/D180	ICU 1/B/D180	5578020	2,59	29696880	0,78	3,31
F10	CR1/T/D0	ICU 1/T/D0	d.n.r.	d.n.r.	d.n.r.	d.n.r.	d.n.r.
F11	CR1/T/D7	ICU 1/T/D7	d.n.r.	d.n.r.	d.n.r.	d.n.r.	d.n.r.
F12	CR1/T/D180	ICU 1/T/D180	d.n.r.	d.n.r.	d.n.r.	d.n.r.	d.n.r.
F13	CR18/B/D0	ICU 18/B/D0	5830965	2,71	31147879	0,82	3,30
F14	CR18/B/D7	ICU 18/B/D7	9902025	4,60	34315678	0,90	5,09
F15	CR18/B/D180	ICU 18/B/D180	7274115	3,38	36800640	0,97	3,49
F16	CR18/T/D0	ICU 18/T/D0	d.n.r.	d.n.r.	d.n.r.	d.n.r.	d.n.r.
F17	CR18/T/D7	ICU 18/T/D7	d.n.r.	d.n.r.	d.n.r.	d.n.r.	d.n.r.
F18	CR18/T/D180	ICU 18/T/D180	d.n.r.	d.n.r.	d.n.r.	d.n.r.	d.n.r.
F19	CR15/B/D0	ICU 12/B/D0	1745865	0,81	45890106	1,21	0,67
F20	CR15/B/D7	ICU 12/B/D7	2874105	1,34	39184088	1,03	1,29
F21	CR15/T/D0	ICU 12/T/D0	d.n.r.	d.n.r.	d.n.r.	d.n.r.	d.n.r.
F22	CR15/T/D7	ICU 12/T/D7	d.n.r.	d.n.r.	d.n.r.	d.n.r.	d.n.r.
	calibrator		2081724	0,97	49358244	1,30	0,74

Tom40

	WB ID	Control/Patient ID	protein density	protein normalized to calibrator		GAPDH density	GAPDH normalized to calibrator	protein/GAPDH
GEL 7/ set ICU 16		cal. mean	5265944		cal. mean	40515874		
	calibrator		6566256	1,25		43007588	1,06	
E15	CR16/B/D0	ICU 16/B/D0	10411042	1,98		37231612	0,92	2,15
E16	CR16/B/D7	ICU 16/B/D7	6811542	1,29		33408164	0,82	1,57
E17	CR16/T/D0	ICU 16/T/D0	d.n.r.	d.n.r.		d.n.r.	d.n.r.	d.n.r.
E18	CR16/T/D7	ICU 16/T/D7	d.n.r.	d.n.r.		d.n.r.	d.n.r.	d.n.r.
	calibrator		3965632	0,75		38024160	0,94	

P-DRP1 (Ser 637)

	WB ID	Control/Patient ID	protein density	protein normalized to calibrator		GAPDH density	GAPDH normalized to calibrator	protein/GAPDH
GEL 1/ set A		cal. mean	2992300		cal. mean	32397000		
	calibrator		2451386	0,82		34486272	1,06	0,77
A1	H1/B	Control 17/B	4011660	1,34		36056208	1,11	1,20
A2	H1/T	Control 17/T	d.n.r.	d.n.r.		d.n.r.	d.n.r.	d.n.r.
A3	Sample X/B	Sample X/B	excl.	#####		27900540	0,86	########
A4	Sample X/T	Sample X/T	excl.	#####		28724784	0,89	########
A5	H3/B	Control 3/B	5213318	1,74		28793205	0,89	1,96
A6	H3/T	Control 3/T	d.n.r.	d.n.r.		d.n.r.	d.n.r.	d.n.r.
A7	CR4/B/D0	ICU 4/B/D0	2710412	0,91		24152530	0,75	1,21
A8	CR4/B/D7	ICU 4/B/D7	4764220	1,59		22376194	0,69	2,31
A9	CR4/T/D0	ICU 4/T/D0	d.n.r.	d.n.r.		d.n.r.	d.n.r.	d.n.r.
A10	CR4/T/D7	ICU 4/T/D7	d.n.r.	d.n.r.		d.n.r.	d.n.r.	d.n.r.
A11	CR8/B/D0	ICU 11/B/D0	8360684	2,79		23807098	0,73	3,80
A12	CR8/B/D7	ICU 11/B/D7	8423566	2,82		24407424	0,75	3,74
A13	CR8/T/D0	ICU 11/T/D0	d.n.r.	d.n.r.		d.n.r.	d.n.r.	d.n.r.
A14	CR8/T/D7	ICU 11/T/D7	d.n.r.	d.n.r.		d.n.r.	d.n.r.	d.n.r.
A15	CR10/B/D0	ICU 13/B/D0	1995296	0,67		23284992	0,72	0,93
A16	CR10/B/D7	ICU 13/B/D7	2249308	0,75		23631488	0,73	1,03
A17	CR10/T/D0	ICU 13/T/D0	d.n.r.	d.n.r.		d.n.r.	d.n.r.	d.n.r.
A18	CR10/T/D7	ICU 13/T/D7	d.n.r.	d.n.r.		d.n.r.	d.n.r.	d.n.r.
A19	CR11/B/D0	ICU 9/B/D0	2218304	0,74		33965550	1,05	0,71
A20	CR11/B/D7	ICU 9/B/D7	2166554	0,72		32601396	1,01	0,72
A21	CR11/T/D0	ICU 9/T/D0	d.n.r.	d.n.r.		d.n.r.	d.n.r.	d.n.r.
A22	CR11/T/D7	ICU 9/T/D7	d.n.r.	d.n.r.		d.n.r.	d.n.r.	d.n.r.
	calibrator		3533214	1,18		30307728	0,94	1,26

P-DRP1 (Ser 637)

	WB ID	Control/Patient ID	protein density	protein normalized to calibrator	GAPDH density	GAPDH normalized to calibrator	protein/GAPDH
GEL 2/ set B		cal. mean	3298747,5		cal. mean	27865678,5	
	calibrator		3525975	1,07	30163392	1,08	0,99
**B1**	**H4/B**	**Control 12/B**	5400450	1,64	27105885	0,97	1,68
**B2**	**H4/T**	**Control 12/T**	d.n.r.	d.n.r.	d.n.r.	d.n.r.	d.n.r.
**B3**	**H5/B**	**Control 6/B**	4220055	1,28	28410290	1,02	1,25
**B4**	**H5/T**	**Control 6/T**	d.n.r.	d.n.r.	d.n.r.	d.n.r.	d.n.r.
**B5**	**H6/B**	**Control 8/B**	4553415	1,38	20371736	0,73	1,89
**B6**	**H6/T**	**Control 8/T**	d.n.r.	d.n.r.	d.n.r.	d.n.r.	d.n.r.
**B7**	**Sample Y/B**	**Sample Y/B**	excl.	#####	22090275	0,79	########
**B8**	**Sample Y/T**	**Sample Y/T**	excl.	#####	19395114	0,70	########
**B9**	**Sample Z/B**	**Sample Z/B**	excl.	#####	20887034	0,75	########
**B10**	**Sample Z/T**	**Sample Z/T**	excl.	#####	18124046	0,65	########
**B11**	**CR9/B/D0**	**ICU 3/B/D0**	2910510	0,88	21057888	0,76	1,17
**B12**	**CR9/B/D7**	**ICU 3/B/D7**	4751685	1,44	20873640	0,75	1,92
**B13**	**CR9/T/D0**	**ICU 3/T/D0**	d.n.r.	d.n.r.	d.n.r.	d.n.r.	d.n.r.
**B14**	**CR9/T/D7**	**ICU 3/T/D7**	d.n.r.	d.n.r.	d.n.r.	d.n.r.	d.n.r.
**B15**	**CR12/B/D0**	**ICU 20/B/D0**	5427585	1,65	26873664	0,96	1,71
**B16**	**CR12/B/D7**	**ICU 20/B/D7**	2650590	0,80	23672428	0,85	0,95
**B17**	**CR12/T/D0**	**ICU 20/T/D0**	d.n.r.	d.n.r.	d.n.r.	d.n.r.	d.n.r.
**B18**	**CR12/T/D7**	**ICU 20/T/D7**	d.n.r.	d.n.r.	d.n.r.	d.n.r.	d.n.r.
**B19**	**CR13/B/D0**	**ICU 22/B/D0**	2379375	0,72	22632840	0,81	0,89
**B20**	**CR13/B/D7**	**ICU 22/B/D7**	3458925	1,05	20968515	0,75	1,39
**B21**	**CR13/T/D0**	**ICU 22/T/D0**	d.n.r.	d.n.r.	d.n.r.	d.n.r.	d.n.r.
**B22**	**CR13/T/D7**	**ICU 22/T/D7**	d.n.r.	d.n.r.	d.n.r.	d.n.r.	d.n.r.
	calibrator		3071520	0,93	25567965	0,92	1,01

P-DRP1 (Ser 637)

	WB ID	Control/Patient ID	protein density	protein normalized to calibrator	GAPDH density	GAPDH normalized to calibrator	protein/GAPDH
GEL 3/ set C		cal. mean	3258810	***	cal. mean	33101325,5	
	calibrator		3258810	1,00	39634150	1,20	0,84
**C1**	**H15/B**	**Control 2/B**	6009696	1,84	41027124	1,24	1,49
**C2**	**H15/T**	**Control 2/T**	d.n.r.	d.n.r.	d.n.r.	d.n.r.	d.n.r.
**C3**	**H16/B**	**Control 14/B**	2742980	0,84	36119890	1,09	0,77
**C4**	**H16/T**	**Control 14/T**	d.n.r.	d.n.r.	d.n.r.	d.n.r.	d.n.r.
**C5**	**H17/B**	**Control 10/B**	1578510	0,48	32155610	0,97	0,50
**C6**	**H17/T**	**Control 10/T**	d.n.r.	d.n.r.	d.n.r.	d.n.r.	d.n.r.
**C7**	**CR3/B/D0**	**ICU 5/B/D0**	2302785	0,71	31662000	0,96	0,74
**C8**	**CR3/B/D7**	**ICU 5/B/D7**	5476365	1,68	32580420	0,98	1,71
**C9**	**CR3/T/D0**	**ICU 5/T/D0**	d.n.r.	d.n.r.	d.n.r.	d.n.r.	d.n.r.
**C10**	**CR3/T/D7**	**ICU 5/T/D7**	d.n.r.	d.n.r.	d.n.r.	d.n.r.	d.n.r.
**C11**	**CR5/B/D0**	**ICU 8/B/D0**	6957300	2,13	35399622	1,07	2,00
**C12**	**CR5/B/D7**	**ICU 8/B/D7**	16564050	5,08	28886670	0,87	5,82
**C13**	**CR5/T/D0**	**ICU 8/T/D0**	d.n.r.	d.n.r.	d.n.r.	d.n.r.	d.n.r.
**C14**	**CR5/T/D7**	**ICU 8/T/D7**	d.n.r.	d.n.r.	d.n.r.	d.n.r.	d.n.r.
**C15**	**CR7/B/D0**	**ICU 6/B/D0**	2771778	0,85	25648220	0,77	1,10
**C16**	**CR7/B/D7**	**ICU 6/B/D7**	1862775	0,57	24801885	0,75	0,76
**C17**	**CD7/T/D0**	**ICU 6/T/D0**	d.n.r.	d.n.r.	d.n.r.	d.n.r.	d.n.r.
**C18**	**CR7/T/D7**	**ICU 6/T/D7**	d.n.r.	d.n.r.	d.n.r.	d.n.r.	d.n.r.
**C19**	**CR20/B/D0**	**ICU 7/B/D0**	1746585	0,54	23268402	0,70	0,76
**C20**	**CR20/B/D7**	**ICU 7/B/D7**	2664585	0,82	26161994	0,79	1,03
**C21**	**CR20/T/D0**	**ICU 7/T/D0**	d.n.r.	d.n.r.	d.n.r.	d.n.r.	d.n.r.
**C22**	**CR20/T/D7**	**ICU 7/T/D7**	d.n.r.	d.n.r.	d.n.r.	d.n.r.	d.n.r.
	calibrator		967920	0,30	26568501	0,80	0,37

P-DRP1 (Ser 637)

	WB ID	Control/Patient ID	protein density	protein normalized to calibrator	GAPDH density	GAPDH normalized to calibrator	protein/GAPDH
**GEL 4/ set D**		cal. mean	4142352	***	cal. mean	37613053,5	
	calibrator		4142352	1,00	35832900	0,95	1,05
**D1**	**H2/B**	**Control 4/B**	5240970	1,27	33954570	0,90	1,40
**D2**	**H2/T**	**Control 4/T**	d.n.r.	d.n.r.	d.n.r.	d.n.r.	d.n.r.
**D3**	**H7/B**	**Control 13/B**	4746832	1,15	37204616	0,99	1,16
**D4**	**H7/T**	**Control 13/T**	d.n.r.	d.n.r.	d.n.r.	d.n.r.	d.n.r.
**D5**	**H8/B**	**Control 18/B**	5325075	1,29	27370416	0,73	1,77
**D6**	**H8/T**	**Control 18/T**	d.n.r.	d.n.r.	d.n.r.	d.n.r.	d.n.r.
**D7**	**H11/B**	**Control 19/B**	3935925	0,95	33493056	0,89	1,07
**D8**	**H11/T**	**Control 19/T**	d.n.r.	d.n.r.	d.n.r.	d.n.r.	d.n.r.
**D9**	**H19/B**	**Control 5/B**	3130605	0,76	29291952	0,78	0,97
**D10**	**H19/T**	**Control 5/T**	d.n.r.	d.n.r.	d.n.r.	d.n.r.	d.n.r.
**D11**	**CR2/B/D0**	**ICU 14/B/D0**	4192408	1,01	26753646	0,71	1,42
**D12**	**CR2/B/D7**	**ICU 14/B/D7**	2185290	0,53	28843383	0,77	0,69
**D13**	**CR2/B/D180**	**ICU 14/B/D180**	3134745	0,76	26981280	0,72	1,05
**D14**	**CR2/T/D0**	**ICU 14/T/D0**	d.n.r.	d.n.r.	d.n.r.	d.n.r.	d.n.r.
**D15**	**CR2/T/D7**	**ICU 14/T/D7**	d.n.r.	d.n.r.	d.n.r.	d.n.r.	d.n.r.
**D16**	**CR2/T/D180**	**ICU 14/T/D180**	d.n.r.	d.n.r.	d.n.r.	d.n.r.	d.n.r.
**D17**	**CR17/B/D0**	**ICU 23/B/D0**	2814165	0,68	28709933	0,76	0,89
**D18**	**CR17/B/D7**	**ICU 23/B/D7**	4329810	1,05	28537335	0,76	1,38
**D19**	**CR17/B/D180**	**ICU 23/B/D180**	10378035	2,51	29401155	0,78	3,21
**D20**	**CR17/T/D0**	**ICU 23/T/D0**	d.n.r.	d.n.r.	d.n.r.	d.n.r.	d.n.r.
**D21**	**CR17/T/D7**	**ICU 23/T/D7**	d.n.r.	d.n.r.	d.n.r.	d.n.r.	d.n.r.
**D22**	**CR17/T/D180**	**ICU 23/T/D180**	d.n.r.	d.n.r.	d.n.r.	d.n.r.	d.n.r.
	calibrator		2121936	0,51	39393207	1,05	0,49

P-DRP1 (Ser 637)

	WB ID	Control/Patient ID	protein density	protein normalized to calibrator	GAPDH density	GAPDH normalized to calibrator	protein/GAPDH
GEL 5/ set E		cal. mean	4893312		cal. mean	33459356	
	calibrator		4093680	0,84	32367400	0,97	0,86
E1	H12/B	Control 7/B	4672052	0,95	33789330	1,01	0,95
E2	H12/T	Control 7/T	d.n.r.	d.n.r.	d.n.r.	d.n.r.	d.n.r.
E3	H13/B	Control 1/B	8830252	1,80	32255844	0,96	1,87
E4	H13/T	Control 1/T	d.n.r.	d.n.r.	d.n.r.	d.n.r.	d.n.r.
E5	H18/B	Control 11/B	12166020	2,49	27424188	0,82	3,03
E6	H18/T	Control 11/T	d.n.r.	d.n.r.	d.n.r.	d.n.r.	d.n.r.
E7	CR6/B/D0	ICU 21/B/D0	10031310	2,05	29010256	0,87	2,36
E8	CR6/B/D7	ICU 21/B/D7	13332285	2,72	22604584	0,68	4,03
E9	CR6/T/D0	ICU 21/T/D0	d.n.r.	d.n.r.	d.n.r.	d.n.r.	d.n.r.
E10	CR6/T/D7	ICU 21/T/D7	d.n.r.	d.n.r.	d.n.r.	d.n.r.	d.n.r.
E11	CR14/B/D0	ICU 10/B/D0	13113628	2,68	24246002	0,72	3,70
E12	CR14/B/D7	ICU 10/B/D7	11918880	2,44	26351913	0,79	3,09
E13	CR14/T/D0	ICU 10/T/D0	d.n.r.	d.n.r.	d.n.r.	d.n.r.	d.n.r.
E14	CR14/T/D7	ICU 10/T/D7	d.n.r.	d.n.r.	d.n.r.	d.n.r.	d.n.r.
E15	CR16/B/D0	ICU 16/B/D0	4153874	0,85	29617830	0,89	0,96
E16	CR16/B/D7	ICU 16/B/D7	2510910	0,51	26893768	0,80	0,64
E17	CR16/T/D0	ICU 16/T/D0	d.n.r.	d.n.r.	d.n.r.	d.n.r.	d.n.r.
E18	CR16/T/D7	ICU 16/T/D7	d.n.r.	d.n.r.	d.n.r.	d.n.r.	d.n.r.
E19	CR19/B/D0	ICU 15/B/D0	6654870	1,36	32525266	0,97	1,40
E20	CR19/B/D7	ICU 15/B/D7	5248804	1,07	38332904	1,15	0,94
E21	CR19/T/D0	ICU 15/T/D0	d.n.r.	d.n.r.	d.n.r.	d.n.r.	d.n.r.
E22	CR19/T/D7	ICU 15/T/D7	d.n.r.	d.n.r.	d.n.r.	d.n.r.	d.n.r.
	calibrator		5692944	1,16	34551312	1,03	1,13

P-DRP1 (Ser 637)

	WB ID	Control/Patient ID	protein density	protein normalized to calibrator	GAPDH density	GAPDH normalized to calibrator	protein/GAPDH
GEL 6/ set F		cal. mean	2534847,5		cal. mean	37954347	
	calibrator		2912800	1,15	26550450	0,70	1,64
F1	H9/B	Control 9/B	9266985	3,66	30236175	0,80	4,59
F2	H9/T	Control 9/T	d.n.r.	d.n.r.	d.n.r.	d.n.r.	d.n.r.
F3	H10/B	Control 15/B	3815680	1,51	28345900	0,75	2,02
F4	H10/T	Control 15/T	d.n.r.	d.n.r.	d.n.r.	d.n.r.	d.n.r.
F5	H14/B	Control 20/B	6793160	2,68	27331912	0,72	3,72
F6	H14/T	Control 20/T	d.n.r.	d.n.r.	d.n.r.	d.n.r.	d.n.r.
F7	CR1/B/D0	ICU 1/B/D0	4377296	1,73	26053802	0,69	2,52
F8	CR1/B/D7	ICU 1/B/D7	6182944	2,44	27329985	0,72	3,39
F9	CR1/B/D180	ICU 1/B/D180	8239275	3,25	29696880	0,78	4,15
F10	CR1/T/D0	ICU 1/T/D0	d.n.r.	d.n.r.	d.n.r.	d.n.r.	d.n.r.
F11	CR1/T/D7	ICU 1/T/D7	d.n.r.	d.n.r.	d.n.r.	d.n.r.	d.n.r.
F12	CR1/T/D180	ICU 1/T/D180	d.n.r.	d.n.r.	d.n.r.	d.n.r.	d.n.r.
F13	CR18/B/D0	ICU 18/B/D0	9189111	3,63	31147879	0,82	4,42
F14	CR18/B/D7	ICU 18/B/D7	2712380	1,07	34315678	0,90	1,18
F15	CR18/B/D180	ICU 18/B/D180	15312976	6,04	36800640	0,97	6,23
F16	CR18/T/D0	ICU 18/T/D0	d.n.r.	d.n.r.	d.n.r.	d.n.r.	d.n.r.
F17	CR18/T/D7	ICU 18/T/D7	d.n.r.	d.n.r.	d.n.r.	d.n.r.	d.n.r.
F18	CR18/T/D180	ICU 18/T/D180	d.n.r.	d.n.r.	d.n.r.	d.n.r.	d.n.r.
F19	CR15/B/D0	ICU 12/B/D0	8114738	3,20	45890106	1,21	2,65
F20	CR15/B/D7	ICU 12/B/D7	21111413	8,33	39184088	1,03	8,07
F21	CR15/T/D0	ICU 12/T/D0	d.n.r.	d.n.r.	d.n.r.	d.n.r.	d.n.r.
F22	CR15/T/D7	ICU 12/T/D7	d.n.r.	d.n.r.	d.n.r.	d.n.r.	d.n.r.
	calibrator		2156895	0,85	49358244	1,30	0,65

DRP1 total (DRP-1 Ser 637 membrane)

	WB ID	Control/Patient ID	protein density	protein normalized to calibrator	GAPDH density	GAPDH normalized to calibrator	protein/GAPDH
GEL 1/ set A		cal. mean	20447491		cal. mean 32397000		
	calibrator		19851813	0,97	34486272	1,06	0,91
A1	H1/B	Control 17/B	16554152	0,81	36056208	1,11	0,73
A2	H1/T	Control 17/T	d.n.r.	d.n.r.	d.n.r.	d.n.r.	d.n.r.
A3	Sample X/B	Sample X/B	excl.	#####	27900540	0,86	########
A4	Sample X/T	Sample X/T	excl.	#####	28724784	0,89	########
A5	H3/B	Control 3/B	17454345	0,85	28793205	0,89	0,96
A6	H3/T	Control 3/T	d.n.r.	d.n.r.	d.n.r.	d.n.r.	d.n.r.
A7	CR4/B/D0	ICU 4/B/D0	19906695	0,97	24152530	0,75	1,31
A8	CR4/B/D7	ICU 4/B/D7	12477344	0,61	22376194	0,69	0,88
A9	CR4/T/D0	ICU 4/T/D0	d.n.r.	d.n.r.	d.n.r.	d.n.r.	d.n.r.
A10	CR4/T/D7	ICU 4/T/D7	d.n.r.	d.n.r.	d.n.r.	d.n.r.	d.n.r.
A11	CR8/B/D0	ICU 11/B/D0	14319855	0,70	23807098	0,73	0,95
A12	CR8/B/D7	ICU 11/B/D7	17135072	0,84	24407424	0,75	1,11
A13	CR8/T/D0	ICU 11/T/D0	d.n.r.	d.n.r.	d.n.r.	d.n.r.	d.n.r.
A14	CR8/T/D7	ICU 11/T/D7	d.n.r.	d.n.r.	d.n.r.	d.n.r.	d.n.r.
A15	CR10/B/D0	ICU 13/B/D0	14442795	0,71	23284992	0,72	0,98
A16	CR10/B/D7	ICU 13/B/D7	17411714	0,85	23631488	0,73	1,17
A17	CR10/T/D0	ICU 13/T/D0	d.n.r.	d.n.r.	d.n.r.	d.n.r.	d.n.r.
A18	CR10/T/D7	ICU 13/T/D7	d.n.r.	d.n.r.	d.n.r.	d.n.r.	d.n.r.
A19	CR11/B/D0	ICU 9/B/D0	17970682	0,88	33965550	1,05	0,84
A20	CR11/B/D7	ICU 9/B/D7	15427454	0,75	32601396	1,01	0,75
A21	CR11/T/D0	ICU 9/T/D0	d.n.r.	d.n.r.	d.n.r.	d.n.r.	d.n.r.
A22	CR11/T/D7	ICU 9/T/D7	d.n.r.	d.n.r.	d.n.r.	d.n.r.	d.n.r.
	calibrator		21043169	1,03	30307728	0,94	1,10

DRP1 total (DRP-1 Ser 637 membrane)

	WB ID	Control/Patient ID	protein density	protein normalized to calibrator	GAPDH density	GAPDH normalized to calibrator	protein/GAPDH
GEL 2/ set B		cal. mean	19760197		cal. mean	27865678,5	
	calibrator		19240742	0,97	30163392	1,08	0,90
**B1**	**H4/B**	**Control 12/B**	16654302	0,84	27105885	0,97	0,87
**B2**	**H4/T**	**Control 12/T**	d.n.r.	d.n.r.	d.n.r.	d.n.r.	d.n.r.
**B3**	**H5/B**	**Control 6/B**	13381764	0,68	28410290	1,02	0,66
**B4**	**H5/T**	**Control 6/T**	d.n.r.	d.n.r.	d.n.r.	d.n.r.	d.n.r.
**B5**	**H6/B**	**Control 8/B**	16278416	0,82	20371736	0,73	1,13
**B6**	**H6/T**	**Control 8/T**	d.n.r.	d.n.r.	d.n.r.	d.n.r.	d.n.r.
**B7**	**Sample Y/B**	**Sample Y/B**	excl.	#####	22090275	0,79	########
**B8**	**Sample Y/T**	**Sample Y/T**	excl.	#####	19395114	0,70	########
**B9**	**Sample Z/B**	**Sample Z/B**	excl.	#####	20887034	0,75	########
**B10**	**Sample Z/T**	**Sample Z/T**	excl.	#####	18124046	0,65	########
**B11**	**CR9/B/D0**	**ICU 3/B/D0**	15626890	0,79	21057888	0,76	1,05
**B12**	**CR9/B/D7**	**ICU 3/B/D7**	16916776	0,86	20873640	0,75	1,14
**B13**	**CR9/T/D0**	**ICU 3/T/D0**	d.n.r.	d.n.r.	d.n.r.	d.n.r.	d.n.r.
**B14**	**CR9/T/D7**	**ICU 3/T/D7**	d.n.r.	d.n.r.	d.n.r.	d.n.r.	d.n.r.
**B15**	**CR12/B/D0**	**ICU 20/B/D0**	14959154	0,76	26873664	0,96	0,78
**B16**	**CR12/B/D7**	**ICU 20/B/D7**	17087160	0,86	23672428	0,85	1,02
**B17**	**CR12/T/D0**	**ICU 20/T/D0**	d.n.r.	d.n.r.	d.n.r.	d.n.r.	d.n.r.
**B18**	**CR12/T/D7**	**ICU 20/T/D7**	d.n.r.	d.n.r.	d.n.r.	d.n.r.	d.n.r.
**B19**	**CR13/B/D0**	**ICU 22/B/D0**	15853596	0,80	22632840	0,81	0,99
**B20**	**CR13/B/D7**	**ICU 22/B/D7**	17871920	0,90	20968515	0,75	1,20
**B21**	**CR13/T/D0**	**ICU 22/T/D0**	d.n.r.	d.n.r.	d.n.r.	d.n.r.	d.n.r.
**B22**	**CR13/T/D7**	**ICU 22/T/D7**	d.n.r.	d.n.r.	d.n.r.	d.n.r.	d.n.r.
	calibrator		20279652	1,03	25567965	0,92	1,12

DRP1 total (DRP-1 Ser 637 membrane)

	WB ID	Control/Patient ID	protein density	protein normalized to calibrator	GAPDH density	GAPDH normalized to calibrator	protein/GAPDH
GEL 3/ set C		cal. mean	27314950,5		cal. mean	33101325,5	
	calibrator		28575180	1,05	39634150	1,20	0,87
**C1**	**H15/B**	**Control 2/B**	28470870	1,04	41027124	1,24	0,84
**C2**	**H15/T**	**Control 2/T**	d.n.r.	d.n.r.	d.n.r.	d.n.r.	d.n.r.
**C3**	**H16/B**	**Control 14/B**	25753024	0,94	36119890	1,09	0,86
**C4**	**H16/T**	**Control 14/T**	d.n.r.	d.n.r.	d.n.r.	d.n.r.	d.n.r.
**C5**	**H17/B**	**Control 10/B**	31206060	1,14	32155610	0,97	1,18
**C6**	**H17/T**	**Control 10/T**	d.n.r.	d.n.r.	d.n.r.	d.n.r.	d.n.r.
**C7**	**CR3/B/D0**	**ICU 5/B/D0**	18451665	0,68	31662000	0,96	0,71
**C8**	**CR3/B/D7**	**ICU 5/B/D7**	18915705	0,69	32580420	0,98	0,70
**C9**	**CR3/T/D0**	**ICU 5/T/D0**	d.n.r.	d.n.r.	d.n.r.	d.n.r.	d.n.r.
**C10**	**CR3/T/D7**	**ICU 5/T/D7**	d.n.r.	d.n.r.	d.n.r.	d.n.r.	d.n.r.
**C11**	**CR5/B/D0**	**ICU 8/B/D0**	21493485	0,79	35399622	1,07	0,74
**C12**	**CR5/B/D7**	**ICU 8/B/D7**	22927095	0,84	28886670	0,87	0,96
**C13**	**CR5/T/D0**	**ICU 8/T/D0**	d.n.r.	d.n.r.	d.n.r.	d.n.r.	d.n.r.
**C14**	**CR5/T/D7**	**ICU 8/T/D7**	d.n.r.	d.n.r.	d.n.r.	d.n.r.	d.n.r.
**C15**	**CR7/B/D0**	**ICU 6/B/D0**	20380635	0,75	25648220	0,77	0,96
**C16**	**CR7/B/D7**	**ICU 6/B/D7**	20218320	0,74	24801885	0,75	0,99
**C17**	**CD7/T/D0**	**ICU 6/T/D0**	d.n.r.	d.n.r.	d.n.r.	d.n.r.	d.n.r.
**C18**	**CR7/T/D7**	**ICU 6/T/D7**	d.n.r.	d.n.r.	d.n.r.	d.n.r.	d.n.r.
**C19**	**CR20/B/D0**	**ICU 7/B/D0**	22083726	0,81	23268402	0,70	1,15
**C20**	**CR20/B/D7**	**ICU 7/B/D7**	20050704	0,73	26161994	0,79	0,93
**C21**	**CR20/T/D0**	**ICU 7/T/D0**	d.n.r.	d.n.r.	d.n.r.	d.n.r.	d.n.r.
**C22**	**CR20/T/D7**	**ICU 7/T/D7**	d.n.r.	d.n.r.	d.n.r.	d.n.r.	d.n.r.
	calibrator		26054721	0,95	26568501	0,80	1,19

DRP1 total (DRP-1 Ser 637 membrane)

WB ID		Control/Patient ID	protein density	protein normalized to calibrator	GAPDH density	GAPDH normalized to calibrator	protein/GAPDH
GEL 4/ set D		cal. mean	29612937		cal. mean 37613053,5		
	calibrator		32250324	1,09	35832900	0,95	1,14
D1	H2/B	Control 4/B	22447264	0,76	33954570	0,90	0,84
D2	H2/T	Control 4/T	d.n.r.	d.n.r.	d.n.r.	d.n.r.	d.n.r.
D3	H7/B	Control 13/B	32323924	1,09	37204616	0,99	1,10
D4	H7/T	Control 13/T	d.n.r.	d.n.r.	d.n.r.	d.n.r.	d.n.r.
D5	H8/B	Control 18/B	36727826	1,24	27370416	0,73	1,70
D6	H8/T	Control 18/T	d.n.r.	d.n.r.	d.n.r.	d.n.r.	d.n.r.
D7	H11/B	Control 19/B	30768112	1,04	33493056	0,89	1,17
D8	H11/T	Control 19/T	d.n.r.	d.n.r.	d.n.r.	d.n.r.	d.n.r.
D9	H19/B	Control 5/B	23954914	0,81	29291952	0,78	1,04
D10	H19/T	Control 5/T	d.n.r.	d.n.r.	d.n.r.	d.n.r.	d.n.r.
D11	CR2/B/D0	ICU 14/B/D0	30753668	1,04	26753646	0,71	1,46
D12	CR2/B/D7	ICU 14/B/D7	28668626	0,97	28843383	0,77	1,26
D13	CR2/B/D180	ICU 14/B/D180	26063922	0,88	26981280	0,72	1,23
D14	CR2/T/D0	ICU 14/T/D0	d.n.r.	d.n.r.	d.n.r.	d.n.r.	d.n.r.
D15	CR2/T/D7	ICU 14/T/D7	d.n.r.	d.n.r.	d.n.r.	d.n.r.	d.n.r.
D16	CR2/T/D180	ICU 14/T/D180	d.n.r.	d.n.r.	d.n.r.	d.n.r.	d.n.r.
D17	CR17/B/D0	ICU 23/B/D0	27033878	0,91	28709933	0,76	1,20
D18	CR17/B/D7	ICU 23/B/D7	25482896	0,86	28537335	0,76	1,13
D19	CR17/B/D180	ICU 23/B/D180	28621980	0,97	29401155	0,78	1,24
D20	CR17/T/D0	ICU 23/T/D0	d.n.r.	d.n.r.	d.n.r.	d.n.r.	d.n.r.
D21	CR17/T/D7	ICU 23/T/D7	d.n.r.	d.n.r.	d.n.r.	d.n.r.	d.n.r.
D22	CR17/T/D180	ICU 23/T/D180	d.n.r.	d.n.r.	d.n.r.	d.n.r.	d.n.r.
	calibrator		26975550	0,91	39393207	1,05	0,87

DRP1 total (DRP-1 Ser 637 membrane)

	WB ID	Control/Patient ID	protein density	protein normalized to calibrator	GAPDH density	GAPDH normalized to calibrator	protein/GAPDH
GEL 5/ set E		cal. mean	30894397	cal. mean	33459356		
	calibrator		32990426	1,07	32367400	0,97	1,10
E1	H12/B	Control 7/B	21289858	0,69	33789330	1,01	0,68
E2	H12/T	Control 7/T	d.n.r.	d.n.r.	d.n.r.	d.n.r.	d.n.r.
E3	H13/B	Control 1/B	18432200	0,60	32255844	0,96	0,62
E4	H13/T	Control 1/T	d.n.r.	d.n.r.	d.n.r.	d.n.r.	d.n.r.
E5	H18/B	Control 11/B	32361885	1,05	27424188	0,82	1,28
E6	H18/T	Control 11/T	d.n.r.	d.n.r.	d.n.r.	d.n.r.	d.n.r.
E7	CR6/B/D0	ICU 21/B/D0	34403400	1,11	29010256	0,87	1,28
E8	CR6/B/D7	ICU 21/B/D7	27952920	0,90	22604584	0,68	1,34
E9	CR6/T/D0	ICU 21/T/D0	d.n.r.	d.n.r.	d.n.r.	d.n.r.	d.n.r.
E10	CR6/T/D7	ICU 21/T/D7	d.n.r.	d.n.r.	d.n.r.	d.n.r.	d.n.r.
E11	CR14/B/D0	ICU 10/B/D0	34884315	1,13	24246002	0,72	1,56
E12	CR14/B/D7	ICU 10/B/D7	32954085	1,07	26351913	0,79	1,35
E13	CR14/T/D0	ICU 10/T/D0	d.n.r.	d.n.r.	d.n.r.	d.n.r.	d.n.r.
E14	CR14/T/D7	ICU 10/T/D7	d.n.r.	d.n.r.	d.n.r.	d.n.r.	d.n.r.
E15	CR16/B/D0	ICU 16/B/D0	27389385	0,89	29617830	0,89	1,00
E16	CR16/B/D7	ICU 16/B/D7	28536975	0,92	26893768	0,80	1,15
E17	CR16/T/D0	ICU 16/T/D0	d.n.r.	d.n.r.	d.n.r.	d.n.r.	d.n.r.
E18	CR16/T/D7	ICU 16/T/D7	d.n.r.	d.n.r.	d.n.r.	d.n.r.	d.n.r.
E19	CR19/B/D0	ICU 15/B/D0	36932220	1,20	32525266	0,97	1,23
E20	CR19/B/D7	ICU 15/B/D7	37293975	1,21	38332904	1,15	1,05
E21	CR19/T/D0	ICU 15/T/D0	d.n.r.	d.n.r.	d.n.r.	d.n.r.	d.n.r.
E22	CR19/T/D7	ICU 15/T/D7	d.n.r.	d.n.r.	d.n.r.	d.n.r.	d.n.r.
	calibrator		28798368	0,93	34551312	1,03	0,90

DRP1 total (DRP-1 Ser 637 membrane)

	WB ID	Control/Patient ID	protein density	protein normalized to calibrator	GAPDH density	GAPDH normalized to calibrator	protein/GAPDH
GEL 6/ set F		cal. mean	33140997,5		cal. mean	37954347	
	calibrator		34865043	1,05	26550450	0,70	1,50
F1	H9/B	Control 9/B	32431635	0,98	30236175	0,80	1,23
F2	H9/T	Control 9/T	d.n.r.	d.n.r.	d.n.r.	d.n.r.	d.n.r.
F3	H10/B	Control 15/B	39592293	1,19	28345900	0,75	1,60
F4	H10/T	Control 15/T	d.n.r.	d.n.r.	d.n.r.	d.n.r.	d.n.r.
F5	H14/B	Control 20/B	40974030	1,24	27331912	0,72	1,72
F6	H14/T	Control 20/T	d.n.r.	d.n.r.	d.n.r.	d.n.r.	d.n.r.
F7	CR1/B/D0	ICU 1/B/D0	24593760	0,74	26053802	0,69	1,08
F8	CR1/B/D7	ICU 1/B/D7	34650810	1,05	27329985	0,72	1,45
F9	CR1/B/D180	ICU 1/B/D180	31007616	0,94	29696880	0,78	1,20
F10	CR1/T/D0	ICU 1/T/D0	d.n.r.	d.n.r.	d.n.r.	d.n.r.	d.n.r.
F11	CR1/T/D7	ICU 1/T/D7	d.n.r.	d.n.r.	d.n.r.	d.n.r.	d.n.r.
F12	CR1/T/D180	ICU 1/T/D180	d.n.r.	d.n.r.	d.n.r.	d.n.r.	d.n.r.
F13	CR18/B/D0	ICU 18/B/D0	27601008	0,83	31147879	0,82	1,01
F14	CR18/B/D7	ICU 18/B/D7	30641585	0,92	34315678	0,90	1,02
F15	CR18/B/D180	ICU 18/B/D180	36203610	1,09	36800640	0,97	1,13
F16	CR18/T/D0	ICU 18/T/D0	d.n.r.	d.n.r.	d.n.r.	d.n.r.	d.n.r.
F17	CR18/T/D7	ICU 18/T/D7	d.n.r.	d.n.r.	d.n.r.	d.n.r.	d.n.r.
F18	CR18/T/D180	ICU 18/T/D180	d.n.r.	d.n.r.	d.n.r.	d.n.r.	d.n.r.
F19	CR15/B/D0	ICU 12/B/D0	34813471	1,05	45890106	1,21	0,87
F20	CR15/B/D7	ICU 12/B/D7	28861470	0,87	39184088	1,03	0,84
F21	CR15/T/D0	ICU 12/T/D0	d.n.r.	d.n.r.	d.n.r.	d.n.r.	d.n.r.
F22	CR15/T/D7	ICU 12/T/D7	d.n.r.	d.n.r.	d.n.r.	d.n.r.	d.n.r.
	calibrator		31416952	0,95	49358244	1,30	0,73

P-DRP1(Ser 637) vs. DRP1

	WB ID	Control/Patient ID	protein density	protein normalized to calibrator	DRP1 density	DRP1 normalized to calibrator	P-DRP1/DRP1
**GEL 1/ set A**		cal. mean	2992300	cal. mean	20447491		
	calibrator		2451386	0,82	19851813	0,97	0,84
**A1**	**H1/B**	**Control 17/B**	4011660	1,34	16554152	0,81	1,66
**A2**	**H1/T**	**Control 17/T**	d.n.r.	d.n.r.	d.n.r.	d.n.r.	d.n.r.
**A3**	**Sample X/B**	**Sample X/B**	excl.	#####	14963011	0,73	########
**A4**	**Sample X/T**	**Sample X/T**	excl.	#####	6715595	0,33	########
**A5**	**H3/B**	**Control 3/B**	5213318	1,74	17454345	0,85	2,04
**A6**	**H3/T**	**Control 3/T**	d.n.r.	d.n.r.	d.n.r.	d.n.r.	d.n.r.
**A7**	**CR4/B/D0**	**ICU 4/B/D0**	2710412	0,91	19906695	0,97	0,93
**A8**	**CR4/B/D7**	**ICU 4/B/D7**	4764220	1,59	12477344	0,61	2,61
**A9**	**CR4/T/D0**	**ICU 4/T/D0**	d.n.r.	d.n.r.	d.n.r.	d.n.r.	d.n.r.
**A10**	**CR4/T/D7**	**ICU 4/T/D7**	d.n.r.	d.n.r.	d.n.r.	d.n.r.	d.n.r.
**A11**	**CR8/B/D0**	**ICU 11/B/D0**	8360684	2,79	14319855	0,70	3,99
**A12**	**CR8/B/D7**	**ICU 11/B/D7**	8423566	2,82	17135072	0,84	3,36
**A13**	**CR8/T/D0**	**ICU 11/T/D0**	d.n.r.	d.n.r.	d.n.r.	d.n.r.	d.n.r.
**A14**	**CR8/T/D7**	**ICU 11/T/D7**	d.n.r.	d.n.r.	d.n.r.	d.n.r.	d.n.r.
**A15**	**CR10/B/D0**	**ICU 13/B/D0**	1995296	0,67	14442795	0,71	0,94
**A16**	**CR10/B/D7**	**ICU 13/B/D7**	2249308	0,75	17411714	0,85	0,88
**A17**	**CR10/T/D0**	**ICU 13/T/D0**	d.n.r.	d.n.r.	d.n.r.	d.n.r.	d.n.r.
**A18**	**CR10/T/D7**	**ICU 13/T/D7**	d.n.r.	d.n.r.	d.n.r.	d.n.r.	d.n.r.
**A19**	**CR11/B/D0**	**ICU 9/B/D0**	2218304	0,74	17970682	0,88	0,84
**A20**	**CR11/B/D7**	**ICU 9/B/D7**	2166554	0,72	15427454	0,75	0,96
**A21**	**CR11/T/D0**	**ICU 9/T/D0**	d.n.r.	d.n.r.	d.n.r.	d.n.r.	d.n.r.
**A22**	**CR11/T/D7**	**ICU 9/T/D7**	d.n.r.	d.n.r.	d.n.r.	d.n.r.	d.n.r.
	calibrator		3533214	1,18	21043169	1,03	1,15

P-DRP1(Ser 637) vs. DRP1

GEL 2/ set B — protein: cal. mean 3298747,5 — DRP1: cal. mean 19760197

	WB ID	Control/Patient ID	protein density	protein normalized to calibrator	DRP1 density	DRP1 normalized to calibrator	P-DRP1/DRP1
	calibrator		3525975	1,07	19240742	0,97	1,10
B1	H4/B	Control 12/B	5400450	1,64	16654302	0,84	1,94
B2	H4/T	Control 12/T	d.n.r.	d.n.r.	d.n.r.	d.n.r.	d.n.r.
B3	H5/B	Control 6/B	4220055	1,28	13381764	0,68	1,89
B4	H5/T	Control 6/T	d.n.r.	d.n.r.	d.n.r.	d.n.r.	d.n.r.
B5	H6/B	Control 8/B	4553415	1,38	16278416	0,82	1,68
B6	H6/T	Control 8/T	d.n.r.	d.n.r.	d.n.r.	d.n.r.	d.n.r.
B7	Sample Y/B	Sample Y/B	excl.	#####	14014774	0,71	########
B8	Sample Y/T	Sample Y/T	excl.	#####	10927346	0,55	########
B9	Sample Z/B	Sample Z/B	excl.	#####	13455092	0,68	########
B10	Sample Z/T	Sample Z/T	excl.	#####	10346826	0,52	########
B11	CR9/B/D0	ICU 3/B/D0	2910510	0,88	15626890	0,79	1,12
B12	CR9/B/D7	ICU 3/B/D7	4751685	1,44	16916776	0,86	1,68
B13	CR9/T/D0	ICU 3/T/D0	d.n.r.	d.n.r.	d.n.r.	d.n.r.	d.n.r.
B14	CR9/T/D7	ICU 3/T/D7	d.n.r.	d.n.r.	d.n.r.	d.n.r.	d.n.r.
B15	CR12/B/D0	ICU 20/B/D0	5427585	1,65	14959154	0,76	2,17
B16	CR12/B/D7	ICU 20/B/D7	2650590	0,80	17087160	0,86	0,93
B17	CR12/T/D0	ICU 20/T/D0	d.n.r.	d.n.r.	d.n.r.	d.n.r.	d.n.r.
B18	CR12/T/D7	ICU 20/T/D7	d.n.r.	d.n.r.	d.n.r.	d.n.r.	d.n.r.
B19	CR13/B/D0	ICU 22/B/D0	2379375	0,72	15853596	0,80	0,90
B20	CR13/B/D7	ICU 22/B/D7	3458925	1,05	17871920	0,90	1,16
B21	CR13/T/D0	ICU 22/T/D0	d.n.r.	d.n.r.	d.n.r.	d.n.r.	d.n.r.
B22	CR13/T/D7	ICU 22/T/D7	d.n.r.	d.n.r.	d.n.r.	d.n.r.	d.n.r.
	calibrator		3071520	0,93	20279652	1,03	0,91

P-DRP1(Ser 637) vs. DRP1

	WB ID	Control/Patient ID	protein density	protein normalized to calibrator	DRP1 density	DRP1 normalized to calibrator	P-DRP1/DRP1
**GEL 3/ set C**		**cal. mean**	3258810 ***		**cal. mean** 27314950,5		
	calibrator		3258810	1,00	28575180	1,05	0,96
**C1**	**H15/B**	**Control 2/B**	6009696	1,84	28470870	1,04	1,77
**C2**	**H15/T**	**Control 2/T**	d.n.r.	d.n.r.	d.n.r.	d.n.r.	d.n.r.
**C3**	**H16/B**	**Control 14/B**	2742980	0,84	25753024	0,94	0,89
**C4**	**H16/T**	**Control 14/T**	d.n.r.	d.n.r.	d.n.r.	d.n.r.	d.n.r.
**C5**	**H17/B**	**Control 10/B**	1578510	0,48	31206060	1,14	0,42
**C6**	**H17/T**	**Control 10/T**	d.n.r.	d.n.r.	d.n.r.	d.n.r.	d.n.r.
**C7**	**CR3/B/D0**	**ICU 5/B/D0**	2302785	0,71	18451665	0,68	1,05
**C8**	**CR3/B/D7**	**ICU 5/B/D7**	5476365	1,68	18915705	0,69	2,43
**C9**	**CR3/T/D0**	**ICU 5/T/D0**	d.n.r.	d.n.r.	d.n.r.	d.n.r.	d.n.r.
**C10**	**CR3/T/D7**	**ICU 5/T/D7**	d.n.r.	d.n.r.	d.n.r.	d.n.r.	d.n.r.
**C11**	**CR5/B/D0**	**ICU 8/B/D0**	6957300	2,13	21493485	0,79	2,71
**C12**	**CR5/B/D7**	**ICU 8/B/D7**	16564050	5,08	22927095	0,84	6,06
**C13**	**CR5/T/D0**	**ICU 8/T/D0**	d.n.r.	d.n.r.	d.n.r.	d.n.r.	d.n.r.
**C14**	**CR5/T/D7**	**ICU 8/T/D7**	d.n.r.	d.n.r.	d.n.r.	d.n.r.	d.n.r.
**C15**	**CR7/B/D0**	**ICU 6/B/D0**	2771778	0,85	20380635	0,75	1,14
**C16**	**CR7/B/D7**	**ICU 6/B/D7**	1862775	0,57	20218320	0,74	0,77
**C17**	**CD7/T/D0**	**ICU 6/T/D0**	d.n.r.	d.n.r.	d.n.r.	d.n.r.	d.n.r.
**C18**	**CR7/T/D7**	**ICU 6/T/D7**	d.n.r.	d.n.r.	d.n.r.	d.n.r.	d.n.r.
**C19**	**CR20/B/D0**	**ICU 7/B/D0**	1746585	0,54	22083726	0,81	0,66
**C20**	**CR20/B/D7**	**ICU 7/B/D7**	2664585	0,82	20050704	0,73	1,11
**C21**	**CR20/T/D0**	**ICU 7/T/D0**	d.n.r.	d.n.r.	d.n.r.	d.n.r.	d.n.r.
**C22**	**CR20/T/D7**	**ICU 7/T/D7**	d.n.r.	d.n.r.	d.n.r.	d.n.r.	d.n.r.
	calibrator		967920	0,30	26054721	0,95	0,31

P-DRP1(Ser 637) vs. DRP1

	WB ID	Control/Patient ID	protein density	protein normalized to calibrator	DRP1 density	DRP1 normalized to calibrator	P-DRP1/DRP1
GEL 4/ set D		cal. mean	4142352	***	cal. mean 29612937		
	calibrator		4142352	1,00	32250324	1,09	0,92
**D1**	**H2/B**	**Control 4/B**	5240970	1,27	22447264	0,76	1,67
**D2**	**H2/T**	**Control 4/T**	d.n.r.	d.n.r.	d.n.r.	d.n.r.	d.n.r.
**D3**	**H7/B**	**Control 13/B**	4746832	1,15	32323924	1,09	1,05
**D4**	**H7/T**	**Control 13/T**	d.n.r.	d.n.r.	d.n.r.	d.n.r.	d.n.r.
**D5**	**H8/B**	**Control 18/B**	5325075	1,29	36727826	1,24	1,04
**D6**	**H8/T**	**Control 18/T**	d.n.r.	d.n.r.	d.n.r.	d.n.r.	d.n.r.
**D7**	**H11/B**	**Control 19/B**	3935925	0,95	30768112	1,04	0,91
**D8**	**H11/T**	**Control 19/T**	d.n.r.	d.n.r.	d.n.r.	d.n.r.	d.n.r.
**D9**	**H19/B**	**Control 5/B**	3130605	0,76	23954914	0,81	0,93
**D10**	**H19/T**	**Control 5/T**	d.n.r.	d.n.r.	d.n.r.	d.n.r.	d.n.r.
**D11**	**CR2/B/D0**	**ICU 14/B/D0**	4192408	1,01	30753668	1,04	0,97
**D12**	**CR2/B/D7**	**ICU 14/B/D7**	2185290	0,53	28668626	0,97	0,54
**D13**	**CR2/B/D180**	**ICU 14/B/D180**	3134745	0,76	26063922	0,88	0,86
**D14**	**CR2/T/D0**	**ICU 14/T/D0**	d.n.r.	d.n.r.	d.n.r.	d.n.r.	d.n.r.
**D15**	**CR2/T/D7**	**ICU 14/T/D7**	d.n.r.	d.n.r.	d.n.r.	d.n.r.	d.n.r.
**D16**	**CR2/T/D180**	**ICU 14/T/D180**	d.n.r.	d.n.r.	d.n.r.	d.n.r.	d.n.r.
**D17**	**CR17/B/D0**	**ICU 23/B/D0**	2814165	0,68	27033878	0,91	0,74
**D18**	**CR17/B/D7**	**ICU 23/B/D7**	4329810	1,05	25482896	0,86	1,21
**D19**	**CR17/B/D180**	**ICU 23/B/D180**	10378035	2,51	28621980	0,97	2,59
**D20**	**CR17/T/D0**	**ICU 23/T/D0**	d.n.r.	d.n.r.	d.n.r.	d.n.r.	d.n.r.
**D21**	**CR17/T/D7**	**ICU 23/T/D7**	d.n.r.	d.n.r.	d.n.r.	d.n.r.	d.n.r.
**D22**	**CR17/T/D180**	**ICU 23/T/D180**	d.n.r.	d.n.r.	d.n.r.	d.n.r.	d.n.r.
	calibrator		2121936	0,51	26975550	0,91	0,56

P-DRP1(Ser 637) vs. DRP1

	WB ID	Control/Patient ID	protein density	protein normalized to calibrator	DRP1 density	DRP1 normalized to calibrator	P-DRP1/DRP1
**GEL 5/ set E**		**cal. mean**	4893312	**cal. mean**	30894397		
	calibrator		4093680	0,84	32990426	1,07	0,78
**E1**	**H12/B**	**Control 7/B**	4672052	0,95	21289858	0,69	1,39
**E2**	**H12/T**	**Control 7/T**	d.n.r.	d.n.r.	d.n.r.	d.n.r.	d.n.r.
**E3**	**H13/B**	**Control 1/B**	8830252	1,80	18432200	0,60	3,02
**E4**	**H13/T**	**Control 1/T**	d.n.r.	d.n.r.	d.n.r.	d.n.r.	d.n.r.
**E5**	**H18/B**	**Control 11/B**	12166020	2,49	32361885	1,05	2,37
**E6**	**H18/T**	**Control 11/T**	d.n.r.	d.n.r.	d.n.r.	d.n.r.	d.n.r.
**E7**	**CR6/B/D0**	**ICU 21/B/D0**	10031310	2,05	34403400	1,11	1,84
**E8**	**CR6/B/D7**	**ICU 21/B/D7**	13332285	2,72	27952920	0,90	3,01
**E9**	**CR6/T/D0**	**ICU 21/T/D0**	d.n.r.	d.n.r.	d.n.r.	d.n.r.	d.n.r.
**E10**	**CR6/T/D7**	**ICU 21/T/D7**	d.n.r.	d.n.r.	d.n.r.	d.n.r.	d.n.r.
**E11**	**CR14/B/D0**	**ICU 10/B/D0**	13113628	2,68	34884315	1,13	2,37
**E12**	**CR14/B/D7**	**ICU 10/B/D7**	11918880	2,44	32954085	1,07	2,28
**E13**	**CR14/T/D0**	**ICU 10/T/D0**	d.n.r.	d.n.r.	d.n.r.	d.n.r.	d.n.r.
**E14**	**CR14/T/D7**	**ICU 10/T/D7**	d.n.r.	d.n.r.	d.n.r.	d.n.r.	d.n.r.
**E15**	**CR16/B/D0**	**ICU 16/B/D0**	4153874	0,85	27389385	0,89	0,96
**E16**	**CR16/B/D7**	**ICU 16/B/D7**	2510910	0,51	28536975	0,92	0,56
**E17**	**CR16/T/D0**	**ICU 16/T/D0**	d.n.r.	d.n.r.	d.n.r.	d.n.r.	d.n.r.
**E18**	**CR16/T/D7**	**ICU 16/T/D7**	d.n.r.	d.n.r.	d.n.r.	d.n.r.	d.n.r.
**E19**	**CR19/B/D0**	**ICU 15/B/D0**	6654870	1,36	36932220	1,20	1,14
**E20**	**CR19/B/D7**	**ICU 15/B/D7**	5248804	1,07	37293975	1,21	0,89
**E21**	**CR19/T/D0**	**ICU 15/T/D0**	d.n.r.	d.n.r.	d.n.r.	d.n.r.	d.n.r.
**E22**	**CR19/T/D7**	**ICU 15/T/D7**	d.n.r.	d.n.r.	d.n.r.	d.n.r.	d.n.r.
	calibrator		5692944	1,16	28798368	0,93	1,25

P-DRP1(Ser 637) vs. DRP1

	WB ID	Control/Patient ID	protein density	protein normalized to calibrator	DRP1 density	DRP1 normalized to calibrator	P-DRP1/DRP1
GEL 6/ set F		cal. mean	2534847,5	cal. mean	33140997,5		
	calibrator		2912800	1,15	34865043	1,05	1,09
F1	H9/B	Control 9/B	9266985	3,66	32431635	0,98	3,74
F2	H9/T	Control 9/T	d.n.r.	d.n.r.	d.n.r.	d.n.r.	d.n.r.
F3	H10/B	Control 15/B	3815680	1,51	39592293	1,19	1,26
F4	H10/T	Control 15/T	d.n.r.	d.n.r.	d.n.r.	d.n.r.	d.n.r.
F5	H14/B	Control 20/B	6793160	2,68	40974030	1,24	2,17
F6	H14/T	Control 20/T	d.n.r.	d.n.r.	d.n.r.	d.n.r.	d.n.r.
F7	CR1/B/D0	ICU 1/B/D0	4377296	1,73	24593760	0,74	2,33
F8	CR1/B/D7	ICU 1/B/D7	6182944	2,44	34650810	1,05	2,33
F9	CR1/B/D180	ICU 1/B/D180	8239275	3,25	31007616	0,94	3,47
F10	CR1/T/D0	ICU 1/T/D0	d.n.r.	d.n.r.	d.n.r.	d.n.r.	d.n.r.
F11	CR1/T/D7	ICU 1/T/D7	d.n.r.	d.n.r.	d.n.r.	d.n.r.	d.n.r.
F12	CR1/T/D180	ICU 1/T/D180	d.n.r.	d.n.r.	d.n.r.	d.n.r.	d.n.r.
F13	CR18/B/D0	ICU 18/B/D0	9189111	3,63	27601008	0,83	4,35
F14	CR18/B/D7	ICU 18/B/D7	2712380	1,07	30641585	0,92	1,16
F15	CR18/B/D180	ICU 18/B/D180	15312976	6,04	36203610	1,09	5,53
F16	CR18/T/D0	ICU 18/T/D0	d.n.r.	d.n.r.	d.n.r.	d.n.r.	d.n.r.
F17	CR18/T/D7	ICU 18/T/D7	d.n.r.	d.n.r.	d.n.r.	d.n.r.	d.n.r.
F18	CR18/T/D180	ICU 18/T/D180	d.n.r.	d.n.r.	d.n.r.	d.n.r.	d.n.r.
F19	CR15/B/D0	ICU 12/B/D0	8114738	3,20	34813471	1,05	3,05
F20	CR15/B/D7	ICU 12/B/D7	21111413	8,33	28861470	0,87	9,56
F21	CR15/T/D0	ICU 12/T/D0	d.n.r.	d.n.r.	d.n.r.	d.n.r.	d.n.r.
F22	CR15/T/D7	ICU 12/T/D7	d.n.r.	d.n.r.	d.n.r.	d.n.r.	d.n.r.
	calibrator		2156895	0,85	31416952	0,95	0,90

P-DRP1 (Ser616)

	WB ID	Control/Patient ID	protein density	protein normalized to calibrator	GAPDH density	GAPDH normalized to calibrator	protein/GAPDH
GEL 1/ set A		cal. mean	13155333		cal. mean	10427420	
	calibrator		13478092	1,02	11665917	1,12	0,92
A1	H1/B	Control 17/B	1860378	0,14	14751528	1,41	0,10
A2	H1/T	Control 17/T	d.n.r.	d.n.r.	d.n.r.	d.n.r.	d.n.r.
A3	Sample X/B	Sample X/B	excl.	#####	11519366	1,10	########
A4	Sample X/T	Sample X/T	excl.	#####	9972366	0,96	########
A5	H3/B	Control 3/B	5922638	0,45	12429150	1,19	0,38
A6	H3/T	Control 3/T	d.n.r.	d.n.r.	d.n.r.	d.n.r.	d.n.r.
A7	CR4/B/D0	ICU 4/B/D0	1236102	0,09	13267540	1,27	0,07
A8	CR4/B/D7	ICU 4/B/D7	1361094	0,10	14906802	1,43	0,07
A9	CR4/T/D0	ICU 4/T/D0	d.n.r.	d.n.r.	d.n.r.	d.n.r.	d.n.r.
A10	CR4/T/D7	ICU 4/T/D7	d.n.r.	d.n.r.	d.n.r.	d.n.r.	d.n.r.
A11	CR8/B/D0	ICU 11/B/D0	5276788	0,40	12271770	1,18	0,34
A12	CR8/B/D7	ICU 11/B/D7	5430960	0,41	13834497	1,33	0,31
A13	CR8/T/D0	ICU 11/T/D0	d.n.r.	d.n.r.	d.n.r.	d.n.r.	d.n.r.
A14	CR8/T/D7	ICU 11/T/D7	d.n.r.	d.n.r.	d.n.r.	d.n.r.	d.n.r.
A15	CR10/B/D0	ICU 13/B/D0	2116845	0,16	16572059	1,59	0,10
A16	CR10/B/D7	ICU 13/B/D7	3168836	0,24	17226359	1,65	0,15
A17	CR10/T/D0	ICU 13/T/D0	d.n.r.	d.n.r.	d.n.r.	d.n.r.	d.n.r.
A18	CR10/T/D7	ICU 13/T/D7	d.n.r.	d.n.r.	d.n.r.	d.n.r.	d.n.r.
A19	CR11/B/D0	ICU 9/B/D0	1480469	0,11	16502540	1,58	0,07
A20	CR11/B/D7	ICU 9/B/D7	1506204	0,11	16566748	1,59	0,07
A21	CR11/T/D0	ICU 9/T/D0	d.n.r.	d.n.r.	d.n.r.	d.n.r.	d.n.r.
A22	CR11/T/D7	ICU 9/T/D7	d.n.r.	d.n.r.	d.n.r.	d.n.r.	d.n.r.
	calibrator		12832574	0,98	9188923	0,88	1,11

P-DRP1 (Ser616)

	WB ID	Control/Patient ID	protein density	protein normalized to calibrator	GAPDH density	GAPDH normalized to calibrator	protein/GAPDH
GEL 2/ set B		cal. mean	15021821	cal. mean	13620209		
	calibrator		15325498	1,02	15103226	1,11	0,92
**B1**	**H4/B**	**Control 12/B**	2279129	0,15	24508110	1,80	0,08
**B2**	**H4/T**	**Control 12/T**	d.n.r.	d.n.r.	d.n.r.	d.n.r.	d.n.r.
**B3**	**H5/B**	**Control 6/B**	1248660	0,08	20180108	1,48	0,06
**B4**	**H5/T**	**Control 6/T**	d.n.r.	d.n.r.	d.n.r.	d.n.r.	d.n.r.
**B5**	**H6/B**	**Control 8/B**	1361272	0,09	15163650	1,11	0,08
**B6**	**H6/T**	**Control 8/T**	d.n.r.	d.n.r.	d.n.r.	d.n.r.	d.n.r.
**B7**	**Sample Y/B**	**Sample Y/B**	excl.	#####	15430378	1,13	########
**B8**	**Sample Y/T**	**Sample Y/T**	excl.	#####	13459094	0,99	########
**B9**	**Sample Z/B**	**Sample Z/B**	excl.	#####	14559168	1,07	########
**B10**	**Sample Z/T**	**Sample Z/T**	excl.	#####	12338764	0,91	########
**B11**	**CR9/B/D0**	**ICU 3/B/D0**	3198150	0,21	15477344	1,14	0,19
**B12**	**CR9/B/D7**	**ICU 3/B/D7**	4367838	0,29	19214250	1,41	0,21
**B13**	**CR9/T/D0**	**ICU 3/T/D0**	d.n.r.	d.n.r.	d.n.r.	d.n.r.	d.n.r.
**B14**	**CR9/T/D7**	**ICU 3/T/D7**	d.n.r.	d.n.r.	d.n.r.	d.n.r.	d.n.r.
**B15**	**CR12/B/D0**	**ICU 20/B/D0**	2361180	0,16	19783015	1,45	0,11
**B16**	**CR12/B/D7**	**ICU 20/B/D7**	3132554	0,21	20225326	1,48	0,14
**B17**	**CR12/T/D0**	**ICU 20/T/D0**	d.n.r.	d.n.r.	d.n.r.	d.n.r.	d.n.r.
**B18**	**CR12/T/D7**	**ICU 20/T/D7**	d.n.r.	d.n.r.	d.n.r.	d.n.r.	d.n.r.
**B19**	**CR13/B/D0**	**ICU 22/B/D0**	1530225	0,10	16459904	1,21	0,08
**B20**	**CR13/B/D7**	**ICU 22/B/D7**	1226958	0,08	15185244	1,11	0,07
**B21**	**CR13/T/D0**	**ICU 22/T/D0**	d.n.r.	d.n.r.	d.n.r.	d.n.r.	d.n.r.
**B22**	**CR13/T/D7**	**ICU 22/T/D7**	d.n.r.	d.n.r.	d.n.r.	d.n.r.	d.n.r.
	calibrator		14718144	0,98	12137192	0,89	1,10

P-DRP1 (Ser616)

WB ID		Control/Patient ID	protein density	protein normalized to calibrator	GAPDH density	GAPDH normalized to calibrator	protein/GAPDH
GEL 3/ set C		cal. mean	24661704 ***		cal. mean 29009047,5		
	calibrator		24661704	1,00	31268520	1,08	0,93
C1	H15/B	Control 2/B	3797892	0,15	32658660	1,13	0,14
C2	H15/T	Control 2/T	d.n.r.	d.n.r.	d.n.r.	d.n.r.	d.n.r.
C3	H16/B	Control 14/B	1220288	0,05	30500228	1,05	0,05
C4	H16/T	Control 14/T	d.n.r.	d.n.r.	d.n.r.	d.n.r.	d.n.r.
C5	H17/B	Control 10/B	1466572	0,06	26647155	0,92	0,06
C6	H17/T	Control 10/T	d.n.r.	d.n.r.	d.n.r.	d.n.r.	d.n.r.
C7	CR3/B/D0	ICU 5/B/D0	471086	0,02	23008587	0,79	0,02
C8	CR3/B/D7	ICU 5/B/D7	887386	0,04	20825559	0,72	0,05
C9	CR3/T/D0	ICU 5/T/D0	d.n.r.	d.n.r.	d.n.r.	d.n.r.	d.n.r.
C10	CR3/T/D7	ICU 5/T/D7	d.n.r.	d.n.r.	d.n.r.	d.n.r.	d.n.r.
C11	CR5/B/D0	ICU 8/B/D0	1312058	0,05	24818544	0,86	0,06
C12	CR5/B/D7	ICU 8/B/D7	1467354	0,06	21221460	0,73	0,08
C13	CR5/T/D0	ICU 8/T/D0	d.n.r.	d.n.r.	d.n.r.	d.n.r.	d.n.r.
C14	CR5/T/D7	ICU 8/T/D7	d.n.r.	d.n.r.	d.n.r.	d.n.r.	d.n.r.
C15	CR7/B/D0	ICU 6/B/D0	804126	0,03	31349850	1,08	0,03
C16	CR7/B/D7	ICU 6/B/D7	779562	0,03	30200625	1,04	0,03
C17	CD7/T/D0	ICU 6/T/D0	d.n.r.	d.n.r.	d.n.r.	d.n.r.	d.n.r.
C18	CR7/T/D7	ICU 6/T/D7	d.n.r.	d.n.r.	d.n.r.	d.n.r.	d.n.r.
C19	CR20/B/D0	ICU 7/B/D0	2678718	0,11	29807955	1,03	0,11
C20	CR20/B/D7	ICU 7/B/D7	5670060	0,23	32177728	1,11	0,21
C21	CR20/T/D0	ICU 7/T/D0	d.n.r.	d.n.r.	d.n.r.	d.n.r.	d.n.r.
C22	CR20/T/D7	ICU 7/T/D7	d.n.r.	d.n.r.	d.n.r.	d.n.r.	d.n.r.
	calibrator		12151020	0,49	26749575	0,92	0,53

P-DRP1 (Ser616)

	WB ID	Control/Patient ID	protein density	protein normalized to calibrator	GAPDH density	GAPDH normalized to calibrator	protein/GAPDH
**GEL 4/ set D**		**cal. mean**	24102166		**cal. mean** 29637461,5		
	calibrator		25517316	1,06	32687753	1,10	0,96
**D1**	**H2/B**	**Control 4/B**	3355880	0,14	25631280	0,86	0,16
**D2**	**H2/T**	**Control 4/T**	d.n.r.	d.n.r.	d.n.r.	d.n.r.	d.n.r.
**D3**	**H7/B**	**Control 13/B**	2171444	0,09	31143112	1,05	0,09
**D4**	**H7/T**	**Control 13/T**	d.n.r.	d.n.r.	d.n.r.	d.n.r.	d.n.r.
**D5**	**H8/B**	**Control 18/B**	6775340	0,28	25175295	0,85	0,33
**D6**	**H8/T**	**Control 18/T**	d.n.r.	d.n.r.	d.n.r.	d.n.r.	d.n.r.
**D7**	**H11/B**	**Control 19/B**	2748064	0,11	25479814	0,86	0,13
**D8**	**H11/T**	**Control 19/T**	d.n.r.	d.n.r.	d.n.r.	d.n.r.	d.n.r.
**D9**	**H19/B**	**Control 5/B**	1118304	0,05	20932520	0,71	0,07
**D10**	**H19/T**	**Control 5/T**	d.n.r.	d.n.r.	d.n.r.	d.n.r.	d.n.r.
**D11**	**CR2/B/D0**	**ICU 14/B/D0**	2075172	0,09	30241344	1,02	0,08
**D12**	**CR2/B/D7**	**ICU 14/B/D7**	1012176	0,04	25133085	0,85	0,05
**D13**	**CR2/B/D180**	**ICU 14/B/D180**	806300	0,03	24795792	0,84	0,04
**D14**	**CR2/T/D0**	**ICU 14/T/D0**	d.n.r.	d.n.r.	d.n.r.	d.n.r.	d.n.r.
**D15**	**CR2/T/D7**	**ICU 14/T/D7**	d.n.r.	d.n.r.	d.n.r.	d.n.r.	d.n.r.
**D16**	**CR2/T/D180**	**ICU 14/T/D180**	d.n.r.	d.n.r.	d.n.r.	d.n.r.	d.n.r.
**D17**	**CR17/B/D0**	**ICU 23/B/D0**	942656	0,04	26724960	0,90	0,04
**D18**	**CR17/B/D7**	**ICU 23/B/D7**	651024	0,03	26946765	0,91	0,03
**D19**	**CR17/B/D180**	**ICU 23/B/D180**	2268332	0,09	30084089	1,02	0,09
**D20**	**CR17/T/D0**	**ICU 23/T/D0**	d.n.r.	d.n.r.	d.n.r.	d.n.r.	d.n.r.
**D21**	**CR17/T/D7**	**ICU 23/T/D7**	d.n.r.	d.n.r.	d.n.r.	d.n.r.	d.n.r.
**D22**	**CR17/T/D180**	**ICU 23/T/D180**	d.n.r.	d.n.r.	d.n.r.	d.n.r.	d.n.r.
	calibrator		22687016	0,94	26587170	0,90	1,05

P-DRP1 (Ser616)

	WB ID	Control/Patient ID	protein density	protein normalized to calibrator	GAPDH density	GAPDH normalized to calibrator	protein/GAPDH
**GEL 5/ set E**		cal. mean	14855670		cal. mean 10923817,5		
	calibrator		15512445	1,04	12237750	1,12	0,93
**E1**	**H12/B**	**Control 7/B**	1026045	0,07	12498750	1,14	0,06
**E2**	**H12/T**	**Control 7/T**	d.n.r.	d.n.r.	d.n.r.	d.n.r.	d.n.r.
**E3**	**H13/B**	**Control 1/B**	478890	0,03	19614149	1,80	0,02
**E4**	**H13/T**	**Control 1/T**	d.n.r.	d.n.r.	d.n.r.	d.n.r.	d.n.r.
**E5**	**H18/B**	**Control 11/B**	3126420	0,21	17353592	1,59	0,13
**E6**	**H18/T**	**Control 11/T**	d.n.r.	d.n.r.	d.n.r.	d.n.r.	d.n.r.
**E7**	**CR6/B/D0**	**ICU 21/B/D0**	4776120	0,32	16839405	1,54	0,21
**E8**	**CR6/B/D7**	**ICU 21/B/D7**	2857770	0,19	16236244	1,49	0,13
**E9**	**CR6/T/D0**	**ICU 21/T/D0**	d.n.r.	d.n.r.	d.n.r.	d.n.r.	d.n.r.
**E10**	**CR6/T/D7**	**ICU 21/T/D7**	d.n.r.	d.n.r.	d.n.r.	d.n.r.	d.n.r.
**E11**	**CR14/B/D0**	**ICU 10/B/D0**	3490875	0,23	19310702	1,77	0,13
**E12**	**CR14/B/D7**	**ICU 10/B/D7**	3847005	0,26	19300910	1,77	0,15
**E13**	**CR14/T/D0**	**ICU 10/T/D0**	d.n.r.	d.n.r.	d.n.r.	d.n.r.	d.n.r.
**E14**	**CR14/T/D7**	**ICU 10/T/D7**	d.n.r.	d.n.r.	d.n.r.	d.n.r.	d.n.r.
**E15**	**CR16/B/D0**	**ICU 16/B/D0**	3723255	0,25	14058180	1,29	0,19
**E16**	**CR16/B/D7**	**ICU 16/B/D7**	3113955	0,21	13015838	1,19	0,18
**E17**	**CR16/T/D0**	**ICU 16/T/D0**	d.n.r.	d.n.r.	d.n.r.	d.n.r.	d.n.r.
**E18**	**CR16/T/D7**	**ICU 16/T/D7**	d.n.r.	d.n.r.	d.n.r.	d.n.r.	d.n.r.
**E19**	**CR19/B/D0**	**ICU 15/B/D0**	4733730	0,32	15185278	1,39	0,23
**E20**	**CR19/B/D7**	**ICU 15/B/D7**	2046780	0,14	14273055	1,31	0,11
**E21**	**CR19/T/D0**	**ICU 15/T/D0**	d.n.r.	d.n.r.	d.n.r.	d.n.r.	d.n.r.
**E22**	**CR19/T/D7**	**ICU 15/T/D7**	d.n.r.	d.n.r.	d.n.r.	d.n.r.	d.n.r.
	calibrator		14198895	0,96	9609885	0,88	1,09

P-DRP1 (Ser616)

	WB ID	Control/Patient ID	protein density	protein normalized to calibrator	GAPDH density	GAPDH normalized to calibrator	protein/GAPDH
GEL 6/ set F		cal. mean	19134292,5	cal. mean	14481045		
	calibrator		20892240	1,09	11853990	0,82	1,33
F1	H9/B	Control 9/B	2622285	0,14	19641015	1,36	0,10
F2	H9/T	Control 9/T	d.n.r.	d.n.r.	d.n.r.	d.n.r.	d.n.r.
F3	H10/B	Control 15/B	4891275	0,26	20068098	1,39	0,18
F4	H10/T	Control 15/T	d.n.r.	d.n.r.	d.n.r.	d.n.r.	d.n.r.
F5	H14/B	Control 20/B	4027770	0,21	17405802	1,20	0,18
F6	H14/T	Control 20/T	d.n.r.	d.n.r.	d.n.r.	d.n.r.	d.n.r.
F7	CR1/B/D0	ICU 1/B/D0	2574405	0,13	15359580	1,06	0,13
F8	CR1/B/D7	ICU 1/B/D7	5365350	0,28	16966201	1,17	0,24
F9	CR1/B/D180	ICU 1/B/D180	3132225	0,16	16526986	1,14	0,14
F10	CR1/T/D0	ICU 1/T/D0	d.n.r.	d.n.r.	d.n.r.	d.n.r.	d.n.r.
F11	CR1/T/D7	ICU 1/T/D7	d.n.r.	d.n.r.	d.n.r.	d.n.r.	d.n.r.
F12	CR1/T/D180	ICU 1/T/D180	d.n.r.	d.n.r.	d.n.r.	d.n.r.	d.n.r.
F13	CR18/B/D0	ICU 18/B/D0	3284910	0,17	16144528	1,11	0,15
F14	CR18/B/D7	ICU 18/B/D7	5788350	0,30	18203006	1,26	0,24
F15	CR18/B/D180	ICU 18/B/D180	5519340	0,29	20387890	1,41	0,20
F16	CR18/T/D0	ICU 18/T/D0	d.n.r.	d.n.r.	d.n.r.	d.n.r.	d.n.r.
F17	CR18/T/D7	ICU 18/T/D7	d.n.r.	d.n.r.	d.n.r.	d.n.r.	d.n.r.
F18	CR18/T/D180	ICU 18/T/D180	d.n.r.	d.n.r.	d.n.r.	d.n.r.	d.n.r.
F19	CR15/B/D0	ICU 12/B/D0	3096720	0,16	18896984	1,30	0,12
F20	CR15/B/D7	ICU 12/B/D7	2158200	0,11	18737415	1,29	0,09
F21	CR15/T/D0	ICU 12/T/D0	d.n.r.	d.n.r.	d.n.r.	d.n.r.	d.n.r.
F22	CR15/T/D7	ICU 12/T/D7	d.n.r.	d.n.r.	d.n.r.	d.n.r.	d.n.r.
	calibrator		17376345	0,91	17108100	1,18	0,77

DRP1 total (P-DRP1 Ser616 membrane)

	WB ID	Control/Patient ID	protein density	protein normalized to calibrator	GAPDH density	GAPDH normalized to calibrator	protein/GAPDH
**GEL 1/ set A**		**cal. mean**	20490527		**cal. mean** 10427420		
	calibrator		21299080	1,04	11665917	1,12	0,93
**A1**	**H1/B**	**Control 17/B**	20846716	1,02	14751528	1,41	0,72
**A2**	**H1/T**	**Control 17/T**	d.n.r.	d.n.r.	d.n.r.	d.n.r.	d.n.r.
**A3**	**Sample X/B**	**Sample X/B**	excl.	#####	11519366	1,10	########
**A4**	**Sample X/T**	**Sample X/T**	excl.	#####	9972366	0,96	########
**A5**	**H3/B**	**Control 3/B**	18109476	0,88	12429150	1,19	0,74
**A6**	**H3/T**	**Control 3/T**	d.n.r.	d.n.r.	d.n.r.	d.n.r.	d.n.r.
**A7**	**CR4/B/D0**	**ICU 4/B/D0**	17183540	0,84	13267540	1,27	0,66
**A8**	**CR4/B/D7**	**ICU 4/B/D7**	15410384	0,75	14906802	1,43	0,53
**A9**	**CR4/T/D0**	**ICU 4/T/D0**	d.n.r.	d.n.r.	d.n.r.	d.n.r.	d.n.r.
**A10**	**CR4/T/D7**	**ICU 4/T/D7**	d.n.r.	d.n.r.	d.n.r.	d.n.r.	d.n.r.
**A11**	**CR8/B/D0**	**ICU 11/B/D0**	19115756	0,93	12271770	1,18	0,79
**A12**	**CR8/B/D7**	**ICU 11/B/D7**	17281968	0,84	13834497	1,33	0,64
**A13**	**CR8/T/D0**	**ICU 11/T/D0**	d.n.r.	d.n.r.	d.n.r.	d.n.r.	d.n.r.
**A14**	**CR8/T/D7**	**ICU 11/T/D7**	d.n.r.	d.n.r.	d.n.r.	d.n.r.	d.n.r.
**A15**	**CR10/B/D0**	**ICU 13/B/D0**	14008324	0,68	16572059	1,59	0,43
**A16**	**CR10/B/D7**	**ICU 13/B/D7**	18447044	0,90	17226359	1,65	0,54
**A17**	**CR10/T/D0**	**ICU 13/T/D0**	d.n.r.	d.n.r.	d.n.r.	d.n.r.	d.n.r.
**A18**	**CR10/T/D7**	**ICU 13/T/D7**	d.n.r.	d.n.r.	d.n.r.	d.n.r.	d.n.r.
**A19**	**CR11/B/D0**	**ICU 9/B/D0**	18058348	0,88	16502540	1,58	0,56
**A20**	**CR11/B/D7**	**ICU 9/B/D7**	15125308	0,74	16566748	1,59	0,46
**A21**	**CR11/T/D0**	**ICU 9/T/D0**	d.n.r.	d.n.r.	d.n.r.	d.n.r.	d.n.r.
**A22**	**CR11/T/D7**	**ICU 9/T/D7**	d.n.r.	d.n.r.	d.n.r.	d.n.r.	d.n.r.
	calibrator		19681974	0,96	9188923	0,88	1,09

DRP1 total (P-DRP1 Ser616 membrane)

	WB ID	Control/Patient ID	protein density	protein normalized to calibrator	GAPDH density	GAPDH normalized to calibrator	protein/GAPDH
GEL 2/ set B		cal. mean	22053389,5		cal. mean	13620209	
	calibrator		20342028	0,92	15103226	1,11	0,83
B1	H4/B	Control 12/B	21041320	0,95	24508110	1,80	0,53
B2	H4/T	Control 12/T	d.n.r.	d.n.r.	d.n.r.	d.n.r.	d.n.r.
B3	H5/B	Control 6/B	19388310	0,88	20180108	1,48	0,59
B4	H5/T	Control 6/T	d.n.r.	d.n.r.	d.n.r.	d.n.r.	d.n.r.
B5	H6/B	Control 8/B	18330556	0,83	15163650	1,11	0,75
B6	H6/T	Control 8/T	d.n.r.	d.n.r.	d.n.r.	d.n.r.	d.n.r.
B7	Sample Y/B	Sample Y/B	excl.	#####	15430378	1,13	########
B8	Sample Y/T	Sample Y/T	excl.	#####	13459094	0,99	########
B9	Sample Z/B	Sample Z/B	excl.	#####	14559168	1,07	########
B10	Sample Z/T	Sample Z/T	excl.	#####	12338764	0,91	########
B11	CR9/B/D0	ICU 3/B/D0	20546084	0,93	15477344	1,14	0,82
B12	CR9/B/D7	ICU 3/B/D7	21659882	0,98	19214250	1,41	0,70
B13	CR9/T/D0	ICU 3/T/D0	d.n.r.	d.n.r.	d.n.r.	d.n.r.	d.n.r.
B14	CR9/T/D7	ICU 3/T/D7	d.n.r.	d.n.r.	d.n.r.	d.n.r.	d.n.r.
B15	CR12/B/D0	ICU 20/B/D0	20499532	0,93	19783015	1,45	0,64
B16	CR12/B/D7	ICU 20/B/D7	22765354	1,03	20225326	1,48	0,70
B17	CR12/T/D0	ICU 20/T/D0	d.n.r.	d.n.r.	d.n.r.	d.n.r.	d.n.r.
B18	CR12/T/D7	ICU 20/T/D7	d.n.r.	d.n.r.	d.n.r.	d.n.r.	d.n.r.
B19	CR13/B/D0	ICU 22/B/D0	20832075	0,94	16459904	1,21	0,78
B20	CR13/B/D7	ICU 22/B/D7	22226372	1,01	15185244	1,11	0,90
B21	CR13/T/D0	ICU 22/T/D0	d.n.r.	d.n.r.	d.n.r.	d.n.r.	d.n.r.
B22	CR13/T/D7	ICU 22/T/D7	d.n.r.	d.n.r.	d.n.r.	d.n.r.	d.n.r.
	calibrator		23764751	1,08	12137192	0,89	1,21

DRP1 total (P-DRP1 Ser616 membrane)

	WB ID	Control/Patient ID	protein density	protein normalized to calibrator	GAPDH density	GAPDH normalized to calibrator	protein/GAPDH
GEL 3/ set C		cal. mean	33811909		cal. mean	29009047,5	
	calibrator		33888062	1,00	31268520	1,08	0,93
**C1**	**H15/B**	**Control 2/B**	36855246	1,09	32658660	1,13	0,97
**C2**	**H15/T**	**Control 2/T**	d.n.r.	d.n.r.	d.n.r.	d.n.r.	d.n.r.
**C3**	**H16/B**	**Control 14/B**	32162452	0,95	30500228	1,05	0,90
**C4**	**H16/T**	**Control 14/T**	d.n.r.	d.n.r.	d.n.r.	d.n.r.	d.n.r.
**C5**	**H17/B**	**Control 10/B**	37903540	1,12	26647155	0,92	1,22
**C6**	**H17/T**	**Control 10/T**	d.n.r.	d.n.r.	d.n.r.	d.n.r.	d.n.r.
**C7**	**CR3/B/D0**	**ICU 5/B/D0**	19575254	0,58	23008587	0,79	0,73
**C8**	**CR3/B/D7**	**ICU 5/B/D7**	21974338	0,65	20825559	0,72	0,91
**C9**	**CR3/T/D0**	**ICU 5/T/D0**	d.n.r.	d.n.r.	d.n.r.	d.n.r.	d.n.r.
**C10**	**CR3/T/D7**	**ICU 5/T/D7**	d.n.r.	d.n.r.	d.n.r.	d.n.r.	d.n.r.
**C11**	**CR5/B/D0**	**ICU 8/B/D0**	33517302	0,99	24818544	0,86	1,16
**C12**	**CR5/B/D7**	**ICU 8/B/D7**	41777016	1,24	21221460	0,73	1,69
**C13**	**CR5/T/D0**	**ICU 8/T/D0**	d.n.r.	d.n.r.	d.n.r.	d.n.r.	d.n.r.
**C14**	**CR5/T/D7**	**ICU 8/T/D7**	d.n.r.	d.n.r.	d.n.r.	d.n.r.	d.n.r.
**C15**	**CR7/B/D0**	**ICU 6/B/D0**	36343006	1,07	31349850	1,08	0,99
**C16**	**CR7/B/D7**	**ICU 6/B/D7**	30973320	0,92	30200625	1,04	0,88
**C17**	**CD7/T/D0**	**ICU 6/T/D0**	d.n.r.	d.n.r.	d.n.r.	d.n.r.	d.n.r.
**C18**	**CR7/T/D7**	**ICU 6/T/D7**	d.n.r.	d.n.r.	d.n.r.	d.n.r.	d.n.r.
**C19**	**CR20/B/D0**	**ICU 7/B/D0**	41217886	1,22	29807955	1,03	1,19
**C20**	**CR20/B/D7**	**ICU 7/B/D7**	41811378	1,24	32177728	1,11	1,11
**C21**	**CR20/T/D0**	**ICU 7/T/D0**	d.n.r.	d.n.r.	d.n.r.	d.n.r.	d.n.r.
**C22**	**CR20/T/D7**	**ICU 7/T/D7**	d.n.r.	d.n.r.	d.n.r.	d.n.r.	d.n.r.
	calibrator		33735756	1,00	26749575	0,92	1,08

DRP1 total (P-DRP1 Ser616 membrane)

	WB ID	Control/Patient ID	protein density	protein normalized to calibrator	GAPDH density	GAPDH normalized to calibrator	protein/GAPDH
GEL 4/ set D		cal. mean	29376405		cal. mean	29637461,5	
	calibrator		31913742	1,09	32687753	1,10	0,98
D1	H2/B	Control 4/B	32334826	1,10	25631280	0,86	1,27
D2	H2/T	Control 4/T	d.n.r.	d.n.r.	d.n.r.	d.n.r.	d.n.r.
D3	H7/B	Control 13/B	44026324	1,50	31143112	1,05	1,43
D4	H7/T	Control 13/T	d.n.r.	d.n.r.	d.n.r.	d.n.r.	d.n.r.
D5	H8/B	Control 18/B	44171512	1,50	25175295	0,85	1,77
D6	H8/T	Control 18/T	d.n.r.	d.n.r.	d.n.r.	d.n.r.	d.n.r.
D7	H11/B	Control 19/B	39353184	1,34	25479814	0,86	1,56
D8	H11/T	Control 19/T	d.n.r.	d.n.r.	d.n.r.	d.n.r.	d.n.r.
D9	H19/B	Control 5/B	19317984	0,66	20932520	0,71	0,93
D10	H19/T	Control 5/T	d.n.r.	d.n.r.	d.n.r.	d.n.r.	d.n.r.
D11	CR2/B/D0	ICU 14/B/D0	34569920	1,18	30241344	1,02	1,15
D12	CR2/B/D7	ICU 14/B/D7	30520172	1,04	25133085	0,85	1,23
D13	CR2/B/D180	ICU 14/B/D180	29586878	1,01	24795792	0,84	1,20
D14	CR2/T/D0	ICU 14/T/D0	d.n.r.	d.n.r.	d.n.r.	d.n.r.	d.n.r.
D15	CR2/T/D7	ICU 14/T/D7	d.n.r.	d.n.r.	d.n.r.	d.n.r.	d.n.r.
D16	CR2/T/D180	ICU 14/T/D180	d.n.r.	d.n.r.	d.n.r.	d.n.r.	d.n.r.
D17	CR17/B/D0	ICU 23/B/D0	34750194	1,18	26724960	0,90	1,31
D18	CR17/B/D7	ICU 23/B/D7	30497808	1,04	26946765	0,91	1,14
D19	CR17/B/D180	ICU 23/B/D180	39151106	1,33	30084089	1,02	1,31
D20	CR17/T/D0	ICU 23/T/D0	d.n.r.	d.n.r.	d.n.r.	d.n.r.	d.n.r.
D21	CR17/T/D7	ICU 23/T/D7	d.n.r.	d.n.r.	d.n.r.	d.n.r.	d.n.r.
D22	CR17/T/D180	ICU 23/T/D180	d.n.r.	d.n.r.	d.n.r.	d.n.r.	d.n.r.
	calibrator		26839068	0,91	26587170	0,90	1,02

DRP1 total (P-DRP1 Ser616 membrane)

	WB ID	Control/Patient ID	protein density	protein normalized to calibrator	GAPDH density	GAPDH normalized to calibrator	protein/GAPDH
**GEL 5/ set E**		**cal. mean**	23540647,5		**cal. mean** 10923817,5		
	calibrator		25810290	1,10	12237750	1,12	0,98
**E1**	**H12/B**	**Control 7/B**	21310380	0,91	12498750	1,14	0,79
**E2**	**H12/T**	**Control 7/T**	d.n.r.	d.n.r.	d.n.r.	d.n.r.	d.n.r.
**E3**	**H13/B**	**Control 1/B**	17777655	0,76	19614149	1,80	0,42
**E4**	**H13/T**	**Control 1/T**	d.n.r.	d.n.r.	d.n.r.	d.n.r.	d.n.r.
**E5**	**H18/B**	**Control 11/B**	32708205	1,39	17353592	1,59	0,87
**E6**	**H18/T**	**Control 11/T**	d.n.r.	d.n.r.	d.n.r.	d.n.r.	d.n.r.
**E7**	**CR6/B/D0**	**ICU 21/B/D0**	30594600	1,30	16839405	1,54	0,84
**E8**	**CR6/B/D7**	**ICU 21/B/D7**	29838240	1,27	16236244	1,49	0,85
**E9**	**CR6/T/D0**	**ICU 21/T/D0**	d.n.r.	d.n.r.	d.n.r.	d.n.r.	d.n.r.
**E10**	**CR6/T/D7**	**ICU 21/T/D7**	d.n.r.	d.n.r.	d.n.r.	d.n.r.	d.n.r.
**E11**	**CR14/B/D0**	**ICU 10/B/D0**	36919890	1,57	19310702	1,77	0,89
**E12**	**CR14/B/D7**	**ICU 10/B/D7**	40245075	1,71	19300910	1,77	0,97
**E13**	**CR14/T/D0**	**ICU 10/T/D0**	d.n.r.	d.n.r.	d.n.r.	d.n.r.	d.n.r.
**E14**	**CR14/T/D7**	**ICU 10/T/D7**	d.n.r.	d.n.r.	d.n.r.	d.n.r.	d.n.r.
**E15**	**CR16/B/D0**	**ICU 16/B/D0**	39698092	1,69	14058180	1,29	1,31
**E16**	**CR16/B/D7**	**ICU 16/B/D7**	31497255	1,34	13015838	1,19	1,12
**E17**	**CR16/T/D0**	**ICU 16/T/D0**	d.n.r.	d.n.r.	d.n.r.	d.n.r.	d.n.r.
**E18**	**CR16/T/D7**	**ICU 16/T/D7**	d.n.r.	d.n.r.	d.n.r.	d.n.r.	d.n.r.
**E19**	**CR19/B/D0**	**ICU 15/B/D0**	40211595	1,71	15185278	1,39	1,23
**E20**	**CR19/B/D7**	**ICU 15/B/D7**	42000570	1,78	14273055	1,31	1,37
**E21**	**CR19/T/D0**	**ICU 15/T/D0**	d.n.r.	d.n.r.	d.n.r.	d.n.r.	d.n.r.
**E22**	**CR19/T/D7**	**ICU 15/T/D7**	d.n.r.	d.n.r.	d.n.r.	d.n.r.	d.n.r.
	calibrator		21271005	0,90	9609885	0,88	1,03

DRP1 total (P-DRP1 Ser616 membrane)

	WB ID	Control/Patient ID	protein density	protein normalized to calibrator	GAPDH density	GAPDH normalized to calibrator	protein/GAPDH
**GEL 6/ set F**		cal. mean	29816525,5		cal. mean	14481045	
	calibrator		29337650	0,98	11853990	0,82	1,20
**F1**	**H9/B**	**Control 9/B**	30040944	1,01	19641015	1,36	0,74
**F2**	**H9/T**	**Control 9/T**	d.n.r.	d.n.r.	d.n.r.	d.n.r.	d.n.r.
**F3**	**H10/B**	**Control 15/B**	49933506	1,67	20068098	1,39	1,21
**F4**	**H10/T**	**Control 15/T**	d.n.r.	d.n.r.	d.n.r.	d.n.r.	d.n.r.
**F5**	**H14/B**	**Control 20/B**	40714872	1,37	17405802	1,20	1,14
**F6**	**H14/T**	**Control 20/T**	d.n.r.	d.n.r.	d.n.r.	d.n.r.	d.n.r.
**F7**	**CR1/B/D0**	**ICU 1/B/D0**	23795892	0,80	15359580	1,06	0,75
**F8**	**CR1/B/D7**	**ICU 1/B/D7**	33495544	1,12	16966201	1,17	0,96
**F9**	**CR1/B/D180**	**ICU 1/B/D180**	30412808	1,02	16526986	1,14	0,89
**F10**	**CR1/T/D0**	**ICU 1/T/D0**	d.n.r.	d.n.r.	d.n.r.	d.n.r.	d.n.r.
**F11**	**CR1/T/D7**	**ICU 1/T/D7**	d.n.r.	d.n.r.	d.n.r.	d.n.r.	d.n.r.
**F12**	**CR1/T/D180**	**ICU 1/T/D180**	d.n.r.	d.n.r.	d.n.r.	d.n.r.	d.n.r.
**F13**	**CR18/B/D0**	**ICU 18/B/D0**	17583776	0,59	16144528	1,11	0,53
**F14**	**CR18/B/D7**	**ICU 18/B/D7**	21266996	0,71	18203006	1,26	0,57
**F15**	**CR18/B/D180**	**ICU 18/B/D180**	38725146	1,30	20387890	1,41	0,92
**F16**	**CR18/T/D0**	**ICU 18/T/D0**	d.n.r.	d.n.r.	d.n.r.	d.n.r.	d.n.r.
**F17**	**CR18/T/D7**	**ICU 18/T/D7**	d.n.r.	d.n.r.	d.n.r.	d.n.r.	d.n.r.
**F18**	**CR18/T/D180**	**ICU 18/T/D180**	d.n.r.	d.n.r.	d.n.r.	d.n.r.	d.n.r.
**F19**	**CR15/B/D0**	**ICU 12/B/D0**	26311494	0,88	18896984	1,30	0,68
**F20**	**CR15/B/D7**	**ICU 12/B/D7**	22636504	0,76	18737415	1,29	0,59
**F21**	**CR15/T/D0**	**ICU 12/T/D0**	d.n.r.	d.n.r.	d.n.r.	d.n.r.	d.n.r.
**F22**	**CR15/T/D7**	**ICU 12/T/D7**	d.n.r.	d.n.r.	d.n.r.	d.n.r.	d.n.r.
	calibrator		30295401	1,02	17108100	1,18	0,86

P-DRP1 (S616) vs. DRP1

	WB ID	Control/Patient ID	protein density	protein normalized to calibrator	DRP1 density	DRP1 normalized to calibrator	P-DRP1/DRP1
**GEL 1/ set A**		cal. mean	13155333	cal. mean	20490527		
	calibrator		13478092	1,02	21299080	1,04	0,99
**A1**	**H1/B**	**Control 17/B**	1860378	0,14	20846716	1,02	0,14
**A2**	**H1/T**	**Control 17/T**	d.n.r.	d.n.r.	d.n.r.	d.n.r.	d.n.r.
**A3**	**Sample X/B**	**Sample X/B**	excl.	#####	16393960	0,80	########
**A4**	**Sample X/T**	**Sample X/T**	excl.	#####	5640624	0,28	########
**A5**	**H3/B**	**Control 3/B**	5922638	0,45	18109476	0,88	0,51
**A6**	**H3/T**	**Control 3/T**	d.n.r.	d.n.r.	d.n.r.	d.n.r.	d.n.r.
**A7**	**CR4/B/D0**	**ICU 4/B/D0**	1236102	0,09	17183540	0,84	0,11
**A8**	**CR4/B/D7**	**ICU 4/B/D7**	1361094	0,10	15410384	0,75	0,14
**A9**	**CR4/T/D0**	**ICU 4/T/D0**	d.n.r.	d.n.r.	d.n.r.	d.n.r.	d.n.r.
**A10**	**CR4/T/D7**	**ICU 4/T/D7**	d.n.r.	d.n.r.	d.n.r.	d.n.r.	d.n.r.
**A11**	**CR8/B/D0**	**ICU 11/B/D0**	5276788	0,40	19115756	0,93	0,43
**A12**	**CR8/B/D7**	**ICU 11/B/D7**	5430960	0,41	17281968	0,84	0,49
**A13**	**CR8/T/D0**	**ICU 11/T/D0**	d.n.r.	d.n.r.	d.n.r.	d.n.r.	d.n.r.
**A14**	**CR8/T/D7**	**ICU 11/T/D7**	d.n.r.	d.n.r.	d.n.r.	d.n.r.	d.n.r.
**A15**	**CR10/B/D0**	**ICU 13/B/D0**	2116845	0,16	14008324	0,68	0,24
**A16**	**CR10/B/D7**	**ICU 13/B/D7**	3168836	0,24	18447044	0,90	0,27
**A17**	**CR10/T/D0**	**ICU 13/T/D0**	d.n.r.	d.n.r.	d.n.r.	d.n.r.	d.n.r.
**A18**	**CR10/T/D7**	**ICU 13/T/D7**	d.n.r.	d.n.r.	d.n.r.	d.n.r.	d.n.r.
**A19**	**CR11/B/D0**	**ICU 9/B/D0**	1480469	0,11	18058348	0,88	0,13
**A20**	**CR11/B/D7**	**ICU 9/B/D7**	1506204	0,11	15125308	0,74	0,16
**A21**	**CR11/T/D0**	**ICU 9/T/D0**	d.n.r.	d.n.r.	d.n.r.	d.n.r.	d.n.r.
**A22**	**CR11/T/D7**	**ICU 9/T/D7**	d.n.r.	d.n.r.	d.n.r.	d.n.r.	d.n.r.
	calibrator		12832574	0,98	19681974	0,96	1,02

P-DRP1 (S616) vs. DRP1

	WB ID	Control/Patient ID	protein density	protein normalized to calibrator	DRP1 density	DRP1 normalized to calibrator	P-DRP1/DRP1
GEL 2/ set B		cal. mean	15021821	cal. mean	22053389,5		
	calibrator		15325498	1,02	20342028	0,92	1,11
**B1**	**H4/B**	**Control 12/B**	2279129	0,15	21041320	0,95	0,16
**B2**	**H4/T**	**Control 12/T**	d.n.r.	d.n.r.	d.n.r.	d.n.r.	d.n.r.
**B3**	**H5/B**	**Control 6/B**	1248660	0,08	19388310	0,88	0,09
**B4**	**H5/T**	**Control 6/T**	d.n.r.	d.n.r.	d.n.r.	d.n.r.	d.n.r.
**B5**	**H6/B**	**Control 8/B**	1361272	0,09	18330556	0,83	0,11
**B6**	**H6/T**	**Control 8/T**	d.n.r.	d.n.r.	d.n.r.	d.n.r.	d.n.r.
**B7**	**Sample Y/B**	**Sample Y/B**	excl.	#####	18612520	0,84	########
**B8**	**Sample Y/T**	**Sample Y/T**	excl.	#####	13257016	0,60	########
**B9**	**Sample Z/B**	**Sample Z/B**	excl.	#####	19560120	0,89	########
**B10**	**Sample Z/T**	**Sample Z/T**	excl.	#####	15995350	0,73	########
**B11**	**CR9/B/D0**	**ICU 3/B/D0**	3198150	0,21	20546084	0,93	0,23
**B12**	**CR9/B/D7**	**ICU 3/B/D7**	4367838	0,29	21659882	0,98	0,30
**B13**	**CR9/T/D0**	**ICU 3/T/D0**	d.n.r.	d.n.r.	d.n.r.	d.n.r.	d.n.r.
**B14**	**CR9/T/D7**	**ICU 3/T/D7**	d.n.r.	d.n.r.	d.n.r.	d.n.r.	d.n.r.
**B15**	**CR12/B/D0**	**ICU 20/B/D0**	2361180	0,16	20499532	0,93	0,17
**B16**	**CR12/B/D7**	**ICU 20/B/D7**	3132554	0,21	22765354	1,03	0,20
**B17**	**CR12/T/D0**	**ICU 20/T/D0**	d.n.r.	d.n.r.	d.n.r.	d.n.r.	d.n.r.
**B18**	**CR12/T/D7**	**ICU 20/T/D7**	d.n.r.	d.n.r.	d.n.r.	d.n.r.	d.n.r.
**B19**	**CR13/B/D0**	**ICU 22/B/D0**	1530225	0,10	20832075	0,94	0,11
**B20**	**CR13/B/D7**	**ICU 22/B/D7**	1226958	0,08	22226372	1,01	0,08
**B21**	**CR13/T/D0**	**ICU 22/T/D0**	d.n.r.	d.n.r.	d.n.r.	d.n.r.	d.n.r.
**B22**	**CR13/T/D7**	**ICU 22/T/D7**	d.n.r.	d.n.r.	d.n.r.	d.n.r.	d.n.r.
	calibrator		14718144	0,98	23764751	1,08	0,91

P-DRP1 (S616) vs. DRP1

	WB ID	Control/Patient ID	protein density	protein normalized to calibrator	DRP1 density	DRP1 normalized to calibrator	P-DRP1/DRP1
GEL 3/ set C		cal. mean	24661704 ***		cal. mean 33811909		
	calibrator		24661704	1,00	33888062	1,00	1,00
**C1**	**H15/B**	**Control 2/B**	3797892	0,15	36855246	1,09	0,14
**C2**	**H15/T**	**Control 2/T**	d.n.r.	d.n.r.	d.n.r.	d.n.r.	d.n.r.
**C3**	**H16/B**	**Control 14/B**	1220288	0,05	32162452	0,95	0,05
**C4**	**H16/T**	**Control 14/T**	d.n.r.	d.n.r.	d.n.r.	d.n.r.	d.n.r.
**C5**	**H17/B**	**Control 10/B**	1466572	0,06	37903540	1,12	0,05
**C6**	**H17/T**	**Control 10/T**	d.n.r.	d.n.r.	d.n.r.	d.n.r.	d.n.r.
**C7**	**CR3/B/D0**	**ICU 5/B/D0**	471086	0,02	19575254	0,58	0,03
**C8**	**CR3/B/D7**	**ICU 5/B/D7**	887386	0,04	21974338	0,65	0,06
**C9**	**CR3/T/D0**	**ICU 5/T/D0**	d.n.r.	d.n.r.	d.n.r.	d.n.r.	d.n.r.
**C10**	**CR3/T/D7**	**ICU 5/T/D7**	d.n.r.	d.n.r.	d.n.r.	d.n.r.	d.n.r.
**C11**	**CR5/B/D0**	**ICU 8/B/D0**	1312058	0,05	33517302	0,99	0,05
**C12**	**CR5/B/D7**	**ICU 8/B/D7**	1467354	0,06	41777016	1,24	0,05
**C13**	**CR5/T/D0**	**ICU 8/T/D0**	d.n.r.	d.n.r.	d.n.r.	d.n.r.	d.n.r.
**C14**	**CR5/T/D7**	**ICU 8/T/D7**	d.n.r.	d.n.r.	d.n.r.	d.n.r.	d.n.r.
**C15**	**CR7/B/D0**	**ICU 6/B/D0**	804126	0,03	36343006	1,07	0,03
**C16**	**CR7/B/D7**	**ICU 6/B/D7**	779562	0,03	30973320	0,92	0,03
**C17**	**CD7/T/D0**	**ICU 6/T/D0**	d.n.r.	d.n.r.	d.n.r.	d.n.r.	d.n.r.
**C18**	**CR7/T/D7**	**ICU 6/T/D7**	d.n.r.	d.n.r.	d.n.r.	d.n.r.	d.n.r.
**C19**	**CR20/B/D0**	**ICU 7/B/D0**	2678718	0,11	41217886	1,22	0,09
**C20**	**CR20/B/D7**	**ICU 7/B/D7**	5670060	0,23	41811378	1,24	0,19
**C21**	**CR20/T/D0**	**ICU 7/T/D0**	d.n.r.	d.n.r.	d.n.r.	d.n.r.	d.n.r.
**C22**	**CR20/T/D7**	**ICU 7/T/D7**	d.n.r.	d.n.r.	d.n.r.	d.n.r.	d.n.r.
	calibrator		12151020	0,49	33735756	1,00	0,49

P-DRP1 (S616) vs. DRP1

	WB ID	Control/Patient ID	protein density	protein normalized to calibrator	DRP1 density	DRP1 normalized to calibrator	P-DRP1/DRP1
GEL 4/ set D		cal. mean	24102166	cal. mean	29637461,5		
	calibrator		25517316	1,06	32687753	1,10	0,96
D1	H2/B	Control 4/B	3355880	0,14	25631280	0,86	0,16
D2	H2/T	Control 4/T	d.n.r.	d.n.r.	d.n.r.	d.n.r.	d.n.r.
D3	H7/B	Control 13/B	2171444	0,09	31143112	1,05	0,09
D4	H7/T	Control 13/T	d.n.r.	d.n.r.	d.n.r.	d.n.r.	d.n.r.
D5	H8/B	Control 18/B	6775340	0,28	25175295	0,85	0,33
D6	H8/T	Control 18/T	d.n.r.	d.n.r.	d.n.r.	d.n.r.	d.n.r.
D7	H11/B	Control 19/B	2748064	0,11	25479814	0,86	0,13
D8	H11/T	Control 19/T	d.n.r.	d.n.r.	d.n.r.	d.n.r.	d.n.r.
D9	H19/B	Control 5/B	1118304	0,05	20932520	0,71	0,07
D10	H19/T	Control 5/T	d.n.r.	d.n.r.	d.n.r.	d.n.r.	d.n.r.
D11	CR2/B/D0	ICU 14/B/D0	2075172	0,09	30241344	1,02	0,08
D12	CR2/B/D7	ICU 14/B/D7	1012176	0,04	25133085	0,85	0,05
D13	CR2/B/D180	ICU 14/B/D180	806300	0,03	24795792	0,84	0,04
D14	CR2/T/D0	ICU 14/T/D0	d.n.r.	d.n.r.	d.n.r.	d.n.r.	d.n.r.
D15	CR2/T/D7	ICU 14/T/D7	d.n.r.	d.n.r.	d.n.r.	d.n.r.	d.n.r.
D16	CR2/T/D180	ICU 14/T/D180	d.n.r.	d.n.r.	d.n.r.	d.n.r.	d.n.r.
D17	CR17/B/D0	ICU 23/B/D0	942656	0,04	26724960	0,90	0,04
D18	CR17/B/D7	ICU 23/B/D7	651024	0,03	26946765	0,91	0,03
D19	CR17/B/D180	ICU 23/B/D180	2268332	0,09	30084089	1,02	0,09
D20	CR17/T/D0	ICU 23/T/D0	d.n.r.	d.n.r.	d.n.r.	d.n.r.	d.n.r.
D21	CR17/T/D7	ICU 23/T/D7	d.n.r.	d.n.r.	d.n.r.	d.n.r.	d.n.r.
D22	CR17/T/D180	ICU 23/T/D180	d.n.r.	d.n.r.	d.n.r.	d.n.r.	d.n.r.
	calibrator		22687016	0,94	26587170	0,90	1,05

P-DRP1 (S616) vs. DRP1

	WB ID	Control/Patient ID	protein density	protein normalized to calibrator	DRP1 density	DRP1 normalized to calibrator	P-DRP1/DRP1
GEL 5/ set E		cal. mean	14855670		cal. mean 23540647,5		
	calibrator		15512445	1,04	25810290	1,10	0,95
**E1**	**H12/B**	**Control 7/B**	1026045	0,07	21310380	0,91	0,08
**E2**	**H12/T**	**Control 7/T**	d.n.r.	d.n.r.	d.n.r.	d.n.r.	d.n.r.
**E3**	**H13/B**	**Control 1/B**	478890	0,03	17777655	0,76	0,04
**E4**	**H13/T**	**Control 1/T**	d.n.r.	d.n.r.	d.n.r.	d.n.r.	d.n.r.
**E5**	**H18/B**	**Control 11/B**	3126420	0,21	32708205	1,39	0,15
**E6**	**H18/T**	**Control 11/T**	d.n.r.	d.n.r.	d.n.r.	d.n.r.	d.n.r.
**E7**	**CR6/B/D0**	**ICU 21/B/D0**	4776120	0,32	30594600	1,30	0,25
**E8**	**CR6/B/D7**	**ICU 21/B/D7**	2857770	0,19	29838240	1,27	0,15
**E9**	**CR6/T/D0**	**ICU 21/T/D0**	d.n.r.	d.n.r.	d.n.r.	d.n.r.	d.n.r.
**E10**	**CR6/T/D7**	**ICU 21/T/D7**	d.n.r.	d.n.r.	d.n.r.	d.n.r.	d.n.r.
**E11**	**CR14/B/D0**	**ICU 10/B/D0**	3490875	0,23	36919890	1,57	0,15
**E12**	**CR14/B/D7**	**ICU 10/B/D7**	3847005	0,26	40245075	1,71	0,15
**E13**	**CR14/T/D0**	**ICU 10/T/D0**	d.n.r.	d.n.r.	d.n.r.	d.n.r.	d.n.r.
**E14**	**CR14/T/D7**	**ICU 10/T/D7**	d.n.r.	d.n.r.	d.n.r.	d.n.r.	d.n.r.
**E15**	**CR16/B/D0**	**ICU 16/B/D0**	3723255	0,25	39698092	1,69	0,15
**E16**	**CR16/B/D7**	**ICU 16/B/D7**	3113955	0,21	31497255	1,34	0,16
**E17**	**CR16/T/D0**	**ICU 16/T/D0**	d.n.r.	d.n.r.	d.n.r.	d.n.r.	d.n.r.
**E18**	**CR16/T/D7**	**ICU 16/T/D7**	d.n.r.	d.n.r.	d.n.r.	d.n.r.	d.n.r.
**E19**	**CR19/B/D0**	**ICU 15/B/D0**	4733730	0,32	40211595	1,71	0,19
**E20**	**CR19/B/D7**	**ICU 15/B/D7**	2046780	0,14	42000570	1,78	0,08
**E21**	**CR19/T/D0**	**ICU 15/T/D0**	d.n.r.	d.n.r.	d.n.r.	d.n.r.	d.n.r.
**E22**	**CR19/T/D7**	**ICU 15/T/D7**	d.n.r.	d.n.r.	d.n.r.	d.n.r.	d.n.r.
	calibrator		14198895	0,96	21271005	0,90	1,06

P-DRP1 (S616) vs. DRP1

	WB ID	Control/Patient ID	protein density	protein normalized to calibrator	DRP1 density	DRP1 normalized to calibrator	P-DRP1/DRP1
GEL 6/ set F		cal. mean	19134292,5		cal. mean	29816525,5	
	calibrator		20892240	1,09	29337650	0,98	1,11
F1	H9/B	Control 9/B	2622285	0,14	30040944	1,01	0,14
F2	H9/T	Control 9/T	d.n.r.	d.n.r.	d.n.r.	d.n.r.	d.n.r.
F3	H10/B	Control 15/B	4891275	0,26	49933506	1,67	0,15
F4	H10/T	Control 15/T	d.n.r.	d.n.r.	d.n.r.	d.n.r.	d.n.r.
F5	H14/B	Control 20/B	4027770	0,21	40714872	1,37	0,15
F6	H14/T	Control 20/T	d.n.r.	d.n.r.	d.n.r.	d.n.r.	d.n.r.
F7	CR1/B/D0	ICU 1/B/D0	2574405	0,13	23795892	0,80	0,17
F8	CR1/B/D7	ICU 1/B/D7	5365350	0,28	33495544	1,12	0,25
F9	CR1/B/D180	ICU 1/B/D180	3132225	0,16	30412808	1,02	0,16
F10	CR1/T/D0	ICU 1/T/D0	d.n.r.	d.n.r.	d.n.r.	d.n.r.	d.n.r.
F11	CR1/T/D7	ICU 1/T/D7	d.n.r.	d.n.r.	d.n.r.	d.n.r.	d.n.r.
F12	CR1/T/D180	ICU 1/T/D180	d.n.r.	d.n.r.	d.n.r.	d.n.r.	d.n.r.
F13	CR18/B/D0	ICU 18/B/D0	3284910	0,17	17583776	0,59	0,29
F14	CR18/B/D7	ICU 18/B/D7	5788350	0,30	21266996	0,71	0,42
F15	CR18/B/D180	ICU 18/B/D180	5519340	0,29	38725146	1,30	0,22
F16	CR18/T/D0	ICU 18/T/D0	d.n.r.	d.n.r.	d.n.r.	d.n.r.	d.n.r.
F17	CR18/T/D7	ICU 18/T/D7	d.n.r.	d.n.r.	d.n.r.	d.n.r.	d.n.r.
F18	CR18/T/D180	ICU 18/T/D180	d.n.r.	d.n.r.	d.n.r.	d.n.r.	d.n.r.
F19	CR15/B/D0	ICU 12/B/D0	3096720	0,16	26311494	0,88	0,18
F20	CR15/B/D7	ICU 12/B/D7	2158200	0,11	22636504	0,76	0,15
F21	CR15/T/D0	ICU 12/T/D0	d.n.r.	d.n.r.	d.n.r.	d.n.r.	d.n.r.
F22	CR15/T/D7	ICU 12/T/D7	d.n.r.	d.n.r.	d.n.r.	d.n.r.	d.n.r.
	calibrator		17376345	0,91	30295401	1,02	0,89

OPA1-L (upper band)

	WB ID	Control/Patient ID	protein density	protein normalized to calibrator	GAPDH density	GAPDH normalized to calibrator	protein/GAPDH
GEL 1/ set A		cal. mean	6214612,5		cal. mean 41570172		
	calibrator		7497630	1,21	42065400	1,01	1,19
A1	H1/B	Control 17/B	7544340	1,21	42967628	1,03	1,17
A2	H1/T	Control 17/T	d.n.r.	d.n.r.	d.n.r.	d.n.r.	d.n.r.
A3	Sample X/B	Sample X/B	excl.	#####	36839820	0,89	########
A4	Sample X/T	Sample X/T	excl.	#####	32757912	0,79	########
A5	H3/B	Control 3/B	7926660	1,28	35733870	0,86	1,48
A6	H3/T	Control 3/T	d.n.r.	d.n.r.	d.n.r.	d.n.r.	d.n.r.
A7	CR4/B/D0	ICU 4/B/D0	5397570	0,87	28368765	0,68	1,27
A8	CR4/B/D7	ICU 4/B/D7	5267475	0,85	30026880	0,72	1,17
A9	CR4/T/D0	ICU 4/T/D0	d.n.r.	d.n.r.	d.n.r.	d.n.r.	d.n.r.
A10	CR4/T/D7	ICU 4/T/D7	d.n.r.	d.n.r.	d.n.r.	d.n.r.	d.n.r.
A11	CR8/B/D0	ICU 11/B/D0	5111010	0,82	26405840	0,64	1,29
A12	CR8/B/D7	ICU 11/B/D7	5631615	0,91	28186268	0,68	1,34
A13	CR8/T/D0	ICU 11/T/D0	d.n.r.	d.n.r.	d.n.r.	d.n.r.	d.n.r.
A14	CR8/T/D7	ICU 11/T/D7	d.n.r.	d.n.r.	d.n.r.	d.n.r.	d.n.r.
A15	CR10/B/D0	ICU 13/B/D0	5443843	0,88	32125544	0,77	1,13
A16	CR10/B/D7	ICU 13/B/D7	5619645	0,90	37040626	0,89	1,01
A17	CR10/T/D0	ICU 13/T/D0	d.n.r.	d.n.r.	d.n.r.	d.n.r.	d.n.r.
A18	CR10/T/D7	ICU 13/T/D7	d.n.r.	d.n.r.	d.n.r.	d.n.r.	d.n.r.
A19	CR11/B/D0	ICU 9/B/D0	5215408	0,84	34330306	0,83	1,02
A20	CR11/B/D7	ICU 9/B/D7	6976578	1,12	33386188	0,80	1,40
A21	CR11/T/D0	ICU 9/T/D0	d.n.r.	d.n.r.	d.n.r.	d.n.r.	d.n.r.
A22	CR11/T/D7	ICU 9/T/D7	d.n.r.	d.n.r.	d.n.r.	d.n.r.	d.n.r.
	calibrator		4931595	0,79	41074944	0,99	0,80

OPA1-L (upper band)

	WB ID	Control/Patient ID	protein density	protein normalized to calibrator	GAPDH density	GAPDH normalized to calibrator	protein/GAPDH
GEL 2/ set B		cal. mean	6667057	cal. mean	39443078,5		
	calibrator		6068835	0,91	31672407	0,80	1,13
B1	H4/B	Control 12/B	5838840	0,88	34761556	0,88	0,99
B2	H4/T	Control 12/T	d.n.r.	d.n.r.	d.n.r.	d.n.r.	d.n.r.
B3	H5/B	Control 6/B	5453280	0,82	31297500	0,79	1,03
B4	H5/T	Control 6/T	d.n.r.	d.n.r.	d.n.r.	d.n.r.	d.n.r.
B5	H6/B	Control 8/B	5134680	0,77	26830935	0,68	1,13
B6	H6/T	Control 8/T	d.n.r.	d.n.r.	d.n.r.	d.n.r.	d.n.r.
B7	Sample Y/B	Sample Y/B	excl.	#####	25290990	0,64	########
B8	Sample Y/T	Sample Y/T	excl.	#####	23090160	0,59	########
B9	Sample Z/B	Sample Z/B	excl.	#####	25183170	0,64	########
B10	Sample Z/T	Sample Z/T	excl.	#####	22048708	0,56	########
B11	CR9/B/D0	ICU 3/B/D0	3499245	0,52	28875120	0,73	0,72
B12	CR9/B/D7	ICU 3/B/D7	3088710	0,46	32768164	0,83	0,56
B13	CR9/T/D0	ICU 3/T/D0	d.n.r.	d.n.r.	d.n.r.	d.n.r.	d.n.r.
B14	CR9/T/D7	ICU 3/T/D7	d.n.r.	d.n.r.	d.n.r.	d.n.r.	d.n.r.
B15	CR12/B/D0	ICU 20/B/D0	3439484	0,52	32436270	0,82	0,63
B16	CR12/B/D7	ICU 20/B/D7	4561744	0,68	33377085	0,85	0,81
B17	CR12/T/D0	ICU 20/T/D0	d.n.r.	d.n.r.	d.n.r.	d.n.r.	d.n.r.
B18	CR12/T/D7	ICU 20/T/D7	d.n.r.	d.n.r.	d.n.r.	d.n.r.	d.n.r.
B19	CR13/B/D0	ICU 22/B/D0	5225616	0,78	35632337	0,90	0,87
B20	CR13/B/D7	ICU 22/B/D7	5349544	0,80	35268420	0,89	0,90
B21	CR13/T/D0	ICU 22/T/D0	d.n.r.	d.n.r.	d.n.r.	d.n.r.	d.n.r.
B22	CR13/T/D7	ICU 22/T/D7	d.n.r.	d.n.r.	d.n.r.	d.n.r.	d.n.r.
	calibrator		7265279	1,09	47213750	1,20	0,91

OPA1-L (upper band)

	WB ID	Control/Patient ID	protein density	protein normalized to calibrator	GAPDH density	GAPDH normalized to calibrator	protein/GAPDH
**GEL 3/ set C**		cal. mean	12017075		cal. mean	22029750	
	calibrator		11766732	0,98	20662800	0,94	1,04
**C1**	**H15/B**	**Control 2/B**	11093084	0,92	20219460	0,92	1,01
**C2**	**H15/T**	**Control 2/T**	d.n.r.	d.n.r.	d.n.r.	d.n.r.	d.n.r.
**C3**	**H16/B**	**Control 14/B**	11870276	0,99	15286365	0,69	1,42
**C4**	**H16/T**	**Control 14/T**	d.n.r.	d.n.r.	d.n.r.	d.n.r.	d.n.r.
**C5**	**H17/B**	**Control 10/B**	11324460	0,94	14141864	0,64	1,47
**C6**	**H17/T**	**Control 10/T**	d.n.r.	d.n.r.	d.n.r.	d.n.r.	d.n.r.
**C7**	**CR3/B/D0**	**ICU 5/B/D0**	7537652	0,63	14270928	0,65	0,97
**C8**	**CR3/B/D7**	**ICU 5/B/D7**	9888942	0,82	13239864	0,60	1,37
**C9**	**CR3/T/D0**	**ICU 5/T/D0**	d.n.r.	d.n.r.	d.n.r.	d.n.r.	d.n.r.
**C10**	**CR3/T/D7**	**ICU 5/T/D7**	d.n.r.	d.n.r.	d.n.r.	d.n.r.	d.n.r.
**C11**	**CR5/B/D0**	**ICU 8/B/D0**	12336970	1,03	18034032	0,82	1,25
**C12**	**CR5/B/D7**	**ICU 8/B/D7**	10709352	0,89	15358512	0,70	1,28
**C13**	**CR5/T/D0**	**ICU 8/T/D0**	d.n.r.	d.n.r.	d.n.r.	d.n.r.	d.n.r.
**C14**	**CR5/T/D7**	**ICU 8/T/D7**	d.n.r.	d.n.r.	d.n.r.	d.n.r.	d.n.r.
**C15**	**CR7/B/D0**	**ICU 6/B/D0**	12870968	1,07	17619684	0,80	1,34
**C16**	**CR7/B/D7**	**ICU 6/B/D7**	10077036	0,84	17926524	0,81	1,03
**C17**	**CD7/T/D0**	**ICU 6/T/D0**	d.n.r.	d.n.r.	d.n.r.	d.n.r.	d.n.r.
**C18**	**CR7/T/D7**	**ICU 6/T/D7**	d.n.r.	d.n.r.	d.n.r.	d.n.r.	d.n.r.
**C19**	**CR20/B/D0**	**ICU 7/B/D0**	12286003	1,02	14741100	0,67	1,53
**C20**	**CR20/B/D7**	**ICU 7/B/D7**	14452924	1,20	15625234	0,71	1,70
**C21**	**CR20/T/D0**	**ICU 7/T/D0**	d.n.r.	d.n.r.	d.n.r.	d.n.r.	d.n.r.
**C22**	**CR20/T/D7**	**ICU 7/T/D7**	d.n.r.	d.n.r.	d.n.r.	d.n.r.	d.n.r.
	calibrator		12267418	1,02	23396700	1,06	0,96

OPA1-L (upper band)

	WB ID	Control/Patient ID	protein density	protein normalized to calibrator	GAPDH density	GAPDH normalized to calibrator	protein/GAPDH
**GEL 4/ set D**		cal. mean	9487102,5		cal. mean	27123350	
	calibrator		10049400	1,06	21501050	0,79	1,34
**D1**	**H2/B**	**Control 4/B**	12756555	1,34	15463789	0,57	2,36
**D2**	**H2/T**	**Control 4/T**	d.n.r.	d.n.r.	d.n.r.	d.n.r.	d.n.r.
**D3**	**H7/B**	**Control 13/B**	5276070	0,56	15370470	0,57	0,98
**D4**	**H7/T**	**Control 13/T**	d.n.r.	d.n.r.	d.n.r.	d.n.r.	d.n.r.
**D5**	**H8/B**	**Control 18/B**	4978980	0,52	13315368	0,49	1,07
**D6**	**H8/T**	**Control 18/T**	d.n.r.	d.n.r.	d.n.r.	d.n.r.	d.n.r.
**D7**	**H11/B**	**Control 19/B**	5876325	0,62	15176872	0,56	1,11
**D8**	**H11/T**	**Control 19/T**	d.n.r.	d.n.r.	d.n.r.	d.n.r.	d.n.r.
**D9**	**H19/B**	**Control 5/B**	7436700	0,78	12146022	0,45	1,75
**D10**	**H19/T**	**Control 5/T**	d.n.r.	d.n.r.	d.n.r.	d.n.r.	d.n.r.
**D11**	**CR2/B/D0**	**ICU 14/B/D0**	8344395	0,88	13352832	0,49	1,79
**D12**	**CR2/B/D7**	**ICU 14/B/D7**	7844400	0,83	14137728	0,52	1,59
**D13**	**CR2/B/D180**	**ICU 14/B/D180**	8646750	0,91	11800978	0,44	2,09
**D14**	**CR2/T/D0**	**ICU 14/T/D0**	d.n.r.	d.n.r.	d.n.r.	d.n.r.	d.n.r.
**D15**	**CR2/T/D7**	**ICU 14/T/D7**	d.n.r.	d.n.r.	d.n.r.	d.n.r.	d.n.r.
**D16**	**CR2/T/D180**	**ICU 14/T/D180**	d.n.r.	d.n.r.	d.n.r.	d.n.r.	d.n.r.
**D17**	**CR17/B/D0**	**ICU 23/B/D0**	13831470	1,46	13229508	0,49	2,99
**D18**	**CR17/B/D7**	**ICU 23/B/D7**	11146500	1,17	11261786	0,42	2,83
**D19**	**CR17/B/D180**	**ICU 23/B/D180**	13643550	1,44	10989998	0,41	3,55
**D20**	**CR17/T/D0**	**ICU 23/T/D0**	d.n.r.	d.n.r.	d.n.r.	d.n.r.	d.n.r.
**D21**	**CR17/T/D7**	**ICU 23/T/D7**	d.n.r.	d.n.r.	d.n.r.	d.n.r.	d.n.r.
**D22**	**CR17/T/D180**	**ICU 23/T/D180**	d.n.r.	d.n.r.	d.n.r.	d.n.r.	d.n.r.
	calibrator		8924805	0,94	32745650	1,21	0,78

OPA1-L (upper band)

	WB ID	Control/Patient ID	protein density	protein normalized to calibrator	GAPDH density	GAPDH normalized to calibrator	protein/GAPDH
GEL 5/ set E		cal. mean	6622605,5		cal. mean	13612646,5	
	calibrator		6786894	1,02	13056250	0,96	1,07
E1	H12/B	Control 7/B	9449632	1,43	15730101	1,16	1,23
E2	H12/T	Control 7/T	d.n.r.	d.n.r.	d.n.r.	d.n.r.	d.n.r.
E3	H13/B	Control 1/B	6779204	1,02	17836110	1,31	0,78
E4	H13/T	Control 1/T	d.n.r.	d.n.r.	d.n.r.	d.n.r.	d.n.r.
E5	H18/B	Control 11/B	7881524	1,19	17137798	1,26	0,95
E6	H18/T	Control 11/T	d.n.r.	d.n.r.	d.n.r.	d.n.r.	d.n.r.
E7	CR6/B/D0	ICU 21/B/D0	9265439	1,40	16121115	1,18	1,18
E8	CR6/B/D7	ICU 21/B/D7	5076990	0,77	12428020	0,91	0,84
E9	CR6/T/D0	ICU 21/T/D0	d.n.r.	d.n.r.	d.n.r.	d.n.r.	d.n.r.
E10	CR6/T/D7	ICU 21/T/D7	d.n.r.	d.n.r.	d.n.r.	d.n.r.	d.n.r.
E11	CR14/B/D0	ICU 10/B/D0	6513435	0,98	12644640	0,93	1,06
E12	CR14/B/D7	ICU 10/B/D7	7567282	1,14	13423200	0,99	1,16
E13	CR14/T/D0	ICU 10/T/D0	d.n.r.	d.n.r.	d.n.r.	d.n.r.	d.n.r.
E14	CR14/T/D7	ICU 10/T/D7	d.n.r.	d.n.r.	d.n.r.	d.n.r.	d.n.r.
E15	Sample W/B/D0	Sample W/B/D0	excl.	#####	13286318	0,98	########
E16	Sample W/B/D7	Sample W/B/D7	excl.	#####	14503083	1,07	########
E17	Sample W/T/D0	Sample W/T/D0	excl.	#####	15358237	1,13	########
E18	Sample W/T/D7	Sample W/T/D7	excl.	#####	14918082	1,10	########
E19	CR19/B/D0	ICU 15/B/D0	8358292	1,26	18376065	1,35	0,93
E20	CR19/B/D7	ICU 15/B/D7	7947588	1,20	18750060	1,38	0,87
E21	CR19/T/D0	ICU 15/T/D0	d.n.r.	d.n.r.	d.n.r.	d.n.r.	d.n.r.
E22	CR19/T/D7	ICU 15/T/D7	d.n.r.	d.n.r.	d.n.r.	d.n.r.	d.n.r.
	calibrator		6458317	0,98	14169043	1,04	0,94

OPA1-L (upper band)

	WB ID	Control/Patient ID	protein density	protein normalized to calibrator	GAPDH density	GAPDH normalized to calibrator	protein/GAPDH
GEL 6/ set F		cal. mean	9168219,5		cal. mean	24950732	
	calibrator		9497525	1,04	25509639	1,02	1,01
F1	H9/B	Control 9/B	12429009	1,36	32156010	1,29	1,05
F2	H9/T	Control 9/T	d.n.r.	d.n.r.	d.n.r.	d.n.r.	d.n.r.
F3	H10/B	Control 15/B	10357650	1,13	31970444	1,28	0,88
F4	H10/T	Control 15/T	d.n.r.	d.n.r.	d.n.r.	d.n.r.	d.n.r.
F5	H14/B	Control 20/B	8423622	0,92	25759862	1,03	0,89
F6	H14/T	Control 20/T	d.n.r.	d.n.r.	d.n.r.	d.n.r.	d.n.r.
F7	CR1/B/D0	ICU 1/B/D0	7570035	0,83	24703341	0,99	0,83
F8	CR1/B/D7	ICU 1/B/D7	5860538	0,64	22559436	0,90	0,71
F9	CR1/B/D180	ICU 1/B/D180	6842224	0,75	26013983	1,04	0,72
F10	CR1/T/D0	ICU 1/T/D0	d.n.r.	d.n.r.	d.n.r.	d.n.r.	d.n.r.
F11	CR1/T/D7	ICU 1/T/D7	d.n.r.	d.n.r.	d.n.r.	d.n.r.	d.n.r.
F12	CR1/T/D180	ICU 1/T/D180	d.n.r.	d.n.r.	d.n.r.	d.n.r.	d.n.r.
F13	CR18/B/D0	ICU 18/B/D0	9948240	1,09	24749871	0,99	1,09
F14	CR18/B/D7	ICU 18/B/D7	10238950	1,12	21148778	0,85	1,32
F15	CR18/B/D180	ICU 18/B/D180	9353295	1,02	21108311	0,85	1,21
F16	CR18/T/D0	ICU 18/T/D0	d.n.r.	d.n.r.	d.n.r.	d.n.r.	d.n.r.
F17	CR18/T/D7	ICU 18/T/D7	d.n.r.	d.n.r.	d.n.r.	d.n.r.	d.n.r.
F18	CR18/T/D180	ICU 18/T/D180	d.n.r.	d.n.r.	d.n.r.	d.n.r.	d.n.r.
F19	CR15/B/D0	ICU 12/B/D0	14508383	1,58	27652779	1,11	1,43
F20	CR15/B/D7	ICU 12/B/D7	11674457	1,27	28943200	1,16	1,10
F21	CR15/T/D0	ICU 12/T/D0	d.n.r.	d.n.r.	d.n.r.	d.n.r.	d.n.r.
F22	CR15/T/D7	ICU 12/T/D7	d.n.r.	d.n.r.	d.n.r.	d.n.r.	d.n.r.
	calibrator		8838914	0,96	24391825	0,98	0,99

OPA1-L (upper band)

	WB ID	Control/Patient ID	protein density	protein normalized to calibrator		GAPDH density	GAPDH normalized to calibrator	protein/GAPDH
GEL 7/ set ICU 16		cal. mean	4300663,5		cal. mean	15623570		
	calibrator		4627422	1,08		17691250	1,13	
E15	CR16/B/D0	ICU 16/B/D0	4714126	1,10		14143732	0,91	1,21
E16	CR16/B/D7	ICU 16/B/D7	5150542	1,20		14543912	0,93	1,29
E17	CR16/T/D0	ICU 16/T/D0	d.n.r.	d.n.r.		d.n.r.	d.n.r.	d.n.r.
E18	CR16/T/D7	ICU 16/T/D7	d.n.r.	d.n.r.		d.n.r.	d.n.r.	d.n.r.
	calibrator		3973905	0,92		13555890	0,87	

OPA1-S (lower band)

	WB ID	Control/Patient ID	protein density	protein normalized to calibrator	GAPDH density	GAPDH normalized to calibrator	protein/GAPDH
**GEL 1/ set A**		cal. mean	5465250		cal. mean	41570172	
	calibrator		5782680	1,06	42065400	1,01	1,05
**A1**	**H1/B**	**Control 17/B**	6501600	1,19	42967628	1,03	1,15
**A2**	**H1/T**	**Control 17/T**	d.n.r.	d.n.r.	d.n.r.	d.n.r.	d.n.r.
**A3**	**Sample X/B**	**Sample X/B**	excl.	#####	36839820	0,89	########
**A4**	**Sample X/T**	**Sample X/T**	excl.	#####	32757912	0,79	########
**A5**	**H3/B**	**Control 3/B**	6432660	1,18	35733870	0,86	1,37
**A6**	**H3/T**	**Control 3/T**	d.n.r.	d.n.r.	d.n.r.	d.n.r.	d.n.r.
**A7**	**CR4/B/D0**	**ICU 4/B/D0**	7877835	1,44	28368765	0,68	2,11
**A8**	**CR4/B/D7**	**ICU 4/B/D7**	6490800	1,19	30026880	0,72	1,64
**A9**	**CR4/T/D0**	**ICU 4/T/D0**	d.n.r.	d.n.r.	d.n.r.	d.n.r.	d.n.r.
**A10**	**CR4/T/D7**	**ICU 4/T/D7**	d.n.r.	d.n.r.	d.n.r.	d.n.r.	d.n.r.
**A11**	**CR8/B/D0**	**ICU 11/B/D0**	5926095	1,08	26405840	0,64	1,71
**A12**	**CR8/B/D7**	**ICU 11/B/D7**	5904135	1,08	28186268	0,68	1,59
**A13**	**CR8/T/D0**	**ICU 11/T/D0**	d.n.r.	d.n.r.	d.n.r.	d.n.r.	d.n.r.
**A14**	**CR8/T/D7**	**ICU 11/T/D7**	d.n.r.	d.n.r.	d.n.r.	d.n.r.	d.n.r.
**A15**	**CR10/B/D0**	**ICU 13/B/D0**	5565834	1,02	32125544	0,77	1,32
**A16**	**CR10/B/D7**	**ICU 13/B/D7**	7017840	1,28	37040626	0,89	1,44
**A17**	**CR10/T/D0**	**ICU 13/T/D0**	d.n.r.	d.n.r.	d.n.r.	d.n.r.	d.n.r.
**A18**	**CR10/T/D7**	**ICU 13/T/D7**	d.n.r.	d.n.r.	d.n.r.	d.n.r.	d.n.r.
**A19**	**CR11/B/D0**	**ICU 9/B/D0**	8033212	1,47	34330306	0,83	1,78
**A20**	**CR11/B/D7**	**ICU 9/B/D7**	5454206	1,00	33386188	0,80	1,24
**A21**	**CR11/T/D0**	**ICU 9/T/D0**	d.n.r.	d.n.r.	d.n.r.	d.n.r.	d.n.r.
**A22**	**CR11/T/D7**	**ICU 9/T/D7**	d.n.r.	d.n.r.	d.n.r.	d.n.r.	d.n.r.
	calibrator		5147820	0,94	41074944	0,99	0,95

OPA1-S (lower band)

	WB ID	Control/Patient ID	protein density	protein normalized to calibrator	GAPDH density	GAPDH normalized to calibrator	protein/GAPDH
GEL 2/ set B		cal. mean	6661993		cal. mean 39443078,5		
	calibrator		5960070	0,89	31672407	0,80	1,11
**B1**	**H4/B**	**Control 12/B**	6200325	0,93	34761556	0,88	1,06
**B2**	**H4/T**	**Control 12/T**	d.n.r.	d.n.r.	d.n.r.	d.n.r.	d.n.r.
**B3**	**H5/B**	**Control 6/B**	6801525	1,02	31297500	0,79	1,29
**B4**	**H5/T**	**Control 6/T**	d.n.r.	d.n.r.	d.n.r.	d.n.r.	d.n.r.
**B5**	**H6/B**	**Control 8/B**	7059060	1,06	26830935	0,68	1,56
**B6**	**H6/T**	**Control 8/T**	d.n.r.	d.n.r.	d.n.r.	d.n.r.	d.n.r.
**B7**	**Sample Y/B**	**Sample Y/B**	excl.	#####	25290990	0,64	########
**B8**	**Sample Y/T**	**Sample Y/T**	excl.	#####	23090160	0,59	########
**B9**	**Sample Z/B**	**Sample Z/B**	excl.	#####	25183170	0,64	########
**B10**	**Sample Z/T**	**Sample Z/T**	excl.	#####	22048708	0,56	########
**B11**	**CR9/B/D0**	**ICU 3/B/D0**	5120100	0,77	28875120	0,73	1,05
**B12**	**CR9/B/D7**	**ICU 3/B/D7**	4548150	0,68	32768164	0,83	0,82
**B13**	**CR9/T/D0**	**ICU 3/T/D0**	d.n.r.	d.n.r.	d.n.r.	d.n.r.	d.n.r.
**B14**	**CR9/T/D7**	**ICU 3/T/D7**	d.n.r.	d.n.r.	d.n.r.	d.n.r.	d.n.r.
**B15**	**CR12/B/D0**	**ICU 20/B/D0**	5397747	0,81	32436270	0,82	0,99
**B16**	**CR12/B/D7**	**ICU 20/B/D7**	7798516	1,17	33377085	0,85	1,38
**B17**	**CR12/T/D0**	**ICU 20/T/D0**	d.n.r.	d.n.r.	d.n.r.	d.n.r.	d.n.r.
**B18**	**CR12/T/D7**	**ICU 20/T/D7**	d.n.r.	d.n.r.	d.n.r.	d.n.r.	d.n.r.
**B19**	**CR13/B/D0**	**ICU 22/B/D0**	9759992	1,47	35632337	0,90	1,62
**B20**	**CR13/B/D7**	**ICU 22/B/D7**	11361976	1,71	35268420	0,89	1,91
**B21**	**CR13/T/D0**	**ICU 22/T/D0**	d.n.r.	d.n.r.	d.n.r.	d.n.r.	d.n.r.
**B22**	**CR13/T/D7**	**ICU 22/T/D7**	d.n.r.	d.n.r.	d.n.r.	d.n.r.	d.n.r.
	calibrator		7363916	1,11	47213750	1,20	0,92

OPA1-S (lower band)

	WB ID	Control/Patient ID	protein density	protein normalized to calibrator	GAPDH density	GAPDH normalized to calibrator	protein/GAPDH
GEL 3/ set C		cal. mean	1345471,5	cal. mean	22029750		
	calibrator		1737731	1,29	20662800	0,94	1,38
C1	H15/B	Control 2/B	1589116	1,18	20219460	0,92	1,29
C2	H15/T	Control 2/T	d.n.r.	d.n.r.	d.n.r.	d.n.r.	d.n.r.
C3	H16/B	Control 14/B	1797268	1,34	15286365	0,69	1,93
C4	H16/T	Control 14/T	d.n.r.	d.n.r.	d.n.r.	d.n.r.	d.n.r.
C5	H17/B	Control 10/B	1386840	1,03	14141864	0,64	1,61
C6	H17/T	Control 10/T	d.n.r.	d.n.r.	d.n.r.	d.n.r.	d.n.r.
C7	CR3/B/D0	ICU 5/B/D0	1674998	1,24	14270928	0,65	1,92
C8	CR3/B/D7	ICU 5/B/D7	1804488	1,34	13239864	0,60	2,23
C9	CR3/T/D0	ICU 5/T/D0	d.n.r.	d.n.r.	d.n.r.	d.n.r.	d.n.r.
C10	CR3/T/D7	ICU 5/T/D7	d.n.r.	d.n.r.	d.n.r.	d.n.r.	d.n.r.
C11	CR5/B/D0	ICU 8/B/D0	1040474	0,77	18034032	0,82	0,94
C12	CR5/B/D7	ICU 8/B/D7	1137810	0,85	15358512	0,70	1,21
C13	CR5/T/D0	ICU 8/T/D0	d.n.r.	d.n.r.	d.n.r.	d.n.r.	d.n.r.
C14	CR5/T/D7	ICU 8/T/D7	d.n.r.	d.n.r.	d.n.r.	d.n.r.	d.n.r.
C15	CR7/B/D0	ICU 6/B/D0	1766160	1,31	17619684	0,80	1,64
C16	CR7/B/D7	ICU 6/B/D7	1928182	1,43	17926524	0,81	1,76
C17	CD7/T/D0	ICU 6/T/D0	d.n.r.	d.n.r.	d.n.r.	d.n.r.	d.n.r.
C18	CR7/T/D7	ICU 6/T/D7	d.n.r.	d.n.r.	d.n.r.	d.n.r.	d.n.r.
C19	CR20/B/D0	ICU 7/B/D0	1964025	1,46	14741100	0,67	2,18
C20	CR20/B/D7	ICU 7/B/D7	1964568	1,46	15625234	0,71	2,06
C21	CR20/T/D0	ICU 7/T/D0	d.n.r.	d.n.r.	d.n.r.	d.n.r.	d.n.r.
C22	CR20/T/D7	ICU 7/T/D7	d.n.r.	d.n.r.	d.n.r.	d.n.r.	d.n.r.
	calibrator		953212	0,71	23396700	1,06	0,67

OPA1-S (lower band)

	WB ID	Control/Patient ID	protein density	protein normalized to calibrator		GAPDH density	GAPDH normalized to calibrator	protein/GAPDH
GEL 4/ set D		cal. mean	1052190		cal. mean	27123350		
	calibrator		1299420	1,23		21501050	0,79	1,56
D1	H2/B	Control 4/B	1446660	1,37		15463789	0,57	2,41
D2	H2/T	Control 4/T	d.n.r.	d.n.r.		d.n.r.	d.n.r.	d.n.r.
D3	H7/B	Control 13/B	467865	0,44		15370470	0,57	0,78
D4	H7/T	Control 13/T	d.n.r.	d.n.r.		d.n.r.	d.n.r.	d.n.r.
D5	H8/B	Control 18/B	749385	0,71		13315368	0,49	1,45
D6	H8/T	Control 18/T	d.n.r.	d.n.r.		d.n.r.	d.n.r.	d.n.r.
D7	H11/B	Control 19/B	747405	0,71		15176872	0,56	1,27
D8	H11/T	Control 19/T	d.n.r.	d.n.r.		d.n.r.	d.n.r.	d.n.r.
D9	H19/B	Control 5/B	1577970	1,50		12146022	0,45	3,35
D10	H19/T	Control 5/T	d.n.r.	d.n.r.		d.n.r.	d.n.r.	d.n.r.
D11	CR2/B/D0	ICU 14/B/D0	1109340	1,05		13352832	0,49	2,14
D12	CR2/B/D7	ICU 14/B/D7	1198350	1,14		14137728	0,52	2,19
D13	CR2/B/D180	ICU 14/B/D180	1285380	1,22		11800978	0,44	2,81
D14	CR2/T/D0	ICU 14/T/D0	d.n.r.	d.n.r.		d.n.r.	d.n.r.	d.n.r.
D15	CR2/T/D7	ICU 14/T/D7	d.n.r.	d.n.r.		d.n.r.	d.n.r.	d.n.r.
D16	CR2/T/D180	ICU 14/T/D180	d.n.r.	d.n.r.		d.n.r.	d.n.r.	d.n.r.
D17	CR17/B/D0	ICU 23/B/D0	2140785	2,03		13229508	0,49	4,17
D18	CR17/B/D7	ICU 23/B/D7	1423890	1,35		11261786	0,42	3,26
D19	CR17/B/D180	ICU 23/B/D180	834750	0,79		10989998	0,41	1,96
D20	CR17/T/D0	ICU 23/T/D0	d.n.r.	d.n.r.		d.n.r.	d.n.r.	d.n.r.
D21	CR17/T/D7	ICU 23/T/D7	d.n.r.	d.n.r.		d.n.r.	d.n.r.	d.n.r.
D22	CR17/T/D180	ICU 23/T/D180	d.n.r.	d.n.r.		d.n.r.	d.n.r.	d.n.r.
	calibrator		804960	0,77		32745650	1,21	0,63

OPA1-S (lower band)

	WB ID	Control/Patient ID	protein density	protein normalized to calibrator	GAPDH density	GAPDH normalized to calibrator	protein/GAPDH
GEL 5/ set E		cal. mean	1124992	cal. mean	13612646,5		
	calibrator		1314167	1,17	13056250	0,96	1,22
E1	H12/B	Control 7/B	2876588	2,56	15730101	1,16	2,21
E2	H12/T	Control 7/T	d.n.r.	d.n.r.	d.n.r.	d.n.r.	d.n.r.
E3	H13/B	Control 1/B	890284	0,79	17836110	1,31	0,60
E4	H13/T	Control 1/T	d.n.r.	d.n.r.	d.n.r.	d.n.r.	d.n.r.
E5	H18/B	Control 11/B	3802676	3,38	17137798	1,26	2,68
E6	H18/T	Control 11/T	d.n.r.	d.n.r.	d.n.r.	d.n.r.	d.n.r.
E7	CR6/B/D0	ICU 21/B/D0	2508155	2,23	16121115	1,18	1,88
E8	CR6/B/D7	ICU 21/B/D7	2714850	2,41	12428020	0,91	2,64
E9	CR6/T/D0	ICU 21/T/D0	d.n.r.	d.n.r.	d.n.r.	d.n.r.	d.n.r.
E10	CR6/T/D7	ICU 21/T/D7	d.n.r.	d.n.r.	d.n.r.	d.n.r.	d.n.r.
E11	CR14/B/D0	ICU 10/B/D0	2091915	1,86	12644640	0,93	2,00
E12	CR14/B/D7	ICU 10/B/D7	2358648	2,10	13423200	0,99	2,13
E13	CR14/T/D0	ICU 10/T/D0	d.n.r.	d.n.r.	d.n.r.	d.n.r.	d.n.r.
E14	CR14/T/D7	ICU 10/T/D7	d.n.r.	d.n.r.	d.n.r.	d.n.r.	d.n.r.
E15	Sample W/B/D0	Sample W/B/D0	excl.	#####	13286318	0,98	########
E16	Sample W/B/D7	Sample W/B/D7	excl.	#####	14503083	1,07	########
E17	Sample W/T/D0	Sample W/T/D0	excl.	#####	15358237	1,13	########
E18	Sample W/T/D7	Sample W/T/D7	excl.	#####	14918082	1,10	########
E19	CR19/B/D0	ICU 15/B/D0	1357219	1,21	18376065	1,35	0,89
E20	CR19/B/D7	ICU 15/B/D7	2092420	1,86	18750060	1,38	1,35
E21	CR19/T/D0	ICU 15/T/D0	d.n.r.	d.n.r.	d.n.r.	d.n.r.	d.n.r.
E22	CR19/T/D7	ICU 15/T/D7	d.n.r.	d.n.r.	d.n.r.	d.n.r.	d.n.r.
	calibrator		935817	0,83	14169043	1,04	0,80

OPA1-S (lower band)

	WB ID	Control/Patient ID	protein density	protein normalized to calibrator	GAPDH density	GAPDH normalized to calibrator	protein/GAPDH
GEL 6/ set F		cal. mean	1426027		cal. mean	24950732	
	calibrator		1944296	1,36	25509639	1,02	1,33
F1	H9/B	Control 9/B	2221267	1,56	32156010	1,29	1,21
F2	H9/T	Control 9/T	d.n.r.	d.n.r.	d.n.r.	d.n.r.	d.n.r.
F3	H10/B	Control 15/B	2624670	1,84	31970444	1,28	1,44
F4	H10/T	Control 15/T	d.n.r.	d.n.r.	d.n.r.	d.n.r.	d.n.r.
F5	H14/B	Control 20/B	1387957	0,97	25759862	1,03	0,94
F6	H14/T	Control 20/T	d.n.r.	d.n.r.	d.n.r.	d.n.r.	d.n.r.
F7	CR1/B/D0	ICU 1/B/D0	1557990	1,09	24703341	0,99	1,10
F8	CR1/B/D7	ICU 1/B/D7	2589570	1,82	22559436	0,90	2,01
F9	CR1/B/D180	ICU 1/B/D180	2190382	1,54	26013983	1,04	1,47
F10	CR1/T/D0	ICU 1/T/D0	d.n.r.	d.n.r.	d.n.r.	d.n.r.	d.n.r.
F11	CR1/T/D7	ICU 1/T/D7	d.n.r.	d.n.r.	d.n.r.	d.n.r.	d.n.r.
F12	CR1/T/D180	ICU 1/T/D180	d.n.r.	d.n.r.	d.n.r.	d.n.r.	d.n.r.
F13	CR18/B/D0	ICU 18/B/D0	2827440	1,98	24749871	0,99	2,00
F14	CR18/B/D7	ICU 18/B/D7	3493275	2,45	21148778	0,85	2,89
F15	CR18/B/D180	ICU 18/B/D180	2266110	1,59	21108311	0,85	1,88
F16	CR18/T/D0	ICU 18/T/D0	d.n.r.	d.n.r.	d.n.r.	d.n.r.	d.n.r.
F17	CR18/T/D7	ICU 18/T/D7	d.n.r.	d.n.r.	d.n.r.	d.n.r.	d.n.r.
F18	CR18/T/D180	ICU 18/T/D180	d.n.r.	d.n.r.	d.n.r.	d.n.r.	d.n.r.
F19	CR15/B/D0	ICU 12/B/D0	1371460	0,96	27652779	1,11	0,87
F20	CR15/B/D7	ICU 12/B/D7	1403391	0,98	28943200	1,16	0,85
F21	CR15/T/D0	ICU 12/T/D0	d.n.r.	d.n.r.	d.n.r.	d.n.r.	d.n.r.
F22	CR15/T/D7	ICU 12/T/D7	d.n.r.	d.n.r.	d.n.r.	d.n.r.	d.n.r.
	calibrator		907758	0,64	24391825	0,98	0,65

OPA1-S (lower band)

	WB ID	Control/Patient ID	protein density	protein normalized to calibrator	GAPDH density	GAPDH normalized to calibrator	protein/GAPDH
GEL 7/ set ICU 16		cal. mean	2279205		cal. mean 15623570		
	calibrator		2556360	1,12	17691250	1,13	
**E15**	**CR16/B/D0**	**ICU 16/B/D0**	3539884	1,55	14143732	0,91	1,72
**E16**	**CR16/B/D7**	**ICU 16/B/D7**	3578345	1,57	14543912	0,93	1,69
**E17**	**CR16/T/D0**	**ICU 16/T/D0**	d.n.r.	d.n.r.	d.n.r.	d.n.r.	d.n.r.
**E18**	**CR16/T/D7**	**ICU 16/T/D7**	d.n.r.	d.n.r.	d.n.r.	d.n.r.	d.n.r.
	calibrator		2002050	0,88	13555890	0,87	

OPA1-L vs. OPA1-S

	WB ID	Control/Patient ID	OPA1-L density	OPA1-L normalized to calibrator	OPA1-S density	OPA1-S normalized to calibrator	OPA1-L/OPA1-S
GEL 1/ set A		cal. mean	6214612,5		cal. mean 5465250		
	calibrator		7497630	1,21	5782680	1,06	1,14
A1	H1/B	Control 17/B	7544340	1,21	6501600	1,19	1,02
A2	H1/T	Control 17/T	d.n.r.	d.n.r.	d.n.r.	d.n.r.	d.n.r.
A3	Sample X/B	Sample X/B	excl.	#####	5757120	1,05	########
A4	Sample X/T	Sample X/T	excl.	#####	3401955	0,62	########
A5	H3/B	Control 3/B	7926660	1,28	6432660	1,18	1,08
A6	H3/T	Control 3/T	d.n.r.	d.n.r.	d.n.r.	d.n.r.	d.n.r.
A7	CR4/B/D0	ICU 4/B/D0	5397570	0,87	7877835	1,44	0,60
A8	CR4/B/D7	ICU 4/B/D7	5267475	0,85	6490800	1,19	0,71
A9	CR4/T/D0	ICU 4/T/D0	d.n.r.	d.n.r.	d.n.r.	d.n.r.	d.n.r.
A10	CR4/T/D7	ICU 4/T/D7	d.n.r.	d.n.r.	d.n.r.	d.n.r.	d.n.r.
A11	CR8/B/D0	ICU 11/B/D0	5111010	0,82	5926095	1,08	0,76
A12	CR8/B/D7	ICU 11/B/D7	5631615	0,91	5904135	1,08	0,84
A13	CR8/T/D0	ICU 11/T/D0	d.n.r.	d.n.r.	d.n.r.	d.n.r.	d.n.r.
A14	CR8/T/D7	ICU 11/T/D7	d.n.r.	d.n.r.	d.n.r.	d.n.r.	d.n.r.
A15	CR10/B/D0	ICU 13/B/D0	5443843	0,88	5565834	1,02	0,86
A16	CR10/B/D7	ICU 13/B/D7	5619645	0,90	7017840	1,28	0,70
A17	CR10/T/D0	ICU 13/T/D0	d.n.r.	d.n.r.	d.n.r.	d.n.r.	d.n.r.
A18	CR10/T/D7	ICU 13/T/D7	d.n.r.	d.n.r.	d.n.r.	d.n.r.	d.n.r.
A19	CR11/B/D0	ICU 9/B/D0	5215408	0,84	8033212	1,47	0,57
A20	CR11/B/D7	ICU 9/B/D7	6976578	1,12	5454206	1,00	1,12
A21	CR11/T/D0	ICU 9/T/D0	d.n.r.	d.n.r.	d.n.r.	d.n.r.	d.n.r.
A22	CR11/T/D7	ICU 9/T/D7	d.n.r.	d.n.r.	d.n.r.	d.n.r.	d.n.r.
	calibrator		4931595	0,79	5147820	0,94	0,84

OPA1-L vs. OPA1-S

GEL 2/ set B

	WB ID	Control/Patient ID	OPA1-L density	OPA1-L normalized to calibrator	OPA1-S density	OPA1-S normalized to calibrator	OPA1-L/OPA1-S
		cal. mean	6667057	cal. mean	6661993		
	calibrator		6068835	0,91	5960070	0,89	1,02
**B1**	**H4/B**	**Control 12/B**	5838840	0,88	6200325	0,93	0,94
**B2**	**H4/T**	**Control 12/T**	d.n.r.	d.n.r.	d.n.r.	d.n.r.	d.n.r.
**B3**	**H5/B**	**Control 6/B**	5453280	0,82	6801525	1,02	0,80
**B4**	**H5/T**	**Control 6/T**	d.n.r.	d.n.r.	d.n.r.	d.n.r.	d.n.r.
**B5**	**H6/B**	**Control 8/B**	5134680	0,77	7059060	1,06	0,73
**B6**	**H6/T**	**Control 8/T**	d.n.r.	d.n.r.	d.n.r.	d.n.r.	d.n.r.
**B7**	**Sample Y/B**	**Sample Y/B**	excl.	#####	5277105	0,79	########
**B8**	**Sample Y/T**	**Sample Y/T**	excl.	#####	4347990	0,65	########
**B9**	**Sample Z/B**	**Sample Z/B**	excl.	#####	5321070	0,80	########
**B10**	**Sample Z/T**	**Sample Z/T**	excl.	#####	4737735	0,71	########
**B11**	**CR9/B/D0**	**ICU 3/B/D0**	3499245	0,52	5120100	0,77	0,68
**B12**	**CR9/B/D7**	**ICU 3/B/D7**	3088710	0,46	4548150	0,68	0,68
**B13**	**CR9/T/D0**	**ICU 3/T/D0**	d.n.r.	d.n.r.	d.n.r.	d.n.r.	d.n.r.
**B14**	**CR9/T/D7**	**ICU 3/T/D7**	d.n.r.	d.n.r.	d.n.r.	d.n.r.	d.n.r.
**B15**	**CR12/B/D0**	**ICU 20/B/D0**	3439484	0,52	5397747	0,81	0,64
**B16**	**CR12/B/D7**	**ICU 20/B/D7**	4561744	0,68	7798516	1,17	0,58
**B17**	**CR12/T/D0**	**ICU 20/T/D0**	d.n.r.	d.n.r.	d.n.r.	d.n.r.	d.n.r.
**B18**	**CR12/T/D7**	**ICU 20/T/D7**	d.n.r.	d.n.r.	d.n.r.	d.n.r.	d.n.r.
**B19**	**CR13/B/D0**	**ICU 22/B/D0**	5225616	0,78	9759992	1,47	0,54
**B20**	**CR13/B/D7**	**ICU 22/B/D7**	5349544	0,80	11361976	1,71	0,47
**B21**	**CR13/T/D0**	**ICU 22/T/D0**	d.n.r.	d.n.r.	d.n.r.	d.n.r.	d.n.r.
**B22**	**CR13/T/D7**	**ICU 22/T/D7**	d.n.r.	d.n.r.	d.n.r.	d.n.r.	d.n.r.
	calibrator		7265279	1,09	7363916	1,11	0,99

OPA1-L vs. OPA1-S

	WB ID	Control/Patient ID	OPA1-L density	OPA1-L normalized to calibrator	OPA1-S density	OPA1-S normalized to calibrator	OPA1-L/OPA1-S
**GEL 3/ set C**		cal. mean	12017075		cal. mean 1345471,5		
	calibrator		11766732	0,98	1737731	1,29	0,76
**C1**	**H15/B**	**Control 2/B**	11093084	0,92	1589116	1,18	0,78
**C2**	**H15/T**	**Control 2/T**	d.n.r.	d.n.r.	d.n.r.	d.n.r.	d.n.r.
**C3**	**H16/B**	**Control 14/B**	11870276	0,99	1797268	1,34	0,74
**C4**	**H16/T**	**Control 14/T**	d.n.r.	d.n.r.	d.n.r.	d.n.r.	d.n.r.
**C5**	**H17/B**	**Control 10/B**	11324460	0,94	1386840	1,03	0,91
**C6**	**H17/T**	**Control 10/T**	d.n.r.	d.n.r.	d.n.r.	d.n.r.	d.n.r.
**C7**	**CR3/B/D0**	**ICU 5/B/D0**	7537652	0,63	1674998	1,24	0,50
**C8**	**CR3/B/D7**	**ICU 5/B/D7**	9888942	0,82	1804488	1,34	0,61
**C9**	**CR3/T/D0**	**ICU 5/T/D0**	d.n.r.	d.n.r.	d.n.r.	d.n.r.	d.n.r.
**C10**	**CR3/T/D7**	**ICU 5/T/D7**	d.n.r.	d.n.r.	d.n.r.	d.n.r.	d.n.r.
**C11**	**CR5/B/D0**	**ICU 8/B/D0**	12336970	1,03	1040474	0,77	1,33
**C12**	**CR5/B/D7**	**ICU 8/B/D7**	10709352	0,89	1137810	0,85	1,05
**C13**	**CR5/T/D0**	**ICU 8/T/D0**	d.n.r.	d.n.r.	d.n.r.	d.n.r.	d.n.r.
**C14**	**CR5/T/D7**	**ICU 8/T/D7**	d.n.r.	d.n.r.	d.n.r.	d.n.r.	d.n.r.
**C15**	**CR7/B/D0**	**ICU 6/B/D0**	12870968	1,07	1766160	1,31	0,82
**C16**	**CR7/B/D7**	**ICU 6/B/D7**	10077036	0,84	1928182	1,43	0,59
**C17**	**CD7/T/D0**	**ICU 6/T/D0**	d.n.r.	d.n.r.	d.n.r.	d.n.r.	d.n.r.
**C18**	**CR7/T/D7**	**ICU 6/T/D7**	d.n.r.	d.n.r.	d.n.r.	d.n.r.	d.n.r.
**C19**	**CR20/B/D0**	**ICU 7/B/D0**	12286003	1,02	1964025	1,46	0,70
**C20**	**CR20/B/D7**	**ICU 7/B/D7**	14452924	1,20	1964568	1,46	0,82
**C21**	**CR20/T/D0**	**ICU 7/T/D0**	d.n.r.	d.n.r.	d.n.r.	d.n.r.	d.n.r.
**C22**	**CR20/T/D7**	**ICU 7/T/D7**	d.n.r.	d.n.r.	d.n.r.	d.n.r.	d.n.r.
	calibrator		12267418	1,02	953212	0,71	1,44

OPA1-L vs. OPA1-S

	WB ID	Control/Patient ID	OPA1-L density	OPA1-L normalized to calibrator	OPA1-S density	OPA1-S normalized to calibrator	OPA1-L/OPA1-S
GEL 4/ set D		cal. mean	9487102,5		cal. mean	1052190	
	calibrator		10049400	1,06	1299420	1,23	0,86
D1	H2/B	Control 4/B	12756555	1,34	1446660	1,37	0,98
D2	H2/T	Control 4/T	d.n.r.	d.n.r.	d.n.r.	d.n.r.	d.n.r.
D3	H7/B	Control 13/B	5276070	0,56	467865	0,44	1,25
D4	H7/T	Control 13/T	d.n.r.	d.n.r.	d.n.r.	d.n.r.	d.n.r.
D5	H8/B	Control 18/B	4978980	0,52	749385	0,71	0,74
D6	H8/T	Control 18/T	d.n.r.	d.n.r.	d.n.r.	d.n.r.	d.n.r.
D7	H11/B	Control 19/B	5876325	0,62	747405	0,71	0,87
D8	H11/T	Control 19/T	d.n.r.	d.n.r.	d.n.r.	d.n.r.	d.n.r.
D9	H19/B	Control 5/B	7436700	0,78	1577970	1,50	0,52
D10	H19/T	Control 5/T	d.n.r.	d.n.r.	d.n.r.	d.n.r.	d.n.r.
D11	CR2/B/D0	ICU 14/B/D0	8344395	0,88	1109340	1,05	0,83
D12	CR2/B/D7	ICU 14/B/D7	7844400	0,83	1198350	1,14	0,73
D13	CR2/B/D180	ICU 14/B/D180	8646750	0,91	1285380	1,22	0,75
D14	CR2/T/D0	ICU 14/T/D0	d.n.r.	d.n.r.	d.n.r.	d.n.r.	d.n.r.
D15	CR2/T/D7	ICU 14/T/D7	d.n.r.	d.n.r.	d.n.r.	d.n.r.	d.n.r.
D16	CR2/T/D180	ICU 14/T/D180	d.n.r.	d.n.r.	d.n.r.	d.n.r.	d.n.r.
D17	CR17/B/D0	ICU 23/B/D0	13831470	1,46	2140785	2,03	0,72
D18	CR17/B/D7	ICU 23/B/D7	11146500	1,17	1423890	1,35	0,87
D19	CR17/B/D180	ICU 23/B/D180	13643550	1,44	834750	0,79	1,81
D20	CR17/T/D0	ICU 23/T/D0	d.n.r.	d.n.r.	d.n.r.	d.n.r.	d.n.r.
D21	CR17/T/D7	ICU 23/T/D7	d.n.r.	d.n.r.	d.n.r.	d.n.r.	d.n.r.
D22	CR17/T/D180	ICU 23/T/D180	d.n.r.	d.n.r.	d.n.r.	d.n.r.	d.n.r.
	calibrator		8924805	0,94	804960	0,77	1,23

OPA1-L vs. OPA1-S

	WB ID	Control/Patient ID	OPA1-L density	OPA1-L normalized to calibrator	OPA1-S density	OPA1-S normalized to calibrator	OPA1-L/OPA1-S
GEL 5/ set E		cal. mean	6622605,5	cal. mean	1124992		
	calibrator		6786894	1,02	1314167	1,17	0,88
E1	H12/B	Control 7/B	9449632	1,43	2876588	2,56	0,56
E2	H12/T	Control 7/T	d.n.r.	d.n.r.	d.n.r.	d.n.r.	d.n.r.
E3	H13/B	Control 1/B	6779204	1,02	890284	0,79	1,29
E4	H13/T	Control 1/T	d.n.r.	d.n.r.	d.n.r.	d.n.r.	d.n.r.
E5	H18/B	Control 11/B	7881524	1,19	3802676	3,38	0,35
E6	H18/T	Control 11/T	d.n.r.	d.n.r.	d.n.r.	d.n.r.	d.n.r.
E7	CR6/B/D0	ICU 21/B/D0	9265439	1,40	2508155	2,23	0,63
E8	CR6/B/D7	ICU 21/B/D7	5076990	0,77	2714850	2,41	0,32
E9	CR6/T/D0	ICU 21/T/D0	d.n.r.	d.n.r.	d.n.r.	d.n.r.	d.n.r.
E10	CR6/T/D7	ICU 21/T/D7	d.n.r.	d.n.r.	d.n.r.	d.n.r.	d.n.r.
E11	CR14/B/D0	ICU 10/B/D0	6513435	0,98	2091915	1,86	0,53
E12	CR14/B/D7	ICU 10/B/D7	7567282	1,14	2358648	2,10	0,55
E13	CR14/T/D0	ICU 10/T/D0	d.n.r.	d.n.r.	d.n.r.	d.n.r.	d.n.r.
E14	CR14/T/D7	ICU 10/T/D7	d.n.r.	d.n.r.	d.n.r.	d.n.r.	d.n.r.
E15	Sample W/B/D0	Sample W/B/D0	excl.	#####	1987965	1,77	########
E16	Sample W/B/D7	Sample W/B/D7	excl.	#####	2193349	1,95	########
E17	Sample W/T/D0	Sample W/T/D0	excl.	#####	28341	0,03	########
E18	Sample W/T/D7	Sample W/T/D7	excl.	#####	23265	0,02	########
E19	CR19/B/D0	ICU 15/B/D0	8358292	1,26	1357219	1,21	1,05
E20	CR19/B/D7	ICU 15/B/D7	7947588	1,20	2092420	1,86	0,65
E21	CR19/T/D0	ICU 15/T/D0	d.n.r.	d.n.r.	d.n.r.	d.n.r.	d.n.r.
E22	CR19/T/D7	ICU 15/T/D7	d.n.r.	d.n.r.	d.n.r.	d.n.r.	d.n.r.
	calibrator		6458317	0,98	935817	0,83	1,17

OPA1-L vs. OPA1-S

	WB ID	Control/Patient ID	OPA1-L density	OPA1-L normalized to calibrator	OPA1-S density	OPA1-S normalized to calibrator	OPA1-L/OPA1-S
GEL 6/ set F		cal. mean	9168219,5		cal. mean	1426027	
	calibrator		9497525	1,04	1944296	1,36	0,76
F1	H9/B	Control 9/B	12429009	1,36	2221267	1,56	0,87
F2	H9/T	Control 9/T	d.n.r.	d.n.r.	d.n.r.	d.n.r.	d.n.r.
F3	H10/B	Control 15/B	10357650	1,13	2624670	1,84	0,61
F4	H10/T	Control 15/T	d.n.r.	d.n.r.	d.n.r.	d.n.r.	d.n.r.
F5	H14/B	Control 20/B	8423622	0,92	1387957	0,97	0,94
F6	H14/T	Control 20/T	d.n.r.	d.n.r.	d.n.r.	d.n.r.	d.n.r.
F7	CR1/B/D0	ICU 1/B/D0	7570035	0,83	1557990	1,09	0,76
F8	CR1/B/D7	ICU 1/B/D7	5860538	0,64	2589570	1,82	0,35
F9	CR1/B/D180	ICU 1/B/D180	6842224	0,75	2190382	1,54	0,49
F10	CR1/T/D0	ICU 1/T/D0	d.n.r.	d.n.r.	d.n.r.	d.n.r.	d.n.r.
F11	CR1/T/D7	ICU 1/T/D7	d.n.r.	d.n.r.	d.n.r.	d.n.r.	d.n.r.
F12	CR1/T/D180	ICU 1/T/D180	d.n.r.	d.n.r.	d.n.r.	d.n.r.	d.n.r.
F13	CR18/B/D0	ICU 18/B/D0	9948240	1,09	2827440	1,98	0,55
F14	CR18/B/D7	ICU 18/B/D7	10238950	1,12	3493275	2,45	0,46
F15	CR18/B/D180	ICU 18/B/D180	9353295	1,02	2266110	1,59	0,64
F16	CR18/T/D0	ICU 18/T/D0	d.n.r.	d.n.r.	d.n.r.	d.n.r.	d.n.r.
F17	CR18/T/D7	ICU 18/T/D7	d.n.r.	d.n.r.	d.n.r.	d.n.r.	d.n.r.
F18	CR18/T/D180	ICU 18/T/D180	d.n.r.	d.n.r.	d.n.r.	d.n.r.	d.n.r.
F19	CR15/B/D0	ICU 12/B/D0	14508383	1,58	1371460	0,96	1,65
F20	CR15/B/D7	ICU 12/B/D7	11674457	1,27	1403391	0,98	1,29
F21	CR15/T/D0	ICU 12/T/D0	d.n.r.	d.n.r.	d.n.r.	d.n.r.	d.n.r.
F22	CR15/T/D7	ICU 12/T/D7	d.n.r.	d.n.r.	d.n.r.	d.n.r.	d.n.r.
	calibrator		8838914	0,96	907758	0,64	1,51

OPA1-L vs. OPA1-S

	WB ID	Control/Patient ID	OPA1-L density	OPA1-L normalized to calibrator	OPA1-S density	OPA1-S normalized to calibrator	OPA1-L/OPA1-S
GEL 7/ set ICU 16		cal. mean	4300663,5	cal. mean	2279205		
	calibrator		4627422	1,08	2556360	1,12	
E15	CR16/B/D0	ICU 16/B/D0	4714126	1,10	3539884	1,55	0,71
E16	CR16/B/D7	ICU 16/B/D7	5150542	1,20	3578345	1,57	0,76
E17	CR16/T/D0	ICU 16/T/D0	d.n.r.	d.n.r.	d.n.r.	d.n.r.	d.n.r.
E18	CR16/T/D7	ICU 16/T/D7	d.n.r.	d.n.r.	d.n.r.	d.n.r.	d.n.r.
	calibrator		3973905	0,92	2002050	0,88	

Lipin 1

	WB ID	Control/Patient ID	protein density	protein normalized to calibrator	GAPDH density	GAPDH normalized to calibrator	protein/GAPDH
GEL 1/ set A		cal. mean	5621254		cal. mean	25207886	
	calibrator		6824688	1,21	26237938	1,04	1,17
**A1**	**H1/B**	**Control 17/B**	6087080	1,08	25525559	1,01	1,07
**A2**	**H1/T**	**Control 17/T**	d.n.r.	d.n.r.	d.n.r.	d.n.r.	d.n.r.
**A3**	**Sample X/B**	**Sample X/B**	excl.	#####	20146728	0,80	########
**A4**	**Sample X/T**	**Sample X/T**	excl.	#####	19557452	0,78	########
**A5**	**H3/B**	**Control 3/B**	3010946	0,54	20382535	0,81	0,66
**A6**	**H3/T**	**Control 3/T**	d.n.r.	d.n.r.	d.n.r.	d.n.r.	d.n.r.
**A7**	**CR4/B/D0**	**ICU 4/B/D0**	4707270	0,84	20062467	0,80	1,05
**A8**	**CR4/B/D7**	**ICU 4/B/D7**	5608748	1,00	19913007	0,79	1,26
**A9**	**CR4/T/D0**	**ICU 4/T/D0**	d.n.r.	d.n.r.	d.n.r.	d.n.r.	d.n.r.
**A10**	**CR4/T/D7**	**ICU 4/T/D7**	d.n.r.	d.n.r.	d.n.r.	d.n.r.	d.n.r.
**A11**	**CR8/B/D0**	**ICU 11/B/D0**	7493395	1,33	16150422	0,64	2,08
**A12**	**CR8/B/D7**	**ICU 11/B/D7**	6137261	1,09	17634541	0,70	1,56
**A13**	**CR8/T/D0**	**ICU 11/T/D0**	d.n.r.	d.n.r.	d.n.r.	d.n.r.	d.n.r.
**A14**	**CR8/T/D7**	**ICU 11/T/D7**	d.n.r.	d.n.r.	d.n.r.	d.n.r.	d.n.r.
**A15**	**CR10/B/D0**	**ICU 13/B/D0**	1748165	0,31	17586742	0,70	0,45
**A16**	**CR10/B/D7**	**ICU 13/B/D7**	3793331	0,67	18543098	0,74	0,92
**A17**	**CR10/T/D0**	**ICU 13/T/D0**	d.n.r.	d.n.r.	d.n.r.	d.n.r.	d.n.r.
**A18**	**CR10/T/D7**	**ICU 13/T/D7**	d.n.r.	d.n.r.	d.n.r.	d.n.r.	d.n.r.
**A19**	**CR11/B/D0**	**ICU 9/B/D0**	1579218	0,28	20294868	0,81	0,35
**A20**	**CR11/B/D7**	**ICU 9/B/D7**	3763317	0,67	21152021	0,84	0,80
**A21**	**CR11/T/D0**	**ICU 9/T/D0**	d.n.r.	d.n.r.	d.n.r.	d.n.r.	d.n.r.
**A22**	**CR11/T/D7**	**ICU 9/T/D7**	d.n.r.	d.n.r.	d.n.r.	d.n.r.	d.n.r.
	calibrator		4417820	0,79	24177834	0,96	0,82

Lipin 1

	WB ID	Control/Patient ID	protein density	protein normalized to calibrator	GAPDH density	GAPDH normalized to calibrator	protein/GAPDH
**GEL 2/ set B**		**cal. mean**	5028018		**cal. mean**	28899095,5	
	calibrator		4658160	0,93	27854350	0,96	0,96
**B1**	**H4/B**	**Control 12/B**	3105546	0,62	28818080	1,00	0,62
**B2**	**H4/T**	**Control 12/T**	d.n.r.	d.n.r.	d.n.r.	d.n.r.	d.n.r.
**B3**	**H5/B**	**Control 6/B**	2547277	0,51	29889932	1,03	0,49
**B4**	**H5/T**	**Control 6/T**	d.n.r.	d.n.r.	d.n.r.	d.n.r.	d.n.r.
**B5**	**H6/B**	**Control 8/B**	6616238	1,32	23959672	0,83	1,59
**B6**	**H6/T**	**Control 8/T**	d.n.r.	d.n.r.	d.n.r.	d.n.r.	d.n.r.
**B7**	**Sample Y/B**	**Sample Y/B**	excl.	#####	29444064	1,02	########
**B8**	**Sample Y/T**	**Sample Y/T**	excl.	#####	27218925	0,94	########
**B9**	**Sample Z/B**	**Sample Z/B**	excl.	#####	25503469	0,88	########
**B10**	**Sample Z/T**	**Sample Z/T**	excl.	#####	22796640	0,79	########
**B11**	**CR9/B/D0**	**ICU 3/B/D0**	6701326	1,33	26684496	0,92	1,44
**B12**	**CR9/B/D7**	**ICU 3/B/D7**	4381743	0,87	27844674	0,96	0,90
**B13**	**CR9/T/D0**	**ICU 3/T/D0**	d.n.r.	d.n.r.	d.n.r.	d.n.r.	d.n.r.
**B14**	**CR9/T/D7**	**ICU 3/T/D7**	d.n.r.	d.n.r.	d.n.r.	d.n.r.	d.n.r.
**B15**	**CR12/B/D0**	**ICU 20/B/D0**	6431596	1,28	29392896	1,02	1,26
**B16**	**CR12/B/D7**	**ICU 20/B/D7**	4443018	0,88	29492584	1,02	0,87
**B17**	**CR12/T/D0**	**ICU 20/T/D0**	d.n.r.	d.n.r.	d.n.r.	d.n.r.	d.n.r.
**B18**	**CR12/T/D7**	**ICU 20/T/D7**	d.n.r.	d.n.r.	d.n.r.	d.n.r.	d.n.r.
**B19**	**CR13/B/D0**	**ICU 22/B/D0**	1716861	0,34	28194210	0,98	0,35
**B20**	**CR13/B/D7**	**ICU 22/B/D7**	3280513	0,65	25151087	0,87	0,75
**B21**	**CR13/T/D0**	**ICU 22/T/D0**	d.n.r.	d.n.r.	d.n.r.	d.n.r.	d.n.r.
**B22**	**CR13/T/D7**	**ICU 22/T/D7**	d.n.r.	d.n.r.	d.n.r.	d.n.r.	d.n.r.
	calibrator		5397876	1,07	29943841	1,04	1,04

Lipin 1

	WB ID	Control/Patient ID	protein density	protein normalized to calibrator	GAPDH density	GAPDH normalized to calibrator	protein/GAPDH
GEL 3/ set C		cal. mean	6716594,5		cal. mean	34079936	
	calibrator		7445284	1,11	34138962	1,00	1,11
**C1**	**H15/B**	**Control 2/B**	6939606	1,03	29730870	0,87	1,18
**C2**	**H15/T**	**Control 2/T**	d.n.r.	d.n.r.	d.n.r.	d.n.r.	d.n.r.
**C3**	**H16/B**	**Control 14/B**	3478198	0,52	30098518	0,88	0,59
**C4**	**H16/T**	**Control 14/T**	d.n.r.	d.n.r.	d.n.r.	d.n.r.	d.n.r.
**C5**	**H17/B**	**Control 10/B**	6764300	1,01	20994664	0,62	1,63
**C6**	**H17/T**	**Control 10/T**	d.n.r.	d.n.r.	d.n.r.	d.n.r.	d.n.r.
**C7**	**CR3/B/D0**	**ICU 5/B/D0**	5170455	0,77	22660589	0,66	1,16
**C8**	**CR3/B/D7**	**ICU 5/B/D7**	5875144	0,87	18025875	0,53	1,65
**C9**	**CR3/T/D0**	**ICU 5/T/D0**	d.n.r.	d.n.r.	d.n.r.	d.n.r.	d.n.r.
**C10**	**CR3/T/D7**	**ICU 5/T/D7**	d.n.r.	d.n.r.	d.n.r.	d.n.r.	d.n.r.
**C11**	**CR5/B/D0**	**ICU 8/B/D0**	3571394	0,53	19994035	0,59	0,91
**C12**	**CR5/B/D7**	**ICU 8/B/D7**	10724532	1,60	17391984	0,51	3,13
**C13**	**CR5/T/D0**	**ICU 8/T/D0**	d.n.r.	d.n.r.	d.n.r.	d.n.r.	d.n.r.
**C14**	**CR5/T/D7**	**ICU 8/T/D7**	d.n.r.	d.n.r.	d.n.r.	d.n.r.	d.n.r.
**C15**	**CR7/B/D0**	**ICU 6/B/D0**	3761696	0,56	22085112	0,65	0,86
**C16**	**CR7/B/D7**	**ICU 6/B/D7**	6569055	0,98	18388750	0,54	1,81
**C17**	**CD7/T/D0**	**ICU 6/T/D0**	d.n.r.	d.n.r.	d.n.r.	d.n.r.	d.n.r.
**C18**	**CR7/T/D7**	**ICU 6/T/D7**	d.n.r.	d.n.r.	d.n.r.	d.n.r.	d.n.r.
**C19**	**CR20/B/D0**	**ICU 7/B/D0**	1398952	0,21	24852613	0,73	0,29
**C20**	**CR20/B/D7**	**ICU 7/B/D7**	2437908	0,36	25778115	0,76	0,48
**C21**	**CR20/T/D0**	**ICU 7/T/D0**	d.n.r.	d.n.r.	d.n.r.	d.n.r.	d.n.r.
**C22**	**CR20/T/D7**	**ICU 7/T/D7**	d.n.r.	d.n.r.	d.n.r.	d.n.r.	d.n.r.
	calibrator		5987905	0,89	34020910	1,00	0,89

Lipin 1

	WB ID	Control/Patient ID	protein density	protein normalized to calibrator	GAPDH density	GAPDH normalized to calibrator	protein/GAPDH
GEL 4/ set D		cal. mean	5003392,5	cal. mean	35190284		
	calibrator		4916385	0,98	37167787	1,06	0,93
D1	H2/B	Control 4/B	780999	0,16	30949908	0,88	0,18
D2	H2/T	Control 4/T	d.n.r.	d.n.r.	d.n.r.	d.n.r.	d.n.r.
D3	H7/B	Control 13/B	2343264	0,47	31992571	0,91	0,52
D4	H7/T	Control 13/T	d.n.r.	d.n.r.	d.n.r.	d.n.r.	d.n.r.
D5	H8/B	Control 18/B	3833106	0,77	24406066	0,69	1,10
D6	H8/T	Control 18/T	d.n.r.	d.n.r.	d.n.r.	d.n.r.	d.n.r.
D7	H11/B	Control 19/B	5075376	1,01	22372376	0,64	1,60
D8	H11/T	Control 19/T	d.n.r.	d.n.r.	d.n.r.	d.n.r.	d.n.r.
D9	H19/B	Control 5/B	474642	0,09	18229138	0,52	0,18
D10	H19/T	Control 5/T	d.n.r.	d.n.r.	d.n.r.	d.n.r.	d.n.r.
D11	CR2/B/D0	ICU 14/B/D0	4864735	0,97	17679473	0,50	1,94
D12	CR2/B/D7	ICU 14/B/D7	3194955	0,64	18461459	0,52	1,22
D13	CR2/B/D180	ICU 14/B/D180	2140380	0,43	19948365	0,57	0,75
D14	CR2/T/D0	ICU 14/T/D0	d.n.r.	d.n.r.	d.n.r.	d.n.r.	d.n.r.
D15	CR2/T/D7	ICU 14/T/D7	d.n.r.	d.n.r.	d.n.r.	d.n.r.	d.n.r.
D16	CR2/T/D180	ICU 14/T/D180	d.n.r.	d.n.r.	d.n.r.	d.n.r.	d.n.r.
D17	CR17/B/D0	ICU 23/B/D0	2402595	0,48	22814050	0,65	0,74
D18	CR17/B/D7	ICU 23/B/D7	1136898	0,23	24909295	0,71	0,32
D19	CR17/B/D180	ICU 23/B/D180	6528764	1,30	26712850	0,76	1,72
D20	CR17/T/D0	ICU 23/T/D0	d.n.r.	d.n.r.	d.n.r.	d.n.r.	d.n.r.
D21	CR17/T/D7	ICU 23/T/D7	d.n.r.	d.n.r.	d.n.r.	d.n.r.	d.n.r.
D22	CR17/T/D180	ICU 23/T/D180	d.n.r.	d.n.r.	d.n.r.	d.n.r.	d.n.r.
	calibrator		5090400	1,02	33212781	0,94	1,08

Lipin 1

	WB ID	Control/Patient ID	protein density	protein normalized to calibrator	GAPDH density	GAPDH normalized to calibrator	protein/GAPDH
**GEL 5/ set E**		**cal. mean**	6462175	**cal. mean**	13612646,5		
	calibrator		4820140	0,75	13056250	0,96	0,78
**E1**	**H12/B**	**Control 7/B**	2479590	0,38	15730101	1,16	0,33
**E2**	**H12/T**	**Control 7/T**	d.n.r.	d.n.r.	d.n.r.	d.n.r.	d.n.r.
**E3**	**H13/B**	**Control 1/B**	2967750	0,46	17836110	1,31	0,35
**E4**	**H13/T**	**Control 1/T**	d.n.r.	d.n.r.	d.n.r.	d.n.r.	d.n.r.
**E5**	**H18/B**	**Control 11/B**	9304875	1,44	17137798	1,26	1,14
**E6**	**H18/T**	**Control 11/T**	d.n.r.	d.n.r.	d.n.r.	d.n.r.	d.n.r.
**E7**	**CR6/B/D0**	**ICU 21/B/D0**	3829905	0,59	16121115	1,18	0,50
**E8**	**CR6/B/D7**	**ICU 21/B/D7**	5019168	0,78	12428020	0,91	0,85
**E9**	**CR6/T/D0**	**ICU 21/T/D0**	d.n.r.	d.n.r.	d.n.r.	d.n.r.	d.n.r.
**E10**	**CR6/T/D7**	**ICU 21/T/D7**	d.n.r.	d.n.r.	d.n.r.	d.n.r.	d.n.r.
**E11**	**CR14/B/D0**	**ICU 10/B/D0**	3202554	0,50	12644640	0,93	0,53
**E12**	**CR14/B/D7**	**ICU 10/B/D7**	7848360	1,21	13423200	0,99	1,23
**E13**	**CR14/T/D0**	**ICU 10/T/D0**	d.n.r.	d.n.r.	d.n.r.	d.n.r.	d.n.r.
**E14**	**CR14/T/D7**	**ICU 10/T/D7**	d.n.r.	d.n.r.	d.n.r.	d.n.r.	d.n.r.
**E15**	**Sample W/B/D0**	**Sample W/B/D0**	excl.	#####	13286318	0,98	########
**E16**	**Sample W/B/D7**	**Sample W/B/D7**	excl.	#####	14503083	1,07	########
**E17**	**Sample W/T/D0**	**Sample W/T/D0**	excl.	#####	15358237	1,13	########
**E18**	**Sample W/T/D7**	**Sample W/T/D7**	excl.	#####	14918082	1,10	########
**E19**	**CR19/B/D0**	**ICU 15/B/D0**	1985421	0,31	18376065	1,35	0,23
**E20**	**CR19/B/D7**	**ICU 15/B/D7**	2376000	0,37	18750060	1,38	0,27
**E21**	**CR19/T/D0**	**ICU 15/T/D0**	d.n.r.	d.n.r.	d.n.r.	d.n.r.	d.n.r.
**E22**	**CR19/T/D7**	**ICU 15/T/D7**	d.n.r.	d.n.r.	d.n.r.	d.n.r.	d.n.r.
	calibrator		8104210	1,25	14169043	1,04	1,20

Lipin 1

	WB ID	Control/Patient ID	protein density	protein normalized to calibrator	GAPDH density	GAPDH normalized to calibrator	protein/GAPDH
**GEL 6/ set F**		cal. mean	7388423,5		cal. mean 24950732		
	calibrator		8051805	1,09	25509639	1,02	1,07
**F1**	**H9/B**	**Control 9/B**	1032562	0,14	32156010	1,29	0,11
**F2**	**H9/T**	**Control 9/T**	d.n.r.	d.n.r.	d.n.r.	d.n.r.	d.n.r.
**F3**	**H10/B**	**Control 15/B**	9614832	1,30	31970444	1,28	1,02
**F4**	**H10/T**	**Control 15/T**	d.n.r.	d.n.r.	d.n.r.	d.n.r.	d.n.r.
**F5**	**H14/B**	**Control 20/B**	6314670	0,85	25759862	1,03	0,83
**F6**	**H14/T**	**Control 20/T**	d.n.r.	d.n.r.	d.n.r.	d.n.r.	d.n.r.
**F7**	**CR1/B/D0**	**ICU 1/B/D0**	4756444	0,64	24703341	0,99	0,65
**F8**	**CR1/B/D7**	**ICU 1/B/D7**	7245348	0,98	22559436	0,90	1,08
**F9**	**CR1/B/D180**	**ICU 1/B/D180**	6471806	0,88	26013983	1,04	0,84
**F10**	**CR1/T/D0**	**ICU 1/T/D0**	d.n.r.	d.n.r.	d.n.r.	d.n.r.	d.n.r.
**F11**	**CR1/T/D7**	**ICU 1/T/D7**	d.n.r.	d.n.r.	d.n.r.	d.n.r.	d.n.r.
**F12**	**CR1/T/D180**	**ICU 1/T/D180**	d.n.r.	d.n.r.	d.n.r.	d.n.r.	d.n.r.
**F13**	**CR18/B/D0**	**ICU 18/B/D0**	4864244	0,66	24749871	0,99	0,66
**F14**	**CR18/B/D7**	**ICU 18/B/D7**	983928	0,13	21148778	0,85	0,16
**F15**	**CR18/B/D180**	**ICU 18/B/D180**	6265429	0,85	21108311	0,85	1,00
**F16**	**CR18/T/D0**	**ICU 18/T/D0**	d.n.r.	d.n.r.	d.n.r.	d.n.r.	d.n.r.
**F17**	**CR18/T/D7**	**ICU 18/T/D7**	d.n.r.	d.n.r.	d.n.r.	d.n.r.	d.n.r.
**F18**	**CR18/T/D180**	**ICU 18/T/D180**	d.n.r.	d.n.r.	d.n.r.	d.n.r.	d.n.r.
**F19**	**CR15/B/D0**	**ICU 12/B/D0**	4003164	0,54	27652779	1,11	0,49
**F20**	**CR15/B/D7**	**ICU 12/B/D7**	5740672	0,78	28943200	1,16	0,67
**F21**	**CR15/T/D0**	**ICU 12/T/D0**	d.n.r.	d.n.r.	d.n.r.	d.n.r.	d.n.r.
**F22**	**CR15/T/D7**	**ICU 12/T/D7**	d.n.r.	d.n.r.	d.n.r.	d.n.r.	d.n.r.
	calibrator		6725042	0,91	24391825	0,98	0,93

Lipin 1

	WB ID	Control/Patient ID	protein density	protein normalized to calibrator	GAPDH density	GAPDH normalized to calibrator	protein/GAPDH
**GEL 7/ set ICU 16**		**cal. mean**	17547382,5		**cal. mean**	40515874	
	calibrator		19029000	1,08	43007588	1,06	
**E15**	**CR16/B/D0**	**ICU 16/B/D0**	6545527	0,37	37231612	0,92	0,41
**E16**	**CR16/B/D7**	**ICU 16/B/D7**	6247381	0,36	33408164	0,82	0,43
**E17**	**CR16/T/D0**	**ICU 16/T/D0**	d.n.r.	d.n.r.	d.n.r.	d.n.r.	d.n.r.
**E18**	**CR16/T/D7**	**ICU 16/T/D7**	d.n.r.	d.n.r.	d.n.r.	d.n.r.	d.n.r.
	calibrator		16065765	0,92	38024160	0,94	

Fatty acid synthase

	WB ID	Control/Patient ID	protein density	protein normalized to calibrator	GAPDH density	GAPDH normalized to calibrator	protein/GAPDH
GEL 1/ set A		cal. mean	7572334		cal. mean 26252326		
	calibrator		7854088	1,04	27774192	1,06	0,98
**A1**	**H1/B**	**Control 17/B**	4867676	0,64	26549130	1,01	0,64
**A2**	**H1/T**	**Control 17/T**	d.n.r.	d.n.r.	d.n.r.	d.n.r.	d.n.r.
**A3**	**Sample X/B**	**Sample X/B**	excl.	#####	23658300	0,90	########
**A4**	**Sample X/T**	**Sample X/T**	excl.	#####	20212632	0,77	########
**A5**	**H3/B**	**Control 3/B**	4953916	0,65	22203368	0,85	0,77
**A6**	**H3/T**	**Control 3/T**	d.n.r.	d.n.r.	d.n.r.	d.n.r.	d.n.r.
**A7**	**CR4/B/D0**	**ICU 4/B/D0**	5220204	0,69	22318120	0,85	0,81
**A8**	**CR4/B/D7**	**ICU 4/B/D7**	5002800	0,66	21786380	0,83	0,80
**A9**	**CR4/T/D0**	**ICU 4/T/D0**	d.n.r.	d.n.r.	d.n.r.	d.n.r.	d.n.r.
**A10**	**CR4/T/D7**	**ICU 4/T/D7**	d.n.r.	d.n.r.	d.n.r.	d.n.r.	d.n.r.
**A11**	**CR8/B/D0**	**ICU 11/B/D0**	5968644	0,79	24650043	0,94	0,84
**A12**	**CR8/B/D7**	**ICU 11/B/D7**	6194760	0,82	26404414	1,01	0,81
**A13**	**CR8/T/D0**	**ICU 11/T/D0**	d.n.r.	d.n.r.	d.n.r.	d.n.r.	d.n.r.
**A14**	**CR8/T/D7**	**ICU 11/T/D7**	d.n.r.	d.n.r.	d.n.r.	d.n.r.	d.n.r.
**A15**	**CR10/B/D0**	**ICU 13/B/D0**	4507140	0,60	21712328	0,83	0,72
**A16**	**CR10/B/D7**	**ICU 13/B/D7**	5211888	0,69	23581122	0,90	0,77
**A17**	**CR10/T/D0**	**ICU 13/T/D0**	d.n.r.	d.n.r.	d.n.r.	d.n.r.	d.n.r.
**A18**	**CR10/T/D7**	**ICU 13/T/D7**	d.n.r.	d.n.r.	d.n.r.	d.n.r.	d.n.r.
**A19**	**CR11/B/D0**	**ICU 9/B/D0**	4378352	0,58	23880825	0,91	0,64
**A20**	**CR11/B/D7**	**ICU 9/B/D7**	4767092	0,63	21025925	0,80	0,79
**A21**	**CR11/T/D0**	**ICU 9/T/D0**	d.n.r.	d.n.r.	d.n.r.	d.n.r.	d.n.r.
**A22**	**CR11/T/D7**	**ICU 9/T/D7**	d.n.r.	d.n.r.	d.n.r.	d.n.r.	d.n.r.
	calibrator		7290580	0,96	24730460	0,94	1,02

Fatty acid synthase

	WB ID	Control/Patient ID	protein density	protein normalized to calibrator	GAPDH density	GAPDH normalized to calibrator	protein/GAPDH
GEL 2/ set B		cal. mean	7240354		cal. mean 28899095,5		
	calibrator		7942452	1,10	27854350	0,96	1,14
B1	H4/B	Control 12/B	6050057	0,84	28818080	1,00	0,84
B2	H4/T	Control 12/T	d.n.r.	d.n.r.	d.n.r.	d.n.r.	d.n.r.
B3	H5/B	Control 6/B	5421606	0,75	29889932	1,03	0,72
B4	H5/T	Control 6/T	d.n.r.	d.n.r.	d.n.r.	d.n.r.	d.n.r.
B5	H6/B	Control 8/B	4475150	0,62	23959672	0,83	0,75
B6	H6/T	Control 8/T	d.n.r.	d.n.r.	d.n.r.	d.n.r.	d.n.r.
B7	Sample Y/B	Sample Y/B	excl.	#####	29444064	1,02	########
B8	Sample Y/T	Sample Y/T	excl.	#####	27218925	0,94	########
B9	Sample Z/B	Sample Z/B	excl.	#####	25503469	0,88	########
B10	Sample Z/T	Sample Z/T	excl.	#####	22796640	0,79	########
B11	CR9/B/D0	ICU 3/B/D0	3355806	0,46	26684496	0,92	0,50
B12	CR9/B/D7	ICU 3/B/D7	2887364	0,40	27844674	0,96	0,41
B13	CR9/T/D0	ICU 3/T/D0	d.n.r.	d.n.r.	d.n.r.	d.n.r.	d.n.r.
B14	CR9/T/D7	ICU 3/T/D7	d.n.r.	d.n.r.	d.n.r.	d.n.r.	d.n.r.
B15	CR12/B/D0	ICU 20/B/D0	4847777	0,67	29392896	1,02	0,66
B16	CR12/B/D7	ICU 20/B/D7	5014828	0,69	29492584	1,02	0,68
B17	CR12/T/D0	ICU 20/T/D0	d.n.r.	d.n.r.	d.n.r.	d.n.r.	d.n.r.
B18	CR12/T/D7	ICU 20/T/D7	d.n.r.	d.n.r.	d.n.r.	d.n.r.	d.n.r.
B19	CR13/B/D0	ICU 22/B/D0	4528734	0,63	28194210	0,98	0,64
B20	CR13/B/D7	ICU 22/B/D7	5336940	0,74	25151087	0,87	0,85
B21	CR13/T/D0	ICU 22/T/D0	d.n.r.	d.n.r.	d.n.r.	d.n.r.	d.n.r.
B22	CR13/T/D7	ICU 22/T/D7	d.n.r.	d.n.r.	d.n.r.	d.n.r.	d.n.r.
	calibrator		6538256	0,90	29943841	1,04	0,87

Fatty acid synthase

	WB ID	Control/Patient ID	protein density	protein normalized to calibrator	GAPDH density	GAPDH normalized to calibrator	protein/GAPDH
GEL 3/ set C		cal. mean	8350909		cal. mean 22029750		
	calibrator		8439712	1,01	20662800	0,94	1,08
C1	H15/B	Control 2/B	6847928	0,82	20219460	0,92	0,89
C2	H15/T	Control 2/T	d.n.r.	d.n.r.	d.n.r.	d.n.r.	d.n.r.
C3	H16/B	Control 14/B	3803608	0,46	15286365	0,69	0,66
C4	H16/T	Control 14/T	d.n.r.	d.n.r.	d.n.r.	d.n.r.	d.n.r.
C5	H17/B	Control 10/B	3776876	0,45	14141864	0,64	0,70
C6	H17/T	Control 10/T	d.n.r.	d.n.r.	d.n.r.	d.n.r.	d.n.r.
C7	CR3/B/D0	ICU 5/B/D0	3882956	0,46	14270928	0,65	0,72
C8	CR3/B/D7	ICU 5/B/D7	3442795	0,41	13239864	0,60	0,69
C9	CR3/T/D0	ICU 5/T/D0	d.n.r.	d.n.r.	d.n.r.	d.n.r.	d.n.r.
C10	CR3/T/D7	ICU 5/T/D7	d.n.r.	d.n.r.	d.n.r.	d.n.r.	d.n.r.
C11	CR5/B/D0	ICU 8/B/D0	2761804	0,33	18034032	0,82	0,40
C12	CR5/B/D7	ICU 8/B/D7	3843708	0,46	15358512	0,70	0,66
C13	CR5/T/D0	ICU 8/T/D0	d.n.r.	d.n.r.	d.n.r.	d.n.r.	d.n.r.
C14	CR5/T/D7	ICU 8/T/D7	d.n.r.	d.n.r.	d.n.r.	d.n.r.	d.n.r.
C15	CR7/B/D0	ICU 6/B/D0	3891830	0,47	17619684	0,80	0,58
C16	CR7/B/D7	ICU 6/B/D7	4403616	0,53	17926524	0,81	0,65
C17	CD7/T/D0	ICU 6/T/D0	d.n.r.	d.n.r.	d.n.r.	d.n.r.	d.n.r.
C18	CR7/T/D7	ICU 6/T/D7	d.n.r.	d.n.r.	d.n.r.	d.n.r.	d.n.r.
C19	CR20/B/D0	ICU 7/B/D0	3199284	0,38	14741100	0,67	0,57
C20	CR20/B/D7	ICU 7/B/D7	4282738	0,51	15625234	0,71	0,72
C21	CR20/T/D0	ICU 7/T/D0	d.n.r.	d.n.r.	d.n.r.	d.n.r.	d.n.r.
C22	CR20/T/D7	ICU 7/T/D7	d.n.r.	d.n.r.	d.n.r.	d.n.r.	d.n.r.
	calibrator		8262106	0,99	23396700	1,06	0,93

Fatty acid synthase

	WB ID	Control/Patient ID	protein density	protein normalized to calibrator	GAPDH density	GAPDH normalized to calibrator	protein/GAPDH
GEL 4/ set D		cal. mean	16248662,5		cal. mean	27123350	
	calibrator		14190797	0,87	21501050	0,79	1,10
**D1**	**H2/B**	**Control 4/B**	5799285	0,36	15463789	0,57	0,63
**D2**	**H2/T**	**Control 4/T**	d.n.r.	d.n.r.	d.n.r.	d.n.r.	d.n.r.
**D3**	**H7/B**	**Control 13/B**	7191062	0,44	15370470	0,57	0,78
**D4**	**H7/T**	**Control 13/T**	d.n.r.	d.n.r.	d.n.r.	d.n.r.	d.n.r.
**D5**	**H8/B**	**Control 18/B**	5841748	0,36	13315368	0,49	0,73
**D6**	**H8/T**	**Control 18/T**	d.n.r.	d.n.r.	d.n.r.	d.n.r.	d.n.r.
**D7**	**H11/B**	**Control 19/B**	7754104	0,48	15176872	0,56	0,85
**D8**	**H11/T**	**Control 19/T**	d.n.r.	d.n.r.	d.n.r.	d.n.r.	d.n.r.
**D9**	**H19/B**	**Control 5/B**	5544585	0,34	12146022	0,45	0,76
**D10**	**H19/T**	**Control 5/T**	d.n.r.	d.n.r.	d.n.r.	d.n.r.	d.n.r.
**D11**	**CR2/B/D0**	**ICU 14/B/D0**	6563434	0,40	13352832	0,49	0,82
**D12**	**CR2/B/D7**	**ICU 14/B/D7**	5413804	0,33	14137728	0,52	0,64
**D13**	**CR2/B/D180**	**ICU 14/B/D180**	6278781	0,39	11800978	0,44	0,89
**D14**	**CR2/T/D0**	**ICU 14/T/D0**	d.n.r.	d.n.r.	d.n.r.	d.n.r.	d.n.r.
**D15**	**CR2/T/D7**	**ICU 14/T/D7**	d.n.r.	d.n.r.	d.n.r.	d.n.r.	d.n.r.
**D16**	**CR2/T/D180**	**ICU 14/T/D180**	d.n.r.	d.n.r.	d.n.r.	d.n.r.	d.n.r.
**D17**	**CR17/B/D0**	**ICU 23/B/D0**	7807768	0,48	13229508	0,49	0,99
**D18**	**CR17/B/D7**	**ICU 23/B/D7**	7394760	0,46	11261786	0,42	1,10
**D19**	**CR17/B/D180**	**ICU 23/B/D180**	7196112	0,44	10989998	0,41	1,09
**D20**	**CR17/T/D0**	**ICU 23/T/D0**	d.n.r.	d.n.r.	d.n.r.	d.n.r.	d.n.r.
**D21**	**CR17/T/D7**	**ICU 23/T/D7**	d.n.r.	d.n.r.	d.n.r.	d.n.r.	d.n.r.
**D22**	**CR17/T/D180**	**ICU 23/T/D180**	d.n.r.	d.n.r.	d.n.r.	d.n.r.	d.n.r.
	calibrator		18306528	1,13	32745650	1,21	0,93

Fatty acid synthase

WB ID		Control/Patient ID	protein density	protein normalized to calibrator	GAPDH density	GAPDH normalized to calibrator	protein/GAPDH
GEL 5/ set E		cal. mean	16457149,5		cal. mean	13612646,5	
	calibrator		17163612	1,04	13056250	0,96	1,09
E1	H12/B	Control 7/B	d.f.	#####	15730101	1,16	########
E2	H12/T	Control 7/T	d.f.	#####	d.n.r.	d.n.r.	########
E3	H13/B	Control 1/B	d.f.	#####	17836110	1,31	########
E4	H13/T	Control 1/T	d.f.	#####	d.n.r.	d.n.r.	########
E5	H18/B	Control 11/B	8570184	0,52	17137798	1,26	0,41
E6	H18/T	Control 11/T	d.n.r.	d.n.r.	d.n.r.	d.n.r.	d.n.r.
E7	CR6/B/D0	ICU 21/B/D0	9086238	0,55	16121115	1,18	0,47
E8	CR6/B/D7	ICU 21/B/D7	9156258	0,56	12428020	0,91	0,61
E9	CR6/T/D0	ICU 21/T/D0	d.n.r.	d.n.r.	d.n.r.	d.n.r.	d.n.r.
E10	CR6/T/D7	ICU 21/T/D7	d.n.r.	d.n.r.	d.n.r.	d.n.r.	d.n.r.
E11	CR14/B/D0	ICU 10/B/D0	7751099	0,47	12644640	0,93	0,51
E12	CR14/B/D7	ICU 10/B/D7	7514595	0,46	13423200	0,99	0,46
E13	CR14/T/D0	ICU 10/T/D0	d.n.r.	d.n.r.	d.n.r.	d.n.r.	d.n.r.
E14	CR14/T/D7	ICU 10/T/D7	d.n.r.	d.n.r.	d.n.r.	d.n.r.	d.n.r.
E15	Sample W/B/D0	Sample W/B/D0	excl.	#####	13286318	0,98	########
E16	Sample W/B/D7	Sample W/B/D7	excl.	#####	14503083	1,07	########
E17	Sample W/T/D0	Sample W/T/D0	excl.	#####	15358237	1,13	########
E18	Sample W/T/D7	Sample W/T/D7	excl.	#####	14918082	1,10	########
E19	CR19/B/D0	ICU 15/B/D0	d.f.	#####	18376065	1,35	########
E20	CR19/B/D7	ICU 15/B/D7	d.f.	#####	18750060	1,38	########
E21	CR19/T/D0	ICU 15/T/D0	d.f.	#####	d.n.r.	d.n.r.	########
E22	CR19/T/D7	ICU 15/T/D7	d.f.	#####	d.n.r.	d.n.r.	########
	calibrator		15750687	0,96	14169043	1,04	0,92

Fatty acid synthase

	WB ID	Control/Patient ID	protein density	protein normalized to calibrator	GAPDH density	GAPDH normalized to calibrator	protein/GAPDH
GEL 6/ set F		cal. mean	13593580		cal. mean 24950732		
	calibrator		12678424	0,93	25509639	1,02	0,91
F1	H9/B	Control 9/B	8863360	0,65	32156010	1,29	0,51
F2	H9/T	Control 9/T	d.n.r.	d.n.r.	d.n.r.	d.n.r.	d.n.r.
F3	H10/B	Control 15/B	11064284	0,81	31970444	1,28	0,64
F4	H10/T	Control 15/T	d.n.r.	d.n.r.	d.n.r.	d.n.r.	d.n.r.
F5	H14/B	Control 20/B	10562640	0,78	25759862	1,03	0,75
F6	H14/T	Control 20/T	d.n.r.	d.n.r.	d.n.r.	d.n.r.	d.n.r.
F7	CR1/B/D0	ICU 1/B/D0	6343304	0,47	24703341	0,99	0,47
F8	CR1/B/D7	ICU 1/B/D7	8683752	0,64	22559436	0,90	0,71
F9	CR1/B/D180	ICU 1/B/D180	9093568	0,67	26013983	1,04	0,64
F10	CR1/T/D0	ICU 1/T/D0	d.n.r.	d.n.r.	d.n.r.	d.n.r.	d.n.r.
F11	CR1/T/D7	ICU 1/T/D7	d.n.r.	d.n.r.	d.n.r.	d.n.r.	d.n.r.
F12	CR1/T/D180	ICU 1/T/D180	d.n.r.	d.n.r.	d.n.r.	d.n.r.	d.n.r.
F13	CR18/B/D0	ICU 18/B/D0	6780928	0,50	24749871	0,99	0,50
F14	CR18/B/D7	ICU 18/B/D7	7652788	0,56	21148778	0,85	0,66
F15	CR18/B/D180	ICU 18/B/D180	7789936	0,57	21108311	0,85	0,68
F16	CR18/T/D0	ICU 18/T/D0	d.n.r.	d.n.r.	d.n.r.	d.n.r.	d.n.r.
F17	CR18/T/D7	ICU 18/T/D7	d.n.r.	d.n.r.	d.n.r.	d.n.r.	d.n.r.
F18	CR18/T/D180	ICU 18/T/D180	d.n.r.	d.n.r.	d.n.r.	d.n.r.	d.n.r.
F19	CR15/B/D0	ICU 12/B/D0	6177600	0,45	27652779	1,11	0,41
F20	CR15/B/D7	ICU 12/B/D7	9864272	0,73	28943200	1,16	0,63
F21	CR15/T/D0	ICU 12/T/D0	d.n.r.	d.n.r.	d.n.r.	d.n.r.	d.n.r.
F22	CR15/T/D7	ICU 12/T/D7	d.n.r.	d.n.r.	d.n.r.	d.n.r.	d.n.r.
	calibrator		14508736	1,07	24391825	0,98	1,09

Fatty acid synthase

	WB ID	Control/Patient ID	protein density	protein normalized to calibrator	GAPDH density	GAPDH normalized to calibrator	protein/GAPDH
**GEL 7/ set ICU 16**		**cal. mean**	6403522		**cal. mean**	40515874	
	calibrator		5669224	0,89	43007588	1,06	
**E15**	**CR16/B/D0**	**ICU 16/B/D0**	6573308	1,03	37231612	0,92	1,12
**E16**	**CR16/B/D7**	**ICU 16/B/D7**	7208890	1,13	33408164	0,82	1,37
**E17**	**CR16/T/D0**	**ICU 16/T/D0**	d.n.r.	d.n.r.	d.n.r.	d.n.r.	d.n.r.
**E18**	**CR16/T/D7**	**ICU 16/T/D7**	d.n.r.	d.n.r.	d.n.r.	d.n.r.	d.n.r.
	calibrator		7137820	1,11	38024160	0,94	

P-ACLY

	WB ID	Control/Patient ID	protein density	protein normalized to calibrator	GAPDH density	GAPDH normalized to calibrator	protein/GAPDH
GEL 1/ set A		cal. mean	9713542,5	cal. mean	26252326		
	calibrator		9109665	0,94	27774192	1,06	0,89
A1	H1/B	Control 17/B	7832610	0,81	26549130	1,01	0,80
A2	H1/T	Control 17/T	d.n.r.	d.n.r.	d.n.r.	d.n.r.	d.n.r.
A3	Sample X/B	Sample X/B	excl.	#####	23658300	0,90	########
A4	Sample X/T	Sample X/T	excl.	#####	20212632	0,77	########
A5	H3/B	Control 3/B	6025230	0,62	22203368	0,85	0,73
A6	H3/T	Control 3/T	d.n.r.	d.n.r.	d.n.r.	d.n.r.	d.n.r.
A7	CR4/B/D0	ICU 4/B/D0	6011820	0,62	22318120	0,85	0,73
A8	CR4/B/D7	ICU 4/B/D7	5617170	0,58	21786380	0,83	0,70
A9	CR4/T/D0	ICU 4/T/D0	d.n.r.	d.n.r.	d.n.r.	d.n.r.	d.n.r.
A10	CR4/T/D7	ICU 4/T/D7	d.n.r.	d.n.r.	d.n.r.	d.n.r.	d.n.r.
A11	CR8/B/D0	ICU 11/B/D0	8766900	0,90	24650043	0,94	0,96
A12	CR8/B/D7	ICU 11/B/D7	7408350	0,76	26404414	1,01	0,76
A13	CR8/T/D0	ICU 11/T/D0	d.n.r.	d.n.r.	d.n.r.	d.n.r.	d.n.r.
A14	CR8/T/D7	ICU 11/T/D7	d.n.r.	d.n.r.	d.n.r.	d.n.r.	d.n.r.
A15	CR10/B/D0	ICU 13/B/D0	3655440	0,38	21712328	0,83	0,46
A16	CR10/B/D7	ICU 13/B/D7	4927095	0,51	23581122	0,90	0,56
A17	CR10/T/D0	ICU 13/T/D0	d.n.r.	d.n.r.	d.n.r.	d.n.r.	d.n.r.
A18	CR10/T/D7	ICU 13/T/D7	d.n.r.	d.n.r.	d.n.r.	d.n.r.	d.n.r.
A19	CR11/B/D0	ICU 9/B/D0	4401924	0,45	23880825	0,91	0,50
A20	CR11/B/D7	ICU 9/B/D7	3338415	0,34	21025925	0,80	0,43
A21	CR11/T/D0	ICU 9/T/D0	d.n.r.	d.n.r.	d.n.r.	d.n.r.	d.n.r.
A22	CR11/T/D7	ICU 9/T/D7	d.n.r.	d.n.r.	d.n.r.	d.n.r.	d.n.r.
	calibrator		10317420	1,06	24730460	0,94	1,13

P-ACLY

	WB ID	Control/Patient ID	protein density	protein normalized to calibrator	GAPDH density	GAPDH normalized to calibrator	protein/GAPDH
GEL 2/ set B		cal. mean	7870924		cal. mean	28899095,5	
	calibrator		9334136	1,19	27854350	0,96	1,23
**B1**	**H4/B**	**Control 12/B**	9167914	1,16	28818080	1,00	1,17
**B2**	**H4/T**	**Control 12/T**	d.n.r.	d.n.r.	d.n.r.	d.n.r.	d.n.r.
**B3**	**H5/B**	**Control 6/B**	6240768	0,79	29889932	1,03	0,77
**B4**	**H5/T**	**Control 6/T**	d.n.r.	d.n.r.	d.n.r.	d.n.r.	d.n.r.
**B5**	**H6/B**	**Control 8/B**	6113138	0,78	23959672	0,83	0,94
**B6**	**H6/T**	**Control 8/T**	d.n.r.	d.n.r.	d.n.r.	d.n.r.	d.n.r.
**B7**	**Sample Y/B**	**Sample Y/B**	excl.	#####	29444064	1,02	########
**B8**	**Sample Y/T**	**Sample Y/T**	excl.	#####	27218925	0,94	########
**B9**	**Sample Z/B**	**Sample Z/B**	excl.	#####	25503469	0,88	########
**B10**	**Sample Z/T**	**Sample Z/T**	excl.	#####	22796640	0,79	########
**B11**	**CR9/B/D0**	**ICU 3/B/D0**	5501979	0,70	26684496	0,92	0,76
**B12**	**CR9/B/D7**	**ICU 3/B/D7**	7260090	0,92	27844674	0,96	0,96
**B13**	**CR9/T/D0**	**ICU 3/T/D0**	d.n.r.	d.n.r.	d.n.r.	d.n.r.	d.n.r.
**B14**	**CR9/T/D7**	**ICU 3/T/D7**	d.n.r.	d.n.r.	d.n.r.	d.n.r.	d.n.r.
**B15**	**CR12/B/D0**	**ICU 20/B/D0**	7864785	1,00	29392896	1,02	0,98
**B16**	**CR12/B/D7**	**ICU 20/B/D7**	8378937	1,06	29492584	1,02	1,04
**B17**	**CR12/T/D0**	**ICU 20/T/D0**	d.n.r.	d.n.r.	d.n.r.	d.n.r.	d.n.r.
**B18**	**CR12/T/D7**	**ICU 20/T/D7**	d.n.r.	d.n.r.	d.n.r.	d.n.r.	d.n.r.
**B19**	**CR13/B/D0**	**ICU 22/B/D0**	6854256	0,87	28194210	0,98	0,89
**B20**	**CR13/B/D7**	**ICU 22/B/D7**	5650169	0,72	25151087	0,87	0,82
**B21**	**CR13/T/D0**	**ICU 22/T/D0**	d.n.r.	d.n.r.	d.n.r.	d.n.r.	d.n.r.
**B22**	**CR13/T/D7**	**ICU 22/T/D7**	d.n.r.	d.n.r.	d.n.r.	d.n.r.	d.n.r.
	calibrator		6407712	0,81	29943841	1,04	0,79

P-ACLY

	WB ID	Control/Patient ID	protein density	protein normalized to calibrator	GAPDH density	GAPDH normalized to calibrator	protein/GAPDH
**GEL 3/ set C**		cal. mean	18353954,5	cal. mean	22029750		
	calibrator		15920149	0,87	20662800	0,94	0,92
**C1**	**H15/B**	**Control 2/B**	10633891	0,58	20219460	0,92	0,63
**C2**	**H15/T**	**Control 2/T**	d.n.r.	d.n.r.	d.n.r.	d.n.r.	d.n.r.
**C3**	**H16/B**	**Control 14/B**	4900194	0,27	15286365	0,69	0,38
**C4**	**H16/T**	**Control 14/T**	d.n.r.	d.n.r.	d.n.r.	d.n.r.	d.n.r.
**C5**	**H17/B**	**Control 10/B**	7010075	0,38	14141864	0,64	0,59
**C6**	**H17/T**	**Control 10/T**	d.n.r.	d.n.r.	d.n.r.	d.n.r.	d.n.r.
**C7**	**CR3/B/D0**	**ICU 5/B/D0**	5969389	0,33	14270928	0,65	0,50
**C8**	**CR3/B/D7**	**ICU 5/B/D7**	6788840	0,37	13239864	0,60	0,62
**C9**	**CR3/T/D0**	**ICU 5/T/D0**	d.n.r.	d.n.r.	d.n.r.	d.n.r.	d.n.r.
**C10**	**CR3/T/D7**	**ICU 5/T/D7**	d.n.r.	d.n.r.	d.n.r.	d.n.r.	d.n.r.
**C11**	**CR5/B/D0**	**ICU 8/B/D0**	6184819	0,34	18034032	0,82	0,41
**C12**	**CR5/B/D7**	**ICU 8/B/D7**	11138389	0,61	15358512	0,70	0,87
**C13**	**CR5/T/D0**	**ICU 8/T/D0**	d.n.r.	d.n.r.	d.n.r.	d.n.r.	d.n.r.
**C14**	**CR5/T/D7**	**ICU 8/T/D7**	d.n.r.	d.n.r.	d.n.r.	d.n.r.	d.n.r.
**C15**	**CR7/B/D0**	**ICU 6/B/D0**	4595216	0,25	17619684	0,80	0,31
**C16**	**CR7/B/D7**	**ICU 6/B/D7**	6394745	0,35	17926524	0,81	0,43
**C17**	**CD7/T/D0**	**ICU 6/T/D0**	d.n.r.	d.n.r.	d.n.r.	d.n.r.	d.n.r.
**C18**	**CR7/T/D7**	**ICU 6/T/D7**	d.n.r.	d.n.r.	d.n.r.	d.n.r.	d.n.r.
**C19**	**CR20/B/D0**	**ICU 7/B/D0**	2881302	0,16	14741100	0,67	0,23
**C20**	**CR20/B/D7**	**ICU 7/B/D7**	6794989	0,37	15625234	0,71	0,52
**C21**	**CR20/T/D0**	**ICU 7/T/D0**	d.n.r.	d.n.r.	d.n.r.	d.n.r.	d.n.r.
**C22**	**CR20/T/D7**	**ICU 7/T/D7**	d.n.r.	d.n.r.	d.n.r.	d.n.r.	d.n.r.
	calibrator		20787760	1,13	23396700	1,06	1,07

P-ACLY

	WB ID	Control/Patient ID	protein density	protein normalized to calibrator	GAPDH density	GAPDH normalized to calibrator	protein/GAPDH
GEL 4/ set D		cal. mean	12835182,5	cal. mean	27123350		
	calibrator		14358274	1,12	21501050	0,79	1,41
**D1**	**H2/B**	**Control 4/B**	2416834	0,19	15463789	0,57	0,33
**D2**	**H2/T**	**Control 4/T**	d.n.r.	d.n.r.	d.n.r.	d.n.r.	d.n.r.
**D3**	**H7/B**	**Control 13/B**	7152448	0,56	15370470	0,57	0,98
**D4**	**H7/T**	**Control 13/T**	d.n.r.	d.n.r.	d.n.r.	d.n.r.	d.n.r.
**D5**	**H8/B**	**Control 18/B**	5201685	0,41	13315368	0,49	0,83
**D6**	**H8/T**	**Control 18/T**	d.n.r.	d.n.r.	d.n.r.	d.n.r.	d.n.r.
**D7**	**H11/B**	**Control 19/B**	6834082	0,53	15176872	0,56	0,95
**D8**	**H11/T**	**Control 19/T**	d.n.r.	d.n.r.	d.n.r.	d.n.r.	d.n.r.
**D9**	**H19/B**	**Control 5/B**	3408186	0,27	12146022	0,45	0,59
**D10**	**H19/T**	**Control 5/T**	d.n.r.	d.n.r.	d.n.r.	d.n.r.	d.n.r.
**D11**	**CR2/B/D0**	**ICU 14/B/D0**	4699244	0,37	13352832	0,49	0,74
**D12**	**CR2/B/D7**	**ICU 14/B/D7**	3572611	0,28	14137728	0,52	0,53
**D13**	**CR2/B/D180**	**ICU 14/B/D180**	3557224	0,28	11800978	0,44	0,64
**D14**	**CR2/T/D0**	**ICU 14/T/D0**	d.n.r.	d.n.r.	d.n.r.	d.n.r.	d.n.r.
**D15**	**CR2/T/D7**	**ICU 14/T/D7**	d.n.r.	d.n.r.	d.n.r.	d.n.r.	d.n.r.
**D16**	**CR2/T/D180**	**ICU 14/T/D180**	d.n.r.	d.n.r.	d.n.r.	d.n.r.	d.n.r.
**D17**	**CR17/B/D0**	**ICU 23/B/D0**	3517536	0,27	13229508	0,49	0,56
**D18**	**CR17/B/D7**	**ICU 23/B/D7**	3981198	0,31	11261786	0,42	0,75
**D19**	**CR17/B/D180**	**ICU 23/B/D180**	5116810	0,40	10989998	0,41	0,98
**D20**	**CR17/T/D0**	**ICU 23/T/D0**	d.n.r.	d.n.r.	d.n.r.	d.n.r.	d.n.r.
**D21**	**CR17/T/D7**	**ICU 23/T/D7**	d.n.r.	d.n.r.	d.n.r.	d.n.r.	d.n.r.
**D22**	**CR17/T/D180**	**ICU 23/T/D180**	d.n.r.	d.n.r.	d.n.r.	d.n.r.	d.n.r.
	calibrator		11312091	0,88	32745650	1,21	0,73

P-ACLY

	WB ID	Control/Patient ID	protein density	protein normalized to calibrator	GAPDH density	GAPDH normalized to calibrator	protein/GAPDH
GEL 5/ set E		cal. mean	10704106	cal. mean	13612646,5		
	calibrator		9021428	0,84	13056250	0,96	0,88
E1	H12/B	Control 7/B	1431728	0,13	15730101	1,16	0,12
E2	H12/T	Control 7/T	d.n.r.	d.n.r.	d.n.r.	d.n.r.	d.n.r.
E3	H13/B	Control 1/B	1268036	0,12	17836110	1,31	0,09
E4	H13/T	Control 1/T	d.n.r.	d.n.r.	d.n.r.	d.n.r.	d.n.r.
E5	H18/B	Control 11/B	6358304	0,59	17137798	1,26	0,47
E6	H18/T	Control 11/T	d.n.r.	d.n.r.	d.n.r.	d.n.r.	d.n.r.
E7	CR6/B/D0	ICU 21/B/D0	5236502	0,49	16121115	1,18	0,41
E8	CR6/B/D7	ICU 21/B/D7	5252188	0,49	12428020	0,91	0,54
E9	CR6/T/D0	ICU 21/T/D0	d.n.r.	d.n.r.	d.n.r.	d.n.r.	d.n.r.
E10	CR6/T/D7	ICU 21/T/D7	d.n.r.	d.n.r.	d.n.r.	d.n.r.	d.n.r.
E11	CR14/B/D0	ICU 10/B/D0	11309100	1,06	12644640	0,93	1,14
E12	CR14/B/D7	ICU 10/B/D7	13765684	1,29	13423200	0,99	1,30
E13	CR14/T/D0	ICU 10/T/D0	d.n.r.	d.n.r.	d.n.r.	d.n.r.	d.n.r.
E14	CR14/T/D7	ICU 10/T/D7	d.n.r.	d.n.r.	d.n.r.	d.n.r.	d.n.r.
E15	Sample W/B/D0	Sample W/B/D0	excl.	#####	13286318	0,98	########
E16	Sample W/B/D7	Sample W/B/D7	excl.	#####	14503083	1,07	########
E17	Sample W/T/D0	Sample W/T/D0	excl.	#####	15358237	1,13	########
E18	Sample W/T/D7	Sample W/T/D7	excl.	#####	14918082	1,10	########
E19	CR19/B/D0	ICU 15/B/D0	5350444	0,50	18376065	1,35	0,37
E20	CR19/B/D7	ICU 15/B/D7	5886630	0,55	18750060	1,38	0,40
E21	CR19/T/D0	ICU 15/T/D0	d.n.r.	d.n.r.	d.n.r.	d.n.r.	d.n.r.
E22	CR19/T/D7	ICU 15/T/D7	d.n.r.	d.n.r.	d.n.r.	d.n.r.	d.n.r.
	calibrator		12386784	1,16	14169043	1,04	1,11

P-ACLY

	WB ID	Control/Patient ID	protein density	protein normalized to calibrator	GAPDH density	GAPDH normalized to calibrator	protein/GAPDH
GEL 6/ set F		cal. mean	16038867,5		cal. mean 24950732		
	calibrator		14696712	0,92	25509639	1,02	0,90
F1	H9/B	Control 9/B	6472752	0,40	32156010	1,29	0,31
F2	H9/T	Control 9/T	d.n.r.	d.n.r.	d.n.r.	d.n.r.	d.n.r.
F3	H10/B	Control 15/B	9608445	0,60	31970444	1,28	0,47
F4	H10/T	Control 15/T	d.n.r.	d.n.r.	d.n.r.	d.n.r.	d.n.r.
F5	H14/B	Control 20/B	8116636	0,51	25759862	1,03	0,49
F6	H14/T	Control 20/T	d.n.r.	d.n.r.	d.n.r.	d.n.r.	d.n.r.
F7	CR1/B/D0	ICU 1/B/D0	2297435	0,14	24703341	0,99	0,14
F8	CR1/B/D7	ICU 1/B/D7	7470650	0,47	22559436	0,90	0,52
F9	CR1/B/D180	ICU 1/B/D180	8836517	0,55	26013983	1,04	0,53
F10	CR1/T/D0	ICU 1/T/D0	d.n.r.	d.n.r.	d.n.r.	d.n.r.	d.n.r.
F11	CR1/T/D7	ICU 1/T/D7	d.n.r.	d.n.r.	d.n.r.	d.n.r.	d.n.r.
F12	CR1/T/D180	ICU 1/T/D180	d.n.r.	d.n.r.	d.n.r.	d.n.r.	d.n.r.
F13	CR18/B/D0	ICU 18/B/D0	4034045	0,25	24749871	0,99	0,25
F14	CR18/B/D7	ICU 18/B/D7	4231316	0,26	21148778	0,85	0,31
F15	CR18/B/D180	ICU 18/B/D180	12209190	0,76	21108311	0,85	0,90
F16	CR18/T/D0	ICU 18/T/D0	d.n.r.	d.n.r.	d.n.r.	d.n.r.	d.n.r.
F17	CR18/T/D7	ICU 18/T/D7	d.n.r.	d.n.r.	d.n.r.	d.n.r.	d.n.r.
F18	CR18/T/D180	ICU 18/T/D180	d.n.r.	d.n.r.	d.n.r.	d.n.r.	d.n.r.
F19	CR15/B/D0	ICU 12/B/D0	9540248	0,59	27652779	1,11	0,54
F20	CR15/B/D7	ICU 12/B/D7	5780017	0,36	28943200	1,16	0,31
F21	CR15/T/D0	ICU 12/T/D0	d.n.r.	d.n.r.	d.n.r.	d.n.r.	d.n.r.
F22	CR15/T/D7	ICU 12/T/D7	d.n.r.	d.n.r.	d.n.r.	d.n.r.	d.n.r.
	calibrator		17381023	1,08	24391825	0,98	1,11

P-ACLY

	WB ID	Control/Patient ID	protein density	protein normalized to calibrator	GAPDH density	GAPDH normalized to calibrator	protein/GAPDH
GEL 7/ set ICU 16		cal. mean	5863873	cal. mean	40515874		
	calibrator		5884182	1,00	43007588	1,06	
**E15**	**CR16/B/D0**	**ICU 16/B/D0**	4460256	0,76	37231612	0,92	0,83
**E16**	**CR16/B/D7**	**ICU 16/B/D7**	3760868	0,64	33408164	0,82	0,78
**E17**	**CR16/T/D0**	**ICU 16/T/D0**	d.n.r.	d.n.r.	d.n.r.	d.n.r.	d.n.r.
**E18**	**CR16/T/D7**	**ICU 16/T/D7**	d.n.r.	d.n.r.	d.n.r.	d.n.r.	d.n.r.
	calibrator		5843564	1,00	38024160	0,94	

ACLY

	WB ID	Control/Patient ID	protein density	protein normalized to calibrator	GAPDH density	GAPDH normalized to calibrator	protein/GAPDH
GEL 1/ set A		cal. mean	5194952	cal. mean	32397000		
	calibrator		3387750	0,65	34486272	1,06	0,61
A1	H1/B	Control 17/B	4945293	0,95	36056208	1,11	0,86
A2	H1/T	Control 17/T	d.n.r.	d.n.r.	d.n.r.	d.n.r.	d.n.r.
A3	Sample X/B	Sample X/B	excl.	#####	27900540	0,86	########
A4	Sample X/T	Sample X/T	excl.	#####	28724784	0,89	########
A5	H3/B	Control 3/B	2473812	0,48	28793205	0,89	0,54
A6	H3/T	Control 3/T	d.n.r.	d.n.r.	d.n.r.	d.n.r.	d.n.r.
A7	CR4/B/D0	ICU 4/B/D0	5037407	0,97	24152530	0,75	1,30
A8	CR4/B/D7	ICU 4/B/D7	3137814	0,60	22376194	0,69	0,87
A9	CR4/T/D0	ICU 4/T/D0	d.n.r.	d.n.r.	d.n.r.	d.n.r.	d.n.r.
A10	CR4/T/D7	ICU 4/T/D7	d.n.r.	d.n.r.	d.n.r.	d.n.r.	d.n.r.
A11	CR8/B/D0	ICU 11/B/D0	9684073	1,86	23807098	0,73	2,54
A12	CR8/B/D7	ICU 11/B/D7	11239345	2,16	24407424	0,75	2,87
A13	CR8/T/D0	ICU 11/T/D0	d.n.r.	d.n.r.	d.n.r.	d.n.r.	d.n.r.
A14	CR8/T/D7	ICU 11/T/D7	d.n.r.	d.n.r.	d.n.r.	d.n.r.	d.n.r.
A15	CR10/B/D0	ICU 13/B/D0	2203245	0,42	23284992	0,72	0,59
A16	CR10/B/D7	ICU 13/B/D7	3645226	0,70	23631488	0,73	0,96
A17	CR10/T/D0	ICU 13/T/D0	d.n.r.	d.n.r.	d.n.r.	d.n.r.	d.n.r.
A18	CR10/T/D7	ICU 13/T/D7	d.n.r.	d.n.r.	d.n.r.	d.n.r.	d.n.r.
A19	CR11/B/D0	ICU 9/B/D0	1725120	0,33	33965550	1,05	0,32
A20	CR11/B/D7	ICU 9/B/D7	1783744	0,34	32601396	1,01	0,34
A21	CR11/T/D0	ICU 9/T/D0	d.n.r.	d.n.r.	d.n.r.	d.n.r.	d.n.r.
A22	CR11/T/D7	ICU 9/T/D7	d.n.r.	d.n.r.	d.n.r.	d.n.r.	d.n.r.
	calibrator		7002154	1,35	30307728	0,94	1,44

ACLY

	WB ID	Control/Patient ID	protein density	protein normalized to calibrator	GAPDH density	GAPDH normalized to calibrator	protein/GAPDH
GEL 2/ set B		cal. mean	5192216		cal. mean	27865678,5	
	calibrator		5467104	1,05	30163392	1,08	0,97
**B1**	**H4/B**	**Control 12/B**	7437985	1,43	27105885	0,97	1,47
**B2**	**H4/T**	**Control 12/T**	d.n.r.	d.n.r.	d.n.r.	d.n.r.	d.n.r.
**B3**	**H5/B**	**Control 6/B**	2197800	0,42	28410290	1,02	0,42
**B4**	**H5/T**	**Control 6/T**	d.n.r.	d.n.r.	d.n.r.	d.n.r.	d.n.r.
**B5**	**H6/B**	**Control 8/B**	3086349	0,59	20371736	0,73	0,81
**B6**	**H6/T**	**Control 8/T**	d.n.r.	d.n.r.	d.n.r.	d.n.r.	d.n.r.
**B7**	**Sample Y/B**	**Sample Y/B**	excl.	#####	22090275	0,79	########
**B8**	**Sample Y/T**	**Sample Y/T**	excl.	#####	19395114	0,70	########
**B9**	**Sample Z/B**	**Sample Z/B**	excl.	#####	20887034	0,75	########
**B10**	**Sample Z/T**	**Sample Z/T**	excl.	#####	18124046	0,65	########
**B11**	**CR9/B/D0**	**ICU 3/B/D0**	3617246	0,70	21057888	0,76	0,92
**B12**	**CR9/B/D7**	**ICU 3/B/D7**	4833000	0,93	20873640	0,75	1,24
**B13**	**CR9/T/D0**	**ICU 3/T/D0**	d.n.r.	d.n.r.	d.n.r.	d.n.r.	d.n.r.
**B14**	**CR9/T/D7**	**ICU 3/T/D7**	d.n.r.	d.n.r.	d.n.r.	d.n.r.	d.n.r.
**B15**	**CR12/B/D0**	**ICU 20/B/D0**	4519845	0,87	26873664	0,96	0,90
**B16**	**CR12/B/D7**	**ICU 20/B/D7**	5448193	1,05	23672428	0,85	1,24
**B17**	**CR12/T/D0**	**ICU 20/T/D0**	d.n.r.	d.n.r.	d.n.r.	d.n.r.	d.n.r.
**B18**	**CR12/T/D7**	**ICU 20/T/D7**	d.n.r.	d.n.r.	d.n.r.	d.n.r.	d.n.r.
**B19**	**CR13/B/D0**	**ICU 22/B/D0**	4276890	0,82	22632840	0,81	1,01
**B20**	**CR13/B/D7**	**ICU 22/B/D7**	d.f.	#####	20968515	0,75	########
**B21**	**CR13/T/D0**	**ICU 22/T/D0**	d.f.	#####	d.n.r.	d.n.r.	########
**B22**	**CR13/T/D7**	**ICU 22/T/D7**	d.f.	#####	d.n.r.	d.n.r.	########
	calibrator		4917328	0,95	25567965	0,92	1,03

ACLY

	WB ID	Control/Patient ID	protein density	protein normalized to calibrator	GAPDH density	GAPDH normalized to calibrator	protein/GAPDH
GEL 3/ set C		cal. mean	5477333		cal. mean	33101325,5	
	calibrator		5443916	0,99	39634150	1,20	0,83
C1	H15/B	Control 2/B	6554208	1,20	41027124	1,24	0,97
C2	H15/T	Control 2/T	d.n.r.	d.n.r.	d.n.r.	d.n.r.	d.n.r.
C3	H16/B	Control 14/B	3664314	0,67	36119890	1,09	0,61
C4	H16/T	Control 14/T	d.n.r.	d.n.r.	d.n.r.	d.n.r.	d.n.r.
C5	H17/B	Control 10/B	6015742	1,10	32155610	0,97	1,13
C6	H17/T	Control 10/T	d.n.r.	d.n.r.	d.n.r.	d.n.r.	d.n.r.
C7	CR3/B/D0	ICU 5/B/D0	2926272	0,53	31662000	0,96	0,56
C8	CR3/B/D7	ICU 5/B/D7	2895705	0,53	32580420	0,98	0,54
C9	CR3/T/D0	ICU 5/T/D0	d.n.r.	d.n.r.	d.n.r.	d.n.r.	d.n.r.
C10	CR3/T/D7	ICU 5/T/D7	d.n.r.	d.n.r.	d.n.r.	d.n.r.	d.n.r.
C11	CR5/B/D0	ICU 8/B/D0	1331088	0,24	35399622	1,07	0,23
C12	CR5/B/D7	ICU 8/B/D7	2021404	0,37	28886670	0,87	0,42
C13	CR5/T/D0	ICU 8/T/D0	d.n.r.	d.n.r.	d.n.r.	d.n.r.	d.n.r.
C14	CR5/T/D7	ICU 8/T/D7	d.n.r.	d.n.r.	d.n.r.	d.n.r.	d.n.r.
C15	CR7/B/D0	ICU 6/B/D0	209367	0,04	25648220	0,77	0,05
C16	CR7/B/D7	ICU 6/B/D7	516810	0,09	24801885	0,75	0,13
C17	CD7/T/D0	ICU 6/T/D0	d.n.r.	d.n.r.	d.n.r.	d.n.r.	d.n.r.
C18	CR7/T/D7	ICU 6/T/D7	d.n.r.	d.n.r.	d.n.r.	d.n.r.	d.n.r.
C19	CR20/B/D0	ICU 7/B/D0	468424	0,09	23268402	0,70	0,12
C20	CR20/B/D7	ICU 7/B/D7	1225900	0,22	26161994	0,79	0,28
C21	CR20/T/D0	ICU 7/T/D0	d.n.r.	d.n.r.	d.n.r.	d.n.r.	d.n.r.
C22	CR20/T/D7	ICU 7/T/D7	d.n.r.	d.n.r.	d.n.r.	d.n.r.	d.n.r.
	calibrator		5510750	1,01	26568501	0,80	1,25

ACLY

	WB ID	Control/Patient ID	protein density	protein normalized to calibrator	GAPDH density	GAPDH normalized to calibrator	protein/GAPDH
GEL 4/ set D		cal. mean	5435325		cal. mean 37613053,5		
	calibrator		4352572	0,80	35832900	0,95	0,84
D1	H2/B	Control 4/B	69930	0,01	33954570	0,90	0,01
D2	H2/T	Control 4/T	d.n.r.	d.n.r.	d.n.r.	d.n.r.	d.n.r.
D3	H7/B	Control 13/B	4406524	0,81	37204616	0,99	0,82
D4	H7/T	Control 13/T	d.n.r.	d.n.r.	d.n.r.	d.n.r.	d.n.r.
D5	H8/B	Control 18/B	7407610	1,36	27370416	0,73	1,87
D6	H8/T	Control 18/T	d.n.r.	d.n.r.	d.n.r.	d.n.r.	d.n.r.
D7	H11/B	Control 19/B	6382086	1,17	33493056	0,89	1,32
D8	H11/T	Control 19/T	d.n.r.	d.n.r.	d.n.r.	d.n.r.	d.n.r.
D9	H19/B	Control 5/B	3019946	0,56	29291952	0,78	0,71
D10	H19/T	Control 5/T	d.n.r.	d.n.r.	d.n.r.	d.n.r.	d.n.r.
D11	CR2/B/D0	ICU 14/B/D0	4832530	0,89	26753646	0,71	1,25
D12	CR2/B/D7	ICU 14/B/D7	4020354	0,74	28843383	0,77	0,96
D13	CR2/B/D180	ICU 14/B/D180	3108674	0,57	26981280	0,72	0,80
D14	CR2/T/D0	ICU 14/T/D0	d.n.r.	d.n.r.	d.n.r.	d.n.r.	d.n.r.
D15	CR2/T/D7	ICU 14/T/D7	d.n.r.	d.n.r.	d.n.r.	d.n.r.	d.n.r.
D16	CR2/T/D180	ICU 14/T/D180	d.n.r.	d.n.r.	d.n.r.	d.n.r.	d.n.r.
D17	CR17/B/D0	ICU 23/B/D0	7653903	1,41	28709933	0,76	1,84
D18	CR17/B/D7	ICU 23/B/D7	4378044	0,81	28537335	0,76	1,06
D19	CR17/B/D180	ICU 23/B/D180	5081850	0,93	29401155	0,78	1,20
D20	CR17/T/D0	ICU 23/T/D0	d.n.r.	d.n.r.	d.n.r.	d.n.r.	d.n.r.
D21	CR17/T/D7	ICU 23/T/D7	d.n.r.	d.n.r.	d.n.r.	d.n.r.	d.n.r.
D22	CR17/T/D180	ICU 23/T/D180	d.n.r.	d.n.r.	d.n.r.	d.n.r.	d.n.r.
	calibrator		6518078	1,20	39393207	1,05	1,15

ACLY

	WB ID	Control/Patient ID	protein density	protein normalized to calibrator	GAPDH density	GAPDH normalized to calibrator	protein/GAPDH
**GEL 5/ set E**		cal. mean	5039923		cal. mean	33459356	
	calibrator		3320096	0,66	32367400	0,97	0,68
**E1**	**H12/B**	**Control 7/B**	1533465	0,30	33789330	1,01	0,30
**E2**	**H12/T**	**Control 7/T**	d.n.r.	d.n.r.	d.n.r.	d.n.r.	d.n.r.
**E3**	**H13/B**	**Control 1/B**	4064974	0,81	32255844	0,96	0,84
**E4**	**H13/T**	**Control 1/T**	d.n.r.	d.n.r.	d.n.r.	d.n.r.	d.n.r.
**E5**	**H18/B**	**Control 11/B**	9602429	1,91	27424188	0,82	2,32
**E6**	**H18/T**	**Control 11/T**	d.n.r.	d.n.r.	d.n.r.	d.n.r.	d.n.r.
**E7**	**CR6/B/D0**	**ICU 21/B/D0**	5665320	1,12	29010256	0,87	1,30
**E8**	**CR6/B/D7**	**ICU 21/B/D7**	4850013	0,96	22604584	0,68	1,42
**E9**	**CR6/T/D0**	**ICU 21/T/D0**	d.n.r.	d.n.r.	d.n.r.	d.n.r.	d.n.r.
**E10**	**CR6/T/D7**	**ICU 21/T/D7**	d.n.r.	d.n.r.	d.n.r.	d.n.r.	d.n.r.
**E11**	**CR14/B/D0**	**ICU 10/B/D0**	3270876	0,65	24246002	0,72	0,90
**E12**	**CR14/B/D7**	**ICU 10/B/D7**	4478220	0,89	26351913	0,79	1,13
**E13**	**CR14/T/D0**	**ICU 10/T/D0**	d.n.r.	d.n.r.	d.n.r.	d.n.r.	d.n.r.
**E14**	**CR14/T/D7**	**ICU 10/T/D7**	d.n.r.	d.n.r.	d.n.r.	d.n.r.	d.n.r.
**E15**	**CR16/B/D0**	**ICU 16/B/D0**	4027584	0,80	29617830	0,89	0,90
**E16**	**CR16/B/D7**	**ICU 16/B/D7**	3735952	0,74	26893768	0,80	0,92
**E17**	**CR16/T/D0**	**ICU 16/T/D0**	d.n.r.	d.n.r.	d.n.r.	d.n.r.	d.n.r.
**E18**	**CR16/T/D7**	**ICU 16/T/D7**	d.n.r.	d.n.r.	d.n.r.	d.n.r.	d.n.r.
**E19**	**CR19/B/D0**	**ICU 15/B/D0**	2686005	0,53	32525266	0,97	0,55
**E20**	**CR19/B/D7**	**ICU 15/B/D7**	3317625	0,66	38332904	1,15	0,57
**E21**	**CR19/T/D0**	**ICU 15/T/D0**	d.n.r.	d.n.r.	d.n.r.	d.n.r.	d.n.r.
**E22**	**CR19/T/D7**	**ICU 15/T/D7**	d.n.r.	d.n.r.	d.n.r.	d.n.r.	d.n.r.
	calibrator		6759750	1,34	34551312	1,03	1,30

ACLY

	WB ID	Control/Patient ID	protein density	protein normalized to calibrator	GAPDH density	GAPDH normalized to calibrator	protein/GAPDH
GEL 6/ set F		cal. mean	###########		cal. mean	37954347	
	calibrator		d.f.	#####	26550450	0,70	########
F1	H9/B	Control 9/B	d.f.	#####	30236175	0,80	########
F2	H9/T	Control 9/T	d.f.	#####	d.n.r.	d.n.r.	########
F3	H10/B	Control 15/B	d.f.	#####	28345900	0,75	########
F4	H10/T	Control 15/T	d.f.	#####	d.n.r.	d.n.r.	########
F5	H14/B	Control 20/B	d.f.	#####	27331912	0,72	########
F6	H14/T	Control 20/T	d.f.	#####	d.n.r.	d.n.r.	########
F7	CR1/B/D0	ICU 1/B/D0	d.f.	#####	26053802	0,69	########
F8	CR1/B/D7	ICU 1/B/D7	d.f.	#####	27329985	0,72	########
F9	CR1/B/D180	ICU 1/B/D180	d.f.	#####	29696880	0,78	########
F10	CR1/T/D0	ICU 1/T/D0	d.f.	#####	d.n.r.	d.n.r.	########
F11	CR1/T/D7	ICU 1/T/D7	d.f.	#####	d.n.r.	d.n.r.	########
F12	CR1/T/D180	ICU 1/T/D180	d.f.	#####	d.n.r.	d.n.r.	########
F13	CR18/B/D0	ICU 18/B/D0	d.f.	#####	31147879	0,82	########
F14	CR18/B/D7	ICU 18/B/D7	d.f.	#####	34315678	0,90	########
F15	CR18/B/D180	ICU 18/B/D180	d.f.	#####	36800640	0,97	########
F16	CR18/T/D0	ICU 18/T/D0	d.f.	#####	d.n.r.	d.n.r.	########
F17	CR18/T/D7	ICU 18/T/D7	d.f.	#####	d.n.r.	d.n.r.	########
F18	CR18/T/D180	ICU 18/T/D180	d.f.	#####	d.n.r.	d.n.r.	########
F19	CR15/B/D0	ICU 12/B/D0	d.f.	#####	45890106	1,21	########
F20	CR15/B/D7	ICU 12/B/D7	d.f.	#####	39184088	1,03	########
F21	CR15/T/D0	ICU 12/T/D0	d.f.	#####	d.n.r.	d.n.r.	########
F22	CR15/T/D7	ICU 12/T/D7	d.f.	#####	d.n.r.	d.n.r.	########
	calibrator			#####	49358244	1,30	########
