# Supplementary material for: Critical illness impairs coordinated myogenesis: a prospective longitudinal study of skeletal muscle progenitor cell biology using serial muscle biopsies (SATELLITE study)
Source: Crit Care. 2026 Jun 16;30:443. doi: 10.1186/s13054-026-06131-5 (PMC13501688; doi:10.1186/s13054-026-06131-5)

Western blot raw data

Krajčová et al.

not intended for publication – for reviewers only !!!
(includes data not related to the reviewed paper)

Membrane 1

set A

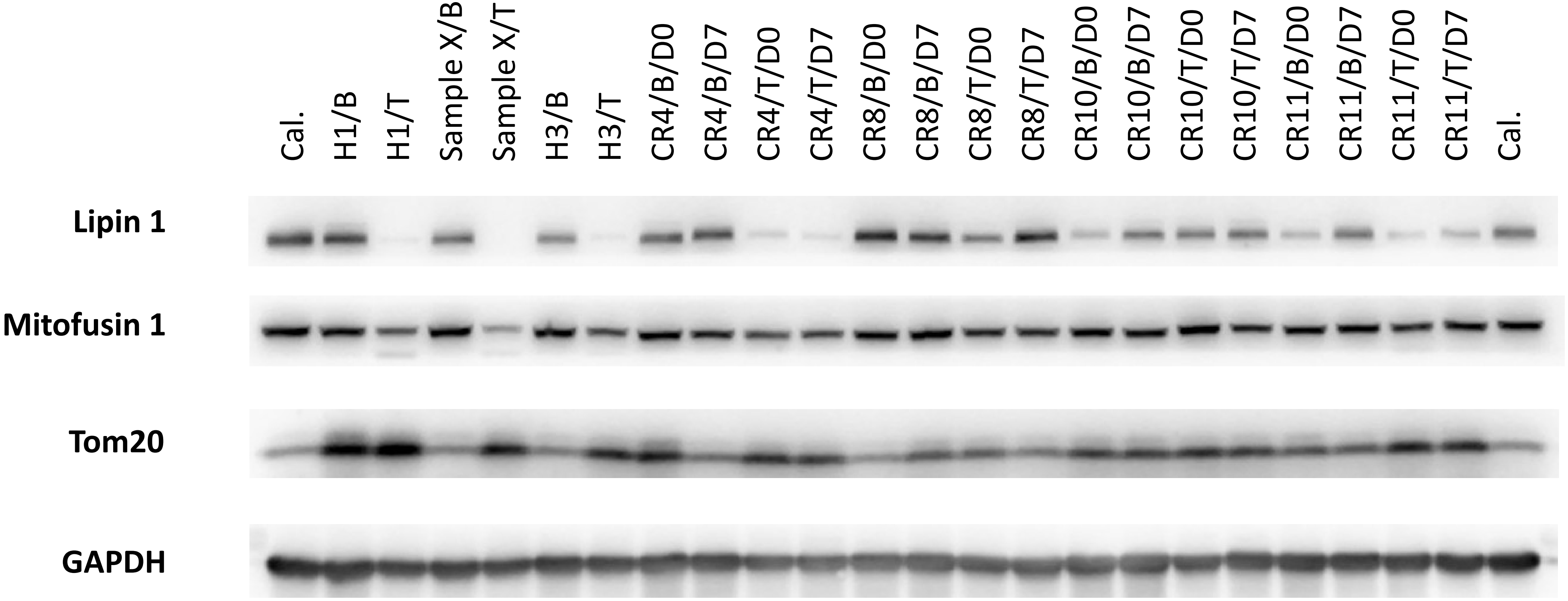

Membrane 2

set A

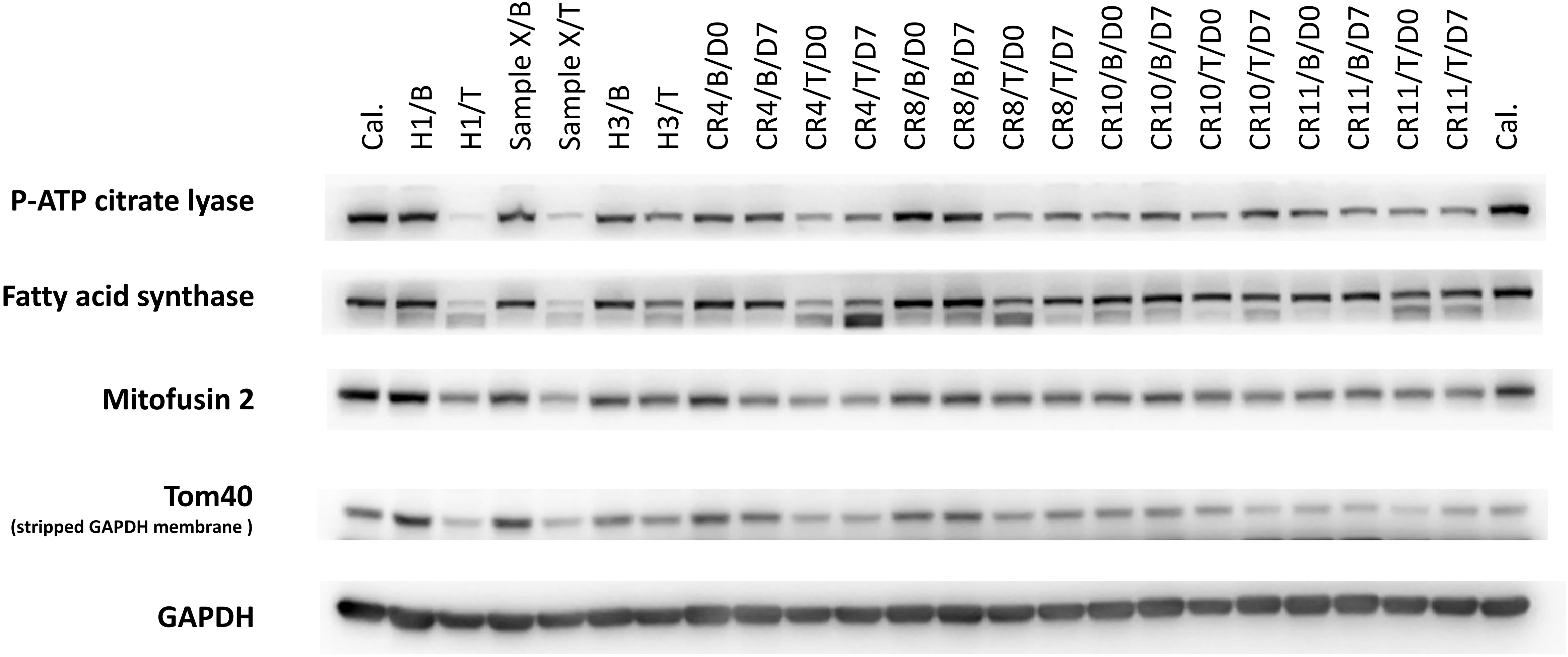

Membrane 3

set A

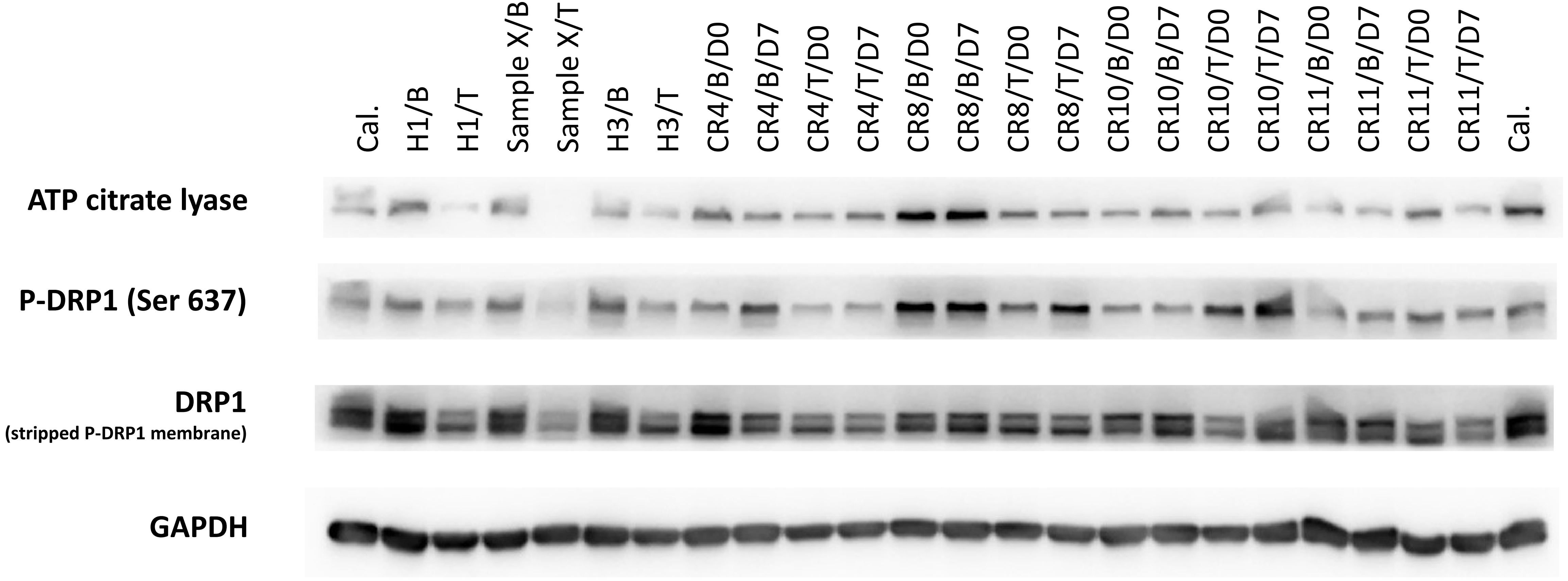

Membrane 4

set A

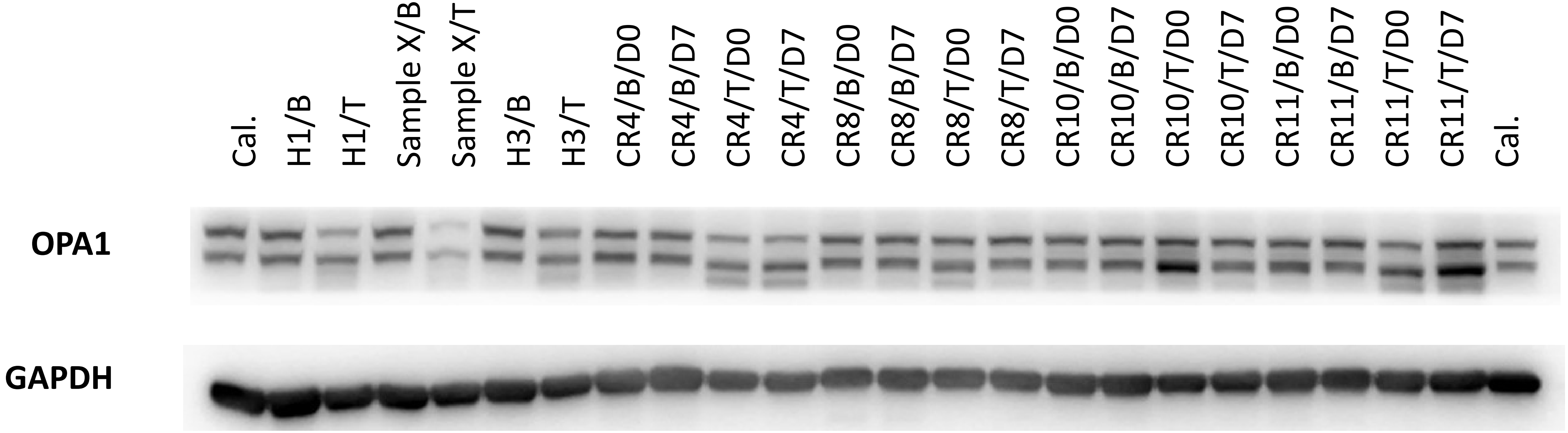

Membrane 5

set A

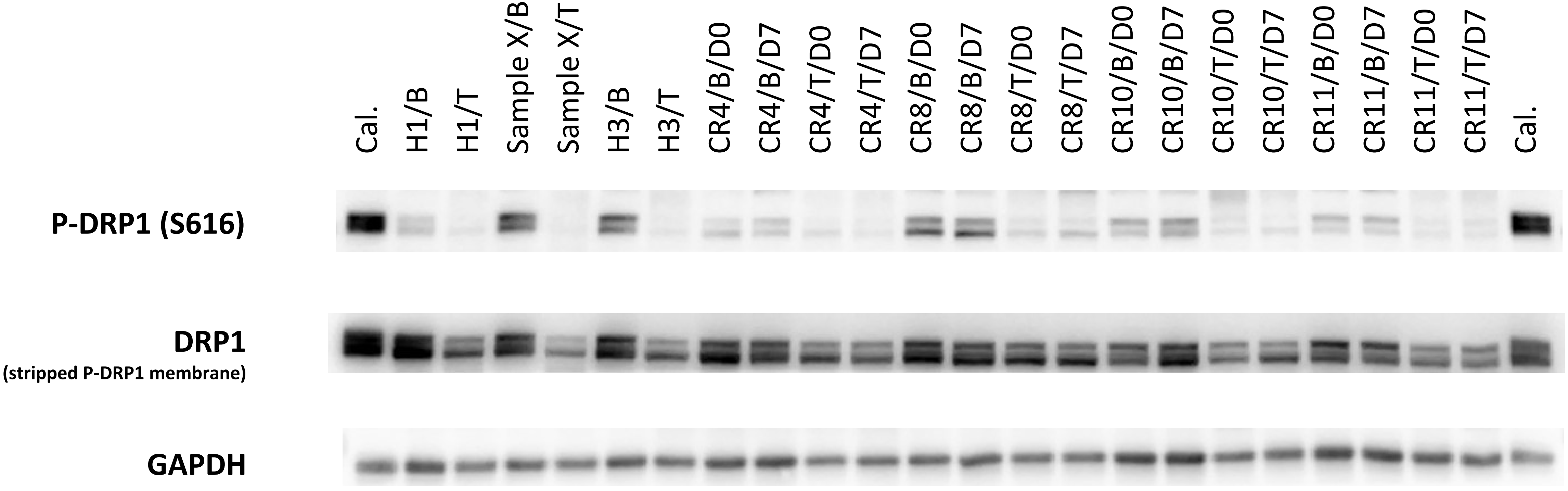

Membrane 1

set B

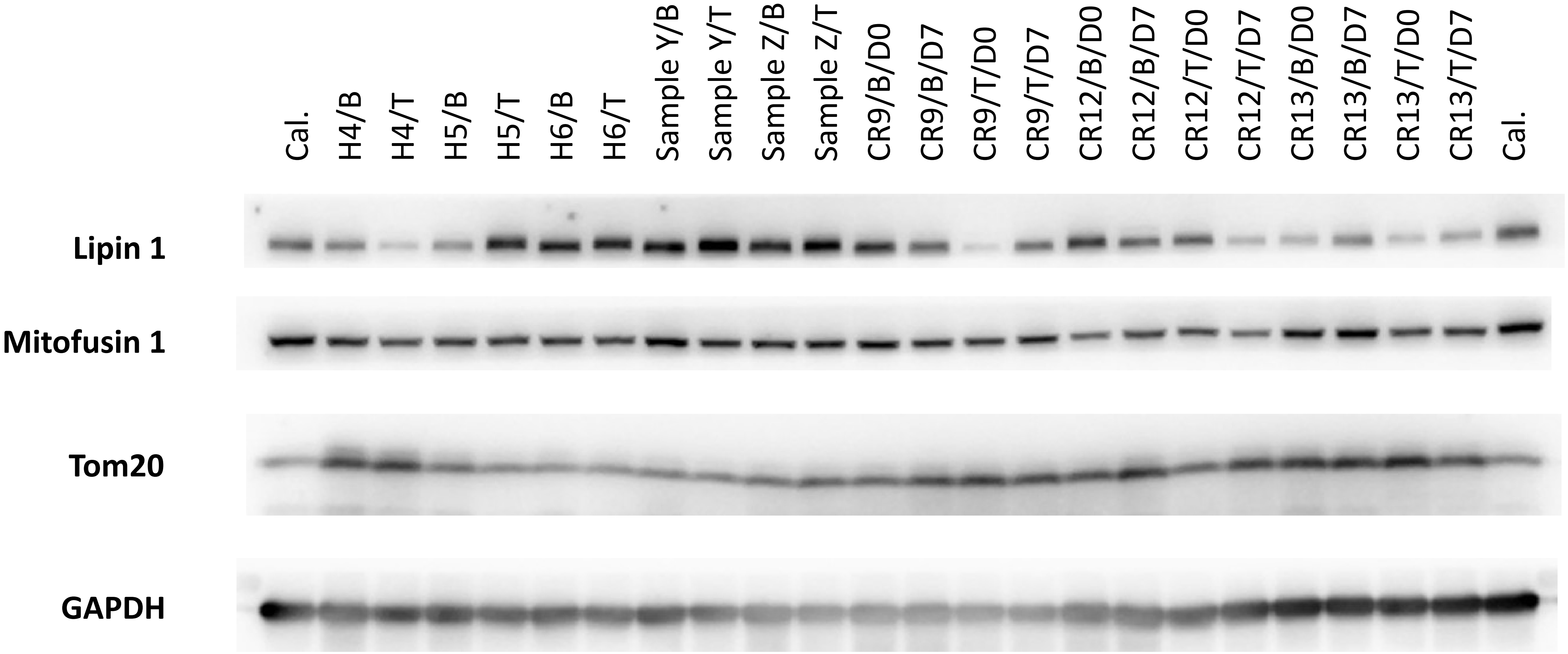

Membrane 2

set B

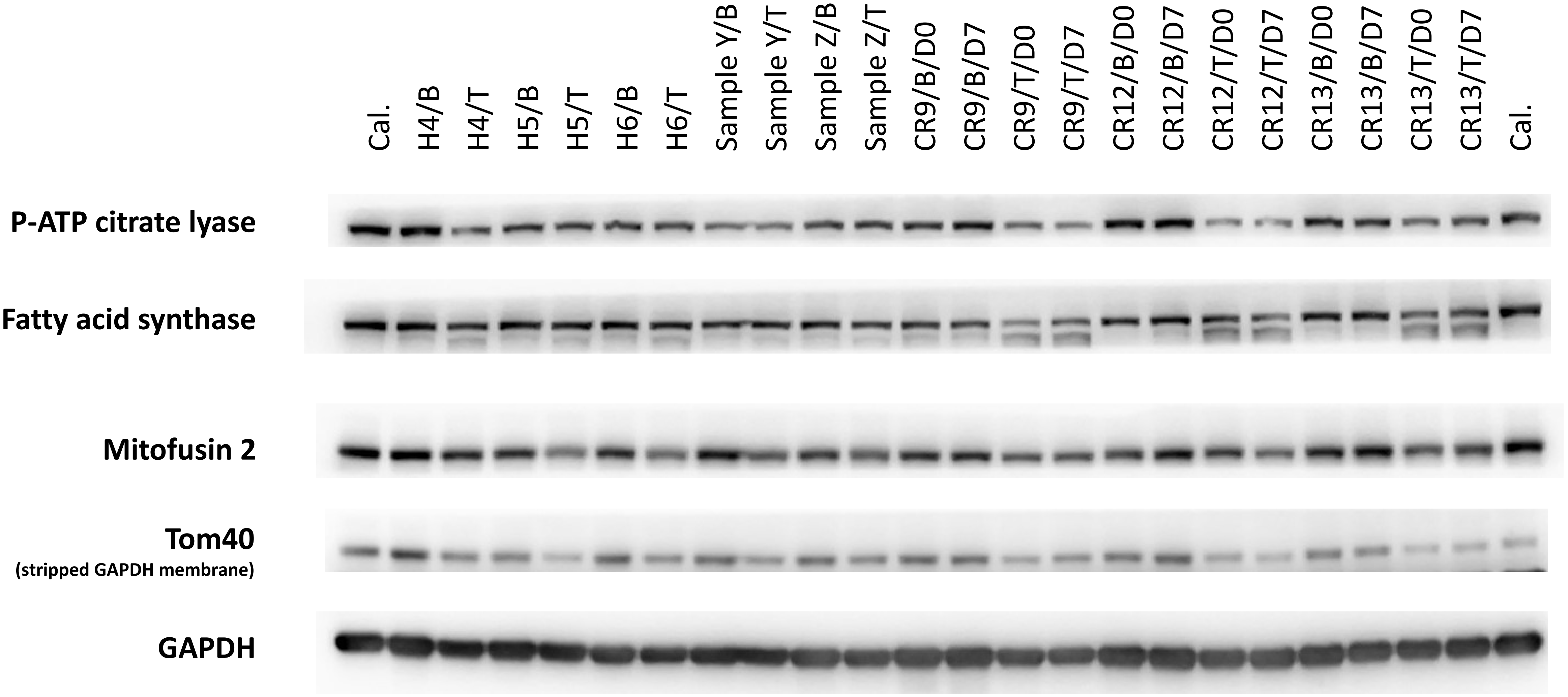

Membrane 3

set B

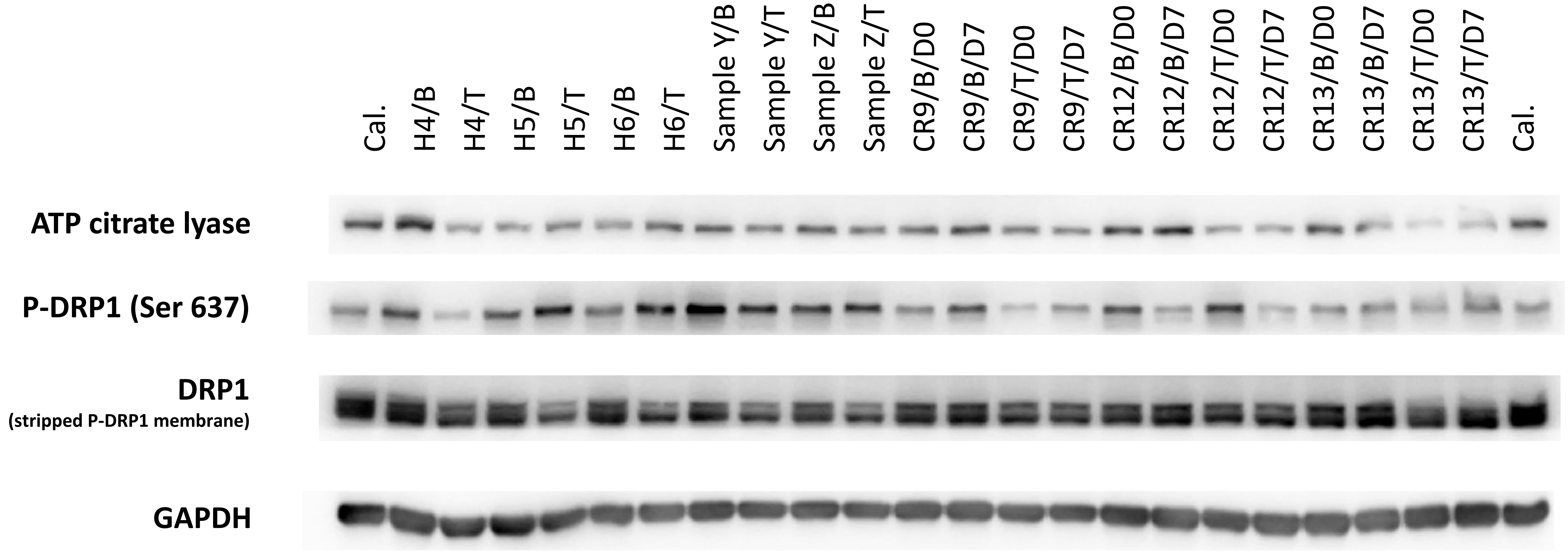

Membrane 4

set B

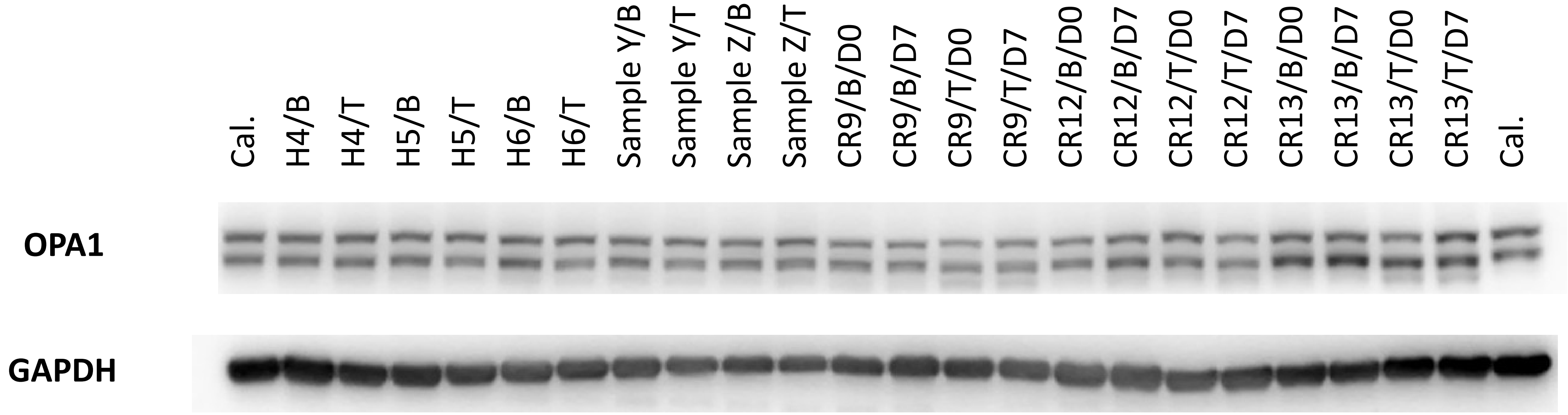

Membrane 5

set B

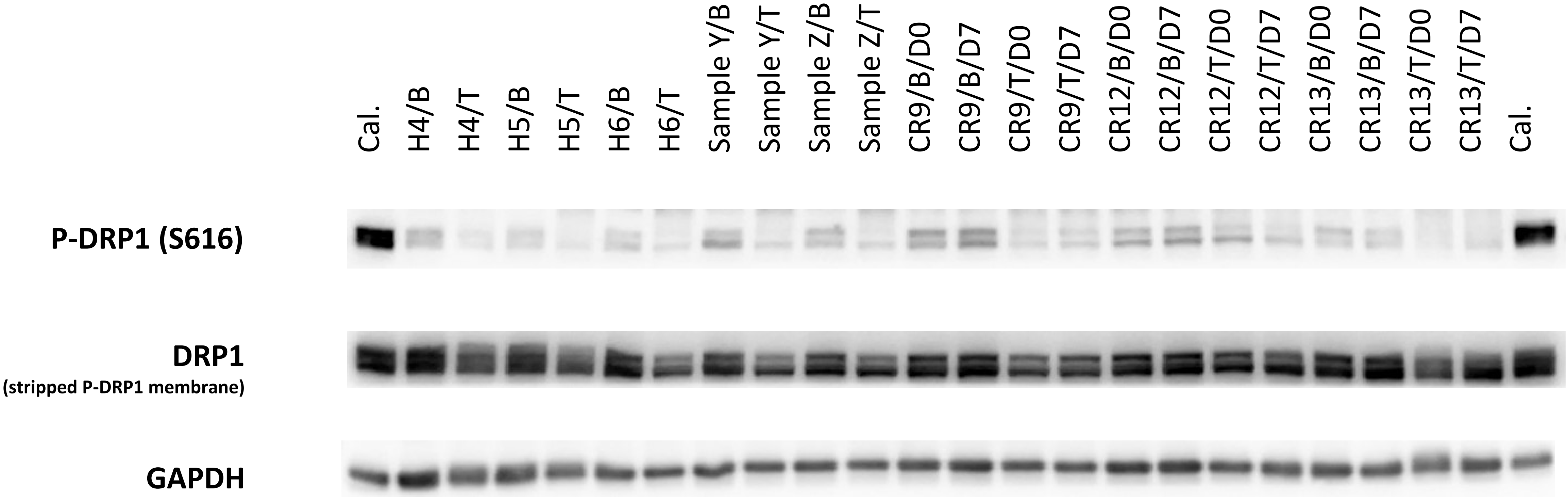

Membrane 1

set C

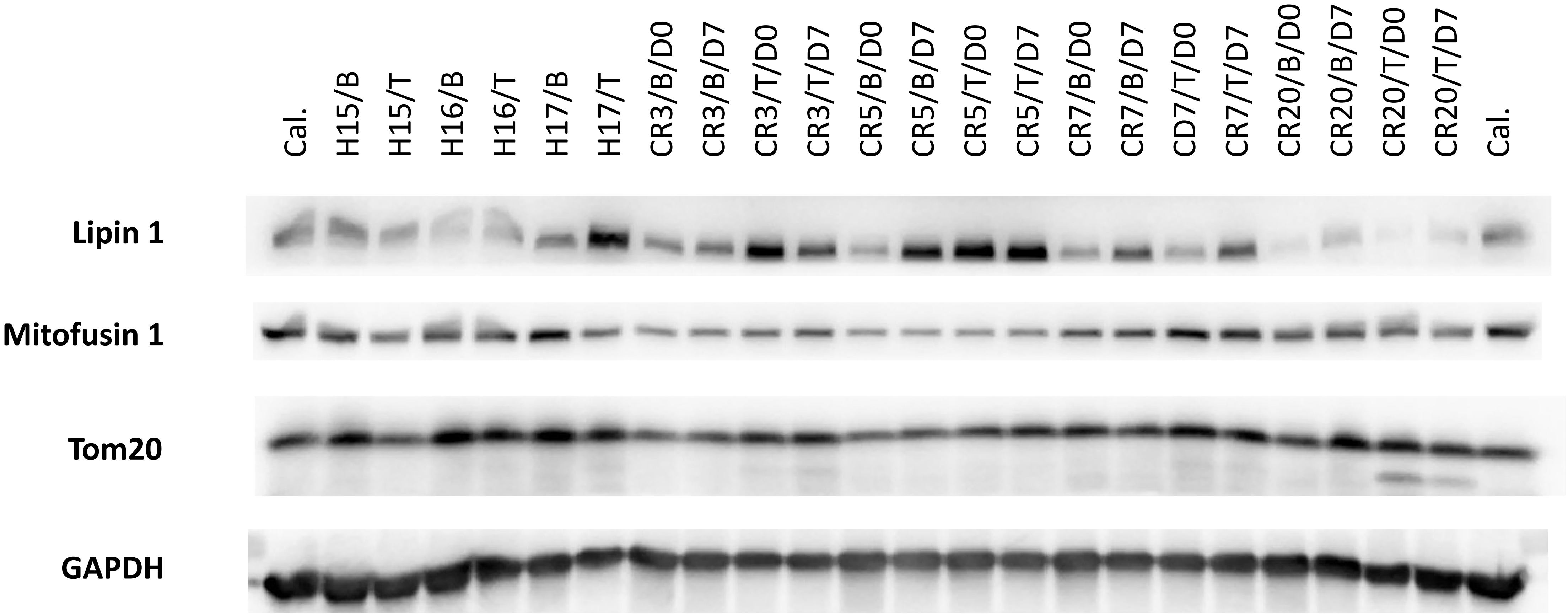

Membrane 2

set C

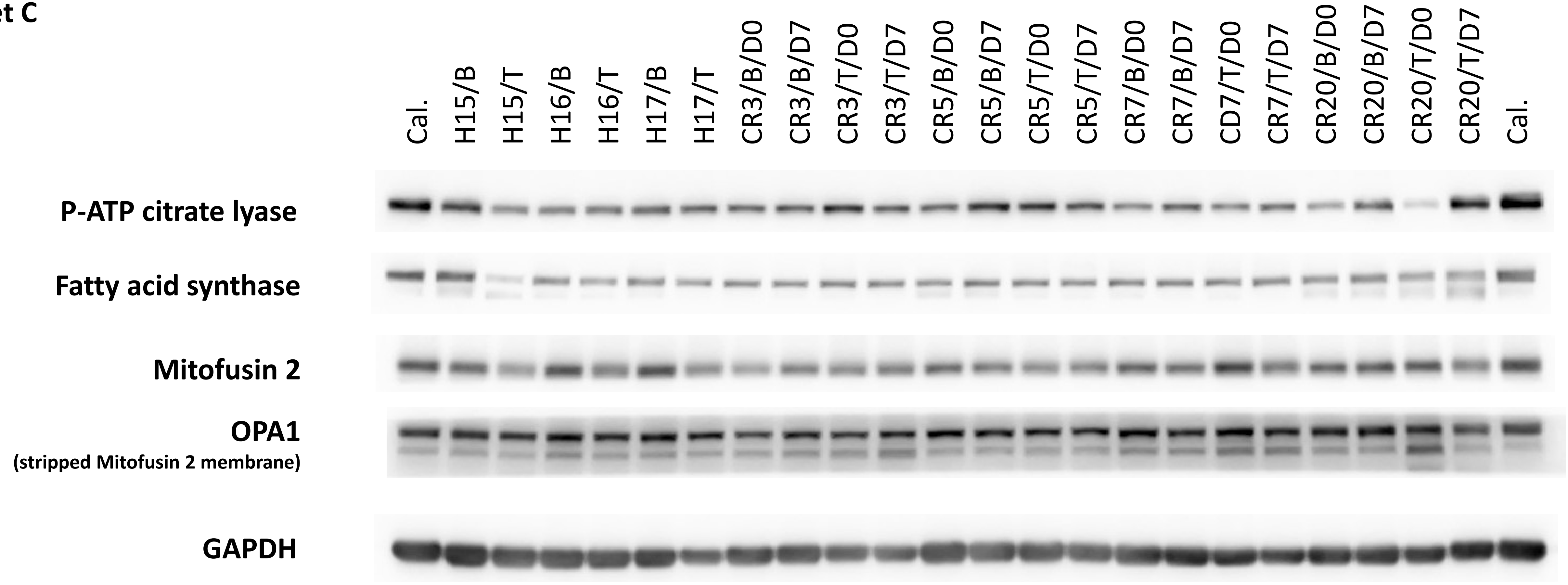

Membrane 3

set C

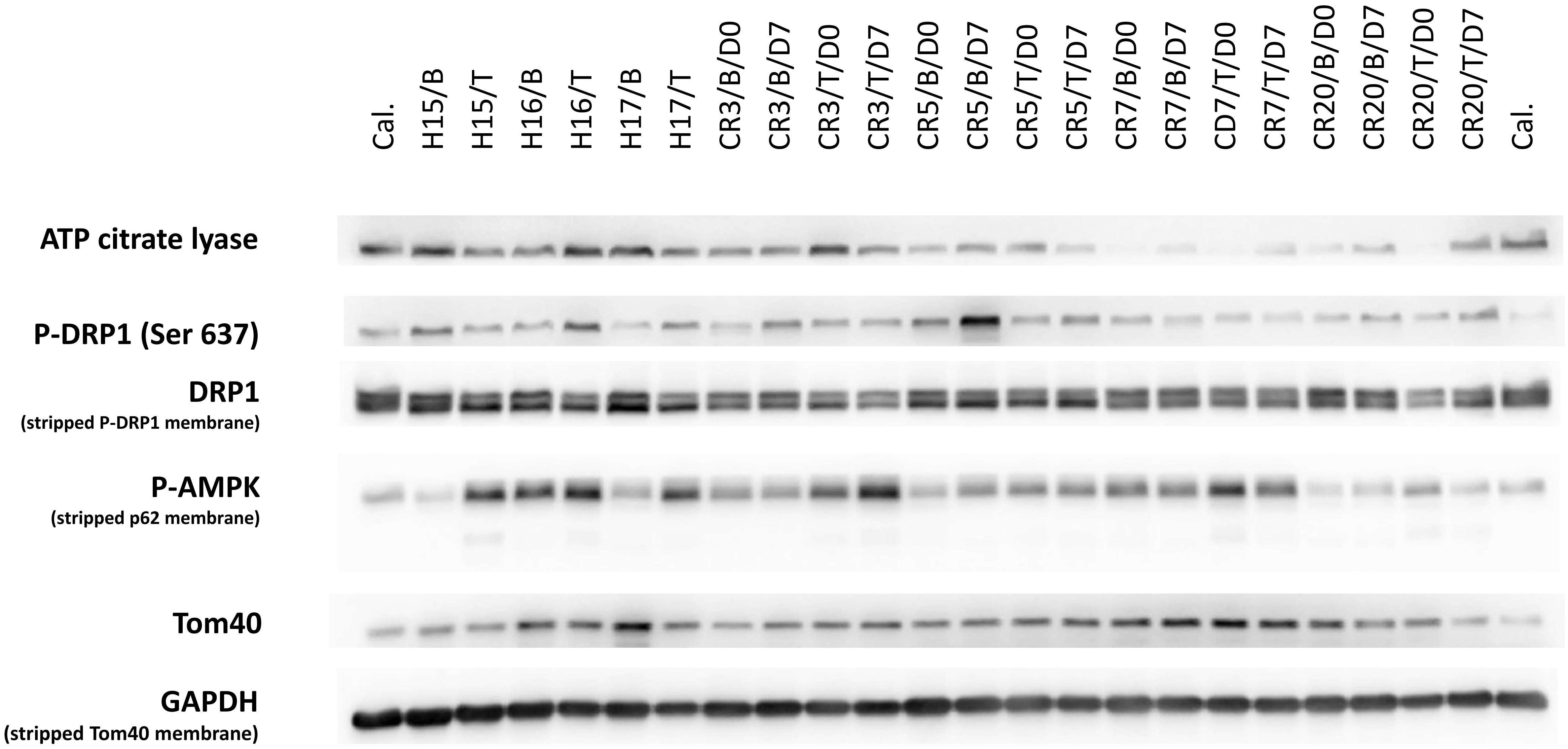

Membrane 4

set C

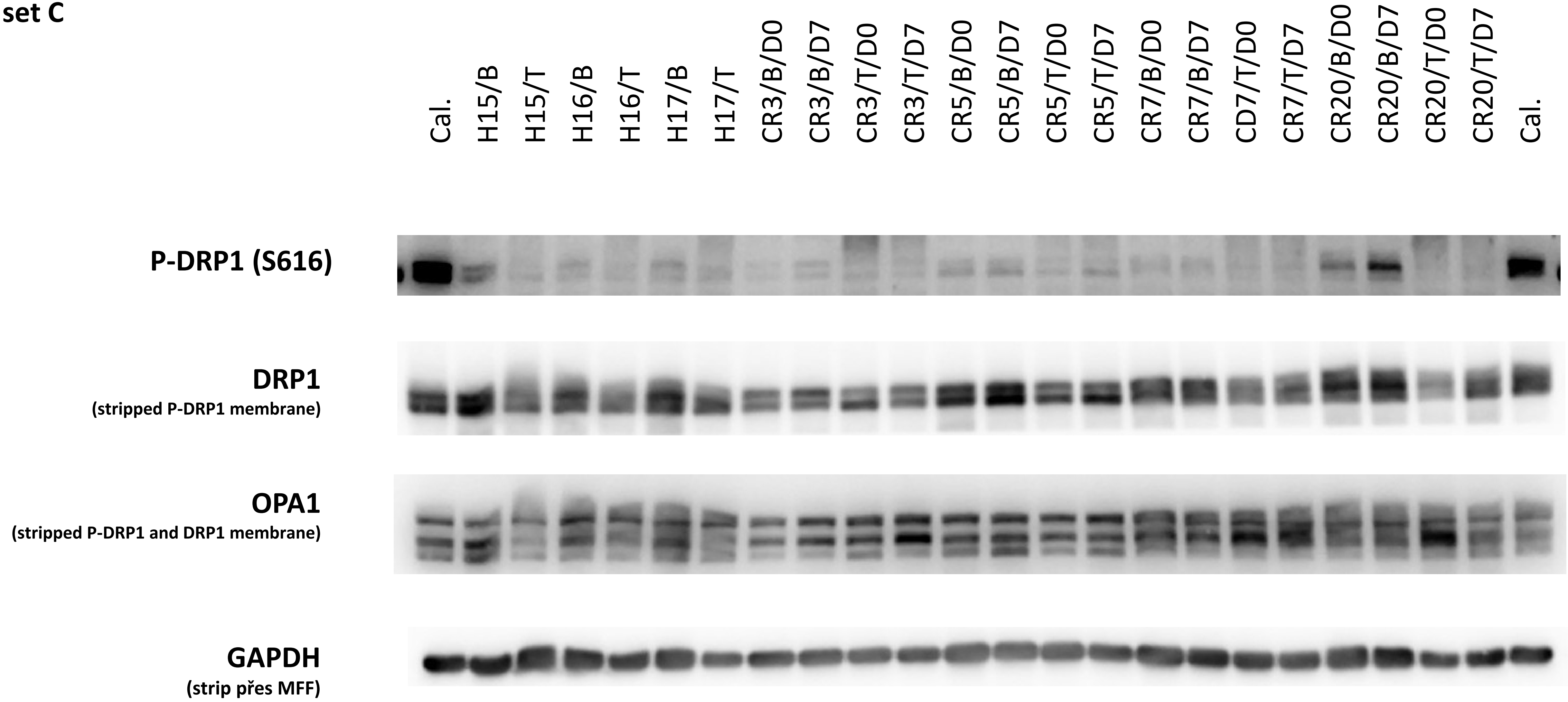

Membrane 1

set D

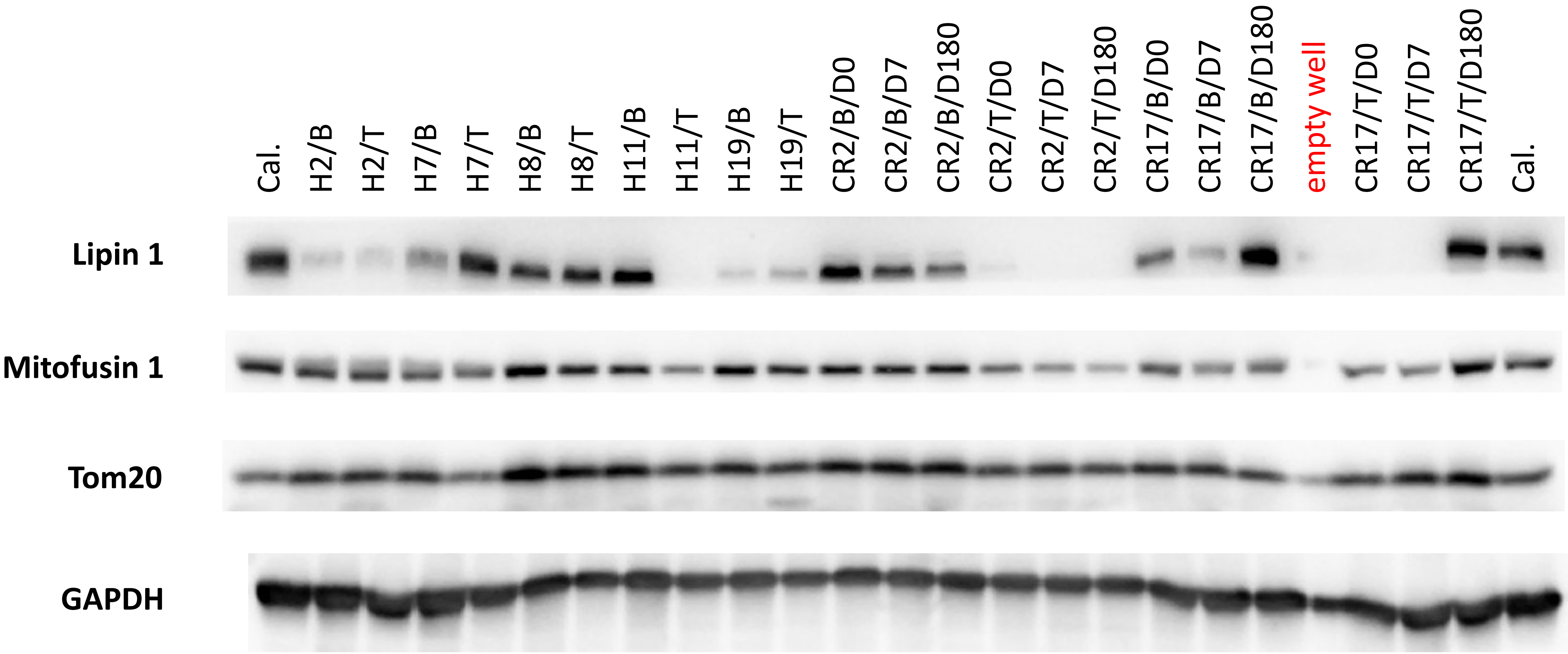

Membrane 2

set D

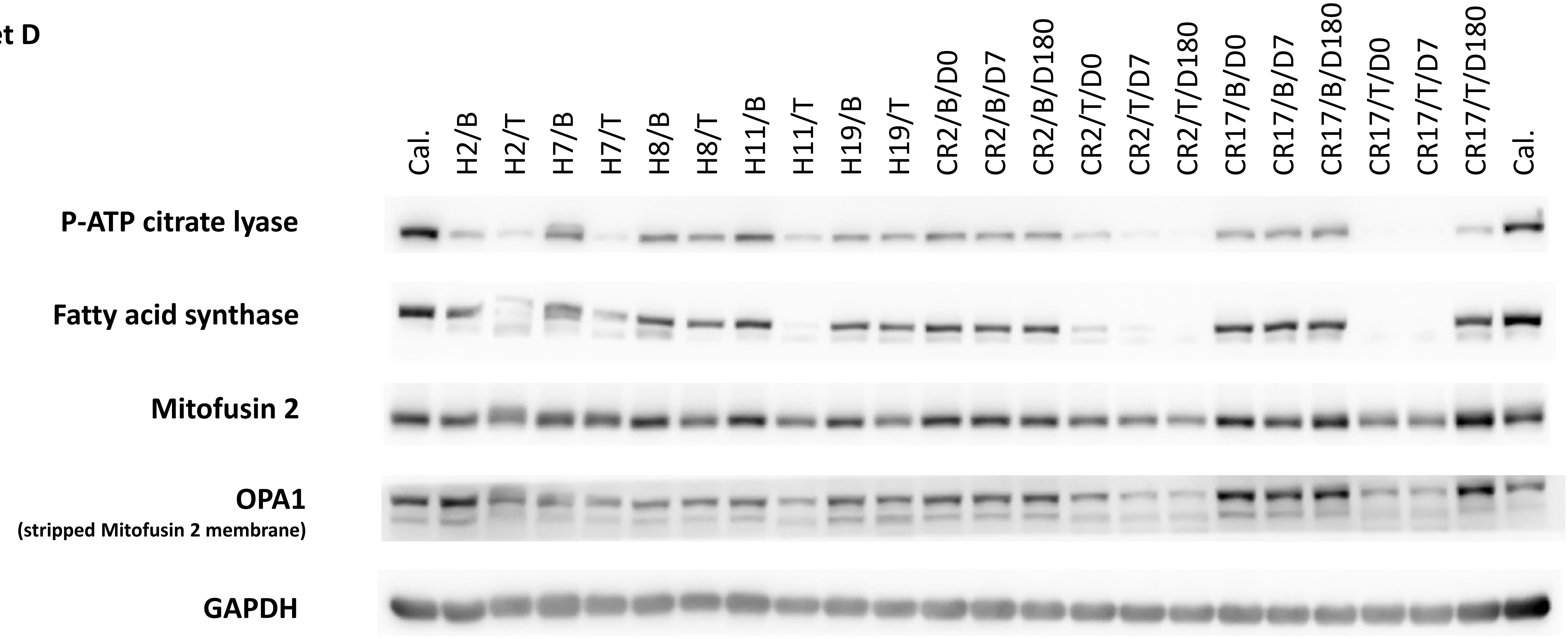

Membrane 3

set D

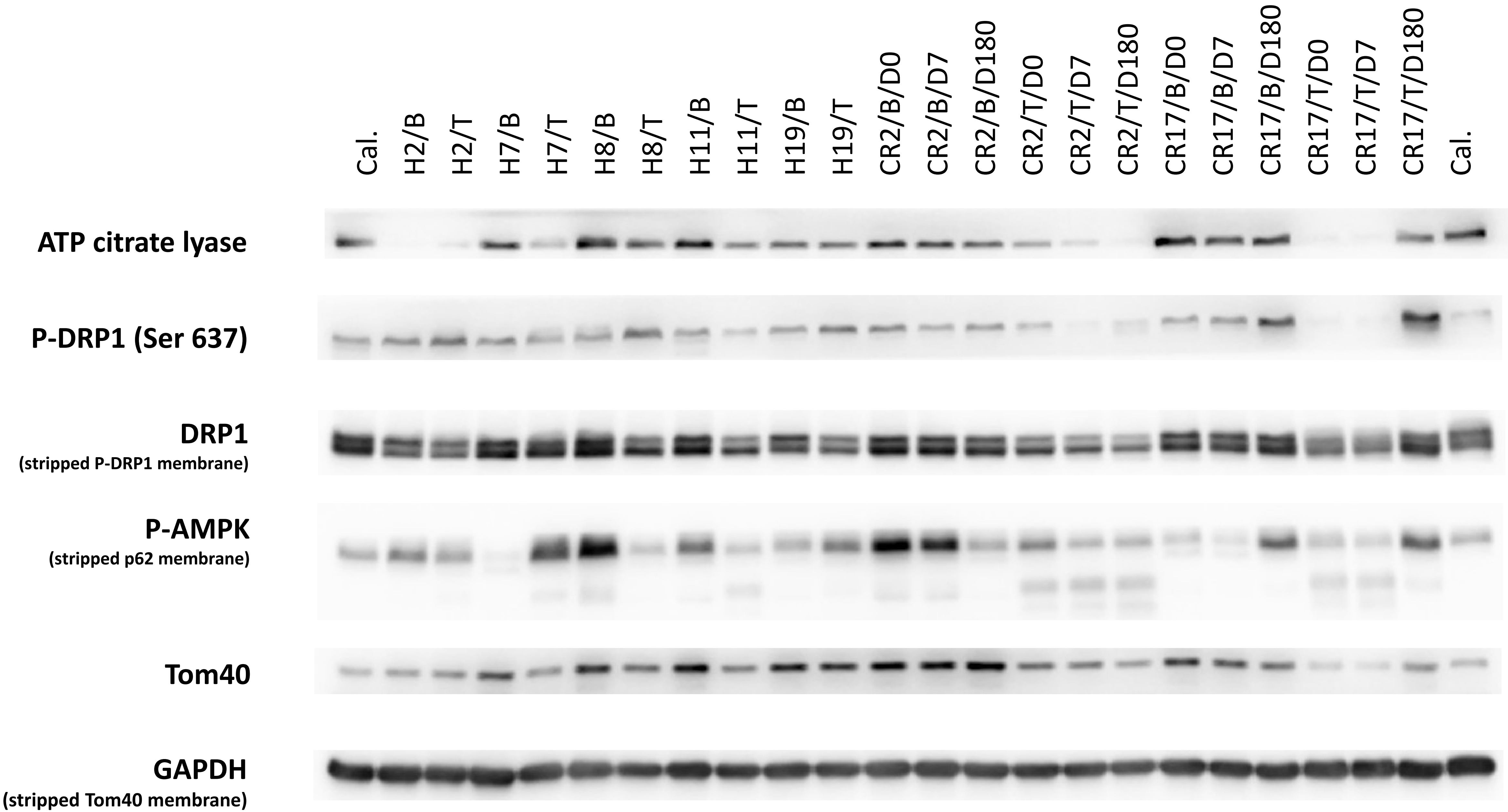

Membrane 4

set D

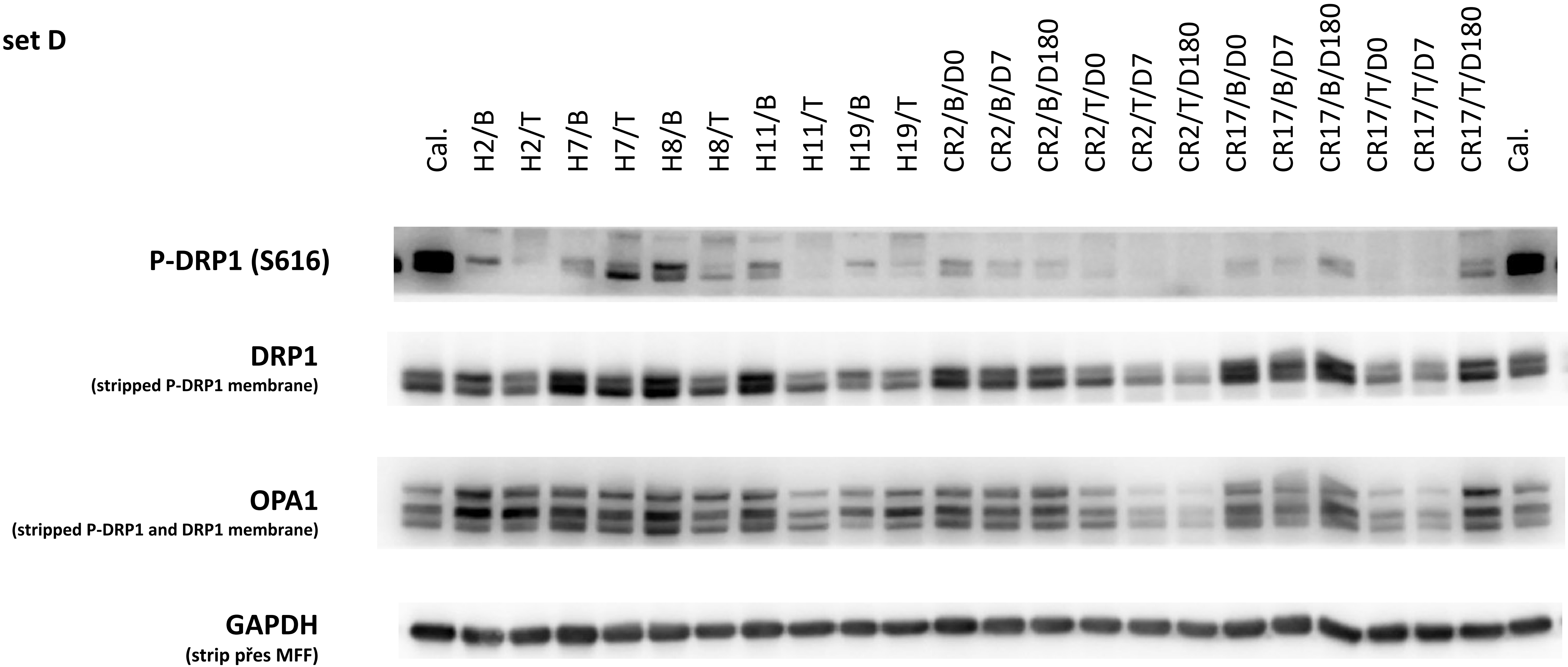

Membrane 1

set E

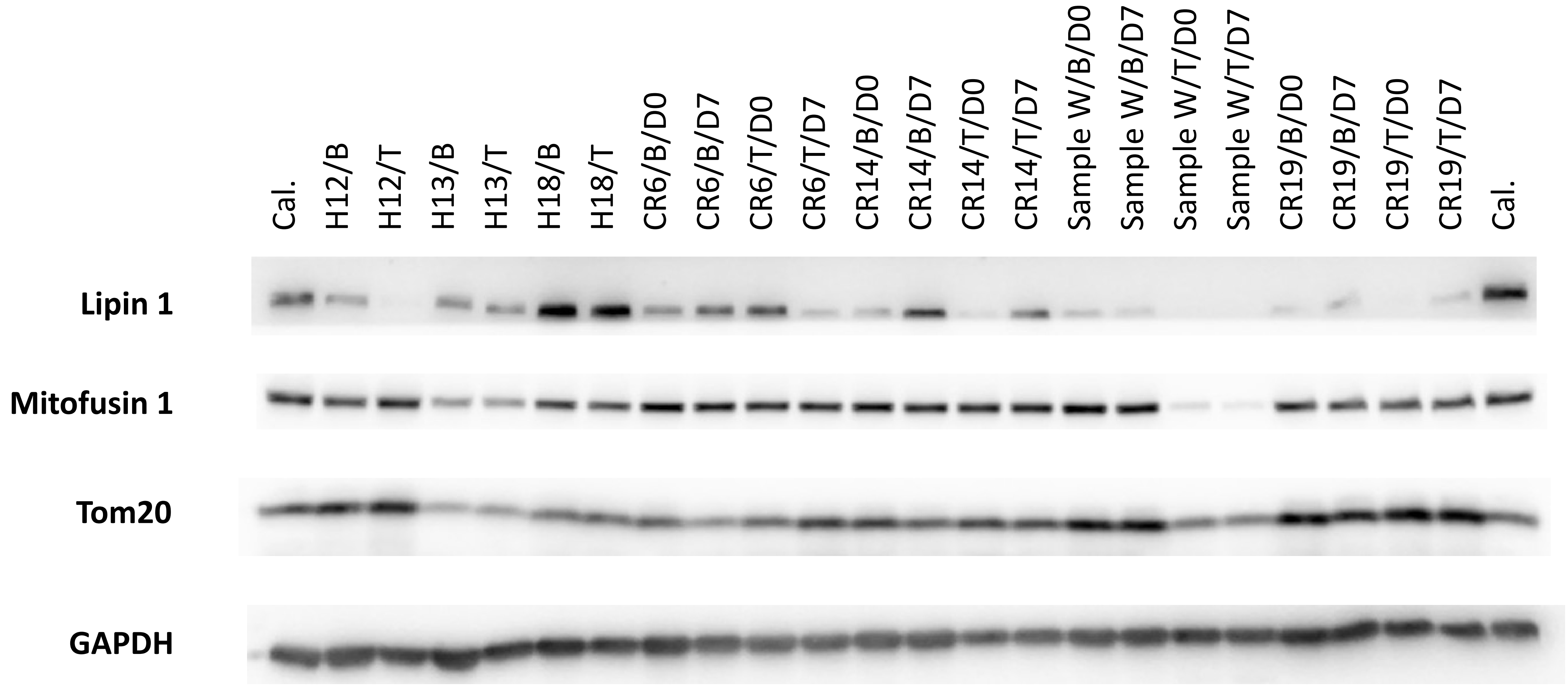

Membrane 2

set E

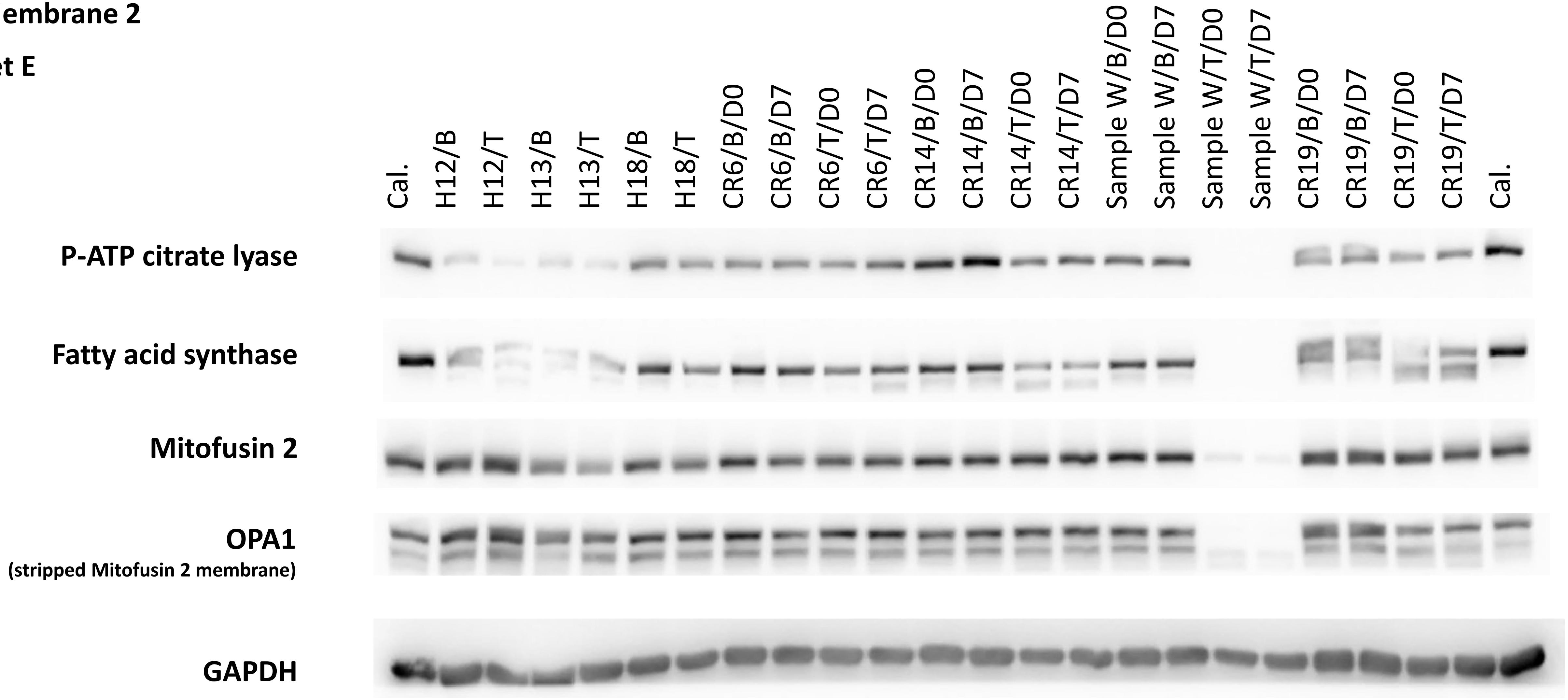

Membrane 3

set E

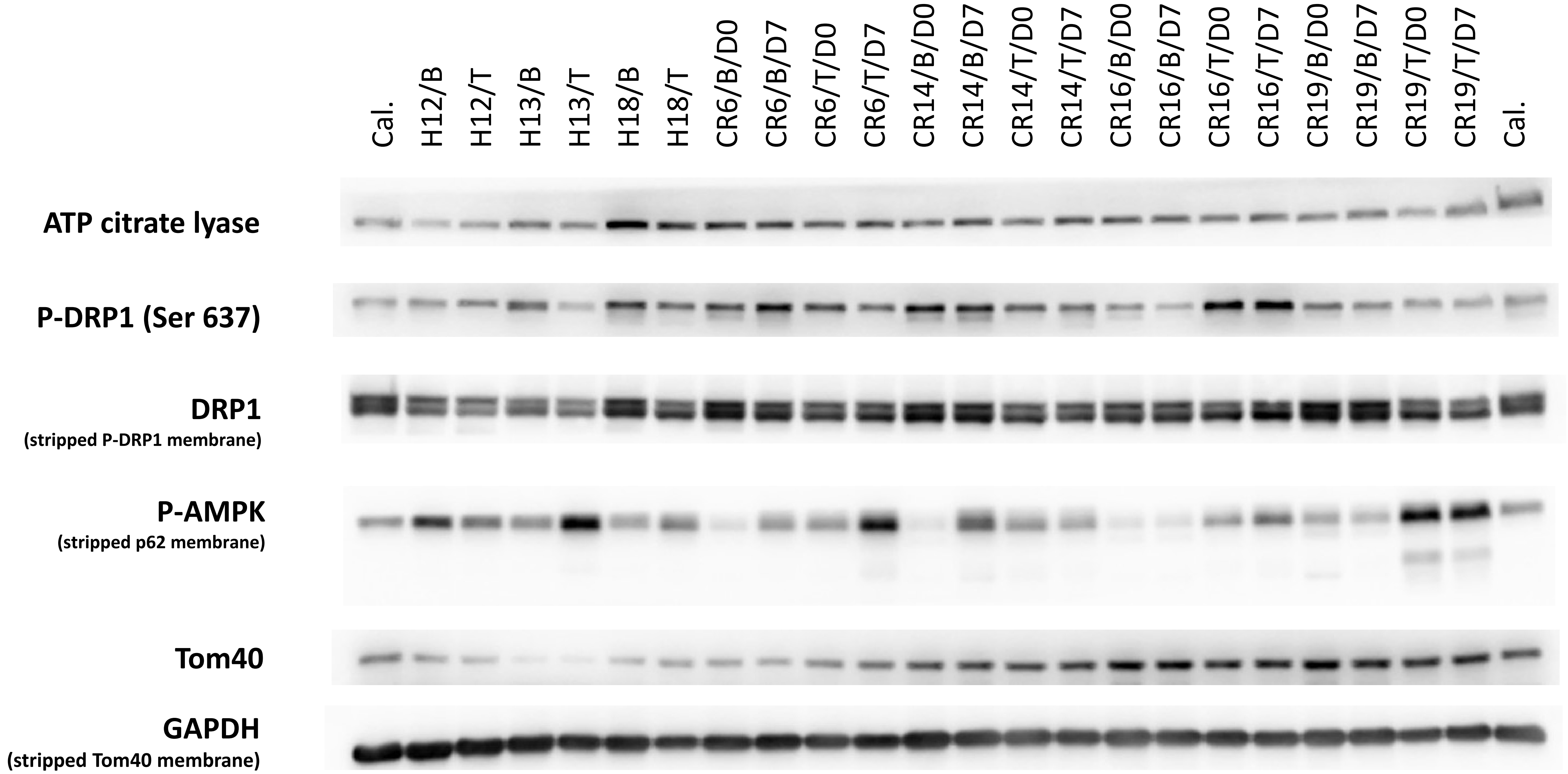

Membrane 4

set E

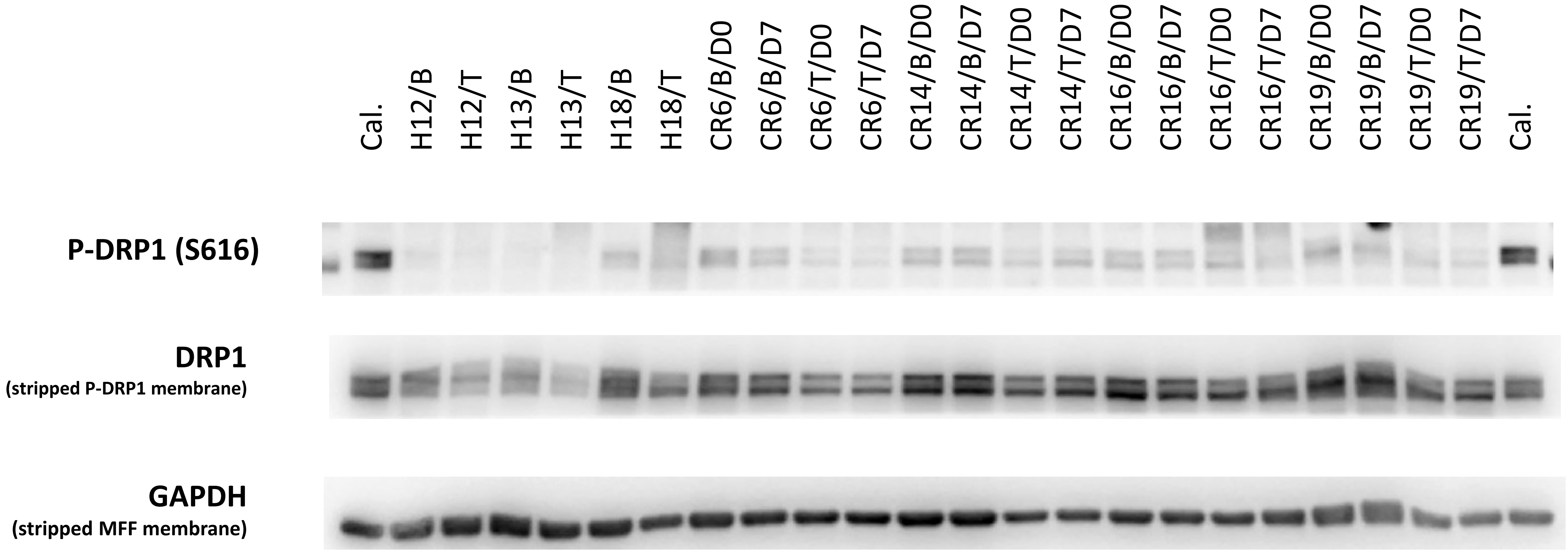

Membrane 1

set F

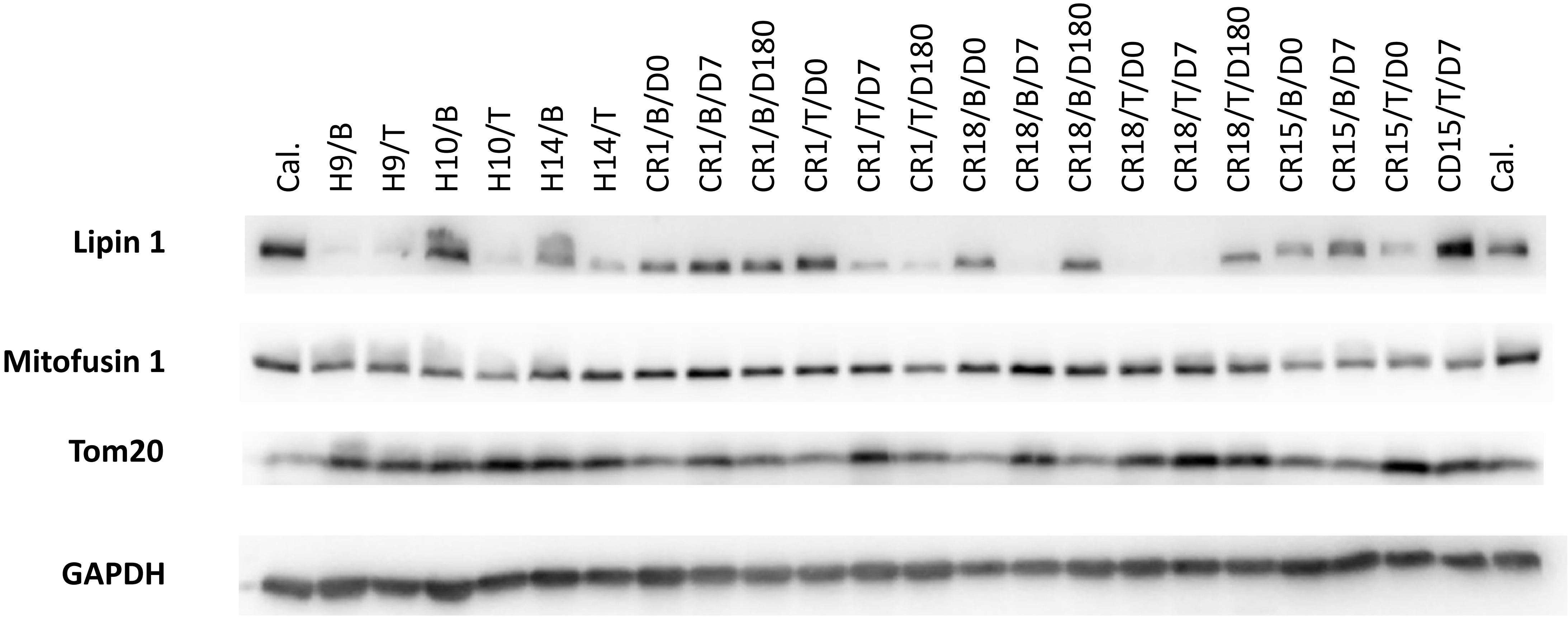

Membrane 2

set F

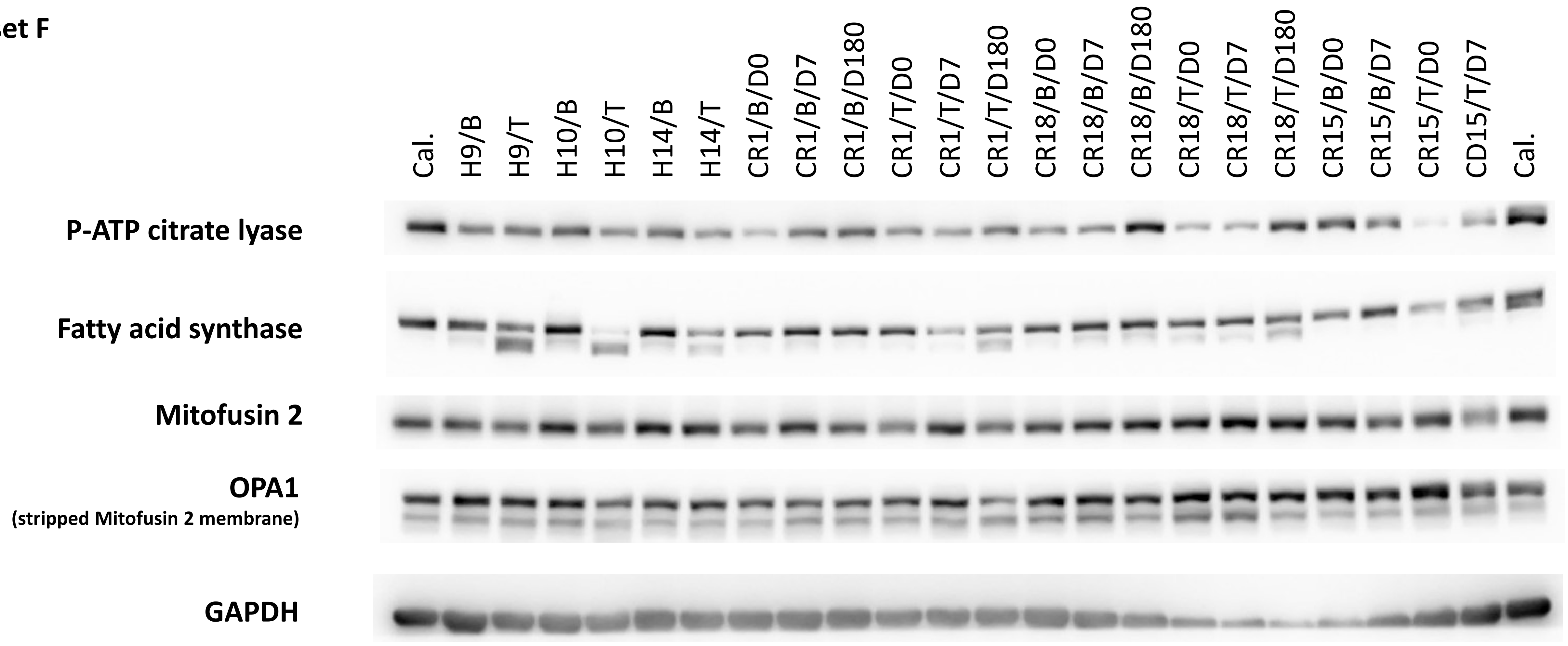

Membrane 3

set F

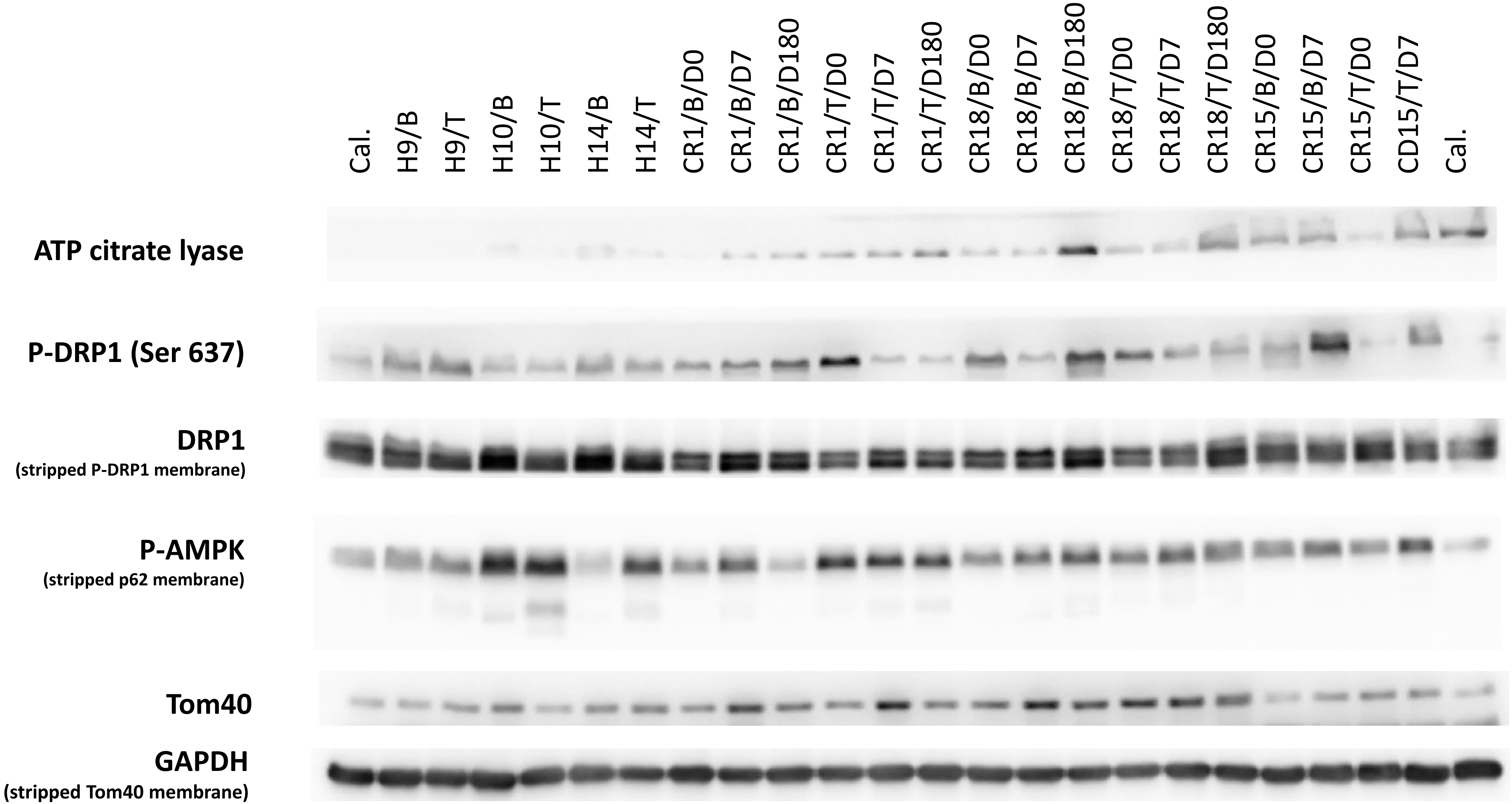

Membrane 4

set F

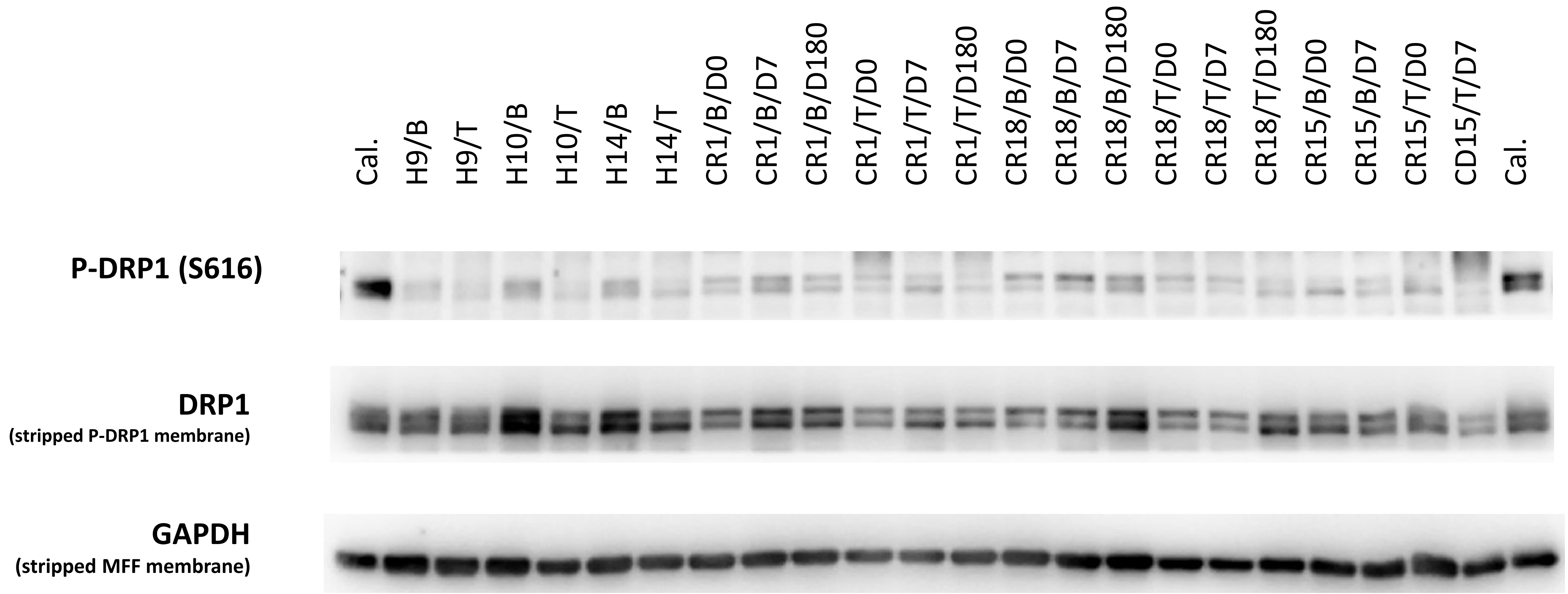

Membrane 1

set ICU16

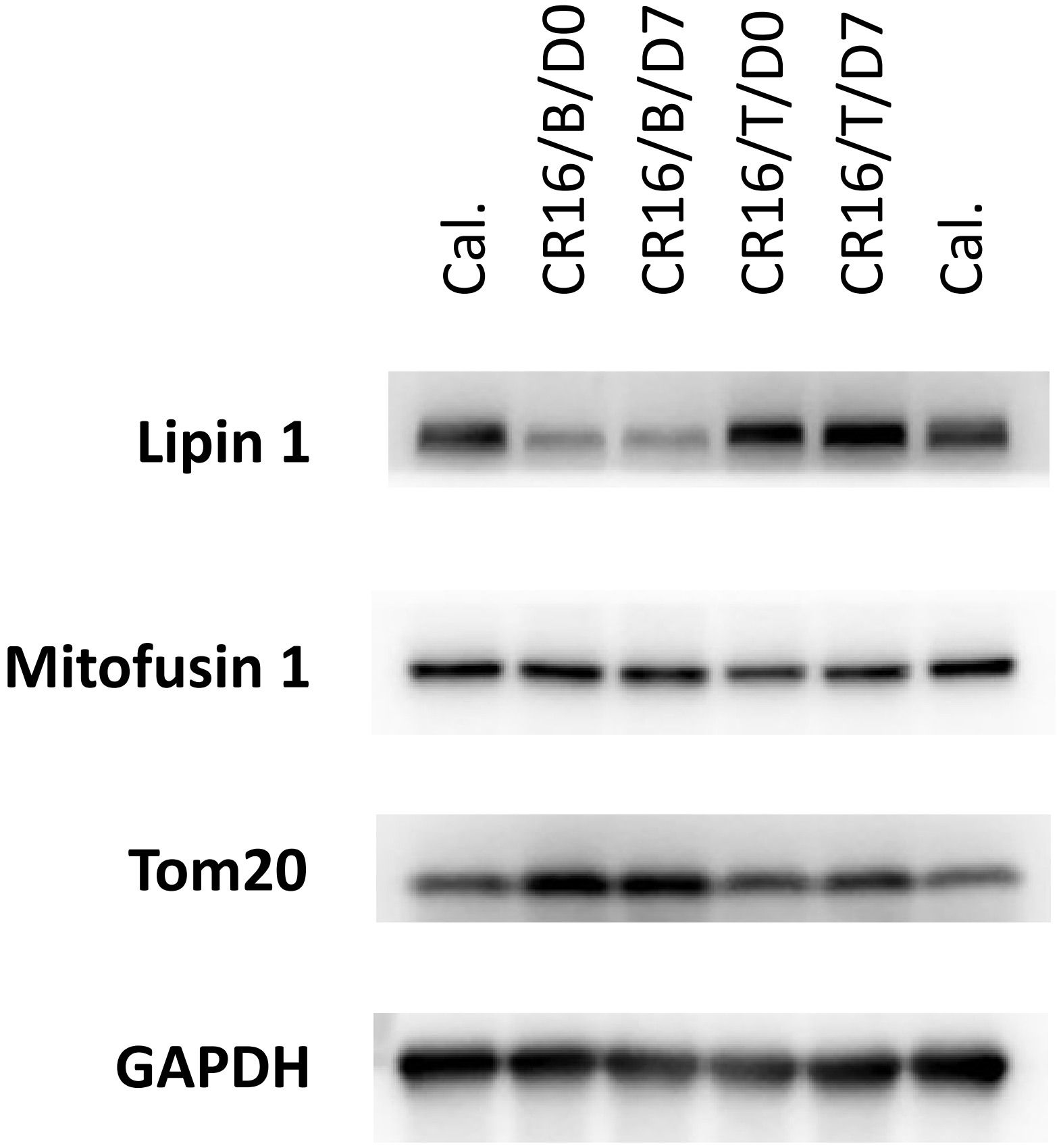

Membrane 2

set ICU16

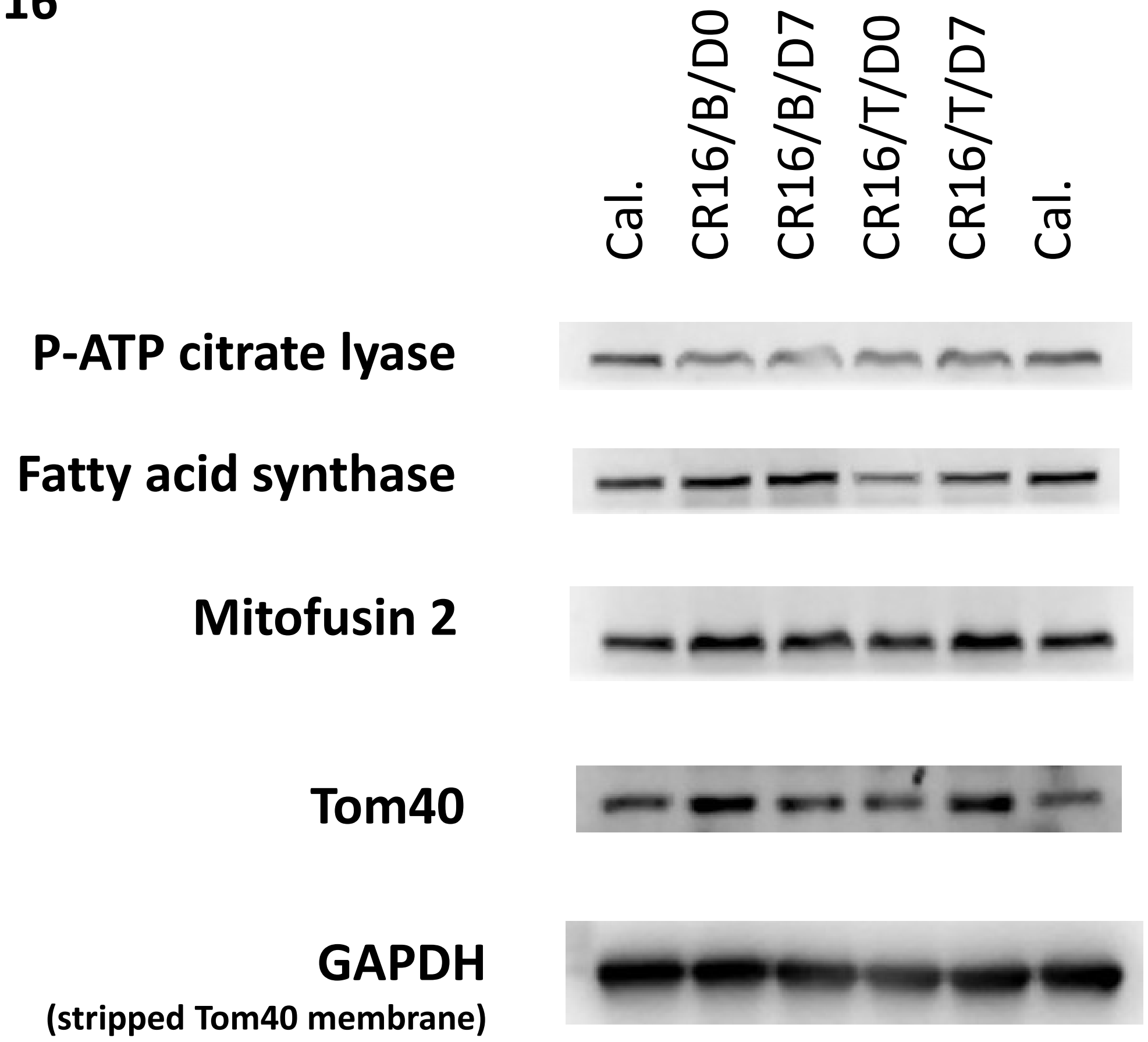

Supplement: Supplementary file 2 [file 13054_2026_6131_MOESM4_ESM.pdf]
