# Supplementary material for: Critical illness impairs coordinated myogenesis: a prospective longitudinal study of skeletal muscle progenitor cell biology using serial muscle biopsies (SATELLITE study)
Source: Crit Care. 2026 Jun 16;30:443. doi: 10.1186/s13054-026-06131-5 (PMC13501688; doi:10.1186/s13054-026-06131-5)
Supplement: Supplementary file 3 [file 13054_2026_6131_MOESM1_ESM.docx]

Supplementary Appendix

To paper: Krajčová et al.: *Critical illness impairs coordinated myogenesis: a prospective longitudinal study of satellite cell biology in ICU survivors*

Contents

[Supplementary Methods	2](#_Toc232460830)

[Supplementary laboratory techniques	2](#_Toc232460831)

[Muscle immunohistochemistry	2](#_Toc232460832)

[Isolation and cultivation of satellite muscle cells	3](#_Toc232460833)

[Cell proliferation Elisa BrdU assay	3](#_Toc232460834)

[Fusion index	3](#_Toc232460835)

[Bioenergetic profile analysis	4](#_Toc232460836)

[Mitochondrial membrane potential and reactive oxygen species production	5](#_Toc232460837)

[Mitochondrial morphology	5](#_Toc232460838)

[Western blots	5](#_Toc232460839)

[Supplementary Statistical Analysis	6](#_Toc232460840)

[Supplementary Results	8](#_Toc232460841)

[SC abundance	8](#_Toc232460842)

[SC proliferation	9](#_Toc232460844)

[Mitochondrial content in SC and ROS production	13](#_Toc232460845)

[Mitochondrial morphology in SC	14](#_Toc232460847)

[Protein expression analyses (Western blots)	15](#_Toc232460848)

[Correlation analysis – selected key associations	16](#_Toc232460849)

[Day 180 survivors versus non-survivors (per-protocol cohort, n = 23)	17](#_Toc232460850)

[Key satellite cell and clinical parameters stratified by Day 180 survival	20](#_Toc232460851)

[Per-protocol versus enrolled without Day 7 biopsy	21](#_Toc232460853)

[SOFA and APACHE II from Day 0 to Day 7	22](#_Toc232460854)

[References:	23](#_Toc232460855)

# Supplementary Methods

## Supplementary laboratory techniques

### Muscle immunohistochemistry

*Cryo-sectioning and fixation.* After biopsy, the muscle tissue was immediately snap-frozen in isopentane cooled by liquid nitrogen and subsequently stored at – 80°C. Later, transverse sections (~ 7 μm) were cut in a cryostat (– 20°C) mounted on Super Frost Plus slides and allowed to dry for ̴̴ 20 min. Sections were then fixed in 4 % paraformaldehyde for 10 min and washed 3x times for 5 min in phosphate-buffered saline (PBS). After that, sections were blocked for 10 min at room temperature in PBS containing 0.5 % Triton and 1 % bovine serum albumin.

*Fibre-type distribution and satellite cell number*

For determination of the satellite cell number and categorization of fibers into type I and II, staining of Pax 7 positive nuclei, total number of nuclei, laminin and type II myosin was performed on the same section as previously described [1]. First, sections were stained with the primary antibodies anti-Pax7 (d MAB1675, Bio-Techne (R&D Systems), Minneapolis, MN, USA; 1:500 dilution), anti-laminin (ab11575, Abcam, Cambridge, UK; 1:1000 dilution) and anti-Fast Myosin Skeletal Heavy Chain (ab91506, Abcam Cambridge, UK; 1:1000 dilution) and incubated overnight in the dark at room temperature. Next day, the sections were washed in PBS 2x times for 5 minutes and incubated in secondary antibodies Alexa-fluor 488 goat-antimouse (A.11029, Thermo Fisher Scientific, USA, 1:1000 dilution) and Alexa-fluor 568 goat-antirabbit (A.11036, Thermo Fisher Scientific, USA, 1:1000 dilution) for 2 hours at room temperature. Then, the sections were washed again 2xtimes in PBS for 5 min, allowed to in the dark and fixed with aqueous anti-fade mounting medium with DAPI (ab104139, Abcam, Cambridge, UK) to visualize nuclei of total cells at 4°C for 24 hours. Staining specificity was confirmed using appropriate negative controls. Microscopy images were obtained at × 20 magnification using the fluorescence microscope Zeiss Axio Scan.Z1. Images were analysed using a software program Image J (Fiji).

### Isolation and cultivation of satellite muscle cells

*Isolation of mixed-cell population.* Second part of muscle tissue was used for isolation of skeletal muscle cells as previously described [2]. In brief, muscle tissue was minced and cells were subsequently isolated by enzymatic digestion in the solution of Collagenase D (5 mg/mL) and Dispase II (1.2 U/mL) at 37°C for ̴̴ 60 minutes. Fetal bovine serum was then added to stop enzymatic process; the solution was filtered through a 100 µm strainer and red blood cell lysis solution was added to remove mononuclear cells followed by additional filtration through 40 µm strainer. Cells were then plated on gelatin-coated cell culture flasks in DMEM containing 15 % fetal bovine serum, 1% solution of 100 U/mL penicillin and 100 μg/mL streptomycin, 5.56 mM glucose, 1 mM pyruvate, 0.05 μg/mL fungizone, 0.4 μg/mL dexamethasone, 0.5 mM L-glutamine, 10 µg/mL insulin, 10 ng/mL epidermal and 1 ng/mL fibroblast growth factors and kept at 37°C in a humidified atmosphere of 5 % CO_2_ [3]. All chemicals were purchased from Merck Millipore (Darmstadt, Germany) or Thermo Fisher Scientific (USA), unless otherwise stated.

*Purification of satellite cells.* After cultivation of	̴̴ 1 week in standard conditions, cells reached	̴̴ 90 % confluence and magnetic-activated cell sorting was performed to obtain a purified myogenic sub-population as previously described [4]. In brief, the mixed muscle-derived population was incubated with magnetic microbeads conjugated to an antibody against CD56 (Miltenyi Biotec Inc., USA) and then passed through a magnetic field, plated and cultured at standard conditions. Images were analysed using a software program Image J (Fiji).

### Cell proliferation Elisa BrdU assay

Proliferation was assessed using BrdU Cell proliferation Elisa colorimetric kit (11647229001; Merck Millipore, Darmstadt, Germany) according to manufacturer’s instructions. Briefly, cells were seeded at the density of 1 x 10^4^ cells per well overnight in 96-multiwell plate. Next day, BrdU was added and cells were subsequently fixed with fixing solution followed by incubation with anti-BrdU antibody. The absorbance was measured at 370 nm using a microplate reader (TECAN Infinity 200 PRO).

### Fusion index

Upon	̴̴ 90 % confluency, medium was changed to DMEM with 2 % Horse serum to promote differentiation of myoblasts and form multinucleated myotubes. After 7-8 days, differentiated myotubes were fixed in 4 % formaldehyde solution in PBS for 15 minutes at room temperature and after that stained with DAPI and CellMask™ Green Actin Tracking Stain (Thermo Fisher Scientific, USA; 1X) to visualize nuclei and cytoskeleton (F-actin), respectively. Microscopy images were obtained at 20 x water objective (Leica TCS SP8 system, Leica Microsystems). The fusion index was calculated as the ratio of at least 3 nuclei within differentiated myotubes over the total number of nuclei in the culture. Analysis was performed using a MATLAB-based software MyoCount [5].

### Bioenergetic profile analysis

*Extracellular Flux analysis.* Extracellular Flux Analyzer (Agilent Technologies Inc., Santa Clara, CA, USA) was used to measure oxygen consumption rate (OCR) in living cells seeded on 96-well plate at 37° C at the baseline and after a sequential addition of four compounds [6]. Prior to experiment, the cells were seeded on XF 96 cell culture plates at the density of 1 x 10^4^ cells/per well overnight. Next day, before starting the XF assay, DMEM medium was replaced by the assay medium containing 10 mM Glucose, 1 mM Pyruvate and 2 mM Glutamine to perform experiment under conditions of saturating substrates [7]. Respiration in intact cells was firstly measured at the baseline (**Figure S1**). After that, substrate preference was determined using addition of specific inhibitors of oxidation pathways (pyruvate inhibitor carrier UK5099 for glycolysis; 2 µM), etomoxir (inhibitor for carnitine palmitoyl-transferase I - long-chain fatty acids oxidation; 4 µM) and BPTES (glutaminase inhibitor; 3 µM). In the control group, medium with no inhibitor was added. Subsequently, oligomycin (1 µM) was added to block ATP synthase for determination of ATP production and proton leak. Uncoupling agent carbonyl cyanide-4- (trifluoromethoxy)phenylhydrazone (FCCP; 1 µM) was then injected to determine maximal respiratory capacity. Finally, antimycin A (AA; 4 µM) and rotenone (rot; 3 µM) was added to block complex III and determine non-mitochondrial respiration. The sequential compound injections in the absence and presence of inhibitor (etomoxir or UK5099 or BPTES) allowed to measure acute response to an inhibitor, and maximal respiration [7]. Each experimental group was measured in tri- to hexa-plicates.

**Supplementary Figure S1.** **Mitochondrial respiratory function indices in satellite cells from ICU patients and healthy controls**. Representative oxygen consumption rate (OCR) traces during mitochondrial stress testing in **(a)** healthy controls and **(b)** ICU patients at day 0, day 7, and day 180. Sequential additions of oligomycin, FCCP, and antimycin A/rotenone (AA/rot) were used to assess mitochondrial respiratory parameters. OCR values are normalized to citrate synthase activity.

### Mitochondrial membrane potential and reactive oxygen species production

Mitochondrial membrane potential and reactive oxygen species (ROS) production were determined as previously described [8, 9]. In brief, membrane potential was determined and quantified in living cells using Confocal Laser Scanning Microscopy with a 63x oil immersion objective (Leica TCS SP8 system, Leica Microsystems). Cells were stained on glass coverslips with a cell permeant positively-charged red dye tetramethylrhodamine ethyl ester (TMRE; 7 nm), that readily accumulates in active mitochondria due to their relative negative charge. Prior to staining with TMRE, cells were also stained with MitoTracker^TM^ Green FM (MTG; 200 nm) to normalize the values on mitochondrial volume. The fluorescence was then examined under normal growth conditions (37°C, 5 % CO_2_). Mitochondrial membrane potential was calculated as a ratio of TMRE fluorescence intensity to MTG fluorescence intensity. For ROS production, MitoSOX Red superoxide indicator (1.25 µM) was used to detect mitochondrial superoxides in live cells. ROS production was calculated as a ratio of MitoSOX intensity to MTG fluorescence intensity as described above. For each experiment, confocal laser scanning imaging was performed in two or three independent replicates per subject. All the images were analysed with Image J (Fiji).

### Mitochondrial morphology

MitoTracker™ Green FM stained mitochondria were used for analysis of mitochondrial network architecture. Parameters of mitochondrial morphology (individuals, networks, mean branch length, mean network size, footprint) were analysed with ImageJ™ plugin “MINA” (Valente) in a software program Image J (Fiji) [10]. For each experiment, confocal laser scanning imaging was performed in two or three independent replicates per subject.

### Western blots

Samples containing 10 µg of proteins were mixed with Laemmli sample buffer with DTT. Proteins (without any heat denaturation prior to loading to prevent aggregation of membrane proteins) were separated on 12% polyacrylamide gels at 150 V and then blotted onto 0.2 μm nitrocellulose membrane (Protran BA83, Schleicher-Schuell, Dassel, Germany) for 3 h at 0.25 A employing Biorad Criterion devices for midi-format electrophoresis and blotting. The membranes were blocked in 5% weight/volume Bovine Serum Albumin in Tris-buffered saline for 30 min at room temperature. The washed membranes were probed at 4°C overnight with Tom40 antibody (sc-365467, Santa Cruz Biotechnology, 1:1000 dilution) or antibodies against Tom20 (#42406), ATP-Citrate Lyase Antibody (#4332), Phospho-ATP-Citrate Lyase (Ser455) Antibody (#4331), Fatty Acid Synthase (C20G5) Rabbit Monoclonal Antibody (#3180), Lipin 1 (D2W9G) Rabbit Monoclonal Antibody (#14906) and Phospho- AMPK alpha (Thr172) (40H9) Rabbit Monoclonal Antibody (#2535), OPA1 (#80471), DRP1 (#8570), Phospho-DRP1 (Ser616) (#4494), Phospho-DRP1 (Ser637) (#6319), Mitofusin-1 (#14739) and Mitofusin-2 (#11925) from Mitochondrial Dynamics Antibody Sampler Kit II (#74792, Cell Signaling, antibodies dilution 1:1000). To demonstrate equal loading, the membrane was probed with anti-GAPDH antibody (dilution 1:1000; sc-365062, Santa Cruz Biotechnology or #5174, Cell Signaling). After washing, the membranes were incubated for 2 hours at room temperature with corresponding horseradish peroxidase-conjugated secondary antibody (dilution 1:10 000; Proteintech). Protein bands were visualized with an enhanced chemiluminescence detection system (Thermo Fisher Scientific, USA) using Alliance Q9 Imaging system (Uvitec Cambridge). Densitometry was performed using the Image Lab 6.1 software (Biorad). For comparison of protein expression across multiple Western blot membranes, necessitated by the large number of patient samples, the same calibrator sample was loaded on both ends of each membrane. The average signal intensity of these calibrator duplicates was used for normalization of target protein levels, enabling reliable comparison across individual blots.

## Supplementary Statistical Analysis

For completeness, group comparisons between healthy controls and ICU patients at ICU day 0 and day 7 were additionally evaluated using the **Mann–Whitney U test**, a non-parametric test assessing whether one distribution tends to have higher values than another. Given the exploratory and mechanistic nature of the study, p-values are reported as supportive information, while effect sizes are prioritized in interpretation.

Formal statistical testing was not performed for ICU day 180 due to the limited number of available samples.

#### Use of Cliff’s Delta for Group Comparisons

Satellite cell proliferation slopes demonstrated non-normal distributions with inter-individual variability, and unequal group sizes. Under these conditions, conventional parametric effect size measures (e.g. Cohen’s *d*) are inappropriate. Therefore, Cliff’s delta (δ) was used to quantify the magnitude and direction of group differences.

Cliff’s delta is a non-parametric effect size that estimates the probability that a randomly selected observation from one group is greater than a randomly selected observation from another group, minus the probability of the reverse:

$$\delta=P(X>Y)-P(X<Y)$$

where *X* and *Y* denote observations from two independent groups. The value of δ ranges from −1 to +1, with 0 indicating complete overlap between groups. Positive values indicate a tendency toward higher values in the first group, while negative values indicate the opposite.

Cliff’s delta makes no assumptions regarding data normality or homoscedasticity and is robust to outliers and unequal sample sizes, making it particularly suitable for heterogeneous biological data derived from human mechanistic studies. As a general guide, absolute δ values of approximately 0.15, 0.33, and 0.47 are interpreted as small, moderate, and large effects, respectively.

In this study, Cliff’s delta was computed by pairwise comparison of all observations between groups, counting the number of instances in which one group’s value exceeded the other. Effect sizes are reported alongside descriptive statistics to emphasize biological magnitude rather than dichotomous statistical significance.

# Supplementary Results

## SC abundance

**Supplementary Figure S2. Fibre type distribution, fibre cross-sectional areas and satellite cell number. Upper part: (a)** Representative fluorescent images of muscle cross-sections: myofibers stained for myosin heavy chain (MHC) II (red, type II), laminin (red) and nuclei (DAPI). Non-labelled myofibers represent type I fibers. Images represent healthy subjects (n = 20), ICU Day 0 (n = 21), ICU Day 7 (n = 20) and ICU Day 180 (n = 6). Scale bar: 100 μm; **(b)** Representative fluorescent image of satellite cell identification in muscle fibers: 2 satellite cells (SC) are identified = arrow heads showing 2 nuclei labelled with both Pax7^+^ and DAPI. Scale bar: 50 μm. **Lower part:** Quantitative comparison of fibre type size, distribution and satellite cell number across groups, including **(c)** cross-sectional area (CSA) of type I fibers, **(d)** Minimal Ferets´diameter of type I fibers, **(e)** Relative abundance of type I fibers, **(f)** cross-sectional area of type II fibers, **(g)** Minimal Ferets´diameter of type II fibers, **(h)** Relative abundance of type II fibers, **(i)** satellite cell number per fibre. Box plots show the median and interquartile range, with whiskers indicating the spread of the data; individual dots represent individual samples. Note: CSA, cross-sectional area, MHC, myosin heavy chain, SC, satellite cells.

## SC proliferation

**Supplementary Figure S3. Distribution of proliferation slopes in controls and ICU patients.** Box-and-whisker plots displaying satellite cell proliferation slopes in healthy controls and ICU patients at each time point. Boxes indicate medians and interquartile ranges, with individual data points overlaid.

**Supplementary Figure S4**: Early satellite-cell proliferative capacity at ICU admission was not associated with absolute muscle mass at day 7 (Spearman r = −0.22, p = 0.37) nor with short-term changes in muscle mass over the first ICU week (Δ day 7–day 0; r = −0.04, p = 0.89). These findings indicate that early regenerative activation does not translate into measurable muscle mass preservation during the acute catabolic phase of critical illness.

Early satellite-cell proliferative capacity does not predict absolute muscle mass at day 7. Patients with higher early proliferation do not systematically have more preserved muscle mass one week later. This suggests that acute muscle mass at day 7 is dominated by catabolic processes (severity, immobility, oedema, proteolysis) rather than regenerative capacity.

**Supplementary Figure S5. Determinants of baseline and recovered satellite-cell proliferative capacity during critical illness.** Scatterplots illustrate relationships between satellite-cell proliferation slopes measured at ICU Day 0 and Day 7. Upper left panel shows the association between baseline proliferation and age. Upper right panel depicts the relationship between recovered proliferation at day 7 and insulin sensitivity assessed by hyperinsulinemic–euglycemic clamp (M-value). Lower left panel shows the association between recovered and baseline proliferative capacity, reflecting within-patient tracking of proliferation over time. Lower right panel illustrates the relationship between baseline proliferation and disease severity at ICU admission (SOFA score). Solid lines represent linear regression fits with shaded 95% confidence intervals. R² and p values are shown for each mode.

Baseline satellite-cell proliferation at ICU admission was inversely associated with age and disease severity, indicating that host factors and acute illness constrain early proliferative capacity. Proliferation at ICU Day 7 was strongly related to baseline proliferation, demonstrating partial conservation of proliferative capacity within individuals, but also showed substantial inter-individual variability. In contrast to baseline proliferation, recovered proliferation at Day 7 was additionally associated with metabolic status, as reflected by insulin sensitivity, suggesting that recovery of proliferative capacity integrates baseline reserve, illness severity, and anabolic support.

#### **Supplementary Table S1****: Individual-level satellite cell proliferation slopes**

Proliferation slopes represent the rate of change in the proportion of G1-phase cells measured at 24, 48, 72, and 96 hours. All data are means from technical tri or quadruplicates.

| **Group** | **Subject ID** | **Slope** |  |  |
| --- | --- | --- | --- | --- |
| Control | C01 | 0.163 |  |  |
| Control | C02 | 0.223 |  |  |
| Control | C03 | 0.177 |  |  |
| Control | C04 | 0.373 |  |  |
| Control | C05 | 0.150 |  |  |
| Control | C06 | 0.534 |  |  |
| Control | C07 | 0.159 |  |  |
| Control | C08 | 0.229 |  |  |
| Control | C09 | 0.182 |  |  |
| Control | C10 | 0.563 |  |  |
| Control | C11 | 0.128 |  |  |
| Control | C12 | 0.307 |  |  |
| Control | C13 | 0.306 |  |  |
| Control | C14 | 0.162 |  |  |
| Control | C15 | 0.199 |  |  |
| Control | C16 | 0.323 |  |  |
| Control | C17 | 0.165 |  |  |
| Control | C18 | 0.366 |  |  |
| Control | C19 | 0.196 |  |  |
| **Group** | **Subject ID** | **Day 0** | **Day 7** | **Day 180** |
| ICU | P01 | 0.15 | 0.22 | 0.16 |
| ICU | P02 | 0.19 | 0.18 | – |
| ICU | P03 | 0.23 | 0.14 | – |
| ICU | P04 | 0.14 | 0.10 | – |
| ICU | P05 | 0.14 | 0.16 | – |
| ICU | P06 | 0.21 | 0.22 | – |
| ICU | P07 | 0.13 | 0.12 | – |
| ICU | P08 | 0.25 | 0.22 | – |
| ICU | P09 | 0.15 | 0.13 | – |
| ICU | P10 | 0.21 | 0.17 | – |
| ICU | P11 | 0.13 | 0.17 | – |
| ICU | P12 | 0.31 | 0.38 | – |
| ICU | P13 | 0.34 | 0.40 | 0.52 |
| ICU | P14 | 0.17 | 0.23 | – |
| ICU | P15 | 0.50 | 0.55 | – |
| ICU | P16 | 0.13 | 0.12 | 0.16 |
| ICU | P17 | 0.16 | 0.15 | – |
| ICU | P18 | 0.15 | 0.11 | – |
| ICU | P19 | 0.15 | 0.13 | – |
| ICU | P20 | 0.39 | 0.58 | 0.20 |

Missing values indicate missing value, due to unavailable or technically unsuitable samples.

## Mitochondrial content in SC and ROS production

**Supplementary Figure S6.** **Mitochondrial content and reactive oxygen species (ROS) production.** **(a)** and **(b)** protein expression of Tom20 and Tom40, and **(c)** mitochondrial footprint (from “MINA” morphology analysis) representing mitochondrial mass; **(d)** Representative confocal microscopy images of satellite cells from controls and ICU patients, stained with NucBlue (nuclei, blue), MitoTracker Green (mitochondrial mass, green), and MitoSOX (ROS production, red). Merged images are shown in the bottom row. Scale bar: 100 μm. **(e)** Quantification of ROS production (MitoSOX/MitoTracker Green ratio). Data are presented as box-and-whisker plots (median, interquartile range, and individual data points). Note: a.u., arbitrary units, MTG, MitoTracker Green, ROS, reactive oxygen species.

## Mitochondrial morphology in SC

**Supplementary Figure S7. Mitochondrial network morphology in control cells and cells obtained from ICU patients over time.** Representative confocal images of mitochondrial staining in cells from healthy controls and ICU patients at Day 0, Day 7, and Day 180. Top row: representative raw confocal images. Middle row: segmented images used to identify mitochondrial objects and connected networks. Bottom row: skeletonized reconstructions used for analysis. Scale bar = 10 µm.

### Protein expression analyses (Western blots)

**Supplementary Figure S8.** **Protein expression analyses of (a)** Mitofusin 1, **(b)** Mitofusin 2, **(c)** OPA1-L, **(d)** OPA1-S, **(e)** P-DRP1(S637), **(f)** P-DRP1(S637)/total DRP1, **(g)** P-DRP1(S616), **(h)** P-DRP1(S616)/total DRP1, **(i)** Lipin 1, **(j)** Fatty acid synthase, **(k)** P-AMPK, **(l)** P-ATP citrate lyase(Ser455), **(m)** ATP citrate lyase.

### Correlation analysis – selected key associations

**Supplementary Figure S9. Satellite cell proliferation rate - Spearman correlations with age, APACHE II, SOFA, and MRC score.**

**Supplementary Figure S10.** **Mitochondrial morphology vs. SC proliferation and maximal respiratory capacity (ICU Day 0).**

**Supplementary Figure S11. Western blot proteins vs. SC proliferation rate (ICU Day 0).**

### Day 180 survivors versus non-survivors (per-protocol cohort, n = 23)

#### **Supplementary Table S2: Functional and muscle structure parameters at day 7**

| **Variable** | **Survivors at day 180**  **(n=10)** | **Non-survivors at day 180**  **(n=13)** | **p-value** |
| --- | --- | --- | --- |
| **MRC score (Day 7)** | 34 [27-48] | 0 [0-1] | 0.001 |
| **Rectus femoris CSA (µm^2^; Day 7)** | 318 [254-392] | 259 [173-301] | 0.12 |
| **Type I fibre CSA (µm^2^; Day 7)** | 1105 [964-1351] | 1092 [836-1341] | ˃0.9 |
| **Type II fibre CSA (µm^2^; Day 7)** | 998 [903-1184] | 1078 [761-1287] | 0.8 |
| **Type I fibre abundance (%; Day 7)** | 52 [44-65] | 46 [37-57] | 0.2 |
| **Type II fibre abundance (%; Day 7)** | 48 [35-56] | 54 [43-63] | 0.2 |
| **M-value (mg/kg/min; Day 7)** | 4.56 [3.94-5.93] | 4.02 [2.61-5.06] | 0.5 |

Data are reported as median [IQR].

#### **Supplementary Table S3: Satellite cell biology**

| **Variable** | **Survivors at day 180**  **(n=10)** | **Non-survivors at day 180**  **(n=13)** | **p-value** |
| --- | --- | --- | --- |
| **SC number per fibre (Day 0)** | 0.09 [0.06-0.11] | 0.10 [0.05-0.14] | 0.5 |
| **SC number per fibre (Day 7)** | 0.12 [0.07-0.15] | 0.08 [0.06-0.10] | 0.2 |
| **SC proliferation slope (Day 0)** | 0.19 [0.15-0.31] | 0.15 [0.14-0.21] | 0.4 |
| **SC proliferation slope (Day 7)** | 0.22 [0.18-0.38] | 0.15 [0.13-0.17] | 0.081 |
| **Fusion index (%, Day 0)** | 72 [68-82] | 67 [59-71] | 0.030 |
| **Fusion index (%, Day 7)** | 67.9 [64.9-72.4] | 72.4 [67.7-74.8] | 0.4 |

Data are reported as median [IQR].

#### **Supplementary Table S4: Mitochondrial morphology and bioenergetics**

| **Variable** | **Survivors at day 180**  **(n=10)** | **Non-survivors at day 180**  **(n=13)** | **p-value** |
| --- | --- | --- | --- |
| **Mito individuals (Day 0)** | 28 [18-46] | 34 [27-56] | 0.3 |
| **Mito individuals (Day 7)** | 30 [18-32] | 33 [28-49] | 0.2 |
| **Mito network size median (Day 0)** | 6.9 [6.0-10.3] | 5.6 [4.9-10.3] | 0.3 |
| **Mito network size median (Day 7)** | 12.19 [5.78-13.69] | 6.84 [5.62-8.14] | 0.2 |
| **Mito branch length median (Day 0)** | 2.03 [1.84-2.19] | 2.04 [1.99-2.08] | ˃0.9 |
| **Mito branch length median (Day 7)** | 2.13 [2.01-2.35] | 2.11 [2.04-2.14] | 0.5 |
| **Normalised max respiration (Day 0)** | 303 [262-492] | 363 [220-452] | 0.9 |
| **Normalised max respiration (Day 7)** | 335 [249-446] | 296 [221-371] | 0.6 |
| **Normalised ATP production (Day 0)** | 175 [139-249] | 150 [126-223] | 0.6 |
| **Normalised ATP production (Day 7)** | 197 [189-202] | 139 [116-208] | 0.4 |
| **Coupling efficiency (Day 0)** | 83.04 [80.04-84.66] | 83.80 [82.48-84.80] | 0.6 |
| **Coupling efficiency (Day 7)** | 85.0 [83.8-86.6] | 84.9 [80.9-86.1] | 0.5 |

Data are reported as median [IQR].

### Key satellite cell and clinical parameters stratified by Day 180 survival

**Supplementary Figure S12.** **Key satellite cell and clinical parameters stratified by Day 180 survival (per-protocol ICU cohort,n=23).** Box plots show median and IQR with individual points overlaid.

### Per-protocol versus enrolled without Day 7 biopsy

#### **Supplementary Table S5: Baseline characteristics and severity**

| **Variable** | **Per-protocol (D0+D7 biopsy)**  **(n=23)** | **Enrolled, no D7 biopsy**  **(n=10)** | **p-value** |
| --- | --- | --- | --- |
| **Age (years)** | 56 [42-64] | 58 [52-72] | 0.3 |
| **Sex: Female** | 2 (8.7 %) | 4 (40 %) |  |
| **BMI (kg/m^2^)** | 28 [26-34] | 25 [23-34] | 0.3 |
| **Admission diagnosis**  **Multiple trauma**  **Traumatic brain injury**  **Sepsis**  **Other** | 8 (35 %)  10 (43 %)  3 (13 %)  2 (8.6 %) | 4 (40 %)  5 (50 %)  0 (0 %)  1 (10 %) | 0.7 |
| **APACHE II (admission)** | 17.0 [15.0-21.0] | 20.5 [18.0-21.0] | 0.3 |
| **SOFA (Day 0)** | 10.00 [7.00-12.00] | 8.50 [8.00-11.00] | 0.6 |

Data are reported as median [IQR].

### SOFA and APACHE II from Day 0 to Day 7

**Supplementary Figure S13. Individual SOFA and APACHE II trajectories from Day 0 to Day 7 in ICU patients (n = 23).** Lines connect within-patient measurements; bold points are group medians.

# References:

1. Parstorfer M, Profit F, Weiberg N, Wehrstein M, Barié A, Friedmann-Bette B. Increased satellite cell apoptosis in vastus lateralis muscle after anterior cruciate ligament reconstruction. J Rehabil Med. 2021 Feb 17;53(2):jrm00153. doi: 10.2340/16501977-2794.
2. Spinazzola J, Gussoni E. Isolation of Primary Human Skeletal Muscle Cells. Bio Protoc. 2017;7. doi:10.21769/bioprotoc.2591.
3. Krajcova A, Ziak J, Jiroutkova K, Patkova J, Elkalaf M, Dzupa V, et al. Normalizing glutamine concentration causes mitochondrial uncoupling in an in vitro model of human skeletal muscle. JPEN J Parenter Enteral Nutr. 2015;39(2):180-9. doi: 10.1177/0148607113513801.
4. Agley CC, Rowlerson AM, Velloso CP, Lazarus NL, Harridge SDR. Isolation and quantitative immunocytochemical characterization of primary myogenic cells and fibroblasts from human skeletal muscle. Journal of Visualized Experiments. 2015. doi:10.3791/52049.
5. Murphy DP, Nicholson T, Jones SW, O'Leary MF. MyoCount: a software tool for the automated quantification of myotube surface area and nuclear fusion index. Wellcome Open Res. 2019 Jan 21:4:6. doi: 10.12688/wellcomeopenres.15055.1.
6. Ferrick DA, Neilson A, Beeson C. Advances in measuring cellular bioenergetics using extracellular flux. Drug Discov Today. 2008;13: 268–274. doi:10.1016/j.drudis.2007.12.008.
7. [XF Substrate Oxidation Stress Test Kits User Manual](https://hpst.cz/sites/default/files/oldfiles/user-manual-substrate-oxidation-stress-test-kits-cell-analysis-5994-1164en-agilent.pdf). Available at <https://hpst.cz/sites/default/files/oldfiles/user-manual-substrate-oxidation-stress-test-kits-cell-analysis-5994-1164en-agilent.pdf>
8. Poot M, Zhang YZ, Krämer JA, et al. Analysis of mitochondrial morphology and function with novel fixable fluorescent stains. J Histochem Cytochem. 1996;44(12):1363–1372. doi: 10.1177/44.12.8985128.
9. Doherty E, Perl A. Measurement of mitochondrial mass by flow cytometry during oxidative stress. React Oxyg Species. 2017;4(10):275–283. doi: 10.20455/ros.2017.839.
10. Valente AJ, Maddalena LA, Robb EL, Moradi F, Stuart JA. A simple ImageJ macro tool for analyzing mitochondrial network morphology in mammalian cell culture. Acta Histochem. 2017 Apr;119(3):315-326. doi: 10.1016/j.acthis.2017.03.001.
